# Medical decision support system using weakly-labeled lung CT scans

**DOI:** 10.3389/fmedt.2022.980735

**Published:** 2022-09-28

**Authors:** Alejandro Murillo-González, David González, Laura Jaramillo, Carlos Galeano, Fabby Tavera, Marcia Mejía, Alejandro Hernández, David Restrepo Rivera, J. G. Paniagua, Leandro Ariza-Jiménez, José Julián Garcés Echeverri, Christian Andrés Diaz León, Diana Lucia Serna-Higuita, Wayner Barrios, Wiston Arrázola, Miguel Ángel Mejía, Sebastián Arango, Daniela Marín Ramírez, Emmanuel Salinas-Miranda, O. L. Quintero

**Affiliations:** ^1^ Department of Mathematical Sciences, Universidad EAFIT, Medellín, Colombia; ^2^ Radiology Department, Universidad CES, Medellín, Colombia; ^3^ Radiology Department, Universidad de Antioquia, Medellín, Colombia; ^4^ Institución Prestadora de Servicios de Salud IPS Universitaria, Medellín, Colombia; ^5^ Instituto Tecnológico Metropolitano, Medellín, Colombia; ^6^ Wiqonn Technologies, Barranquilla, Colombia; ^7^ Lunefield Institute, Mount Sinai, Toronto, Canada

**Keywords:** computational tomography, weak-labels, volume classification, lesion segmentation / quantification, machine learning

## Abstract

**Purpose:**

Determination and development of an effective set of models leveraging Artificial Intelligence techniques to generate a system able to support clinical practitioners working with COVID-19 patients. It involves a pipeline including classification, lung and lesion segmentation, as well as lesion quantification of axial lung CT studies.

**Approach:**

A deep neural network architecture based on DenseNet is introduced for the classification of weakly-labeled, variable-sized (and possibly sparse) axial lung CT scans. The models are trained and tested on aggregated, publicly available data sets with over 10 categories. To further assess the models, a data set was collected from multiple medical institutions in Colombia, which includes healthy, COVID-19 and patients with other diseases. It is composed of 1,322 CT studies from a diverse set of CT machines and institutions that make over 550,000 slices. Each CT study was labeled based on a clinical test, and no per-slice annotation took place. This enabled a classification into Normal vs. Abnormal patients, and for those that were considered abnormal, an extra classification step into Abnormal (other diseases) vs. COVID-19. Additionally, the pipeline features a methodology to segment and quantify lesions of COVID-19 patients on the complete CT study, enabling easier localization and progress tracking. Moreover, multiple ablation studies were performed to appropriately assess the elements composing the classification pipeline.

**Results:**

The best performing lung CT study classification models achieved 0.83 accuracy, 0.79 sensitivity, 0.87 specificity, 0.82 F1 score and 0.85 precision for the Normal vs. Abnormal task. For the Abnormal vs COVID-19 task, the model obtained 0.86 accuracy, 0.81 sensitivity, 0.91 specificity, 0.84 F1 score and 0.88 precision. The ablation studies showed that using the complete CT study in the pipeline resulted in greater classification performance, restating that relevant COVID-19 patterns cannot be ignored towards the top and bottom of the lung volume.

**Discussion:**

The lung CT classification architecture introduced has shown that it can handle weakly-labeled, variable-sized and possibly sparse axial lung studies, reducing the need for expert annotations at a per-slice level.

**Conclusions:**

This work presents a working methodology that can guide the development of decision support systems for clinical reasoning in future interventionist or prospective studies.

## 1. Introduction

Artificial Intelligence (AI) models can be implemented to process and extract knowledge from medical data. As stated in Quintero (1), *“There is no ambiguity that a machine will never replace an MD expert, but machine intelligence will benefit and its aimed for human decision making*.” Following such line, this work introduces a medical decision support system deployed to help doctors treating COVID-19 patients in underdeveloped countries.

COVID-19 is a disease caused by the SARS-CoV-2 virus (2). Different authors have evaluated chest Computed Tomography (CT) studies^1^ to identify changes in the lung suggestive of COVID-19. The main radiological patterns identified and associated to COVID-19 pneumonia are peripheral ground glass or nodular-type opacities, which have been seen in both symptomatic and asymptomatic patients (4); although other findings that may resemble other infectious or non-infectious processes have also been described (5). Nonetheless, the current gold-standard diagnostic test for this disease is the Polymerase Chain Reaction (PCR) (6). Therefore, from a clinical point of view, it is important to develop a clinical decision support system that allows health personnel to take early measures regarding the treatment of patients suspected of having COVID-19 infection, given that the result of the PCR test might take several days, the test’s false negative rate is reported to range from 2% up to 29% (7) and the healthcare systems collapse during peak infection rates.

Our approach to the problem is holistic. Developing solutions is not just training a preconceived convolutional/deep network, neither machine learning is just a matter of a toolbox (1). It requires interdisciplinary work and mathematical background to imagine and formalize a proper feature extractor closest to the human perception of the world. Consequently, the first step is to develop a feasibility study testing several learning machines for CT while acquiring images under a standardized protocol for image acquisition. Secondly, a retrospective study of the performance of the learning machines and finally, the prospective study validated on line by medical experts.

Concretely, the contributions of this paper are:

1. Introduction of an architecture to classify *weakly-labeled*, *variable-sized* (and possibly *sparse*) lung CT volumes. This ensures that the complete volume is taken into account before returning a diagnosis. It is based on DenseNet and has been chosen given that it outperforms other methodologies to classify weakly-labeled lung CT scans.
2. Description of a *deployed* implementation for a medical diagnosis pipeline (decision support system) involving *classification*, *segmentation* and *lesion quantification* tasks using axial lung CT scans.

The remaining of this article is organized as follows. Section 2 gives an overview of the related work. Section 3 covers a detailed description of the pipeline and its components, as well as a description of the data sets employed. Section 4 presents the main findings. Section 5 discusses the results. Finally, Section 6 goes over the conclusions of this work.

## 2. Related work

One of the challenges faced by a system handling CT scans from multiple sources with a diverse set of patients (age, gender, comorbidities, etc.) is that all the registrations will vary in resolution, depth and position. Image registration involves a set of techniques to minimize these challenges. In (8) it is defined as the process of transforming different image data sets into one coordinate system with matched imaging content, and in the same document they provide an overview of recently proposed solutions, among which they exhibit the case of CT scan, of different body parts, including chest and lungs. Some of them involve supervised, unsupervised and weakly supervised methodologies, with a CNN as the common factor among all.

Once images can be considered homogenized, the classification phase can start. Most works involve a granular process. For example, Kavitha et al. (9) propose a ML pipeline to determine lung cancer stage. The authors propose a region based Fuzzy C-Means Clustering technique for segmenting the lung cancer region and a Support Vector Machine based classifier to diagnose the cancer stage from the segmented regions. They also argue that CT scans are a good source of information since they poses higher image sensitivity and resolution with good isotopic acquisition. This pipeline achieves a classification accuracy of 93% for 70 training samples. Another work by Huang et al. (10) focused on lung nodule diagnosis on CT images. They wanted to determine if a nodule was benign or malignant. For that, they exploit a pretrained CNN on the ImageNet data set, to extract high level features. After that, an Extreme Learning Machine is used to classify the features returned by the CNN. The authors also recount what has traditionally been done in this field to tackle similar problems. According to them it involves: (1) feature extraction using hand crafted segmentation algorithms, (2) feature recognition using one of the traditional ML algorithms, and (3) producing a diagnosis taking into account the whole CT images according to their previous characterization. Such approach does not take advantage of the volumetric information present in a CT. Xie et al. (11) gives another example, where a semi-supervised adversarial classification model is trained using both labeled and unlabeled data for benign-malignant lung nodule classification.

The surge of COVID-19 cases has prompted a handful of investigations regarding CT scans for case diagnosis. Most of this works use methods derived from DL, many of which leverage supervised learning. Nonetheless, there is a drawback with most approaches. This is due to the fact that they require labeled slices, which is an expensive and time consuming procedure that can only be carried out by expert radiologists (12–23). On the other hand, other works do not employ the complete CT volume to return a diagnosis, which might decrease the model’s real-world performance, where it is possible to see regions with little or no patterns in the lung suggesting COVID-19 (12,13,16,20,22,24). Furthermore, there are approaches that only use a single slice, or process the volume slice-by-slice, which means that they might lose relevant 3D information (13–15,17,18,21–24). Table 1 shows a comparison of the classification performance of this methods.

**Table 1:** Performance comparison among COVID-19 lung CT classification models, as reported by the authors, on their test sets. Where multiple classifiers were tested, the results without data augmentation are reported.

Model	Accuracy (%)	Sensitivity (%)	Specificity (%)	Labeled slices	Support
(15)	90.11	87.03	96.60	Yes	3,199
(16)	96.00	**100.0**	96.00	Yes	1,100
(20)	**99.87**	99.58	**100.0**	Yes	799
(21)	79.30	67.00	83.00	Yes	290
(23)	96.00	94.00	98.00	Yes	270
(22)	98.00	94.96	98.70	Yes	245
(18)	85.00	85.40	85.70	Yes	203
(13)	90.23	91.18	89.23	Yes	133
(14)	96.00	95.00	96.00	Yes	119
(19)	86.70	86.60	86.80	Yes	90
(24)	81.00	80.20	82.60	**No**	45

The following is a quick description of some of the most successful DL approaches referenced above. Zhang et al. (12) developed a two-stage segmentation framework for accurately segmenting lung lesions and background on raw CT slices. Then, they stacked the slices to form a volume that was preprocessed and then fed to a 3D classification network. Bai et al. (14) train a classification model to distinguish between slices with and without pneumonia-like findings. The model is based on the EfficientNet architecture and is reported to have better results than radiologists. In Jin et al. (15) a COVID-19 diagnosis system is developed. It achieves a remarkable performance when tested on over 3000 CT volumes, but they used manually labeled slices. Finally, Hu et al. (24) use weakly supervised DL for infection detection. Basically they use a segmentation network based on U-Net which returns a structure which will later be used to classify, each slice, based on the VGG architecture. This particular model achieved 81.0% accuracy classifying CT slices.

## 3. Pipeline for weakly-labeled lung CT scans

**Figure 1:**
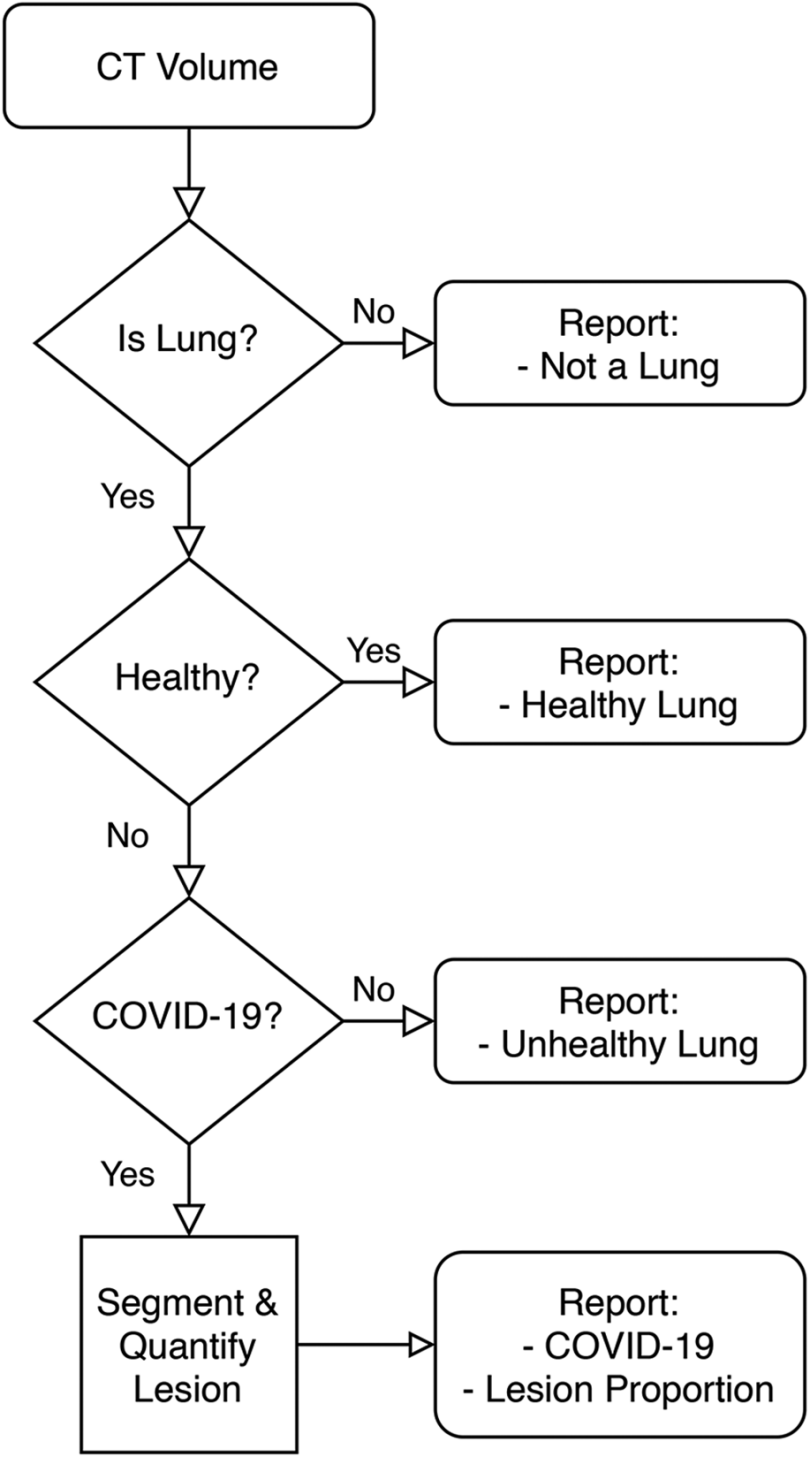
High-level overview of the Decision Support System.

Figure 1 is a high-level overview of the components of the developed Medical Diagnosis Pipeline. The pipeline takes an *axial* CT study and preprocesses it to remove images that do not contain portions of a lung. Then, this preprocessed volume is used to classify the patient. Finally, if the patient is determined to belong to the COVID-19 class, the volume is fed to a lesion segmentation network to highlight and quantify the lesion. The following is an in-depth description of each component.

### 3.1. Lung segmentation

This model is used for three sub-tasks:

1. *Lung Detection*. Using the segmentation map it is possible to determine if each CT slice contains a portion of a lung. If it does, the image is kept.
2. *Bounding Box Segmentation*. Once all non-lung slices are removed, the lung portion in each slice is cropped about its model-given bounding box.
3. *Lung Area Quantification*. The non-zero pixels in the segmentation map are counted to determine an approximate lung area in each slice.

The architecture employed is U-Net (25). This segmentation network was designed to extract context using a contracting path, and to enable precise localization via a symmetric expanding path. Therefore, it performed well on sequences of images where variation was due to a changing lesion pattern and lung shape. The model used DenseNet-121 (26), pretrained on ImageNet, as encoder. The loss function was the MSE between the predicted and target lung bounding box. The optimizer was Adam and it was trained for 18 epochs using batches of size 8. The model achieved a 0.98 IoU score on test data. U-Net was also used in other works related to COVID-19 lung segmentation, such as (15,24).

### 3.2. Lung classification

**Figure 2:**
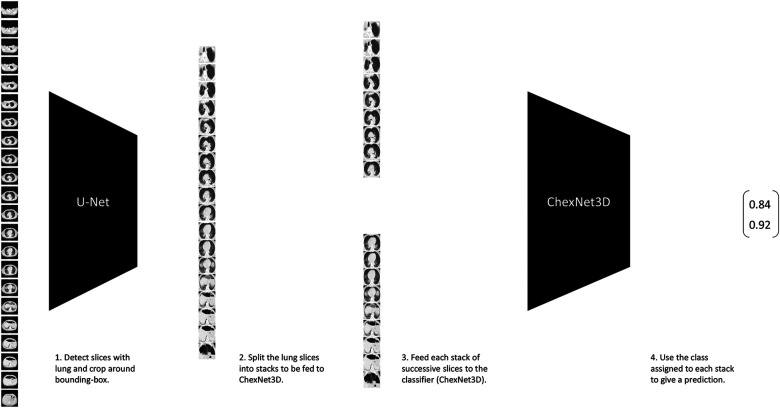
Lung CT classification pipeline. It takes stacks of n successive slices from the CT volume, and inputs them to the proposed ChexNet3D. Before that, the study is fed to U-Net to select the slices that contain lung portions and segment around the lung’s bounding box.

Classification of lung CT volumes was a challenge, since the data was weakly-labeled according to a gold standard test or a doctor’s diagnostic, which means that *none* of the CT slices were individually annotated by an expert. Furthermore, the studies vary in size due to a number of factors (machine capabilities, predefined settings, person’s height, etc.), and that it was sparse. To solve this, the ChexNet3D architecture is introduced. It is a deep learning (27) model composed of a series of successive mathematical transformations for an input tensor that describes the grayscale-values of each pixel of each slice (resized to 256×256 pixels) from the CT study, which results in a 2×1 vector describing the probability that the stack of successive images reconstructing the lung belongs to a class (the position of the value with higher weight in the output vector is considered the class predicted for the input). It is inspired on the work by Rajpurkar et al. (28) with RX images. It exploits the fact that neighboring slices contain similar lesion patches, both in position and shape. Some examples that have successfully used such prior on CT studies are (19,29). Figure 2 gives an overview of the classification pipeline.

The proposed model uses DenseNet-121 as backbone (pretrained on ImageNet), but the first layer is replaced. Specifically, in place of the first layer, two new convolutional layers are added. The first one takes a stack of *n* grayscale images (one-channel each), and outputs a volume with 64-channels. The second (new) layer takes the previous 64-channel volume and outputs another 64-channel volume. The output of this second layer is then fed as input to the second layer of the original DenseNet-121 backbone. On top of this network, a dense layer with softmax activation is used to get a class for the stack that was originally fed. An architecture based on DenseNet-121 presented multiple advantages such as alleviation of vanishing-gradients, stronger feature propagation, with less trainable parameters and reduced computational requirements, as shown in Huang et al. (26). Also, the higher connectivity allows feature reuse via identity mappings, deep supervision and diversified depth as the authors showed in their manuscript. Furthermore, as mentioned before, works such as Rajpurkar et al. (28) have already shown important results including a better F1 performance compared to expert radiologists in the task of chest X-ray classification, which although is not directly transferable to the axial lung CT scan domain, is a relevant proxy for the capabilities of the model for our task.

The new convolutional layers use a 7×7 kernel with stride 2, and were initialized by randomly sampling from a Normal distribution with mean 0 and variance 0.1. The cost function is the Cross Entropy Loss. Optimization took place using the Adam optimizer with learning rate $3\times 10^{-3}$. The parameters were estimated via transfer learning. Specifically, training was only performed on the first two (new) and the last twentyfive layers. This allowed us to reuse the feature maps obtained by the model pretrained on ImageNet and decrease the computational requirements at training time, which resulted in larger batch sizes and enabled the use of all lung slices from the CT study for training. The batch size was of 6 randomly chosen studies; but, since each study was split on stacks of 30 successive slices, the network effectively saw more than 6 inputs per parameter update. The stacks within each random batch were shuffled to guarantee that successive regions of the whole lung volume were not contiguous before being fed to the network. Training took place until the performance on the validation data set decreased for two successive epochs, as a signal of overfitting to the training data. The final model was initialized with the parameter set from the training epoch with best performance on the validation data.

To classify the complete CT study, the volume obtained after removing slices that do not contain portions of the lung (as described in Section 3.1), is splitted into successive stacks of n grayscale-slices. When the number of slices in this volume is not a multiple of n, the last stack uses slices from the previous stack to complete the n-slices volume. If the whole volume contains less than n slices, the volume is completed with black images. Each stack is given the same label of the CT study. Accuracy and *At Least One* were the metrics used during training to assess the performance on the validation data set. But, during inference and evaluation on the test set, different metrics are used (accuracy-per-stack and average of the stacks in the study). The *At Least One* metric, as the name suggests, assigns the class of interest to the whole study when at least one stack from the study is classified as belonging to such class. Therefore, to be classified as not-in-the-class of interest, all the stacks need to be classified in that other class.

For instance, if the classifier is determining if a patient’s lung is normal/healthy or unhealthy, and the class of interest is *unhealthy*, then the patient is classified as healthy only if all stacks are determined to be “healthy.” In contrast, the patient is classified as unhealthy if *at least one* of the stacks is determined to be “unhealthy.” The idea behind the *At Least One* metric is to be able to correctly classify those cases where sparse data is present. For example, a patient’s CT study might show COVID-19-related lesions only on a small portion of the lung, and the remaining portions could seem “healthy.” Therefore, this metric, when the classifier finds the ‘COVID-19’ portion, will guarantee an accurate classification. It also leads to a high level of sensitivity when the model is learning irrelevant patterns, so it forces the model to adequately learn all classes to achieve a balance with the specificity.

### 3.3. Lesion segmentation

Lungs that are classified as belonging to the COVID-19 class are then fed into a lesion segmentation network to obtain a lesion map. The architecture implemented in this work is Inf-Net (30). Particularly, the semi-supervised approach is used. The same network and data set presented by the authors is employed. Inf-Net features a parallel partial decoder to aggregate high-level features and generate a global lesion map, which is later enhanced using implicit reverse attention and explicit edge-attention.

A drawback of this network is that supervised training took place on a small data set that mostly contained segmented samples around the center of the volume. This leads to segmentation performance degradation in the slices towards the start and the end of the volume, where the lung is smaller and has a different shape. To solve this, the network’s output is resized to the size of the lung segmentation mask (Section 3.1) and the coordinates of the lung’s bounding-box are applied to the resized lesion mask. This leads to better performance on small and diversely-shaped lung portions.

### 3.4. Lesion quantification

This procedure is only carried out on those patients that were previously classified as COVID-19. It involves the lung segmentation mask obtained in Section 3.1 and the lesion segmentation mask obtained in Section 3.3. The first mask is used to count the number of non-zero pixels (A), and this is taken as the lung’s area in that slice. Then, the number of non-zero pixels (a) in the lesion mask of that slice are also counted. Finally, the approximate quantification of the lesion’s area for each slice is L=a/A.

### 3.5. Data sets

A data set of axial CT studies was aggregated from (31–44). It was split in three main categories: Healthy, Unhealthy and COVID-19 cases. Figure 4C, shows the distribution of the three. In total there are 6,439 studies, composed of over 400,000 slices. The Unhealthy class includes patients with cancer, pneumonia, tumor, atelectasis, adhesion, effusion, fibrosis, nodules, adenocarcinomas and ephysemas.

**Figure 3:**
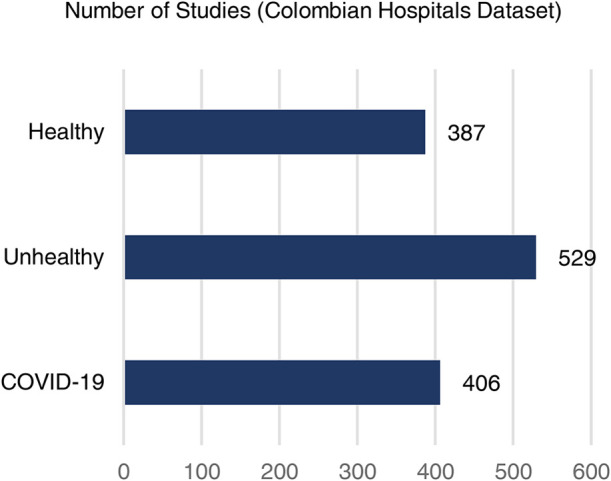
Data distribution of the CT studies collected from Colombian medical institutions.

Also, a data collection protocol was established with the following Colombian medical institutions: Clinica CES, IPS Universitaria, Hospital Pablo Tobón Uribe / Hospital San Vicente Fundación. The data was obtained, in DICOM format, from patients from those institutions that gave their consent and it was anonymized by a medical professional. Then, they were distributed to the modelling team, where it was cleaned (removing non-axial studies, etc.) and saved as a PNG image using the lung window (−600 up to 1,500 in Hounsfield units). In total there are 1,322 studies with over 550,000 slices. Figure 3 shows the data distribution.

During training, the data sets were shuffled and balanced according to the category with less samples. The data was divided as follows: 70% training, 15% validation and 15% testing.

## 4. Main findings

This section covers the experiments that were carried out and the results from each of them. It includes the results obtained from implementing the proposed ChexNet3D architecture for the task of lung CT classification with weak labels. It will also go over the findings, and show some examples, regarding the lesion segmentation and quantification methodology. Finally, via a series of ablation studies, it will be possible to see how different design decisions affect the classification pipeline’s performance.

### 4.1. Lung classification

The proposed classification architecture can be employed to tackle weakly-supervised tasks in Computer Vision. Its performance on the test set, as shown in Table 2, is above other weakly-supervised methodologies (last row of Table 1), and is similar to other models where all slices are expertly-labeled (Table 1).

**Table 2:** Performance comparison, on test data, of weakly-labeled lung CT classification tasks, using the proposed architecture (ChexNet3D).

Model	Accuracy (%)	Sensitivity (%)	Specificity (%)	F1 score (%)	Precision (%)	Support
Healthy vs. unhealthy^a^	71.00	67.00	76.00	71.00	75.00	1,372
Other diseases vs. COVID-19^a^	75.00	65.00	82.00	68.00	71.00	1,445
Healthy vs. unhealthy^b^	83.00	79.00	87.00	82.00	85.00	117
Other diseases vs. COVID-19^b^	86.00	81.00	91.00	84.00	88.00	122
Healthy vs. unhealthy^c^	84.00	92.00	74.00	85.00	79.00	364
Other diseases vs. COVID-19^c^	90.00	99.00	81.00	91.00	84.00	759

The Supplementary Material contains tables and figures with additional relevant performance metrics and figures.

^a^ Results on hospitals data set with accuracy-per-stack metric.

^b^ Results on hospitals data set with stacks-average metric.

^c^ Results on the model pre-trained with public data using the at-least-one metric.

Since this classification architecture splits the volume, training and inference is significantly faster and less computationally expensive. This aspect is also noted by Jin et al. (15), who suggest that other 3D settings are limited by GPU memory. The convolutional kernels employed in the first two layers of the network are two-dimensional. A 3D convolutional kernel was tried, but as shown in Section 4.3 the network’s performance diminished.

To classify the patients, a hierarchy of models is developed to progressively reach a diagnosis. The first model (*Healthy vs. Unhealthy*) determines if the patient’s lung is healthy or unhealthy (85% precision). If it is unhealthy, the same input is then fed to a model (*Other Diseases vs. COVID-19*) that determines if the lesions on the lung suggest COVID-19 or any other disease (88% precision). The models were trained on the public data sets and then fine-tuned on the smaller (Colombian institutions) data set.

**Figure 4:**
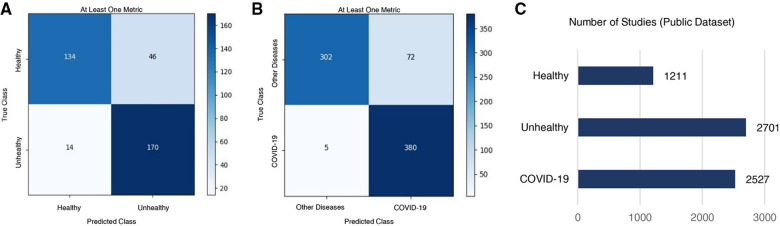
Classification results on the public test set. Confusion matrices for (**A**) healthy vs. unhealthy and (**B)** other-diseases vs. COVID-19 classifiers. (**C**) Per-class data distribution.

**Figure 5:**
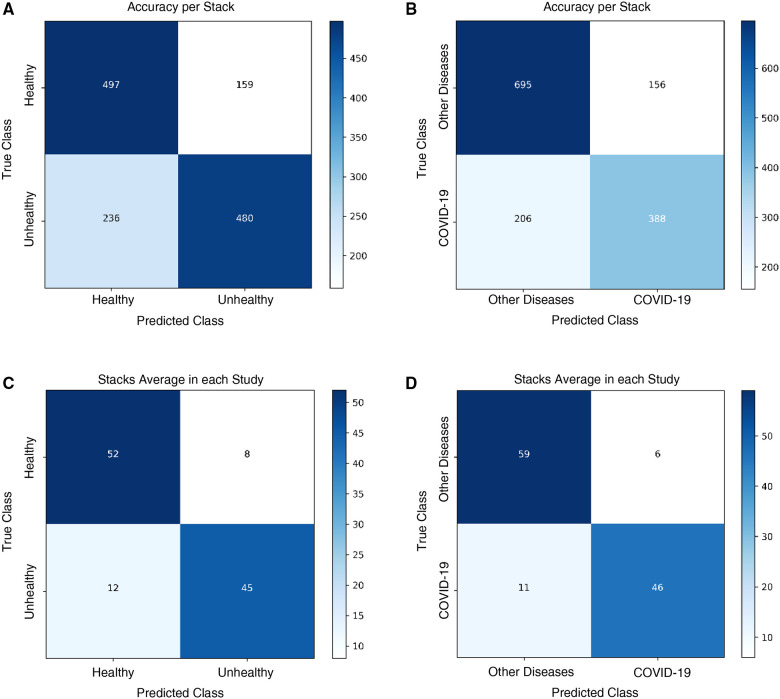
Classification results on the Colombian medical institutions test set. Confusion matrices for the (**A**) healthy vs. unhealthy classifier with the accuracy-per-stack metric, (**B**) other diseases vs. COVID-19 classifier with the accuracy-per-stack metric, (**C**) healthy vs. unhealthy classifier with the stacks-average metric, and (**D**) other diseases vs. COVID-19 classifier with the stacks-average metric.

The Healthy vs. Unhealthy model’s sensitivity, specificity, accuracy and F1 score—on the test set—is 0.92, 0.74, 0.84 and 0.85, respectively. The test set included 180 healthy patients and 184 unhealthy patients (COVID-19 / Other Diseases). Figure 4A, shows the obtained confusion matrix on this data set. For the hospitals test set, they are 0.67, 0.76, 0.71 and 0.71 for the accuracy-per-stack metric (Figure 5A); and 0.79, 0.87, 0.83, 0.82 for the stack-average metric (Figure 5C).

For the Other Diseases vs. COVID-19 classifier, it achieved 0.99 sensitivity, 0.81 specificity, 0.90 accuracy and 0.91 F1 score on the test set. It contained 385 patients with COVID-19 and 374 patients with other diseases. Figure 4B, shows the confusion matrix built from the model’s predictions. For the hospitals test set, this values are 0.65, 0.82, 0.75 and 0.68 for the accuracy-per-stack metric (Figure 5B); and 0.81, 0.91, 0.86 and 0.84 for the stacks-average metric (Figure 5D). As can be seen in Table 1, it outperforms other weakly-supervised settings by at least 5% and is similar to approaches that have annotated slices and/or that do not use the whole volume to return a prediction.

The Supplementary Materials contain data and figures further describing the model performance with other clinically relevant metrics on multiple data sets.

### 4.2. Lesion segmentation / quantification

**Figure 6:**
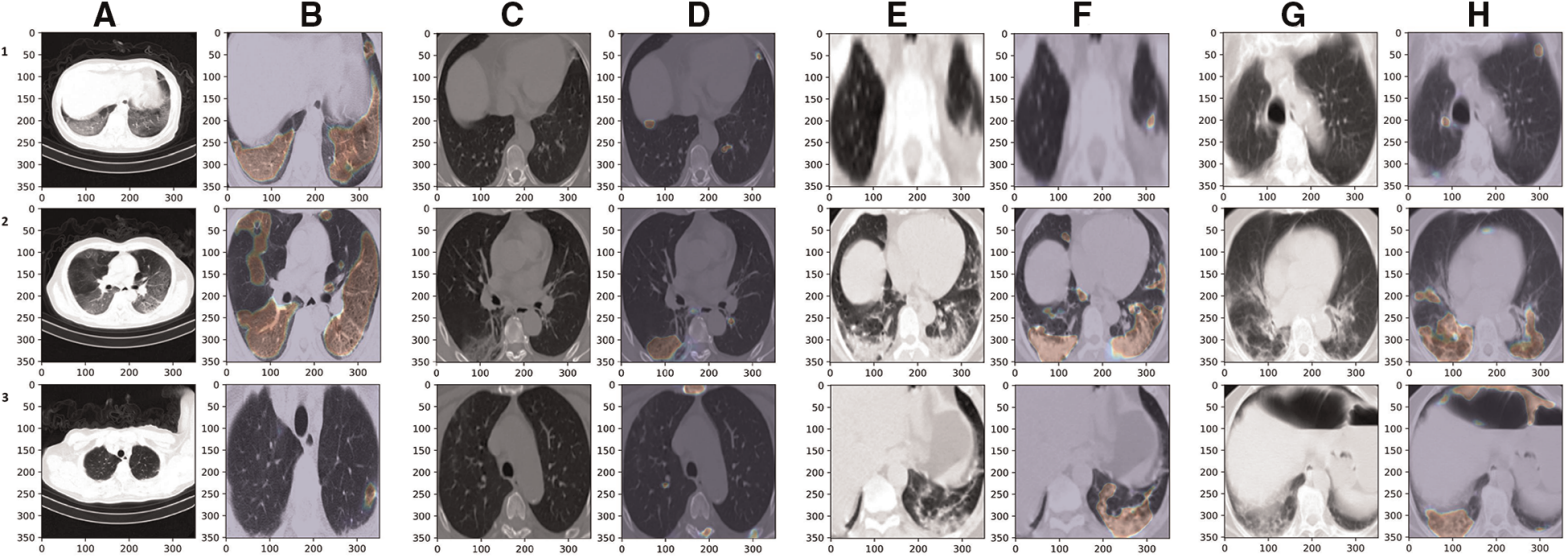
Axial CT study of four *randomly* sampled patients with COVID-19. Columns (**A**), (**C**), (**E**)/ (**G**) show the original images (columns **C**, **E**, / **G** segmented). Columns (**B**), (**D**), (**F**) / (**H**) show the bounding-box and lesion segmentation of the image at its left. Rows (**1**), (**2**) / (**3**) are different slices of the same patient at the start, middle and end of the study, respectively. The lesion quantification model determines the approximate lesion proportion, from top to bottom, as 59.11%, 56.49% / 11.30% for the figures on column (**B**); as 7.36%, 13.29% / 7.55% for those on column (**D**); as 15.61%, 40.72% / 38.11% for those on column (**F**); and as 11.73%, 27.91% / 79.97% for the slices on column (**H**). Note the patient on column (**C**), where the top and bottom slices show very little or no sign of lesions. That is the reason why it is necessary to process the complete CT volume. The Supplementary Material contains the fully processed studies for the four patients presented above.

For the lesion segmentation network, (30) report 0.73, 0.72, 0.96 for the dice, sensitivity and specificity scores, in the case of the semi-supervised network, which is the one being used here. During testing, the model has returned reliable results. Figure 6 shows on columns B, D, F / H, that the model appropriately finds the regions with lesions, even when there are significant changes in the size and shape of the lung. To achieve such performance on the diverse set of shapes and sizes, the lesion-localization-refinement setting introduced in Section 3.3 was critical. It is important to note in the patient on column C, how the top and bottom slices show very little or no sign of lesions due to COVID-19. This is possible among patients and is the reason why it is necessary to process the complete CT volume, to minimize the risk of only reviewing a portion that might led to a wrong classification. Also, Figure 6 shows multiple patients once the lung was cropped to its bounding-box and the lesions were segmented and quantified.

### 4.3. Ablation studies

This subsection covers multiple experiments that show how the pipeline’s performance changes according to different design choices. Table 3 presents the summary of the multiple experiments that were carried out and evaluated on the hospitals test data set. The specific experiments are listed below:

1. *Segmentation*. Other lung segmentation models are tried, e.g., DeepLabv3 (45).
2. *3D Convolution*. Kernels with 3-dimensions on the first layers of the ChexNet3D architecture.
3. *Reduced Volume*. Effect of reducing the lung bounds on large CT studies (≥180 lung slices).

**Table 3:** Ablation studies results.

Model	Ablation	Accuracy (%)	Sensitivity (%)	Specificity (%)	Support
Healthy vs. unhealthy^a^	Segmentation	71.00	74.00	68.00	872
Healthy vs. unhealthy^b^	Segmentation	74.00	81.00	67.00	89
Other diseases vs. COVID-19^a^	Segmentation	69.00	70.00	68.00	340
Other diseases vs. COVID-19^b^	Segmentation	74.00	68.00	81.00	43
Healthy vs. unhealthy^a^	3D Convolution	68.00	72.00	63.00	1,372
Healthy vs. unhealthy^b^	3D Convolution	78.00	81.00	75.00	117
Other diseases vs. COVID-19^a^	3D Convolution	68.00	66.00	68.00	1,445
Other diseases vs. COVID-19^b^	3D Convolution	76.00	84.00	69.00	122
Healthy vs. unhealthy^a^	Reduced Volume	72.00	66.00	79.00	1,184
Healthy vs. unhealthy^b^	Reduced Volume	83.00	75.00	90.00	117
Other diseases vs. COVID-19^a^	Reduced Volume	75.00	65.00	82.00	1,265
Other diseases vs. COVID-19^b^	Reduced Volume	86.00	81.00	91.00	122
Healthy vs. unhealthy^a^	3D Conv. + Red. Volume	69.00	68.00	70.00	1,184
Healthy vs. unhealthy^b^	3D Conv. + Red. Volume	75.00	77.00	73.00	117
Other diseases vs. COVID-19^a^	3D Conv. + Red. Volume	70.00	69.00	71.00	1,265
Other diseases vs. COVID-19^b^	3D Conv. + Red. Volume	77.00	86.00	69.00	122

Performance comparison of the classifier when different elements of the classification pipeline are replaced.

^a^ Results on hospitals data set with accuracy-per-stack metric.

^b^ Results on hospitals data set with stacks-average metric.

As shown in Table 3, using DeepLabv3 greatly decreases classification performance. For instance, the model’s accuracy is 10% (or more) below the values reported on Table 2. Furthermore, since this model is significantly larger than U-Net, the latter is a better option to integrate to the pipeline.

The 3D convolutions were tried drawing inspiration from other works where this kernels have been applied to classify CT studies when labeled slices are available (12,19). The implemented model featured a 7×7×7 kernel with stride 2 and 16 output channels on the first layer, which are then connected to the remaining layers of ChexNet3D via a reshape operation. Nonetheless, as Table 3 shows, the model performs worse than in the 2D setting. Jin et al. (15) also report performance degradation when implementing 3D convolutions for supervised CT classification.

Next, the effect of imposing tighter bounds on the lung bottom / top extremes was studied. As can be seen in Figure 2, before feeding the CT volume to U-Net, there are slices that do not contain visible portions of lung [(22) show this with greater detail]; therefore, it is necessary to remove them. However, defining where the cut is going to take place is not obvious, since it is possible to argue that some slices towards the extremes are not clear or informative enough. This is specially true when the volume is composed of a lot of thin slices and the change among successive slices is not significant/apparent. With this in mind, after feeding the CT through U-Net and selecting the lung slices, the volumes with 180 or more slices showing lung portions, were reduced by removing the top and bottom stacks. This allowed for higher certainty in the fact that, during inference, the models would be seeing slices with larger lung areas.

As can be seen from Table 3, the reduced volume setting did not move the classification results significantly, even though it saw around 200 less stacks (≈6,000 fewer slices) per model. In fact, using the stacks-average metric, we see the exact same performance in the Other Diseases vs. COVID-19 model, using the same 122 CT studies retrieved from the partner institutions.

Finally, the combined effect of a reduced volume on the same models with 3D convolutions was studied. But, as Table 3 shows, model performance is still suboptimal when compared with the proposed classification pipeline. Additionally, since the 2D convolution model has 7,241,410 parameters vs. 7,554,178 parameters of the 3D convolution model, the former is still a better option as it has better performance with fewer parameters.

## 5. Discussion

Table 2 shows that among the trained models, the Healthy vs. Unhealthy model’s performance was weaker. Mainly due to a sensitivity of 0.79. Increasing the number of CT studies of Healthy patients might improve this, since, as shown in Figure 3, is the category with the least amount of available data. On the other hand, the diversity of diseases aggregated in the unhealthy class (which includes 10 diseases different from COVID-19) ensures that the model can better distinguish cases of interest (COVID-19) from those that are not. Nonetheless, both models had better performance than the other weakly-supervised approaches found in the literature for this task. Both models have a remarkable precision with 85% for the Healthy vs. Unhealthy model, and 88% for the Other Diseases vs COVID-19 model. Also, both showed specificity levels 5%+ above the baselines.

The lung segmentation models play a crucial role in removing the slices that contain anatomical parts different to the lung, and helps the classifier by cropping and centering the lung around a bounding-box. Also, it is used during lesion quantification. This means that having a good model is important, since many pieces rely on it and the flow of the pipeline needs an accurate segmentation. Other architectures, such as DeepLabv3, although achieving 90% IoU on the test set, showed lower overall performance. This was particularly evidenced in patients where the lung was mostly covered by ground-glass opacities, or when both lungs showed opacities in the top/bottom and were clear in the bottom/top.

From the ablation studies, it is possible to confirm that the 2D convolution approach is better. Additionally, the results when evaluating the model with a reduced volume are good initial empirical results, that might help medical professionals assess the importance of the patterns towards the top and bottom of a lung when diagnosing COVID-19 patients. This, since it was possible to see that including them might not play a big role, although not using them weakens the model’s performance.

The lesion segmentation network performed well in slices around the center of the CT volume. The methodology proposed in Section 3.3 is critical to guarantee that the model performs well on lung slices with different shapes and sizes to those towards the center of the patient’s study. The quantification algorithm relies on the output of two models, which leads to degraded performance, but overall it returned appropriate approximations of the lesion area at each slice, as shown in Figure 6.

## 6. Conclusion

This work introduces a Medical Diagnosis Pipeline using axial lung CT volumes, where all studies are labeled according to a gold-standard test or a doctor’s diagnostic. It focuses on determining whether or not a patient’s lungs show patterns that suggest COVID-19. It involves a novel architecture that accurately classifies the regions of a patient’s lung that contain characteristics of a particular class. All of this is done in a weakly-supervised fashion and taking the whole lung volume into account, even though the number of slices might vary from one patient to the other. Additionally, a lesion segmentation model is tested and evaluated together with a lung segmentation network. This models enable further refinement of the classifier’s input and make possible an approximation of the area of the lesion in each slice.

## Data Availability

The datasets presented in this article are not readily available due to privacy agreements with the medical institutions that provided the data. Currently it is not possible to make the data collected in the Colombian medical institutions available. However, the public datasets mentioned through the manuscript are available in the referenced sources. Requests to access the datasets should be directed to amurillog@eafit.edu.co.

## Footnotes

^1^A CT study is a set of ordered slices (CT scans) that generates an accurate 3D reconstruction of the organs (3).
