# Supplementary material for: Medical decision support system using weakly-labeled lung CT scans
Source: Front Med Technol. 2022 Sep 28;4:980735. doi: 10.3389/fmedt.2022.980735 (PMC9554434; doi:10.3389/fmedt.2022.980735)

Supplementary Material

This document includes the supplementary materials of the work “Medical Decision Support System using Weakly-Labeled Lung CT Scans”. Its contents are distributed as follows:

1. Example Output from the Medical Decision Support System.
2. Results on Validation Datasets.
3. (More) Performance Metrics on Test Datasets.
4. Developed website to be used by medical professionals during deployment as a diagnostic support system.

1 Example Output from the Medical Decision Support System

The main components of the pipeline are:

- Lung Segmentation and removal of non-lung slices.
- Classification of stacks of successive slices in the CT volume.
- Segmentation and quantification of lesion on patients classified as COVID-19.

In this case, we take a patient with COVID-19 and input the CT volume through the developed pipeline. The attached documents **Annex1.pdf**, **Annex2.pdf** & **Annex3.pdf** contain the results of each phase.

Annex1.pdf shows the study once it has been segmented, non-lung slices have been removed and the remaining volume has been divided into stacks of 30 slices, which are then used as input to the classification model: *Healthy vs. Unhealthy*. Each page in the document contains the successive slices that were used to get the probability or confidence level (shown in the page's header) that such lung region shows patterns that suggest that the patient is *unhealthy*. When the probability is less than or equal to 0.5, the lung region is considered healthy; otherwise, it is considered unhealthy.

Annex2.pdf shows the study once it has been determined that the patient is unhealthy, and we want to determine whether it shows patterns that suggest COVID-19 or a different disease. In this case, the axial CT volume has also been segmented and divided into stacks of successive slices of lung, which are then used as input to the classification model: *Unhealthy vs. COVID-19*. Each page in the document contains the successive slices that were used as input and the confidence level (shown in the page's header) that the patient has patterns that suggest *COVID-19*. When the confidence level is less than or equal to 0.5, the lung region is considered unhealthy; otherwise, such region is classified as COVID-19.

Annex3.pdf contains the segmented and quantified lesion of the patient's lungs once it has been determined that she/he is unhealthy and has COVID-19. In the document, each page shows in the left-hand side the original axial CT slice and, in the right-hand side, the same image once both the lung and lesion have been segmented. Furthermore, the header of each page shows an approximate quantification of the proportion of lung that has been affected.

Annex4.pdf, **Annex5.pdf**, **Annex6.pdf** contain the segmented and quantified lesion for other randomly selected COVID-19 patients. In **Annex4.pdf**, it is possible to see an example of a patient where the lesions suggestive of COVID-19 are not particularly easy to spot; and in some portions of the volume, the lung does not show lesions, making it clear why it is necessary to consider the whole scan to return a diagnosis.

2 Results on Validation Datasets

Colombian Hospitals Dataset

Model: Healthy vs. Unhealthy

Metric: Accuracy Per Stack

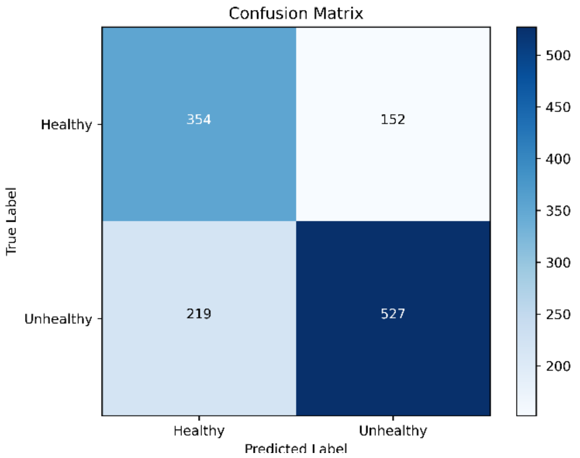

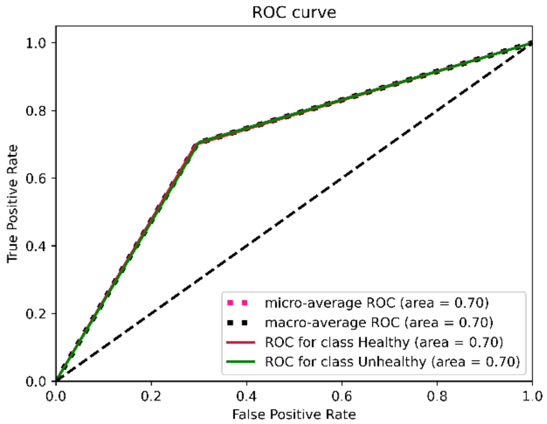

Metric: Stacks Average in each Study

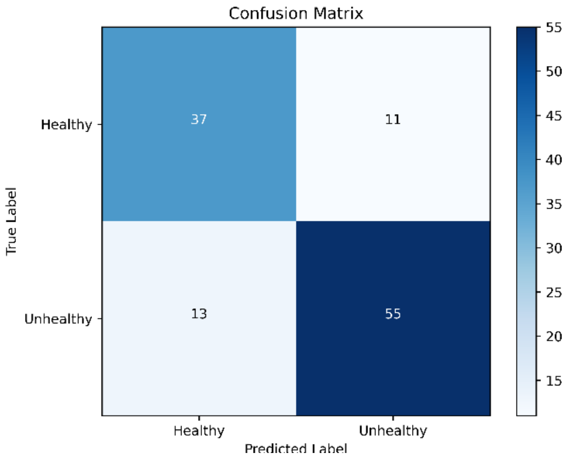

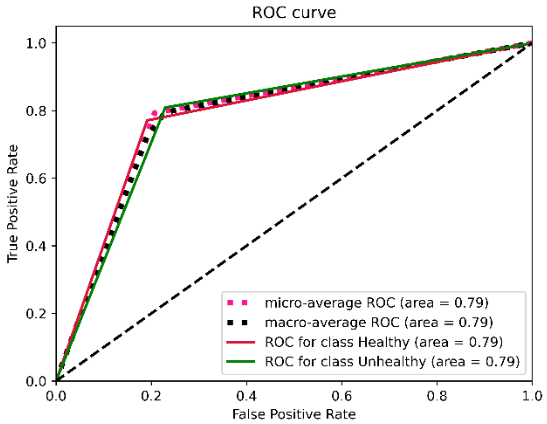

Model: Unhealthy vs. COVID-19

Metric: Accuracy Per Stack

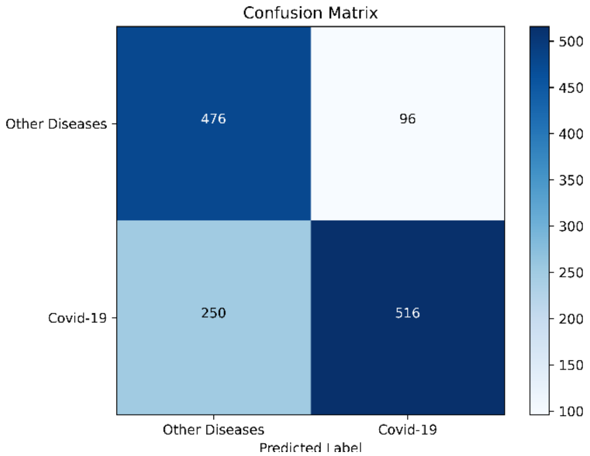

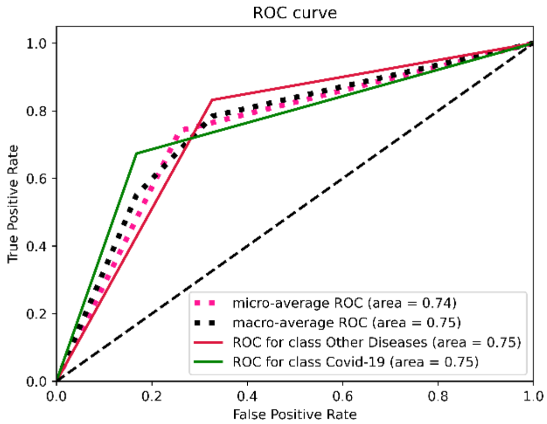

Metric: Stacks Average in each Study

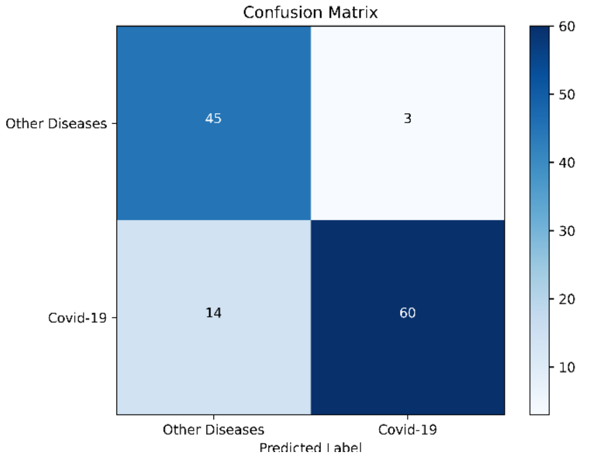

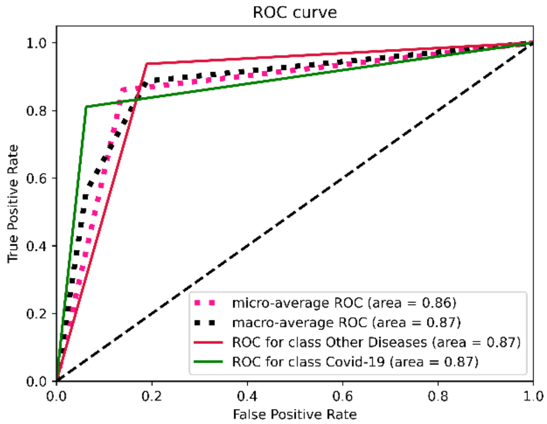

3 Performance Metrics on Test Datasets

Public Dataset

The following tables contain relevant performance metrics of the introduced models (pre-finetunning setting) using different diagnostic metrics on the *test set* of an aggregated dataset of axial lung CT scans collected from multiple public sources.

Model: Healthy vs. Unhealthy

Diagnostic Metric: *At Least One*

Classification Report				
	Precision	Recall	F1-Score	Support
Healthy	0.91	0.74	0.82	180
Unhealthy	0.79	0.92	0.85	184
Accuracy			0.84	364
Macro avg	0.85	0.83	0.83	364
Weighted avg	0.85	0.84	0.83	364

Relevant Medical Statistics	
Sensitivity	0.92
Specificity	0.74
Precision	0.79
Negative Pred. Value	0.91
False Positive Rate	0.26
False Discovery Rate	0.21
False Negative Rate	0.08
Accuracy	0.84
F1-score	0.85
Matthews Corr. Coeff.	0.68

Model: Unhealthy vs. COVID-19

Diagnostic Metric: *At Least One*

Classification Report				
	Precision	Recall	F1-Score	Support
Healthy	0.98	0.81	0.89	374
Unhealthy	0.84	0.99	0.91	385
Accuracy			0.90	759
Macro avg	0.91	0.90	0.90	759
Weighted avg	0.91	0.90	0.90	759

Relevant Medical Statistics	
Sensitivity	0.99
Specificity	0.81
Precision	0.84
Negative Pred. Value	0.98
False Positive Rate	0.19
False Discovery Rate	0.16
False Negative Rate	0.01
Accuracy	0.90
F1-score	0.91
Matthews Corr. Coeff.	0.81

Colombian Hospitals Dataset

The following tables contain relevant performance metrics of the introduced models using different diagnostic metrics on the *test set* of the retrospectively collected dataset of axial CT scans from patients in multiple Colombian health institutions.

Model: Healthy vs. Unhealthy

Diagnostic Metric: *Accuracy Per Stack*

Classification Report				
	**Precision**	**Recall**	**F1-Score**	**Support**
**Healthy**	0.68	0.76	0.72	656
**Unhealthy**	0.75	0.67	0.71	716
**Accuracy**			0.71	1372
**Macro avg**	0.71	0.71	0.71	1372
**Weighted avg**	0.72	0.71	0.71	1372

Relevant Medical Statistics	
**Sensitivity**	0.67
**Specificity**	0.76
**Precision**	0.75
**Negative Pred. Value**	0.68
**False Positive Rate**	0.24
**False Discovery Rate**	0.25
**False Negative Rate**	0.33
**Accuracy**	0.71
**F1-score**	0.71
**Matthews Corr. Coeff.**	0.43

Model: Healthy vs. Unhealthy

Diagnostic Metric: *Stacks Average in each Study*

Classification Report				
	**Precision**	**Recall**	**F1-Score**	**Support**
**Healthy**	0.81	0.87	0.84	60
**Unhealthy**	0.85	0.79	0.82	57
**Accuracy**			0.83	117
**Macro avg**	0.83	0.83	0.83	117
**Weighted avg**	0.83	0.83	0.83	117

Relevant Medical Statistics	
**Sensitivity**	0.79
**Specificity**	0.87
**Precision**	0.85
**Negative Pred. Value**	0.81
**False Positive Rate**	0.13
**False Discovery Rate**	0.15
**False Negative Rate**	0.21
**Accuracy**	0.83
**F1-score**	0.82
**Matthews Corr. Coeff.**	0.66

Model: Unhealthy vs. COVID-19

Diagnostic Metric: *Accuracy Per Stack*

Classification Report				
	Precision	Recall	F1-Score	Support
Healthy	0.77	0.82	0.79	851
Unhealthy	0.71	0.65	0.68	594
Accuracy			0.75	1445
Macro avg	0.74	0.73	0.74	1445
Weighted avg	0.75	0.75	0.75	1445

Relevant Medical Statistics	
Sensitivity	0.65
Specificity	0.82
Precision	0.71
Negative Pred. Value	0.77
False Positive Rate	0.18
False Discovery Rate	0.29
False Negative Rate	0.35
Accuracy	0.75
F1-score	0.68
Matthews Corr. Coeff.	0.48

Model: Unhealthy vs. COVID-19

Diagnostic Metric: *Stacks Average in each Study*

Classification Report				
	Precision	Recall	F1-Score	Support
Healthy	0.84	0.91	0.87	65
Unhealthy	0.88	0.81	0.84	57
Accuracy			0.86	122
Macro avg	0.86	0.86	0.86	122
Weighted avg	0.86	0.86	0.86	122

Relevant Medical Statistics	
Sensitivity	0.81
Specificity	0.91
Precision	0.88
Negative Pred. Value	0.84
False Positive Rate	0.09
False Discovery Rate	0.12
False Negative Rate	0.19
Accuracy	0.86
F1-score	0.84
Matthews Corr. Coeff.	0.72

4 Developed Website

The following is a set of screenshots of the website that is going to be deployed as a decision support system for medical professionals.

Model Home Screen:

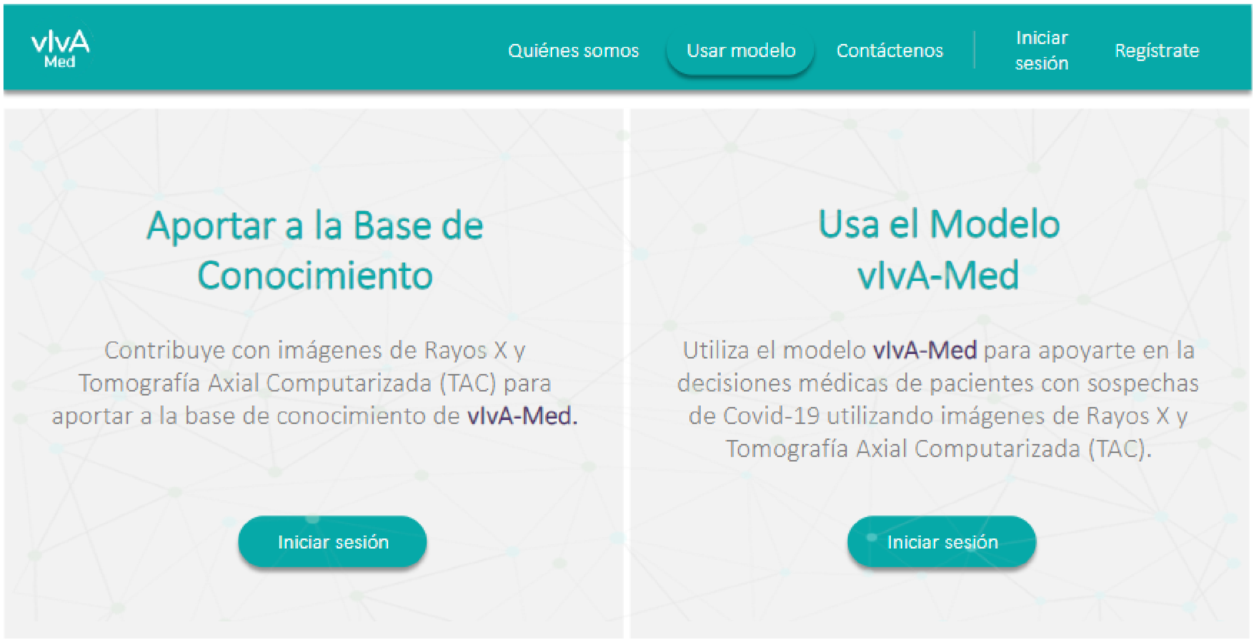

Disclaimers:

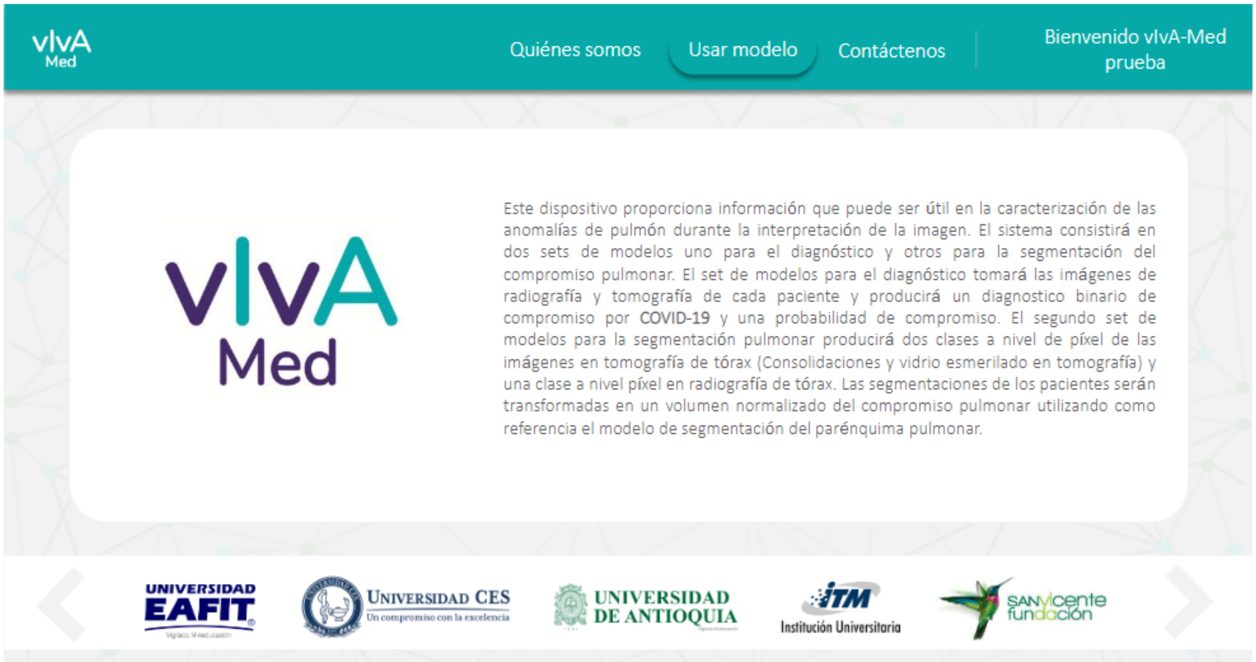

Choose between using RX or CT model:

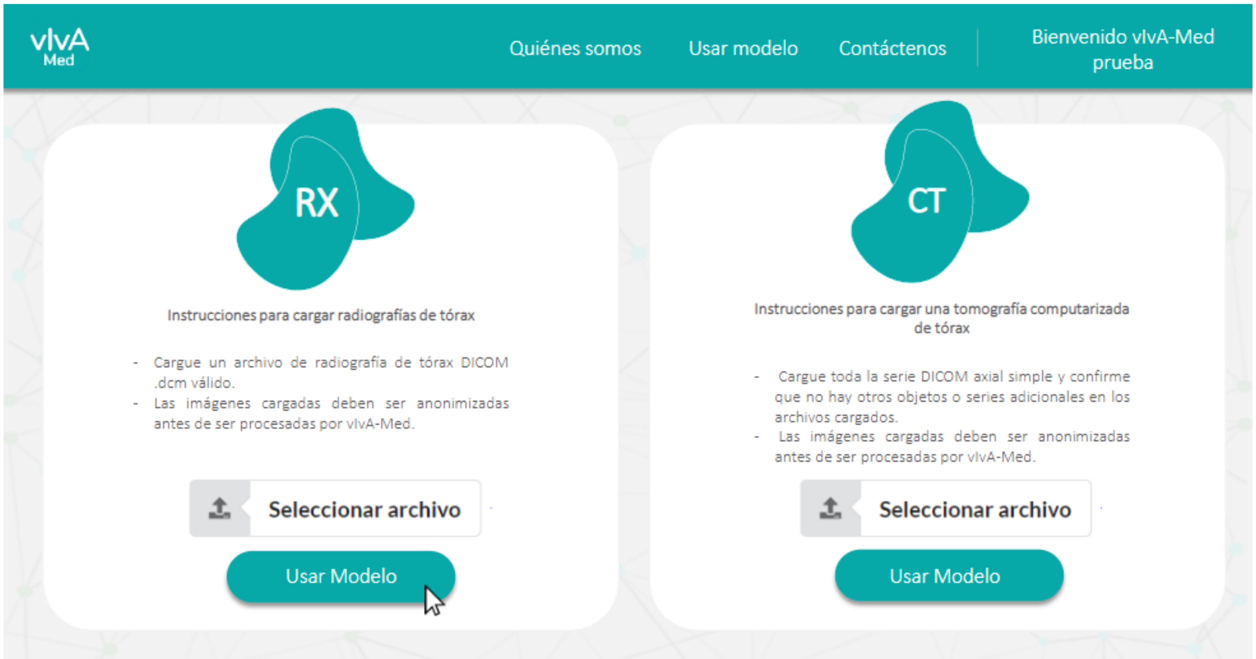

Reminder to users about the supported files:

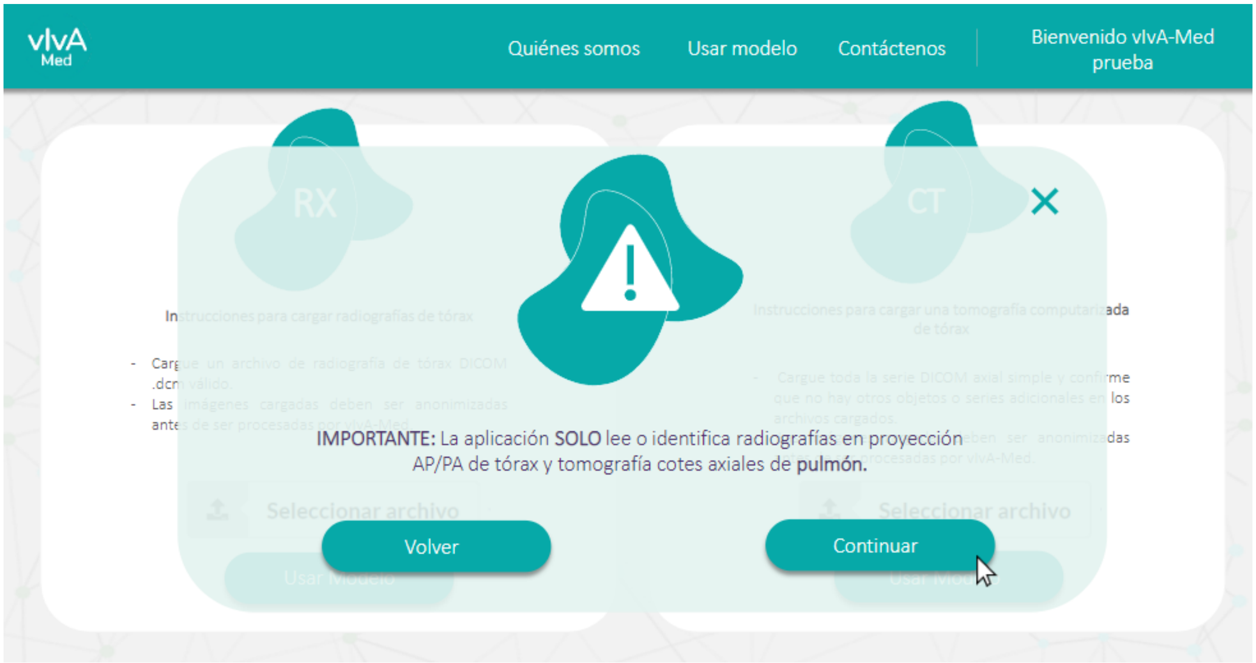

Output for each stack in the CT volume:

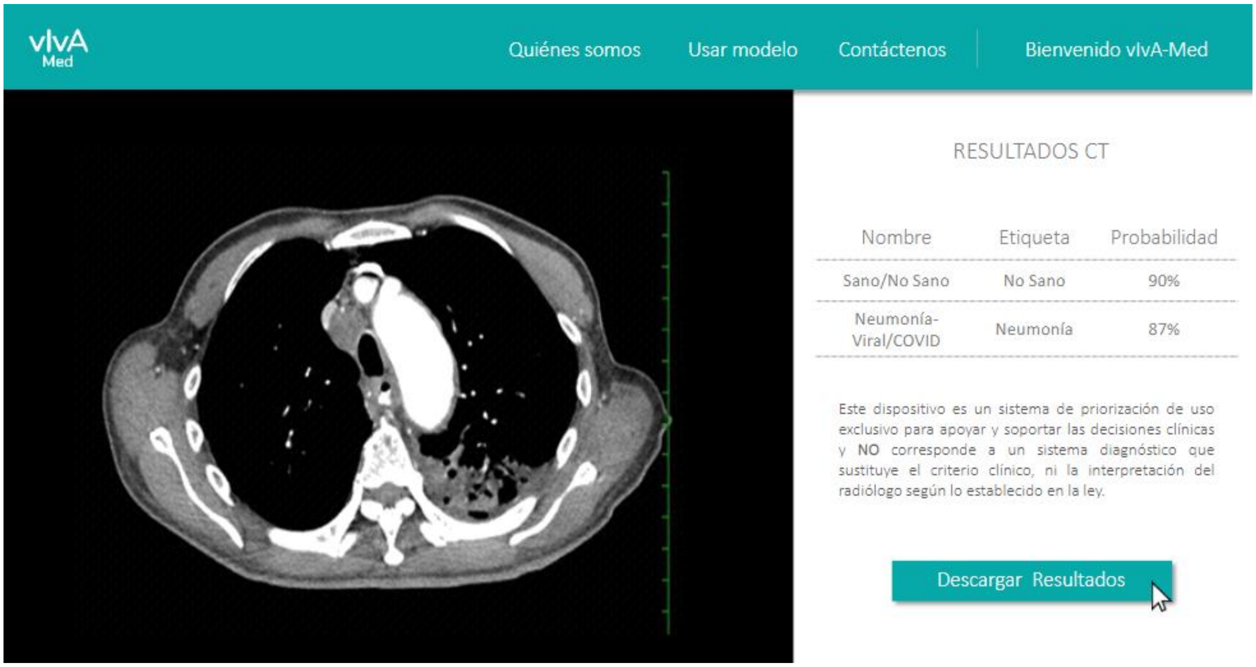

Analysis History section:

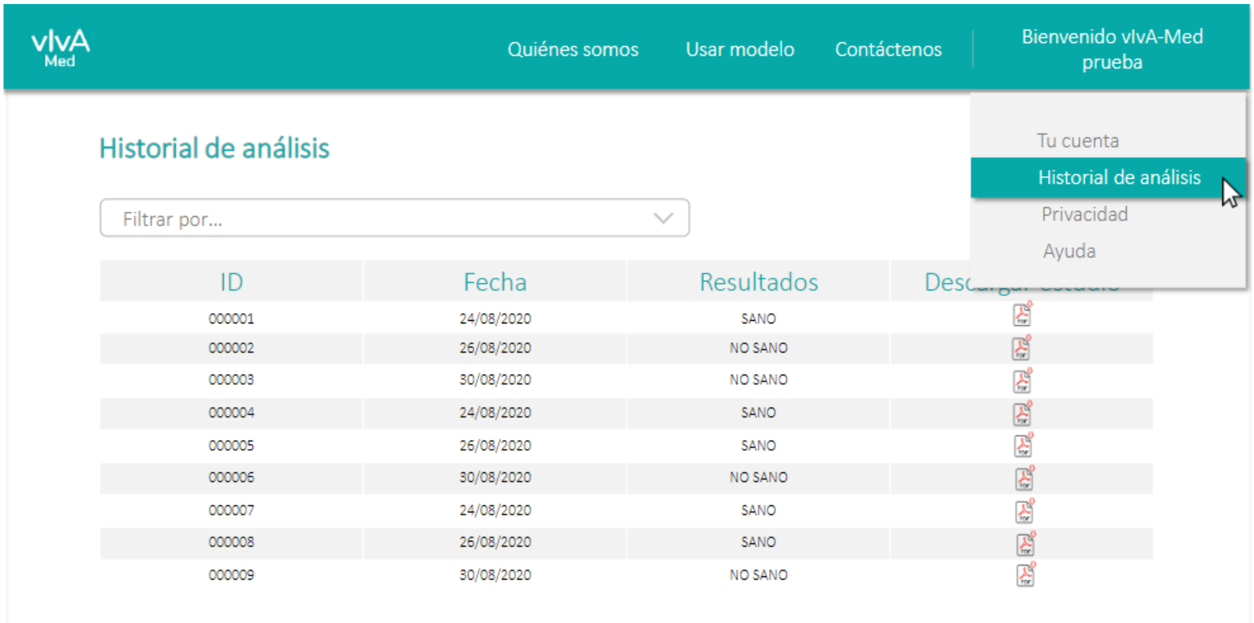

Supplement: Supplementary file 1 [file DataSheet1.pdf]
