# Supplementary figures and images for: Medical decision support system using weakly-labeled lung CT scans
Source: Front Med Technol. 2022 Sep 28;4:980735. doi: 10.3389/fmedt.2022.980735 (PMC9554434; doi:10.3389/fmedt.2022.980735)

HealthyUnhealthy | Confidence = 0.7923389077186584

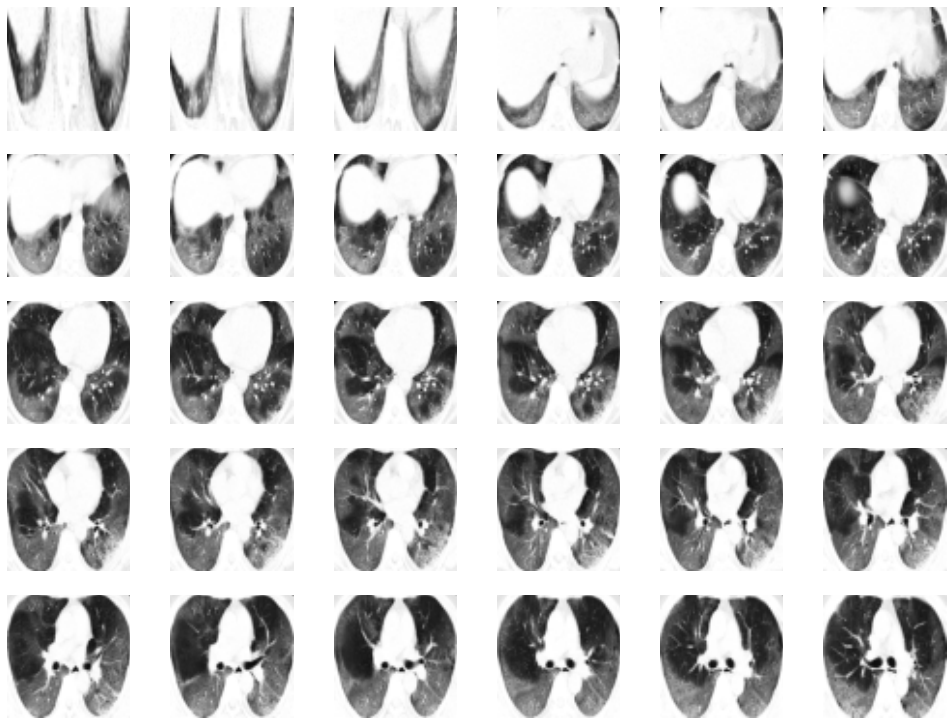

HealthyUnhealthy | Confidence = 0.9135421514511108

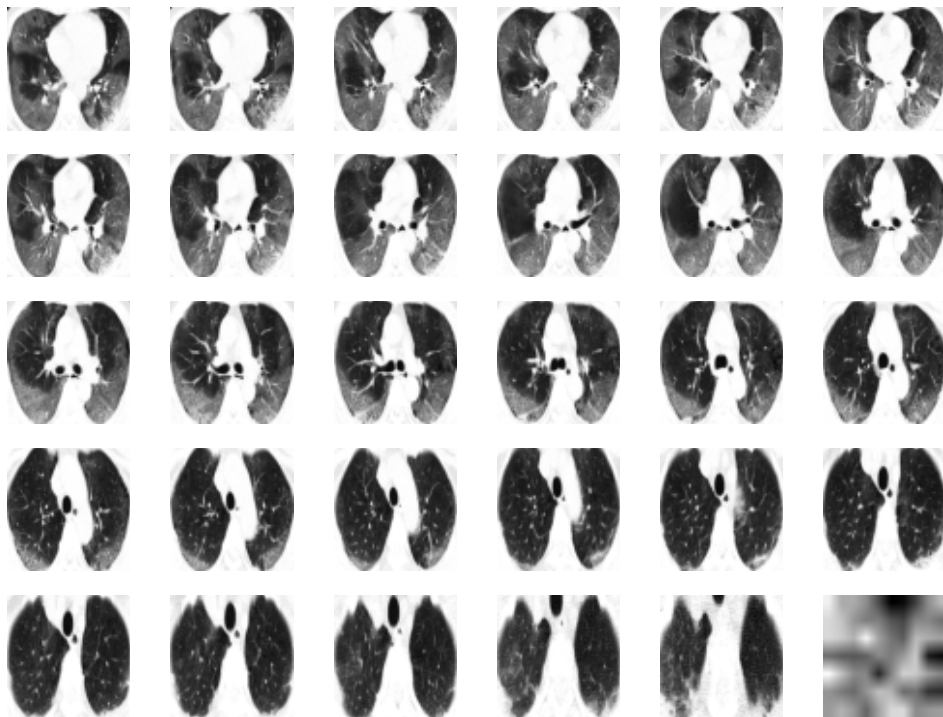

Supplement: Supplementary file 2 [file DataSheet2.pdf]

UnhealthyCovid19 | Confidence = 0.9938596487045288

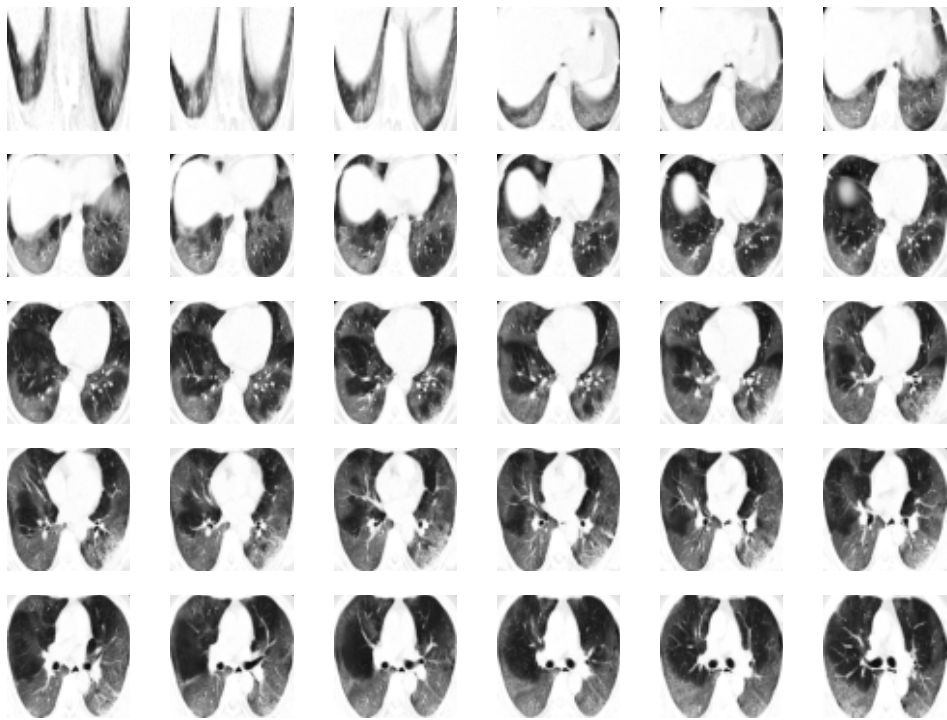

UnhealthyCovid19 | Confidence = 0.9991759657859802

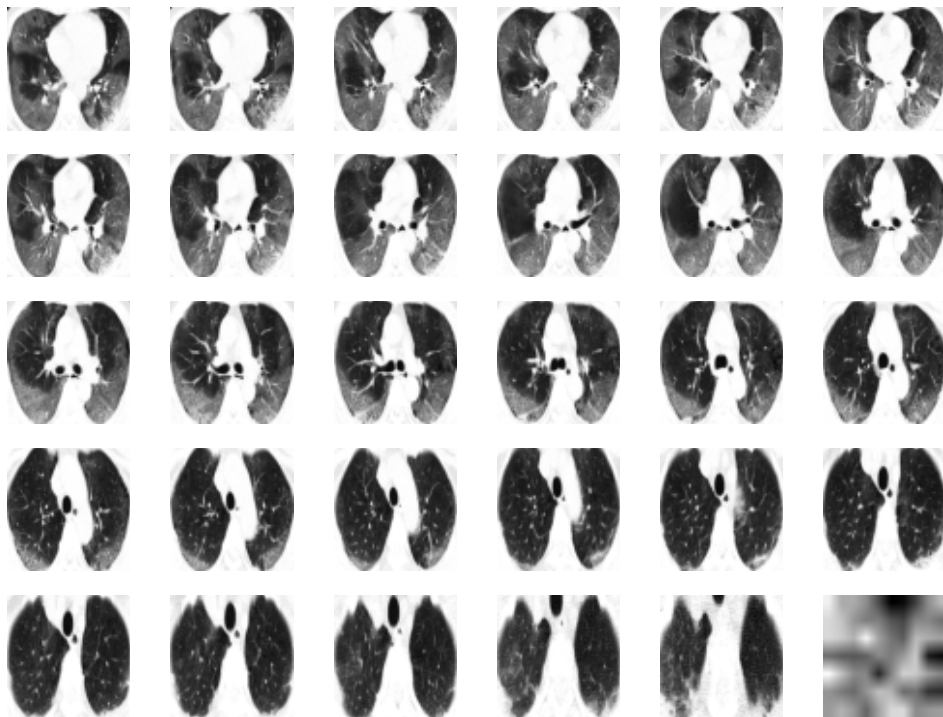

Supplement: Supplementary file 3 [file DataSheet3.pdf]
