# Supplementary material for: Medical decision support system using weakly-labeled lung CT scans
Source: Front Med Technol. 2022 Sep 28;4:980735. doi: 10.3389/fmedt.2022.980735 (PMC9554434; doi:10.3389/fmedt.2022.980735)

Lesion Proportion: 40.56%

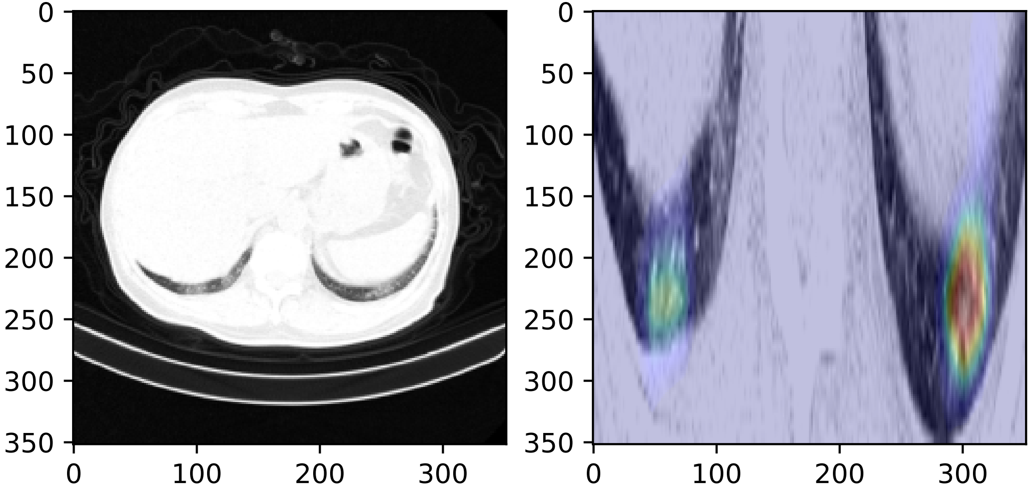

Lesion Proportion: 34.65%

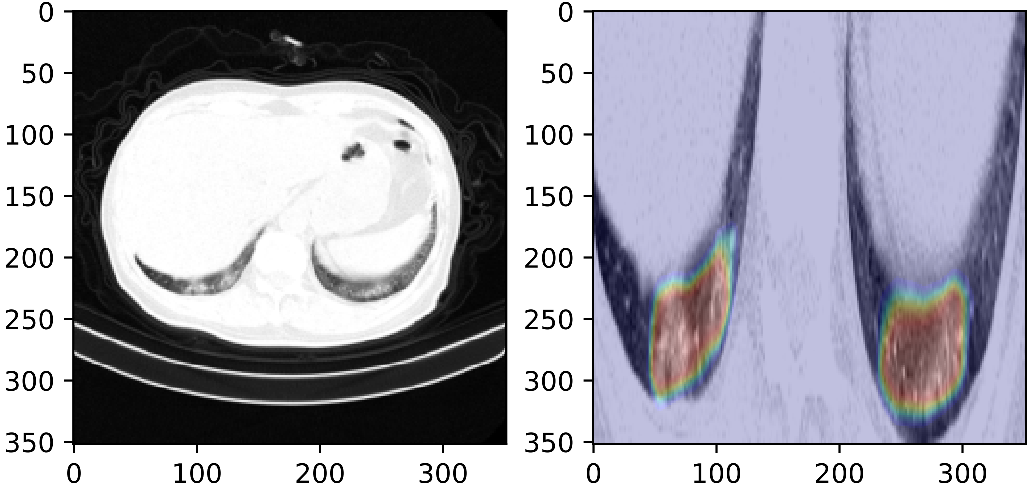

Lesion Proportion: 29.56%

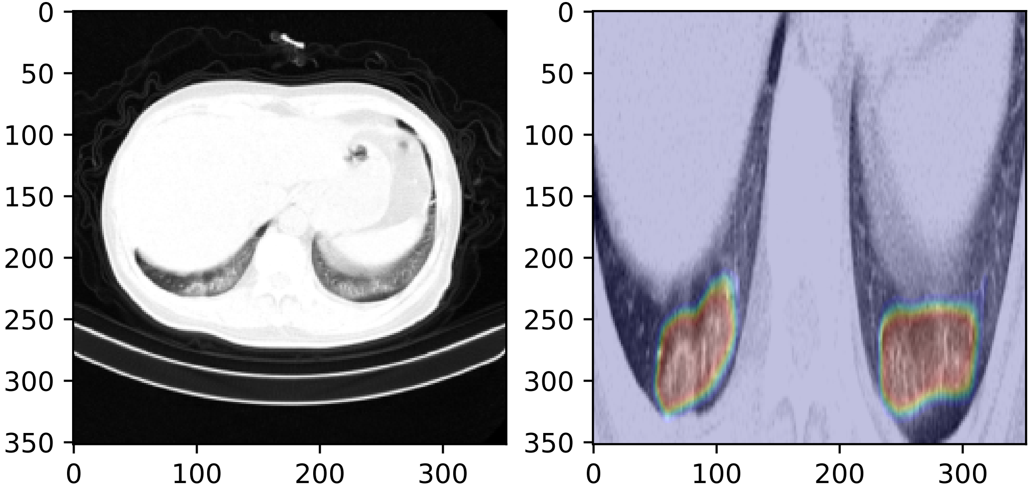

Lesion Proportion: 38.73%

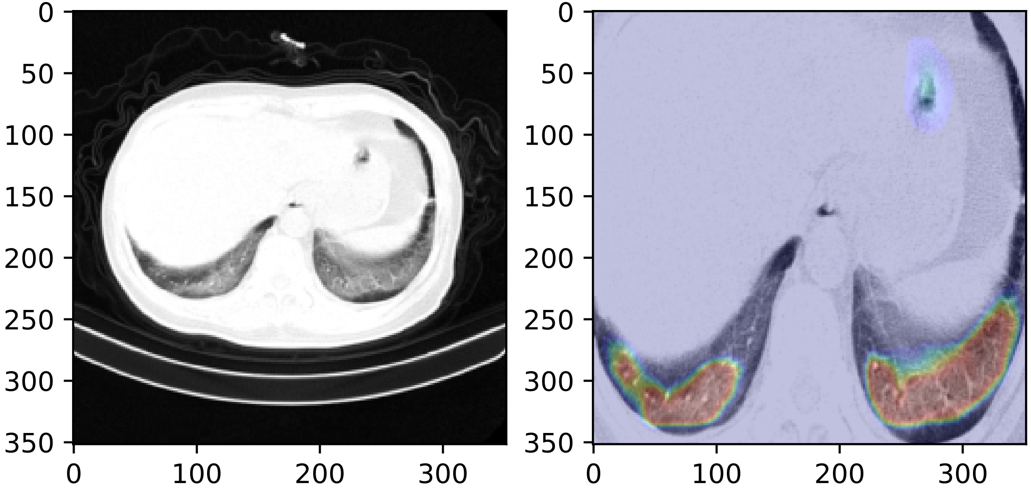

Lesion Proportion: 51.03%

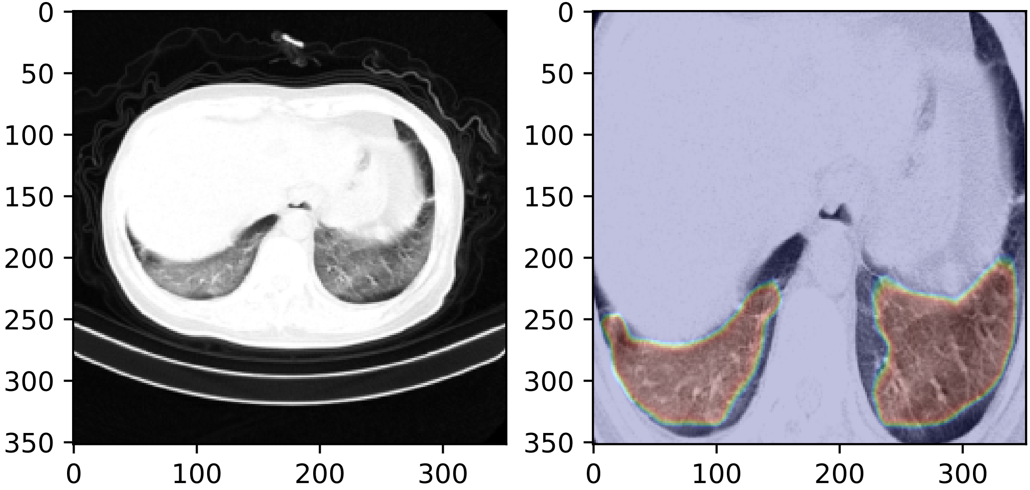

Lesion Proportion: 59.11%

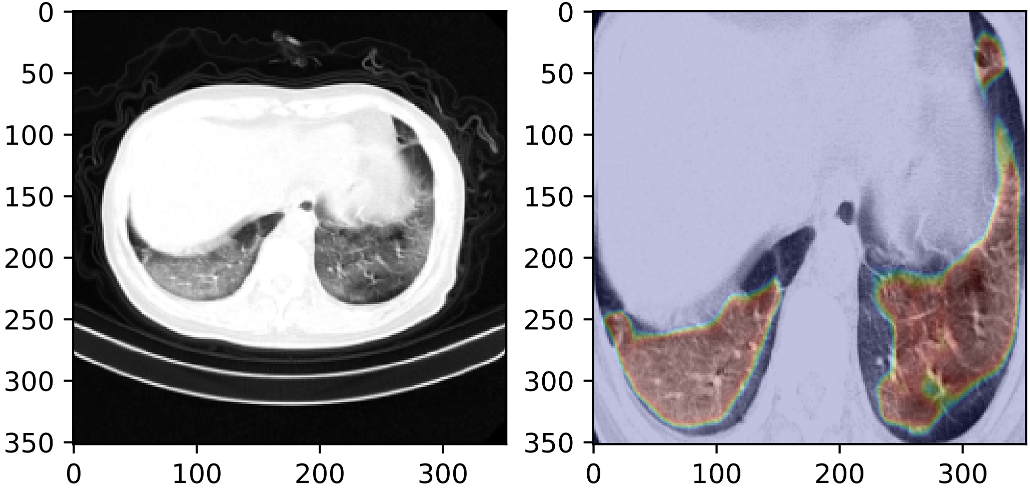

Lesion Proportion: 61.31%

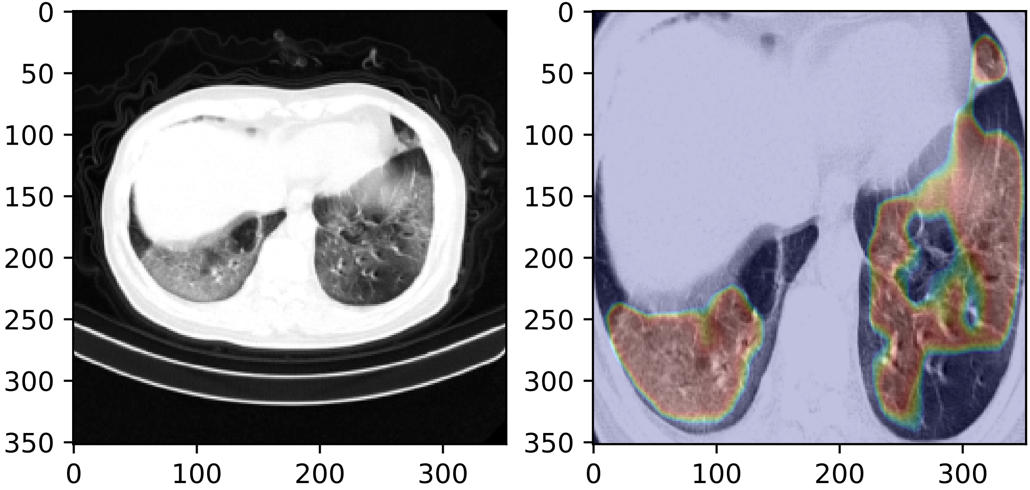

Lesion Proportion: 56.76%

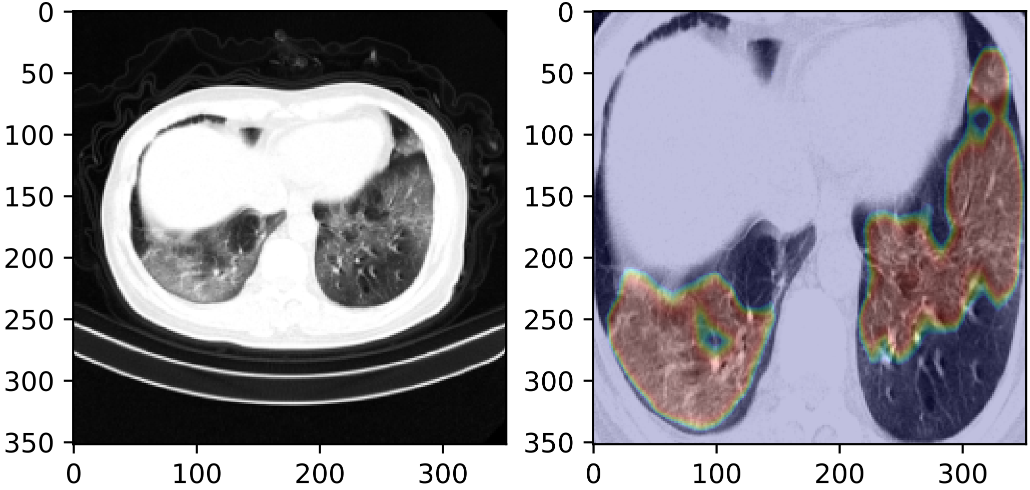

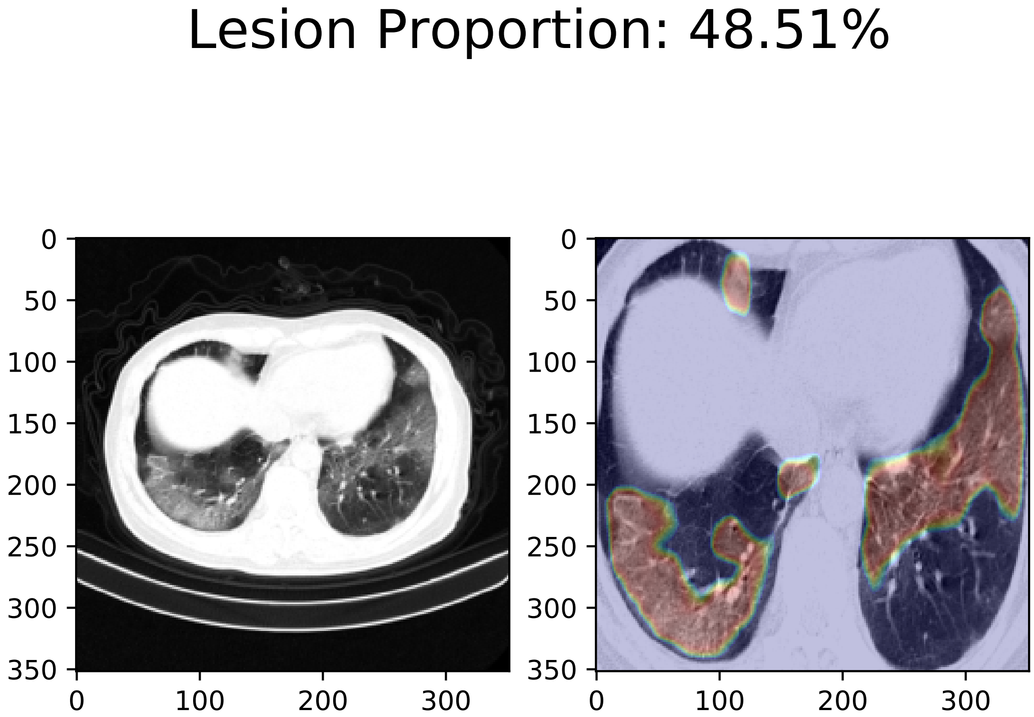
Lesion Proportion: 48.51%

Lesion Proportion: 48.28%

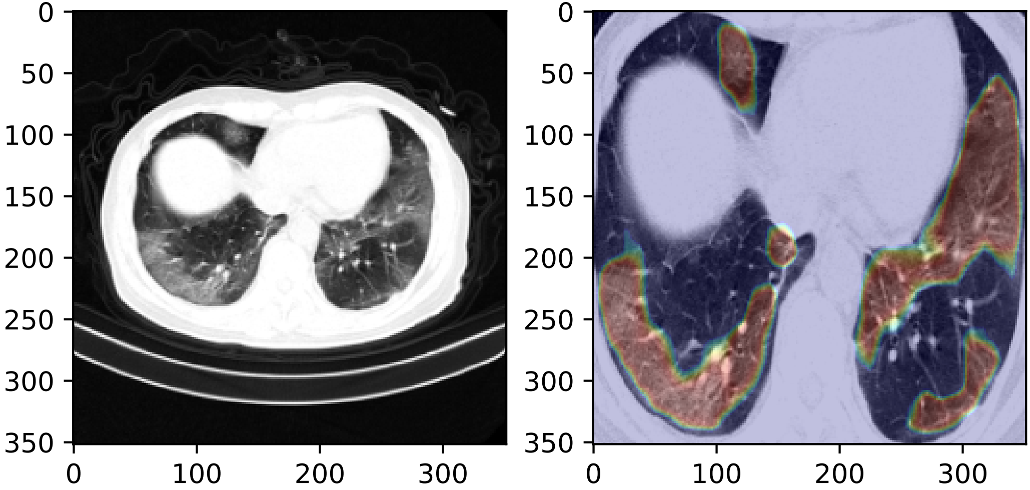

Lesion Proportion: 41.48%

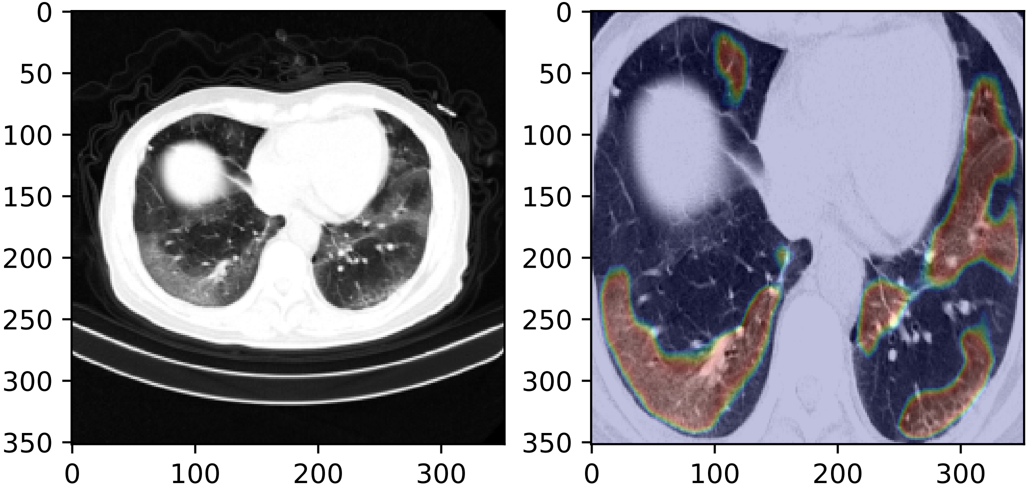

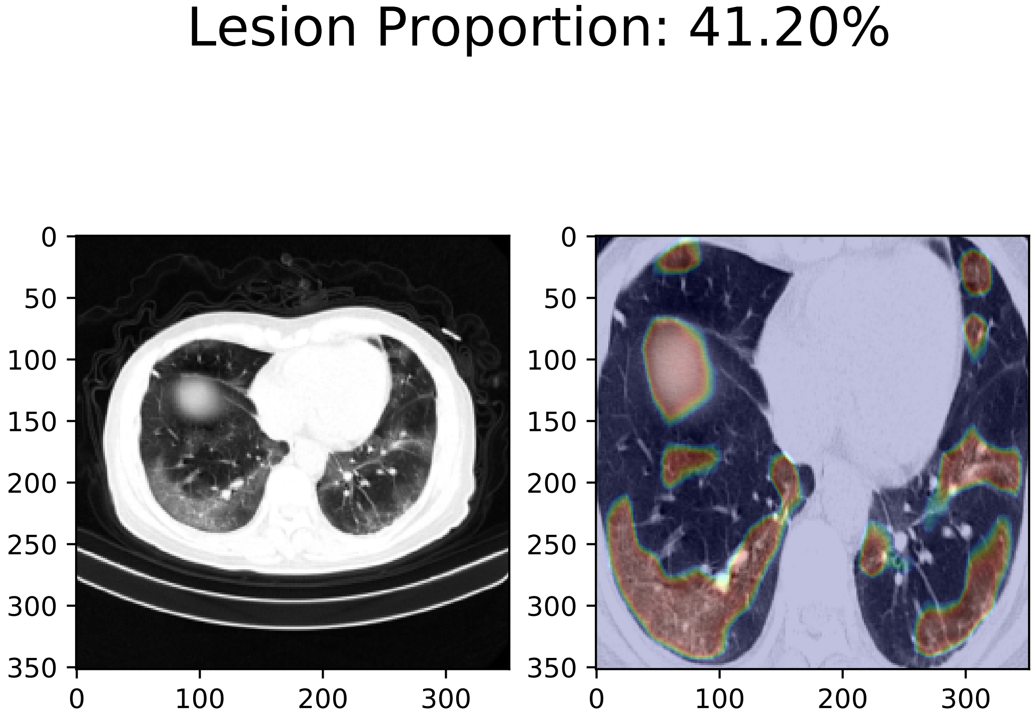
Lesion Proportion: 41.20%
0
50
100
150
200
250
300
350
0
100
200
300
0
50
100
150
200
250
300
350
0
100
200
300

Lesion Proportion: 38.06%

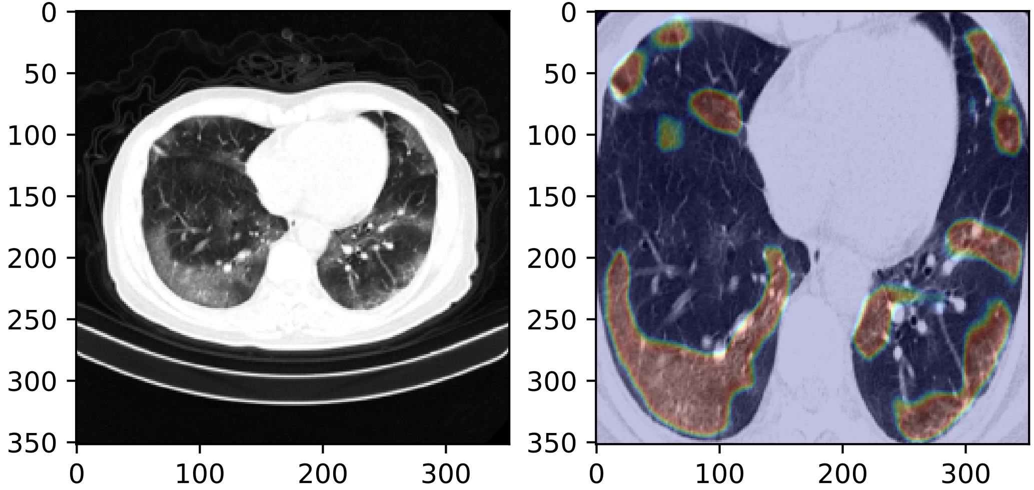

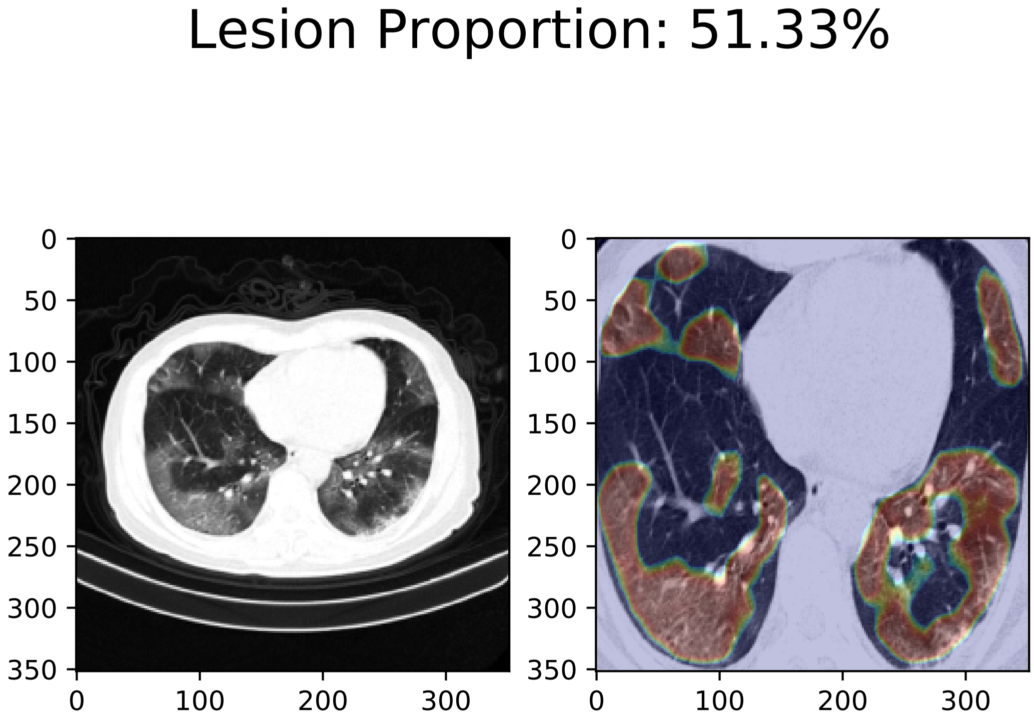
Lesion Proportion: 51.33%
0
50
100
150
200
250
300
350
0
100
200
300
0
50
100
150
200
250
300
350
0
100
200
300

Lesion Proportion: 55.00%

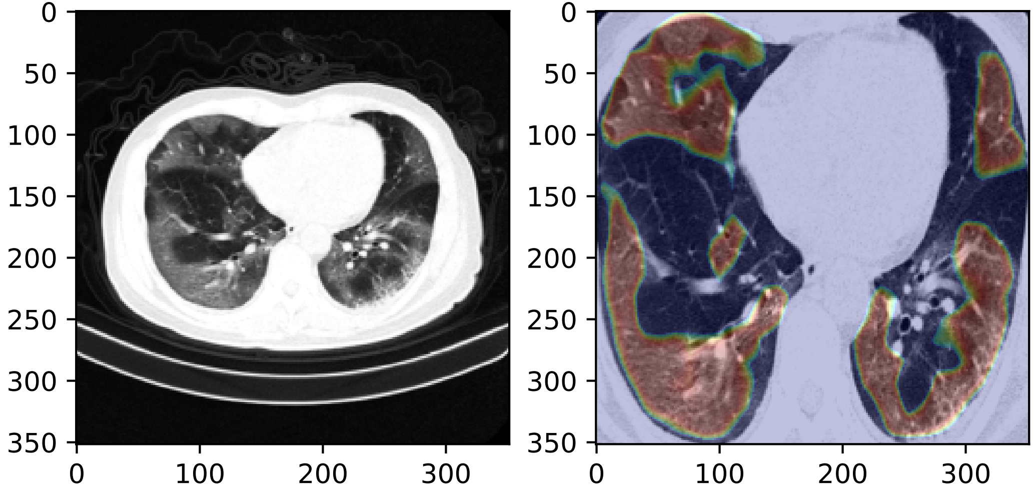

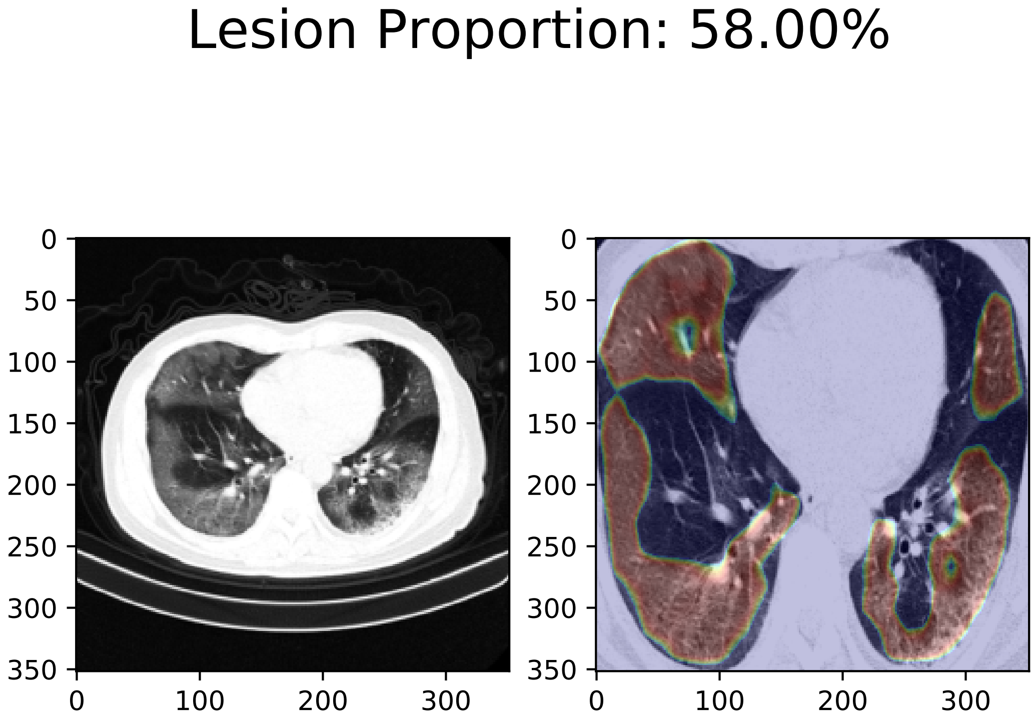

Lesion Proportion: 58.00%
0
50
100
150
200
250
300
350
0
100
200
300
0
50
100
150
200
250
300
350
0
100
200
300

Lesion Proportion: 60.41%

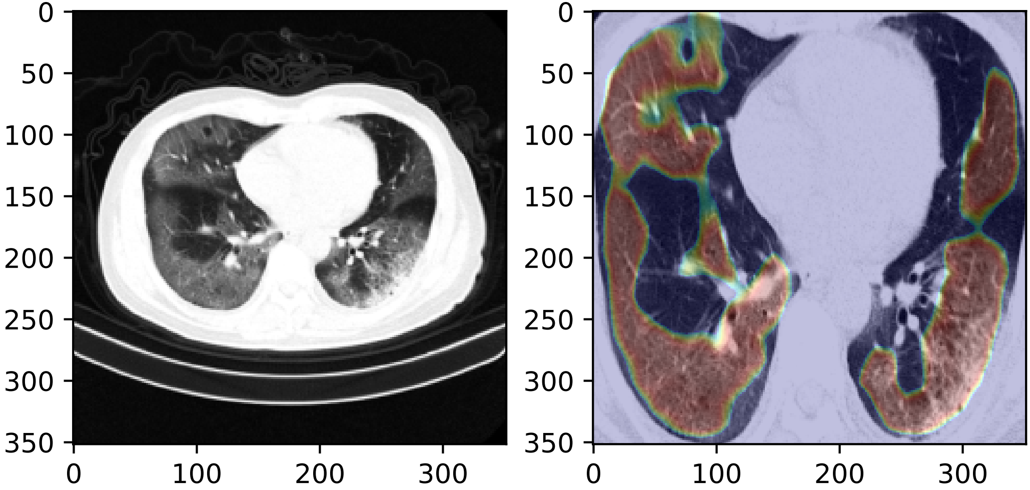

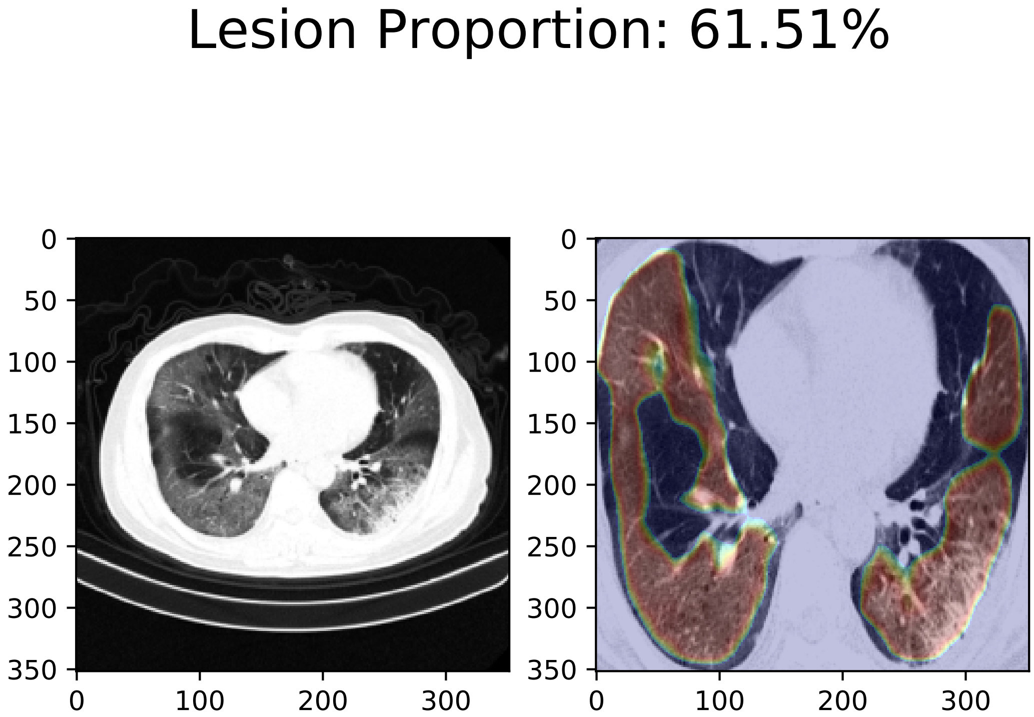
Lesion Proportion: 61.51%
0
50
100
150
200
250
300
350
0
100
200
300
0
50
100
150
200
250
300
350
0
100
200
300

Lesion Proportion: 55.82%

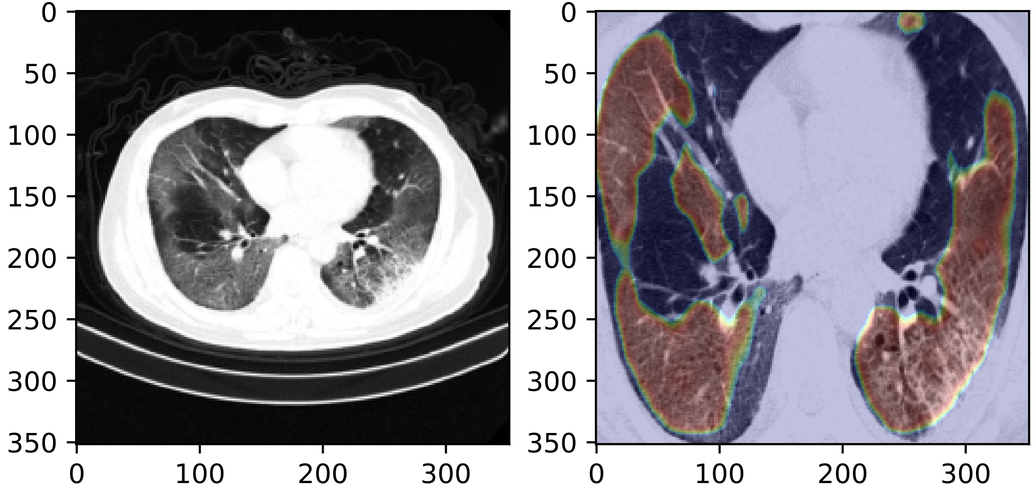

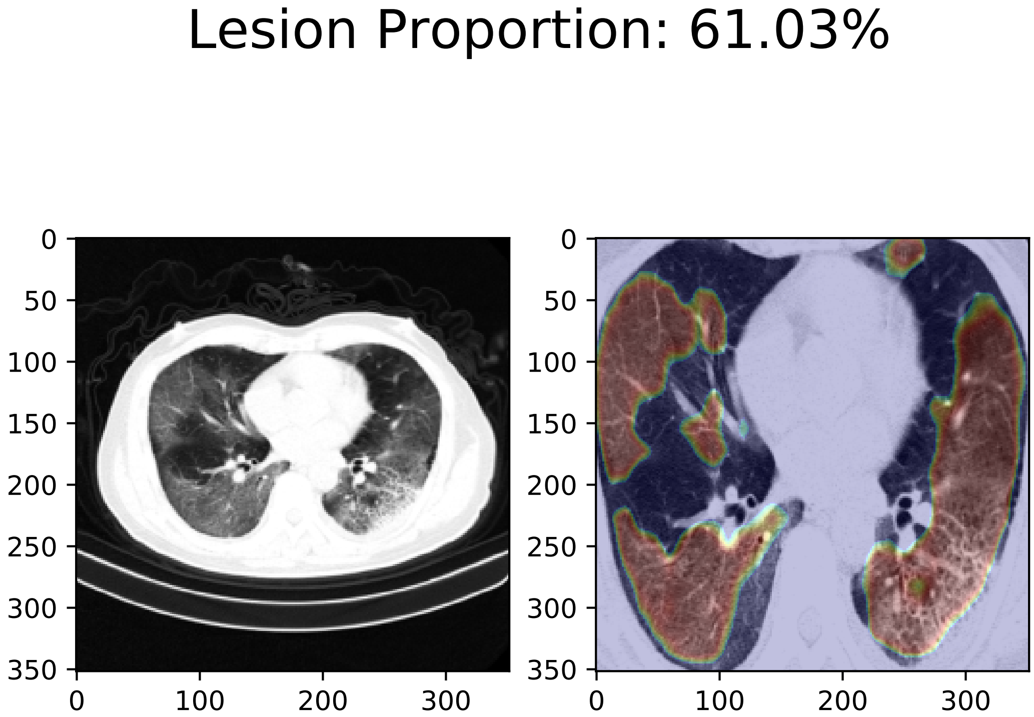

Lesion Proportion: 61.03%
0
50
100
150
200
250
300
350
0
100
200
300

Lesion Proportion: 60.68%

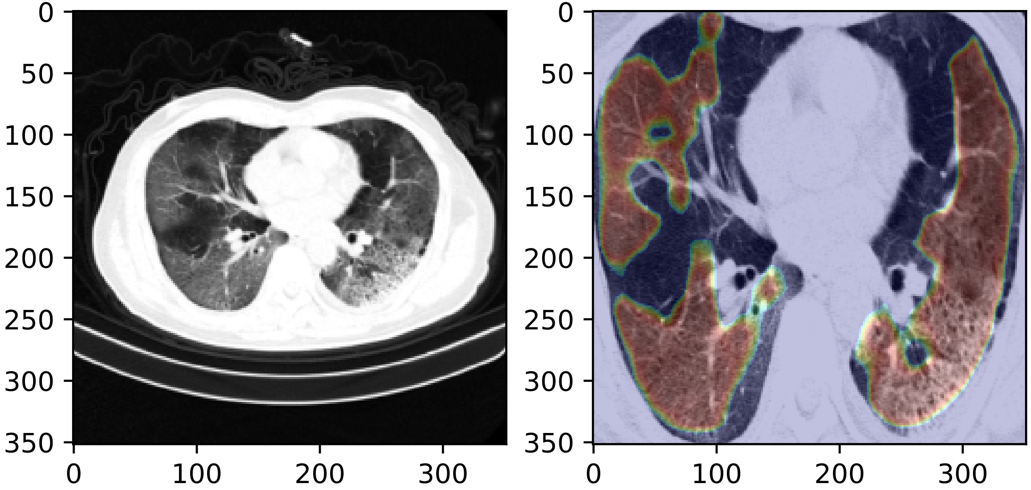

Lesion Proportion: 61.76%

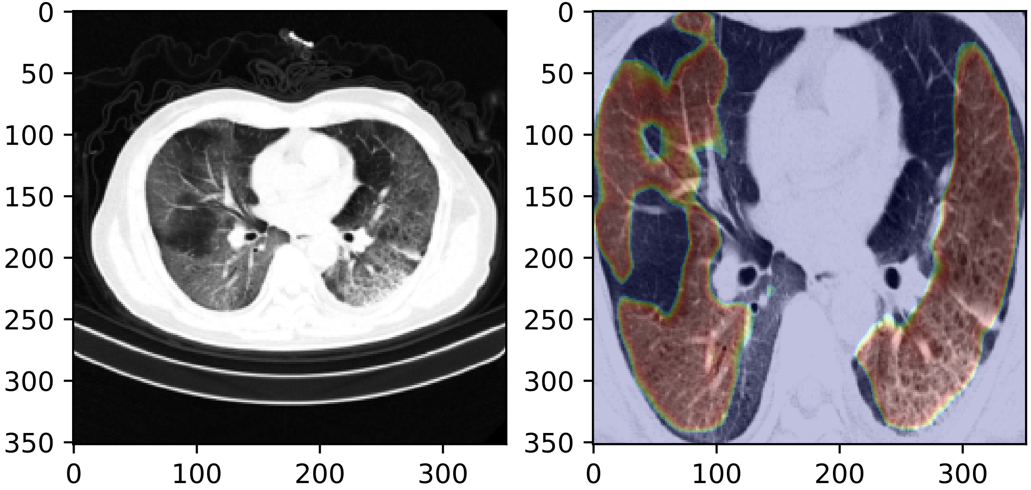

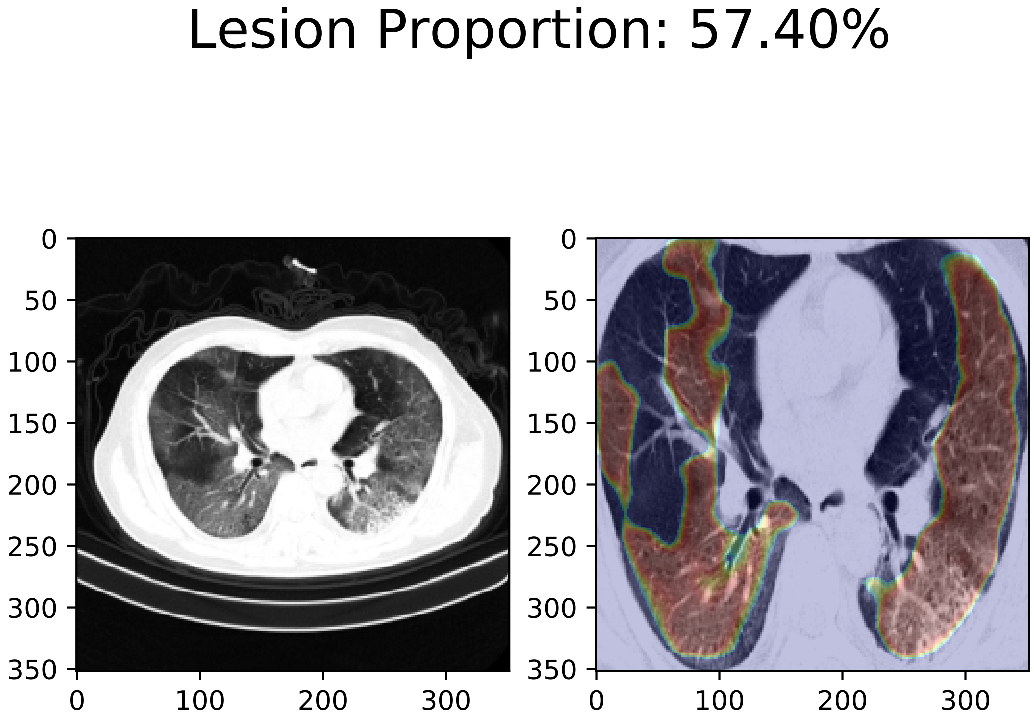

Lesion Proportion: 57.40%
0
50
100
150
200
250
300
350
0
100
200
300

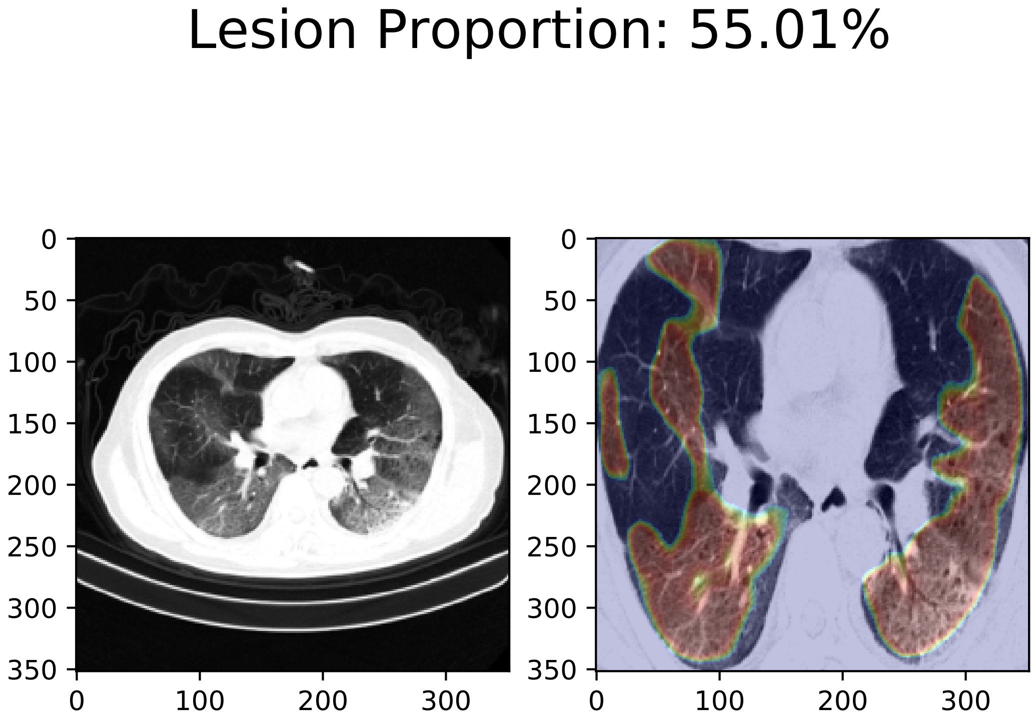
Lesion Proportion: 55.01%
0
50
100
150
200
250
300
350
0
100
200
300
0
50
100
150
200
250
300
350
0
100
200
300

Lesion Proportion: 56.49%

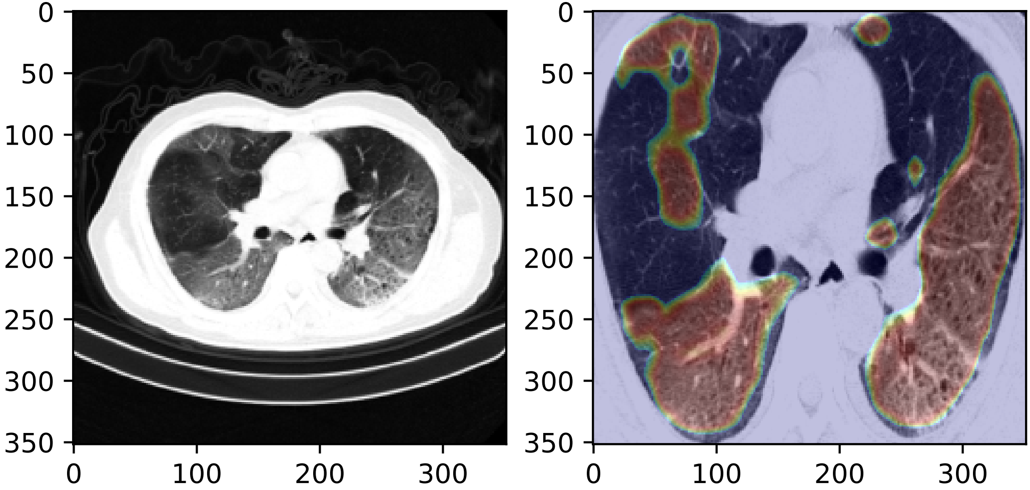

Lesion Proportion: 49.12%

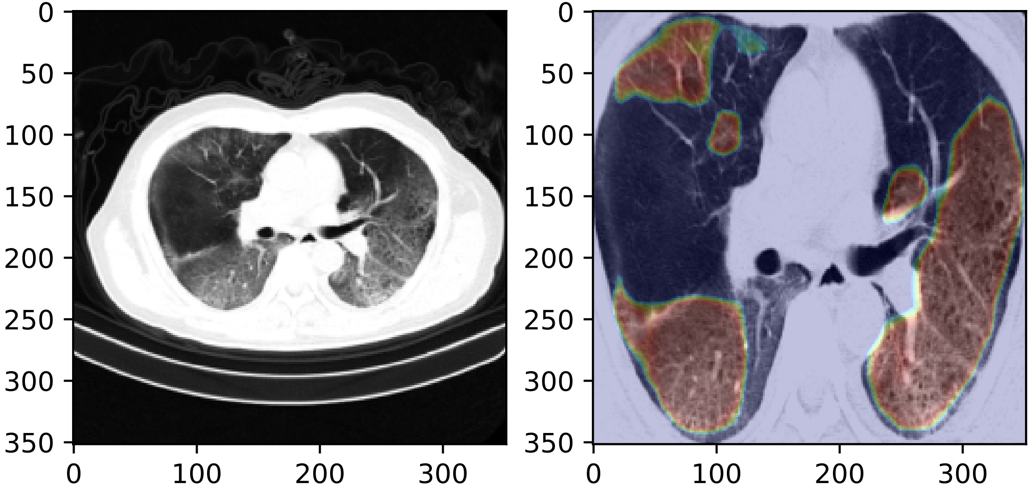

Lesion Proportion: 54.63%

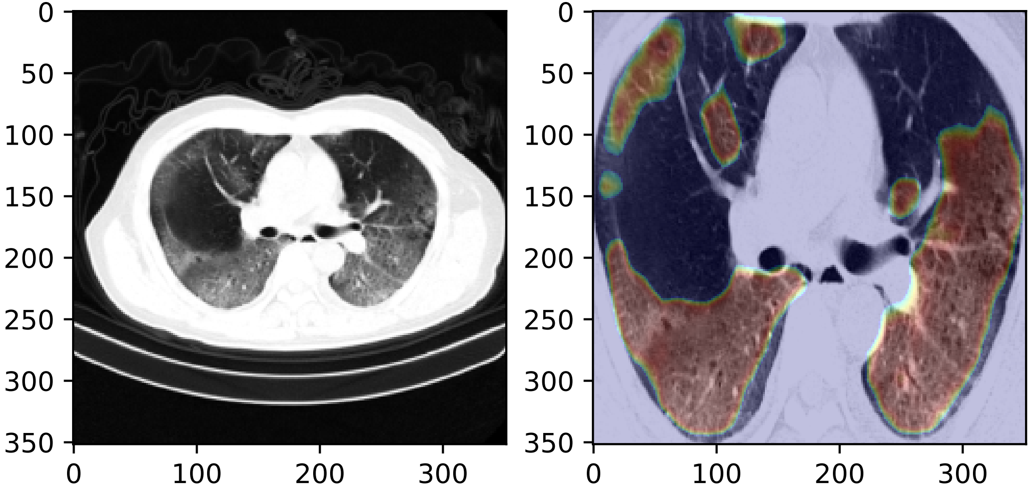

Lesion Proportion: 46.03%

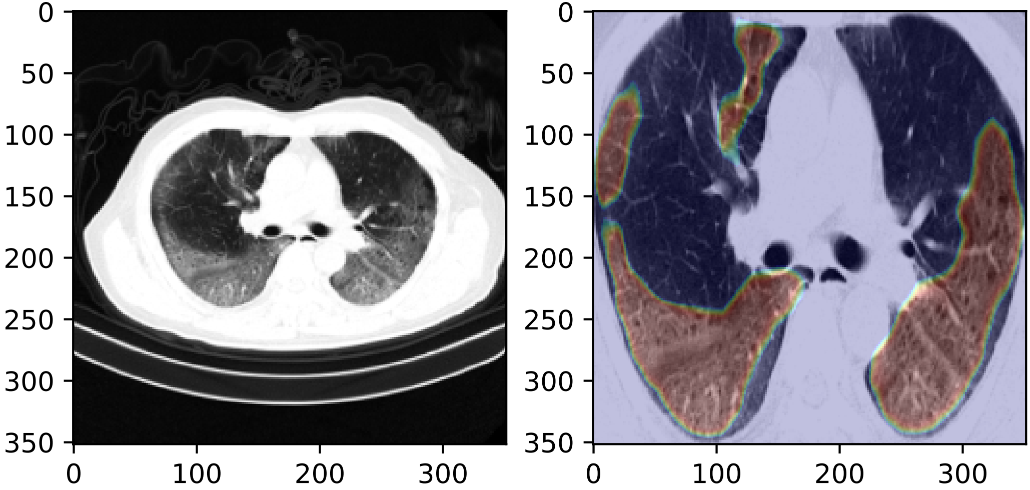

Lesion Proportion: 47.84%

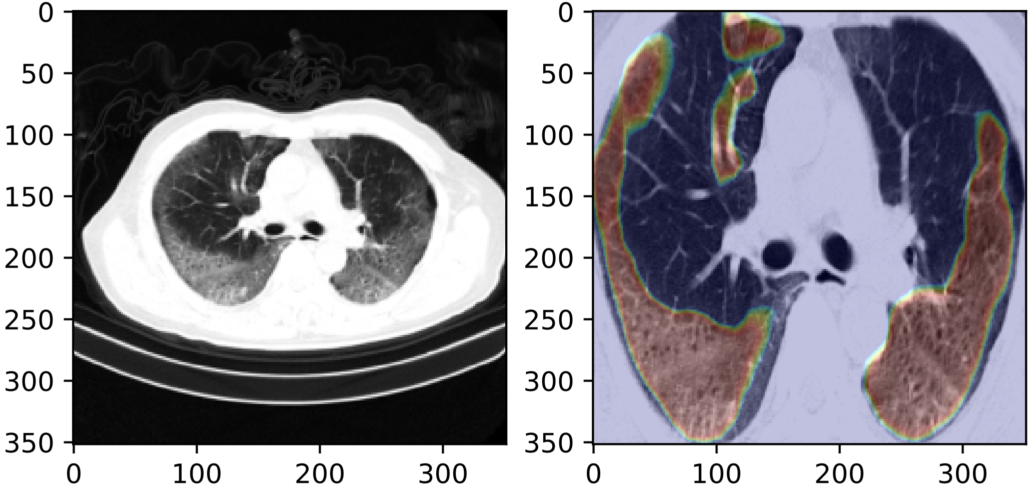

Lesion Proportion: 42.61%

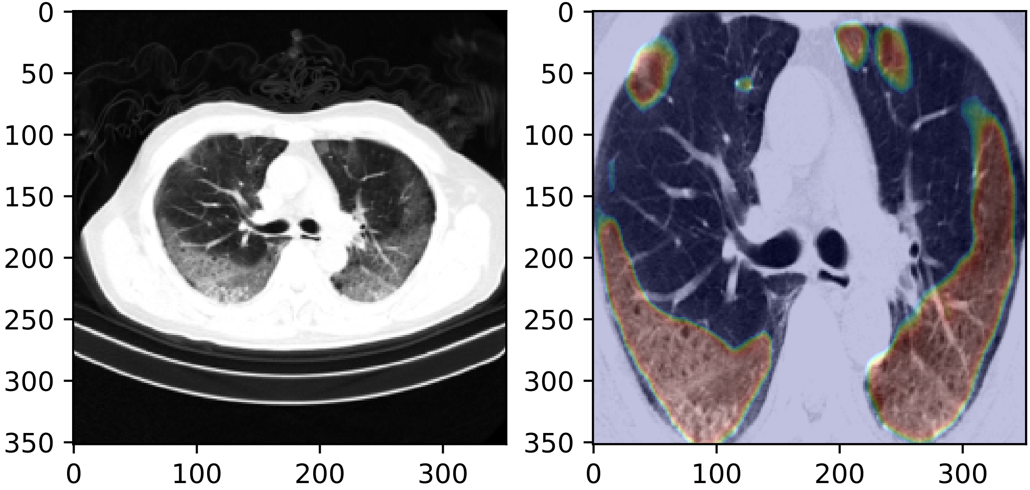

Lesion Proportion: 38.89%

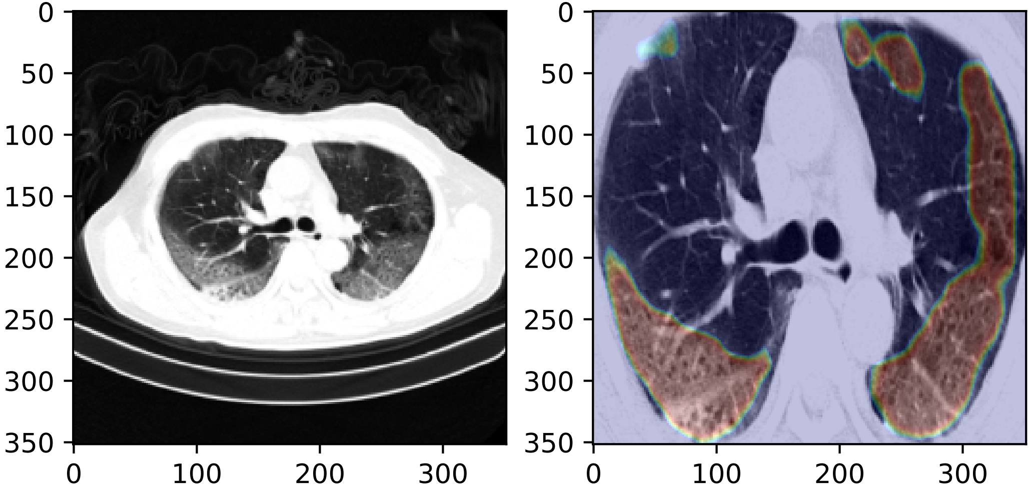

Lesion Proportion: 31.39%

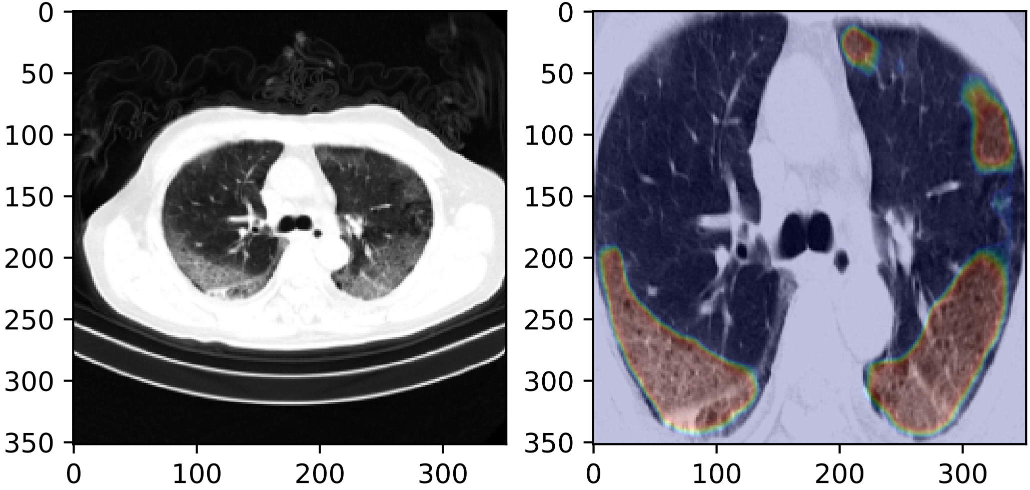

Lesion Proportion: 29.07%

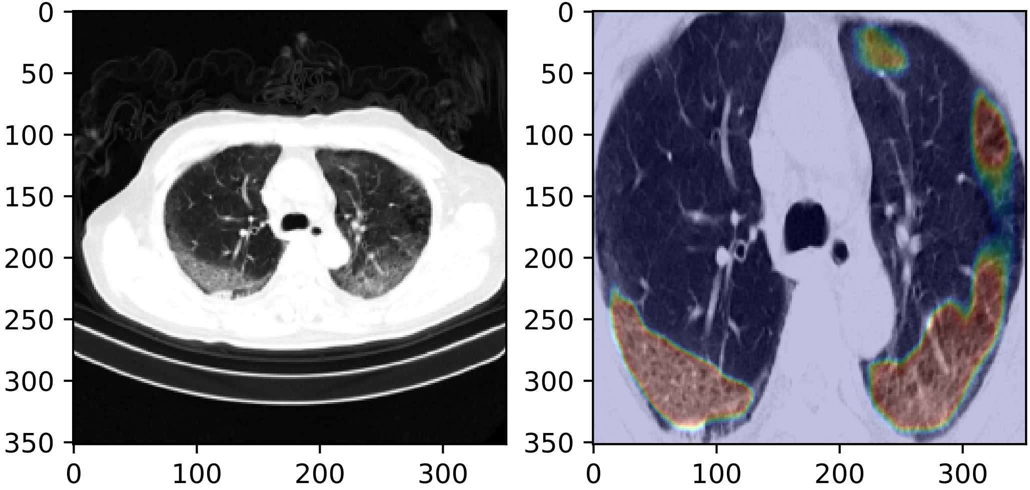

Lesion Proportion: 25.86%

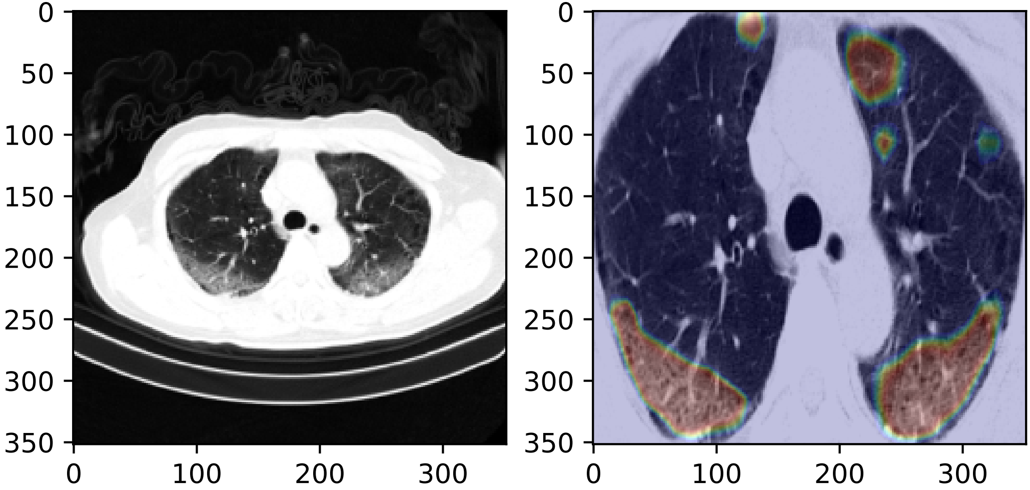

Lesion Proportion: 21.17%

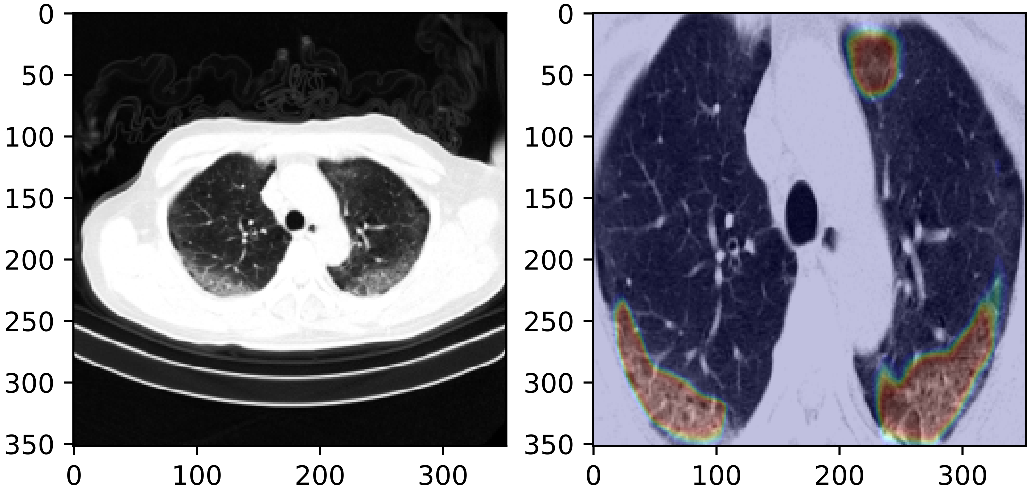

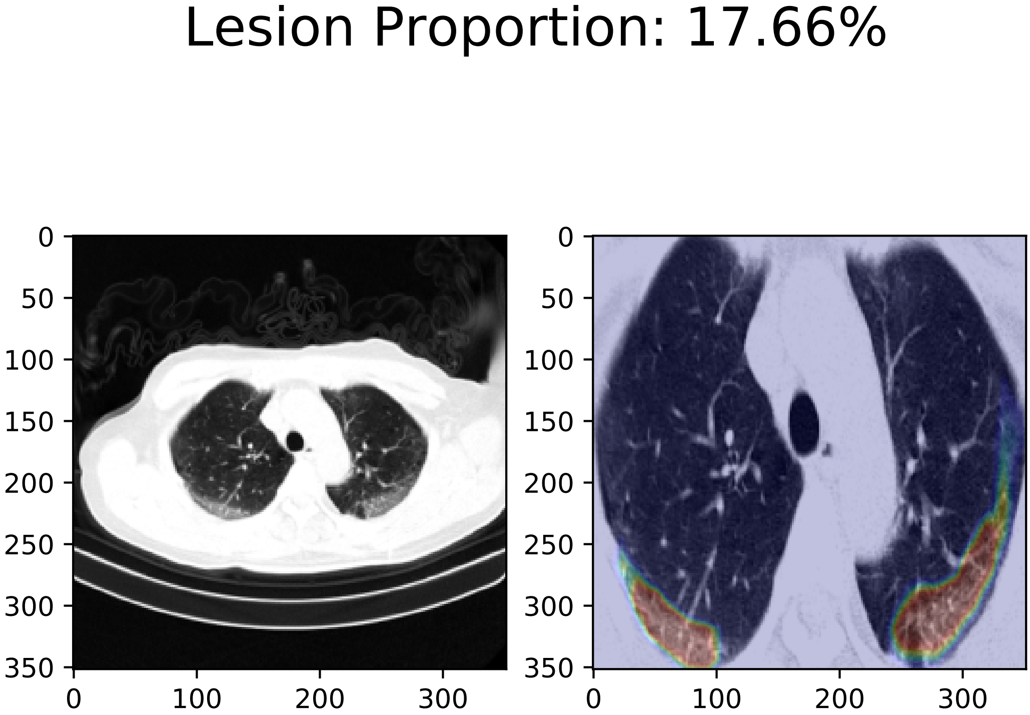
Lesion Proportion: 17.66%
0
50
100
150
200
250
300
350
0
100
200
300

Lesion Proportion: 11.63%

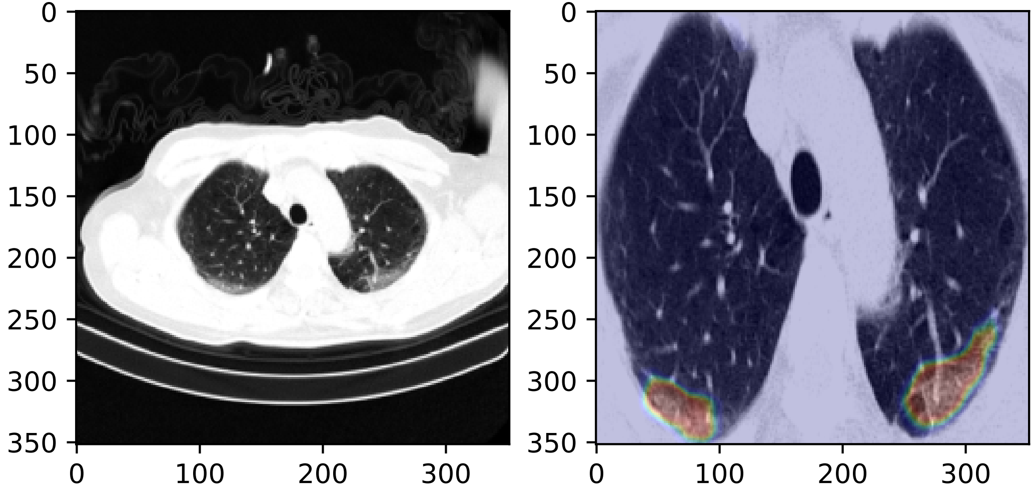

Lesion Proportion: 7.23%

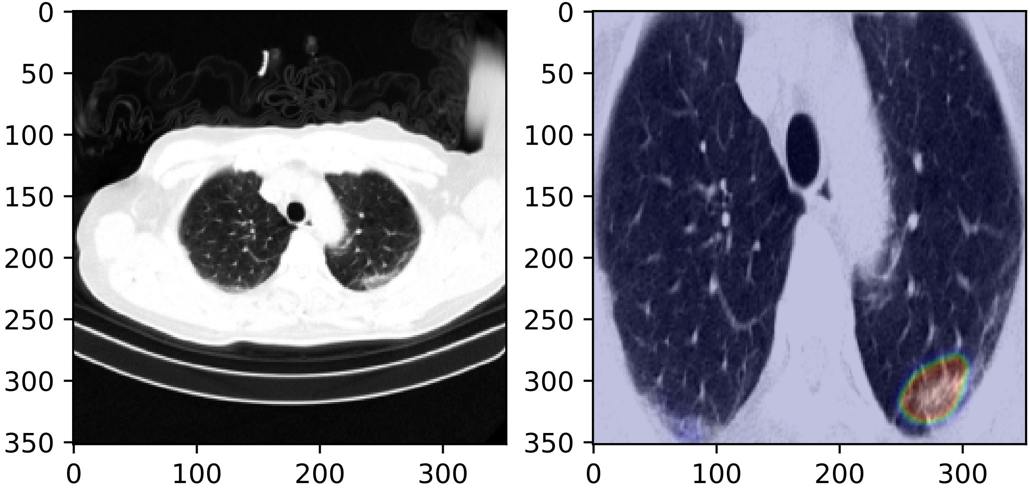

Lesion Proportion: 12.62%

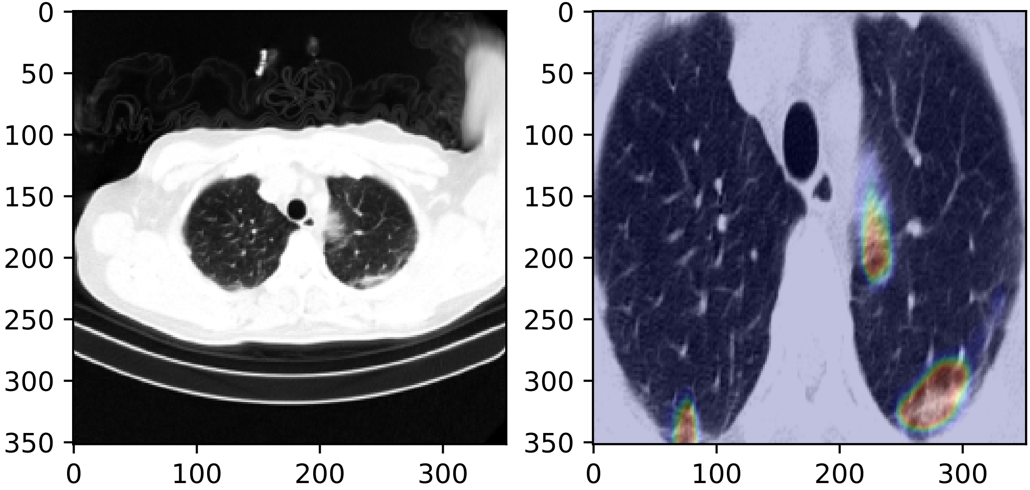

Lesion Proportion: 18.04%

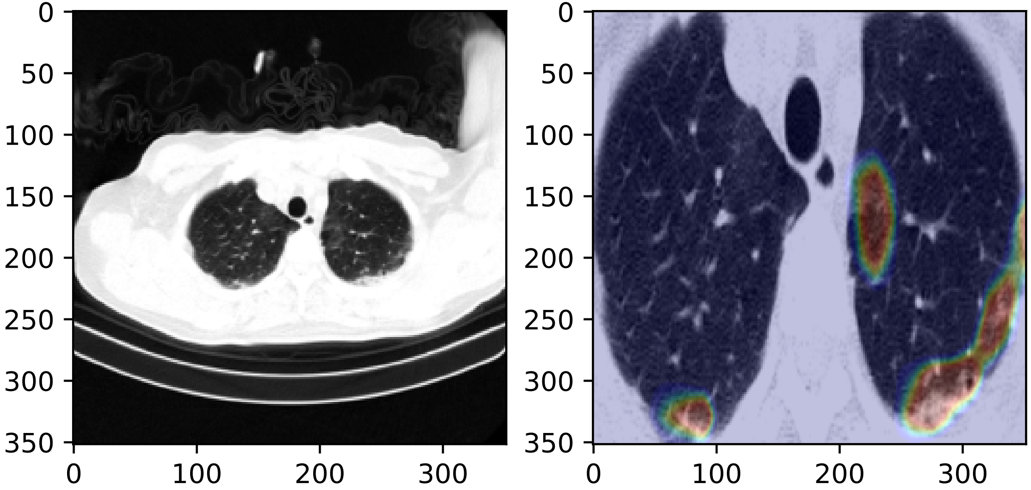

Lesion Proportion: 11.30%

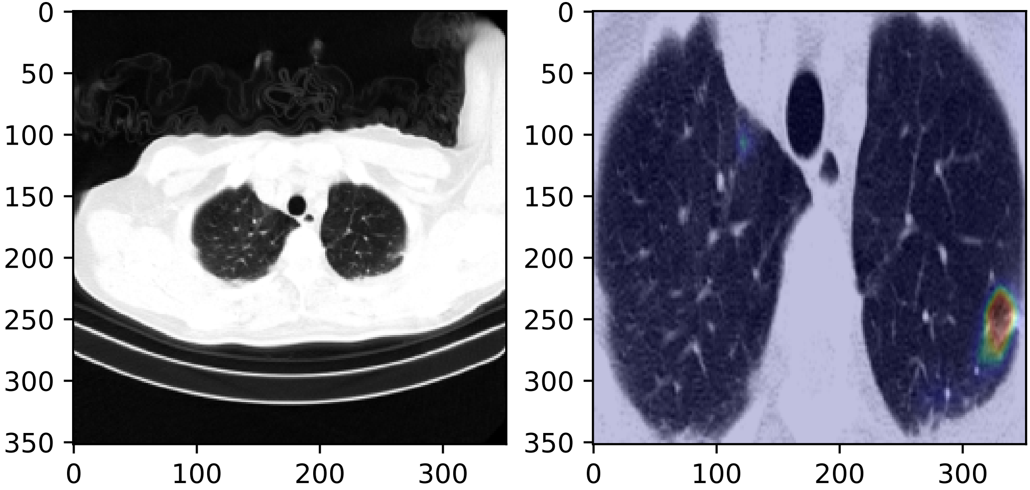

Lesion Proportion: 13.75%

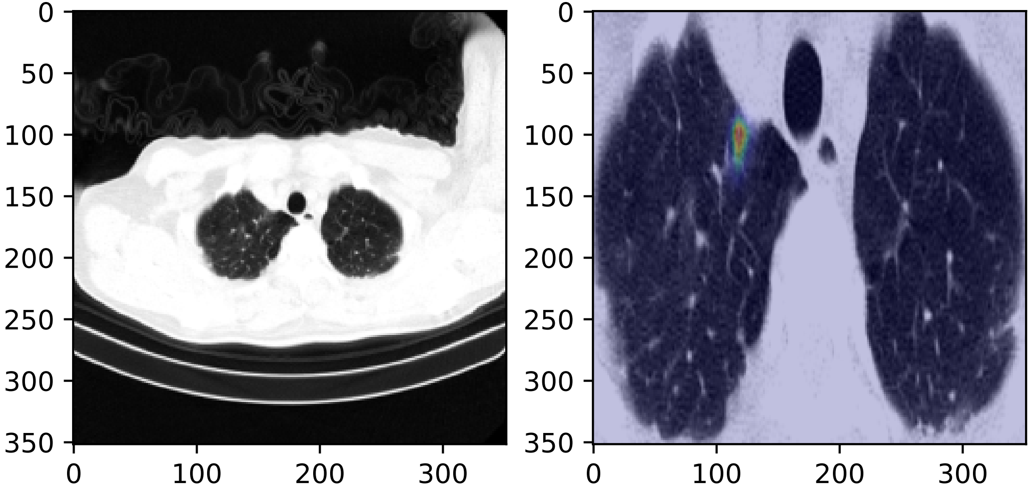

Lesion Proportion: 20.06%

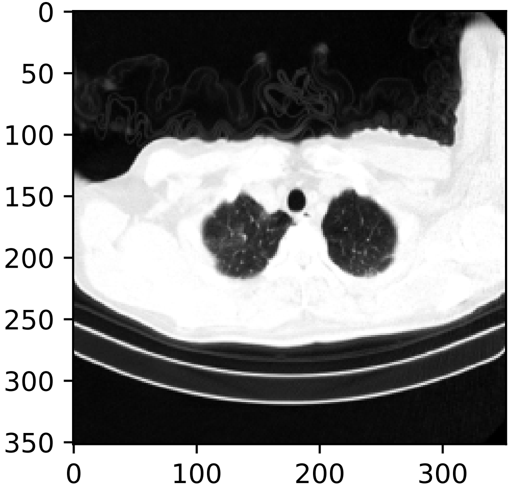

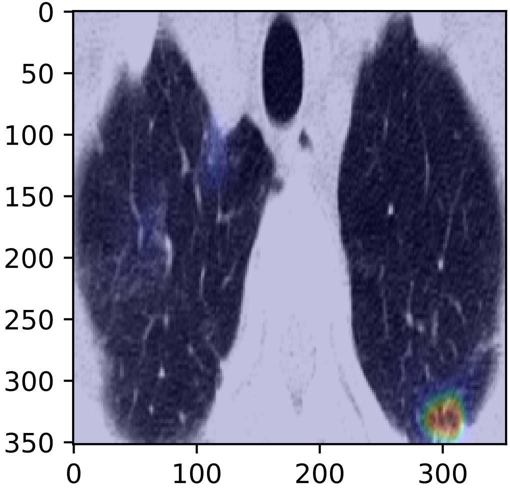

Lesion Proportion: 42.02%

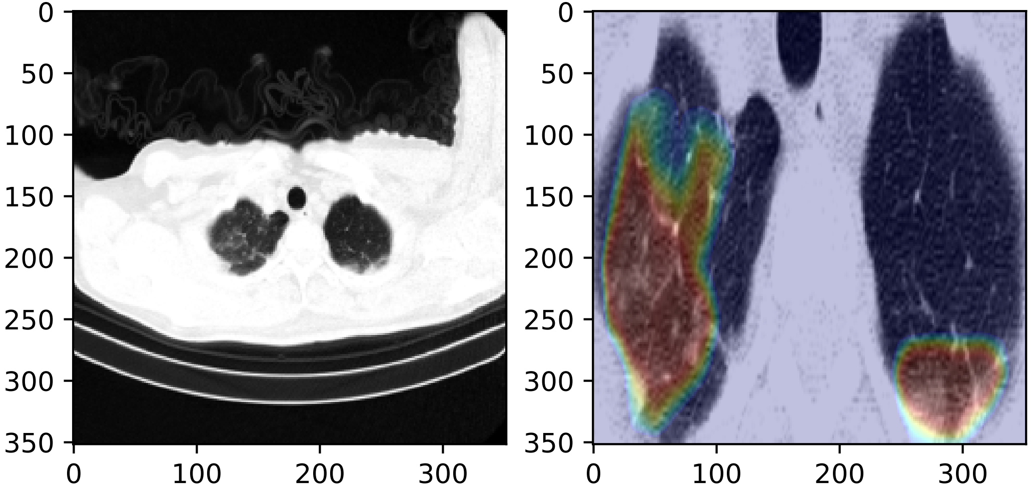

Lesion Proportion: 33.65%

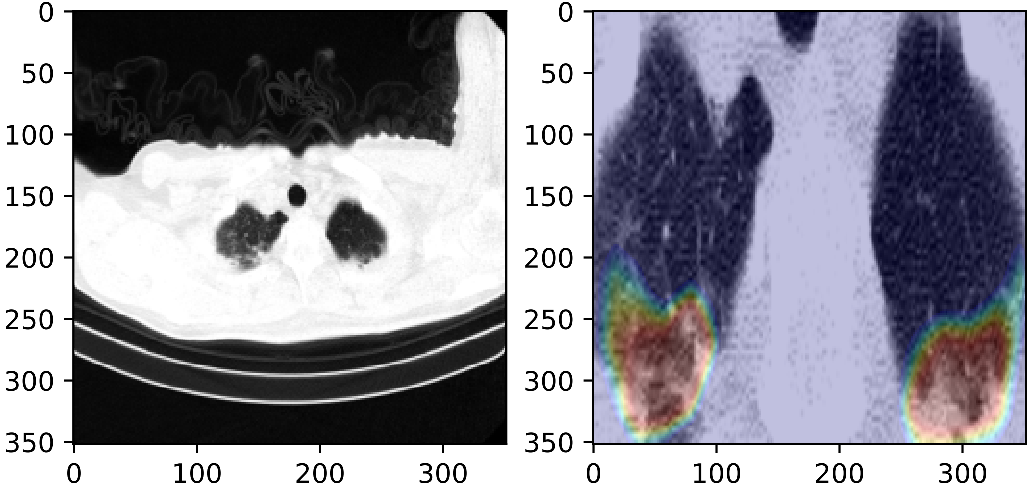

Lesion Proportion: 65.16%

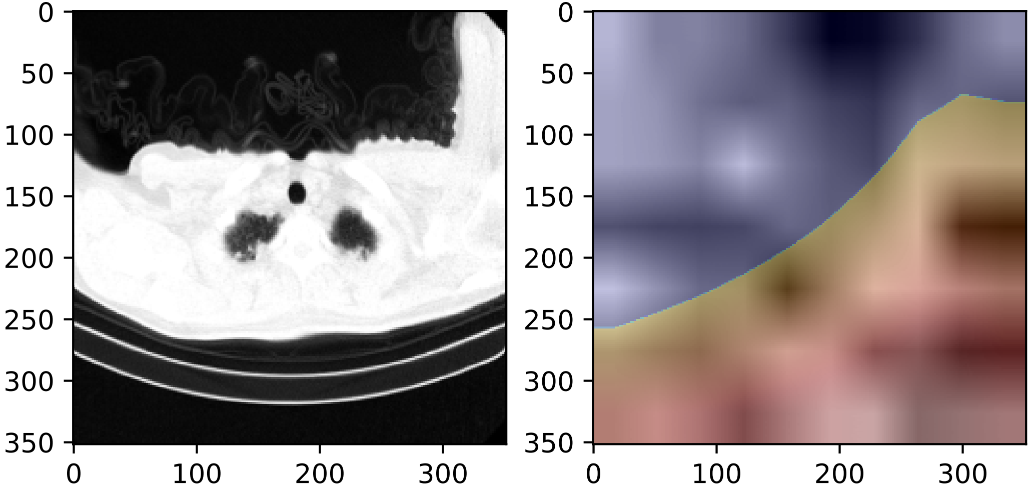

Supplement: Supplementary file 4 [file DataSheet4.pdf]
