# Supplementary material for: Medical decision support system using weakly-labeled lung CT scans
Source: Front Med Technol. 2022 Sep 28;4:980735. doi: 10.3389/fmedt.2022.980735 (PMC9554434; doi:10.3389/fmedt.2022.980735)

Lesion Proportion: 10.65%

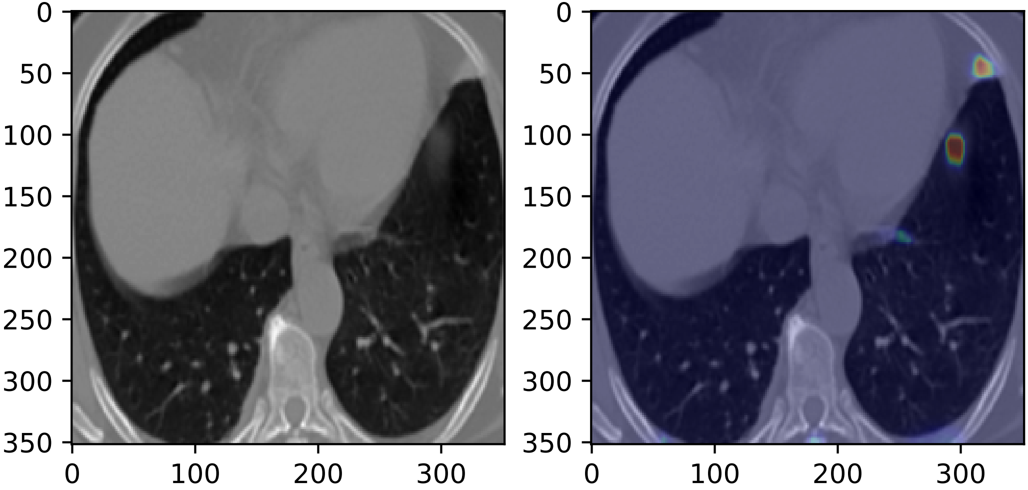

Lesion Proportion: 8.34%

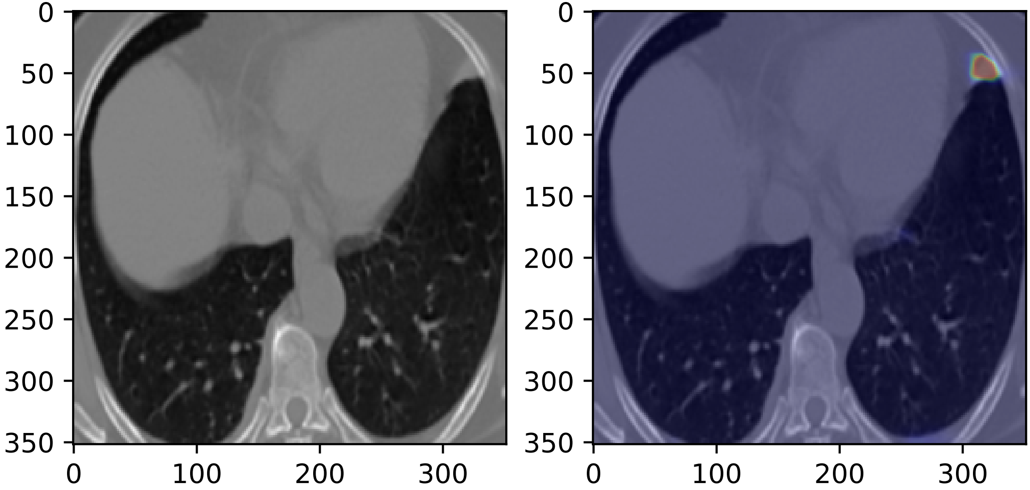

Lesion Proportion: 7.85%

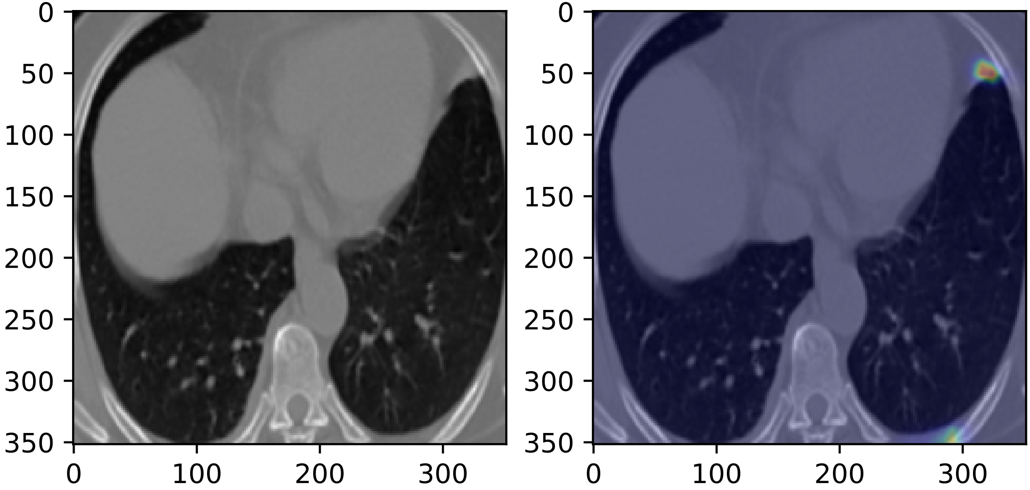

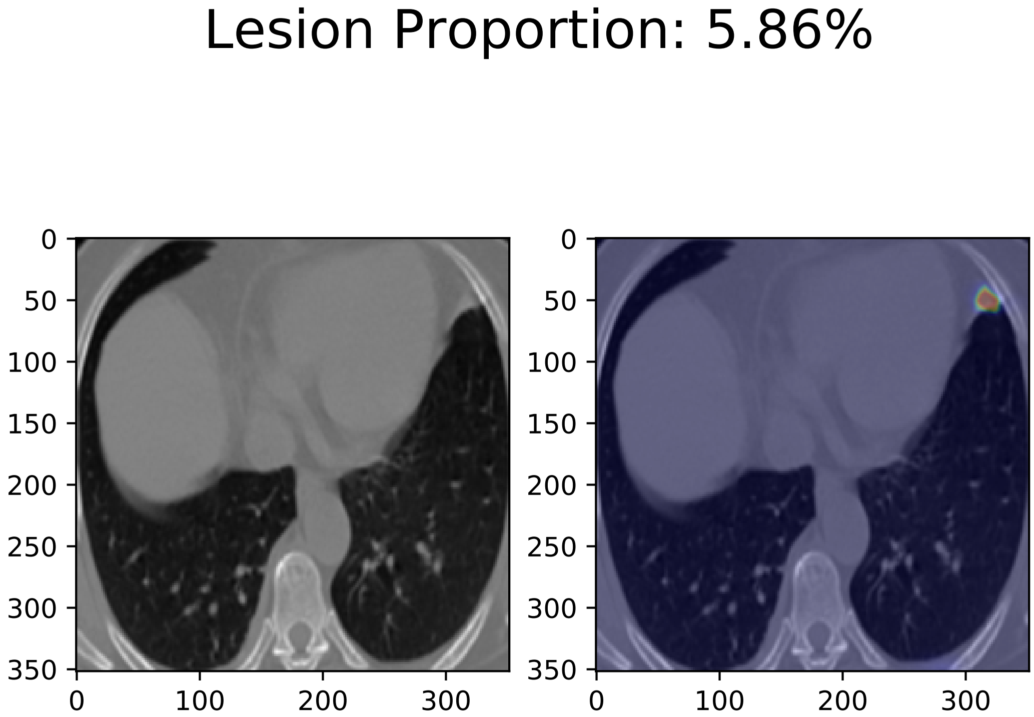
Lesion Proportion: 5.86%
0
50
100
150
200
250
300
350
0
100
200
300
0
50
100
150
200
250
300
350
0
100
200
300

Lesion Proportion: 8.49%

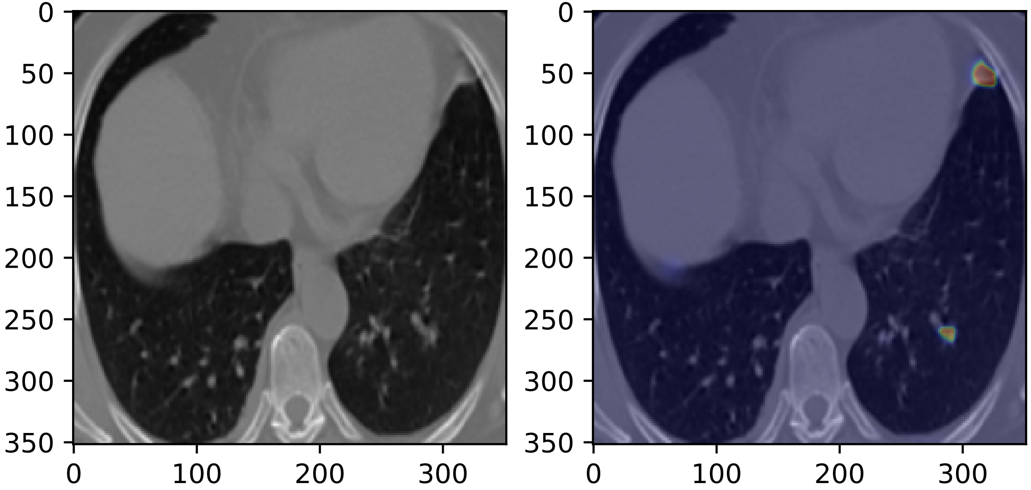

Lesion Proportion: 7.36%

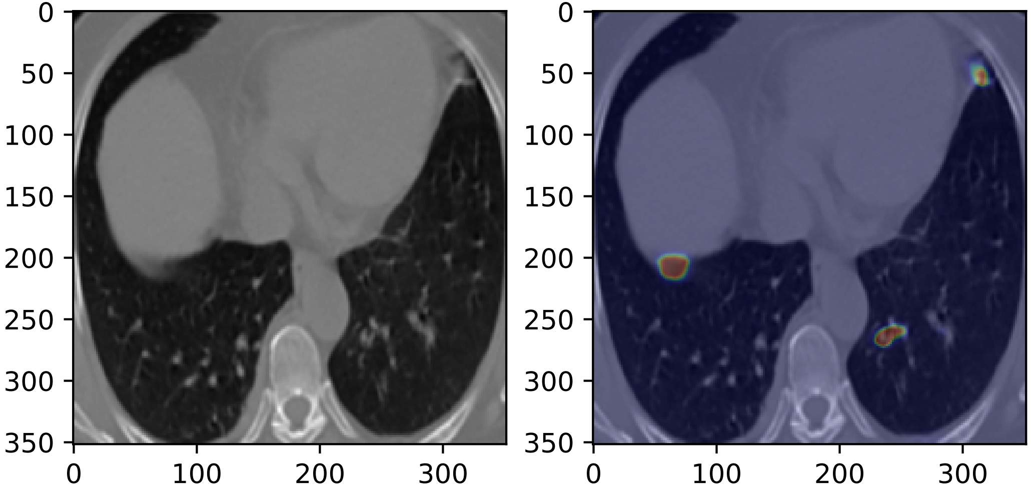

Lesion Proportion: 3.97%

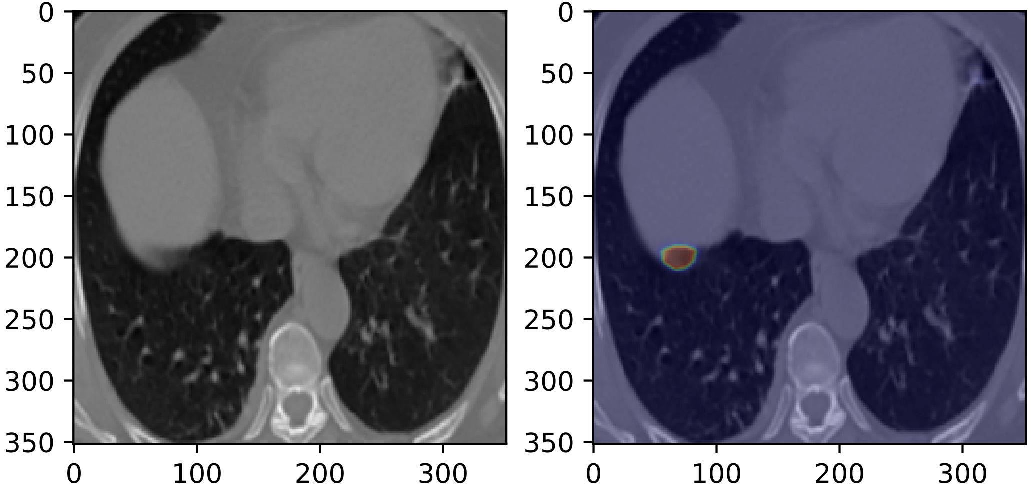

Lesion Proportion: 5.29%

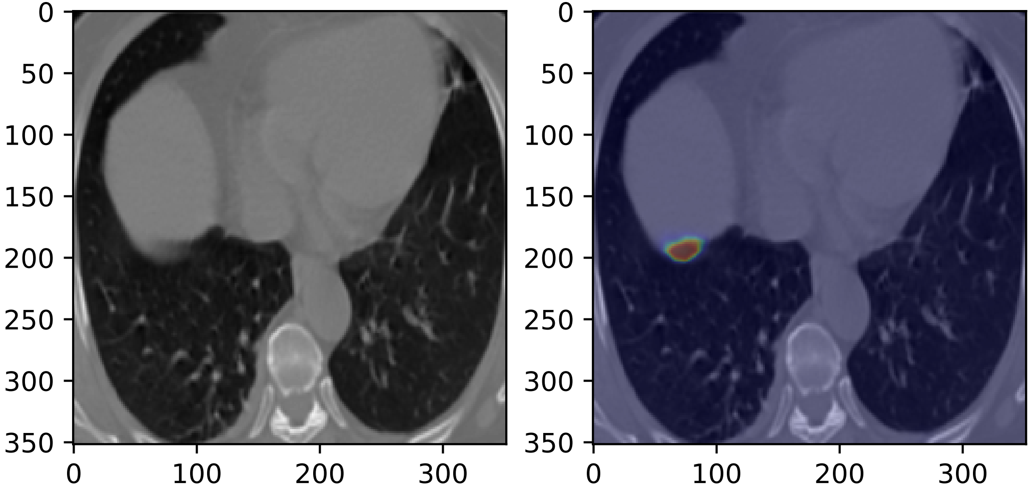

Lesion Proportion: 8.17%

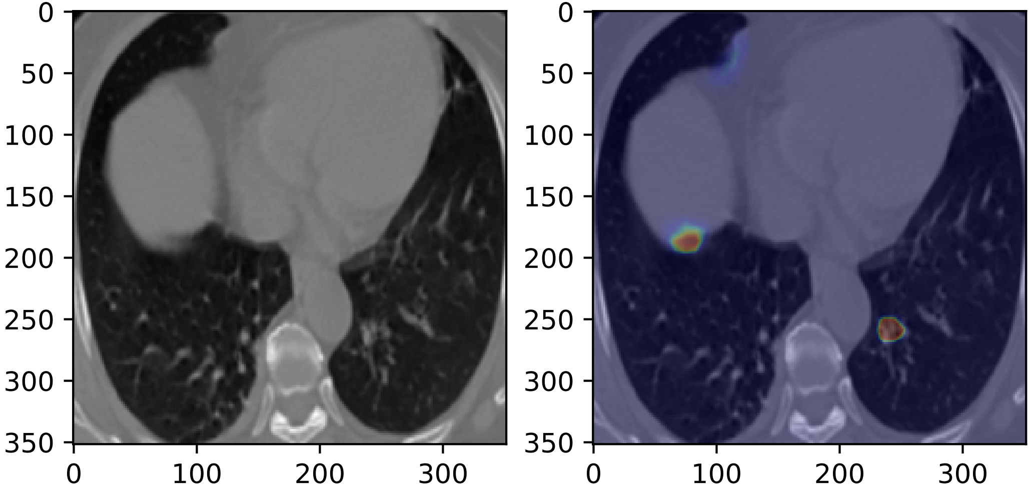

Lesion Proportion: 14.08%

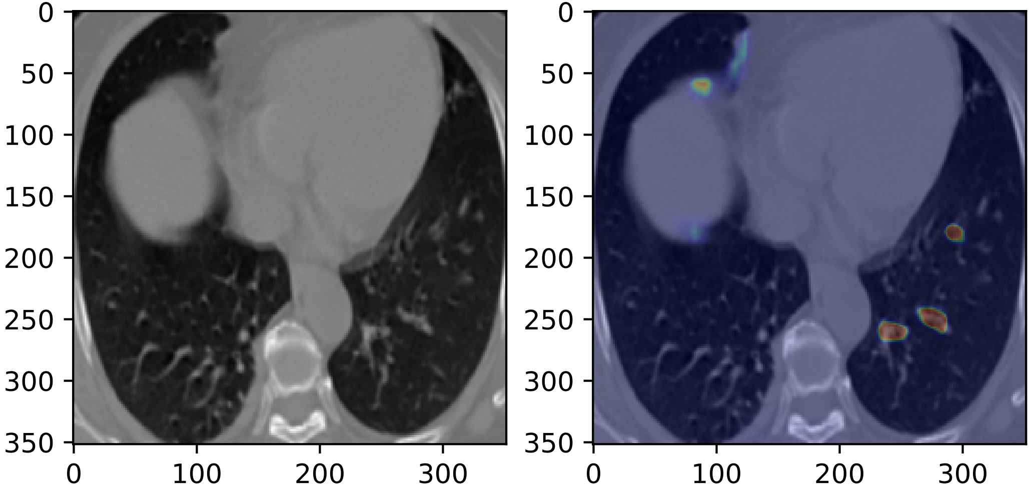

Lesion Proportion: 11.74%

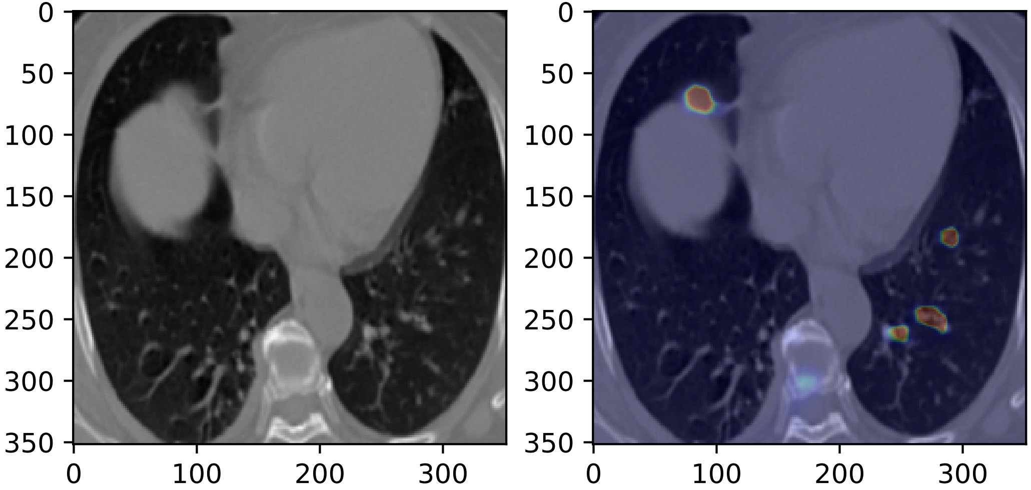

Lesion Proportion: 8.52%

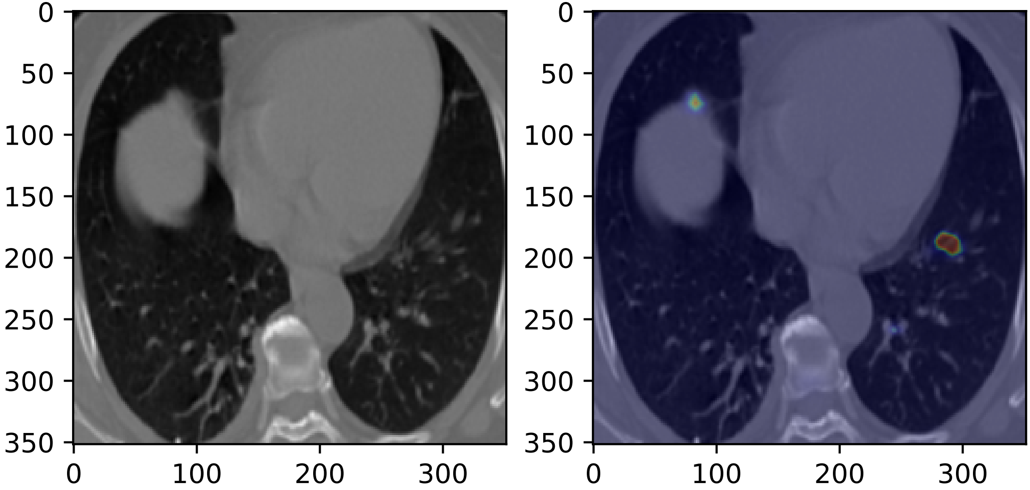

Lesion Proportion: 17.65%

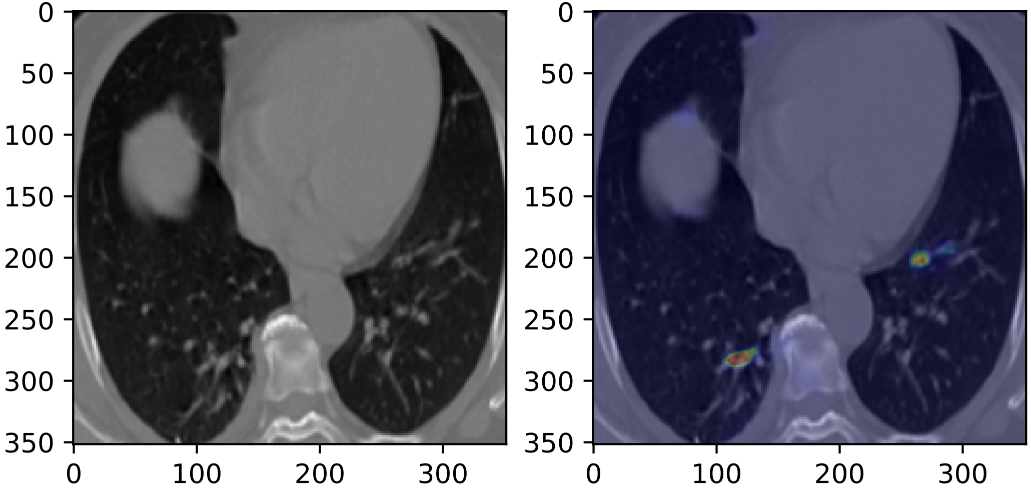

Lesion Proportion: 11.70%

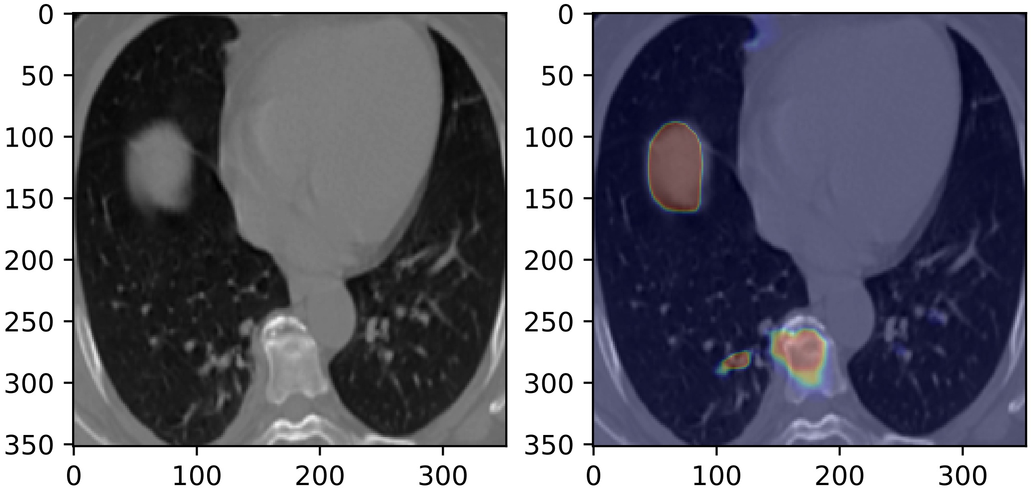

Lesion Proportion: 12.13%

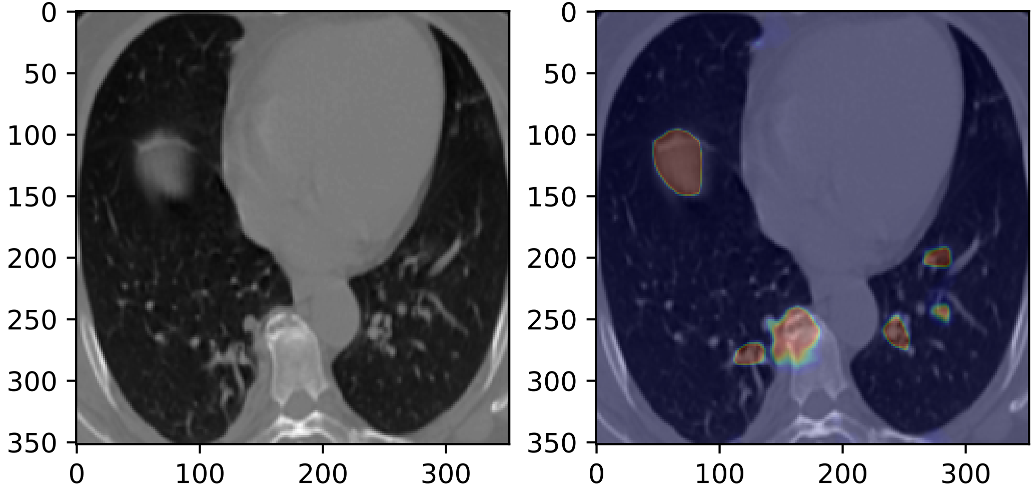

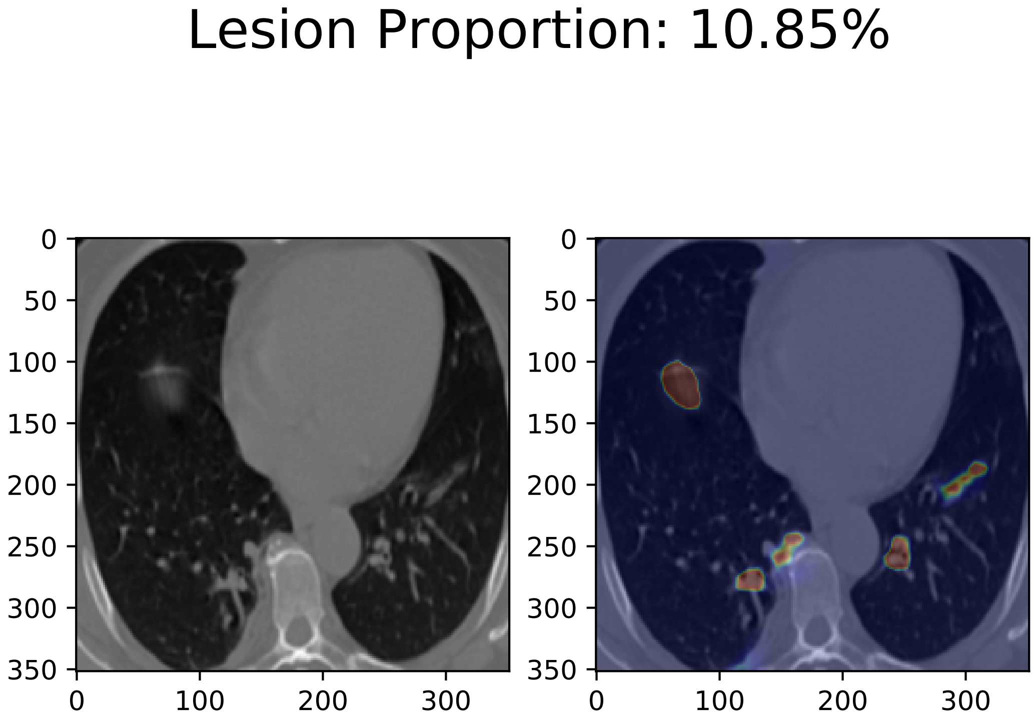

Lesion Proportion: 10.85%
0
50
100
150
200
250
300
350
0
100
200
300
0
50
100
150
200
250
300
350
0
100
200
300

Lesion Proportion: 13.65%

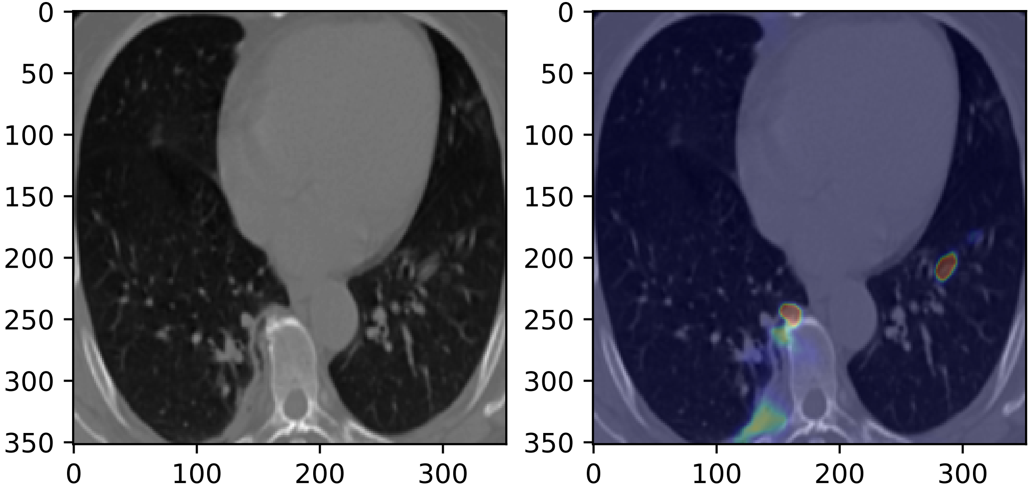

Lesion Proportion: 15.58%

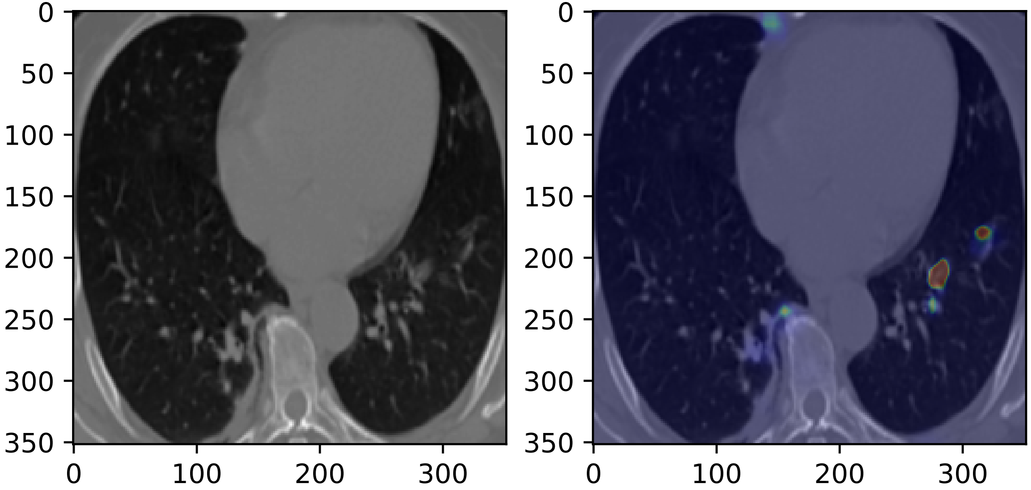

Lesion Proportion: 12.39%

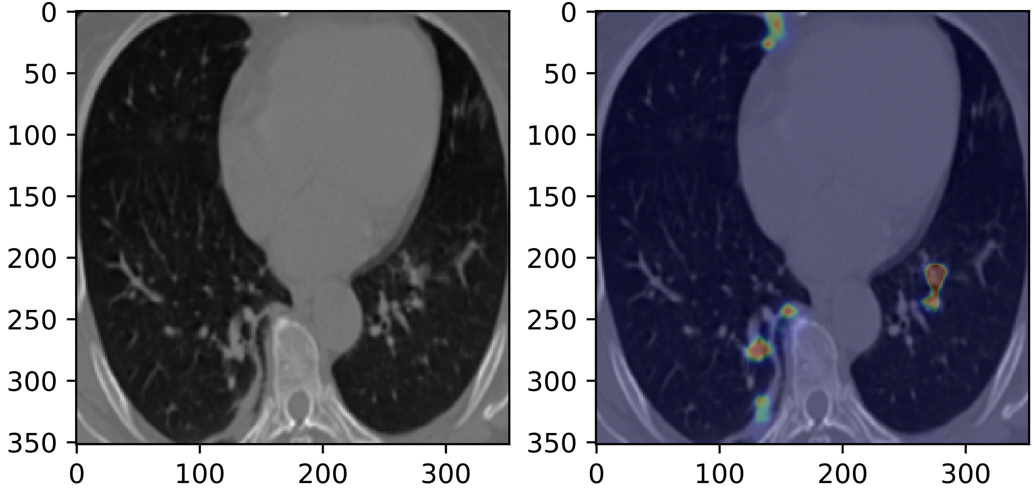

Lesion Proportion: 8.62%

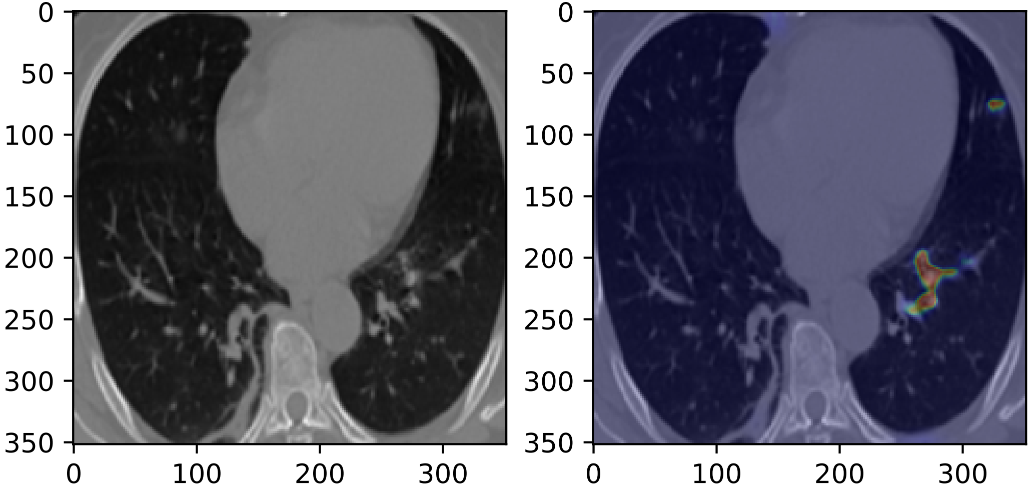

Lesion Proportion: 7.87%

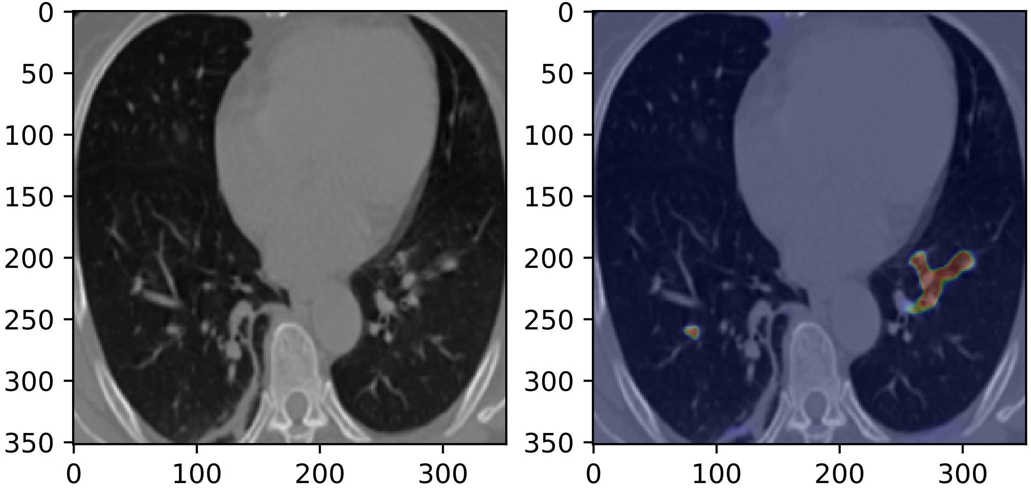

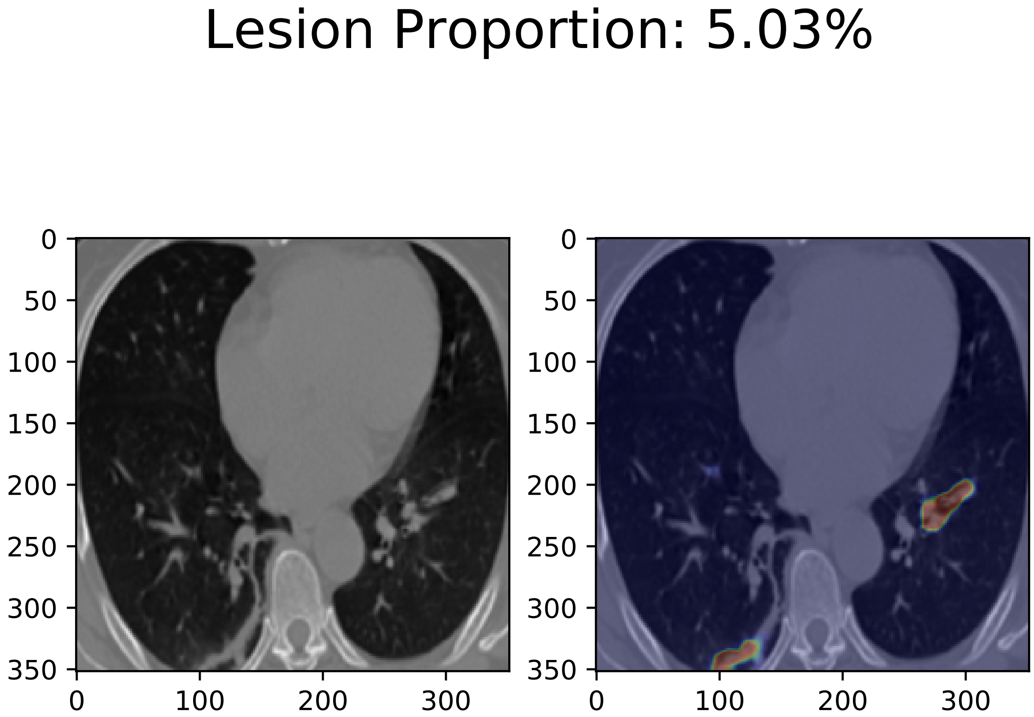
Lesion Proportion: 5.03%
0
50
100
150
200
250
300
350
0
100
200
300
0
50
100
150
200
250
300
350
0
100
200
300

Lesion Proportion: 7.97%

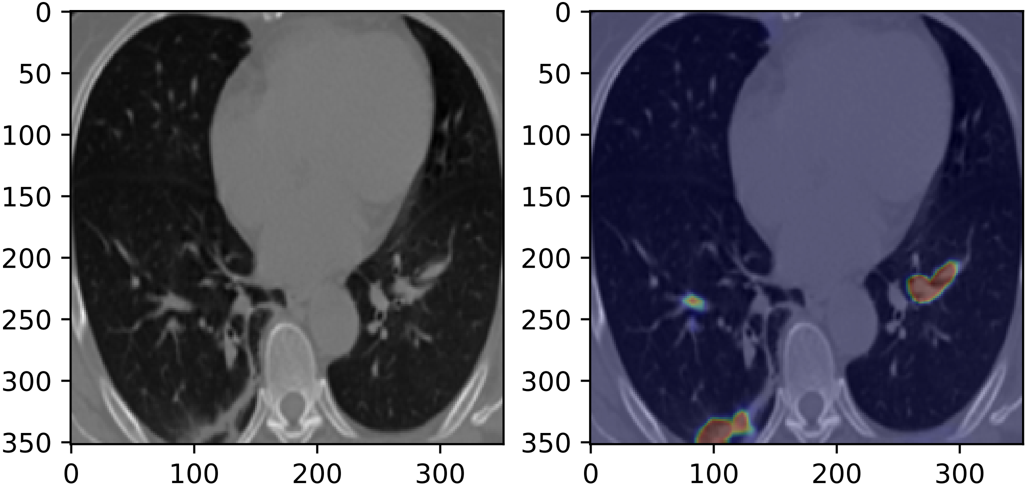

Lesion Proportion: 8.39%

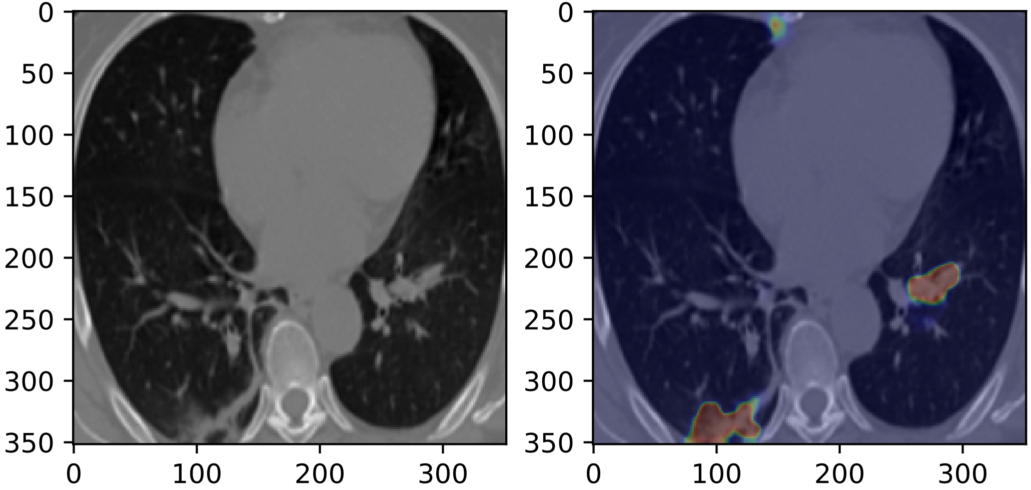

Lesion Proportion: 11.43%

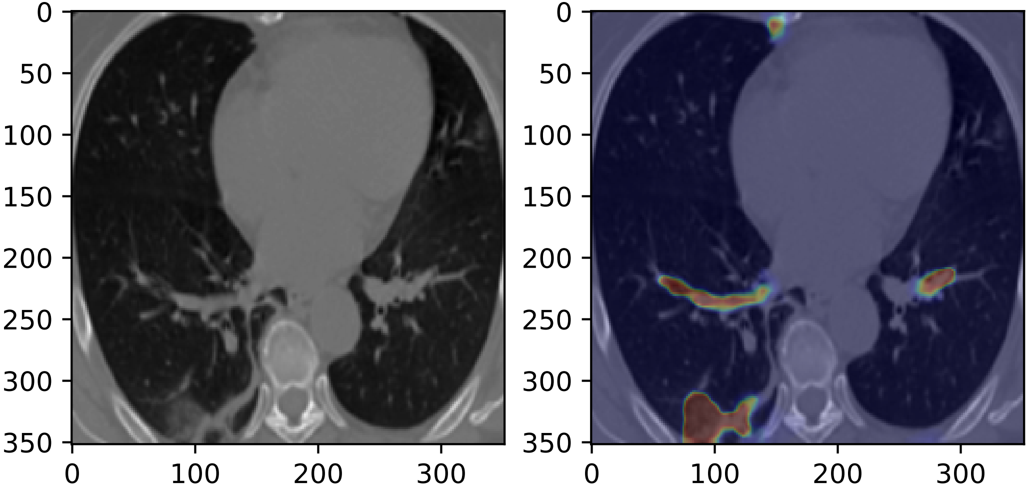

Lesion Proportion: 8.92%

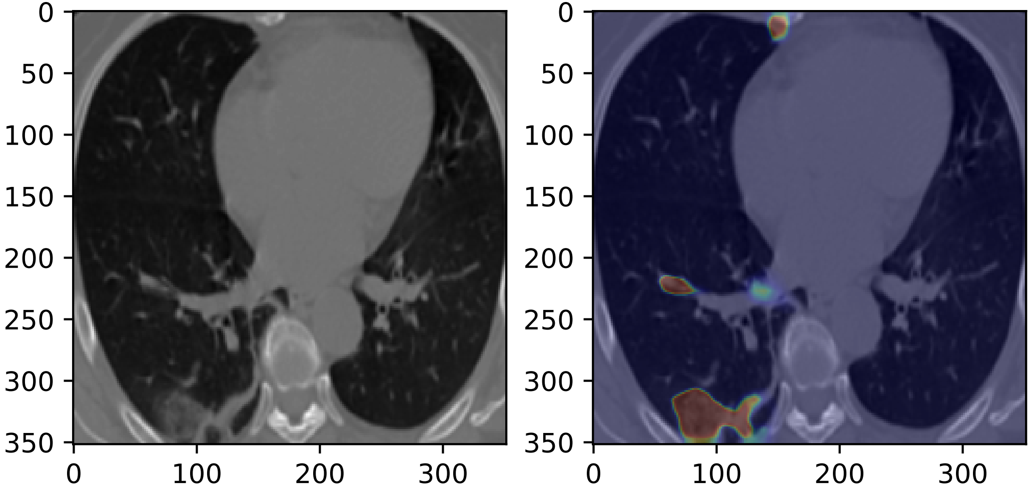

Lesion Proportion: 9.29%

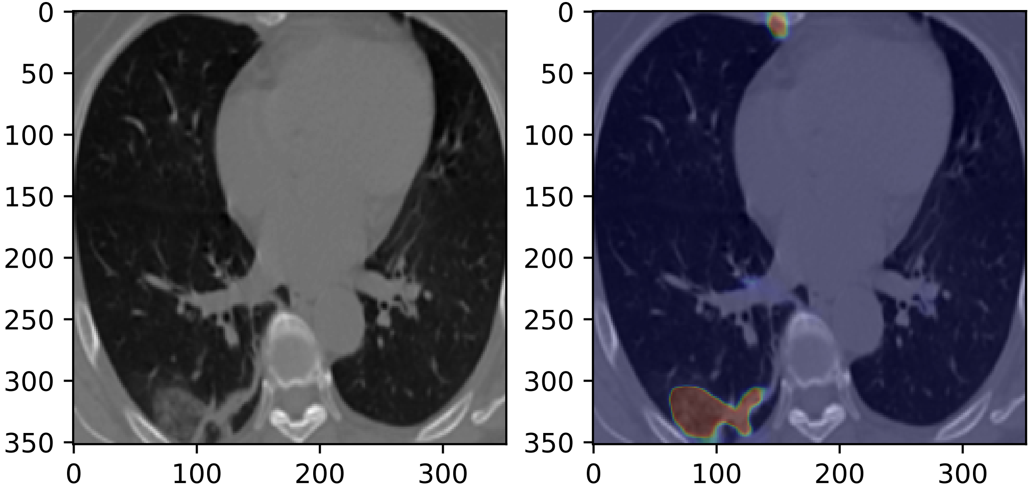

Lesion Proportion: 12.03%

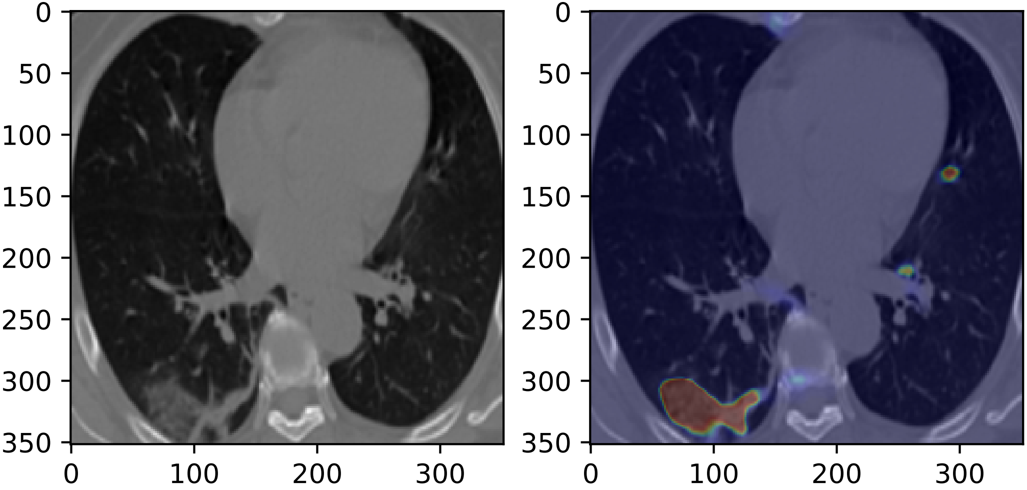

Lesion Proportion: 17.12%

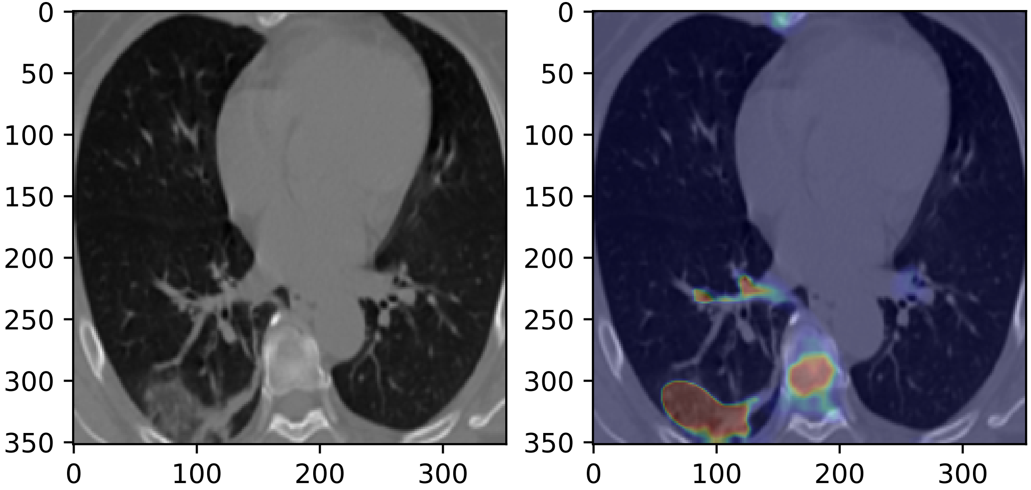

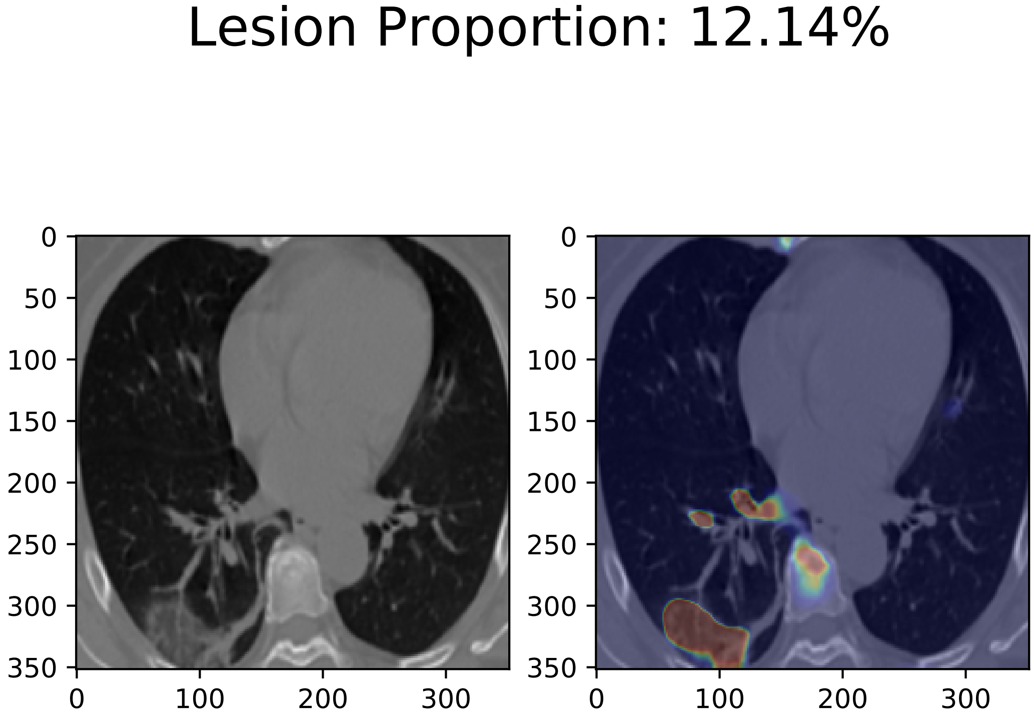
Lesion Proportion: 12.14%
0
50
100
150
200
250
300
350
0
100
200
300
0
50
100
150
200
250
300
350
0
100
200
300

Lesion Proportion: 7.94%

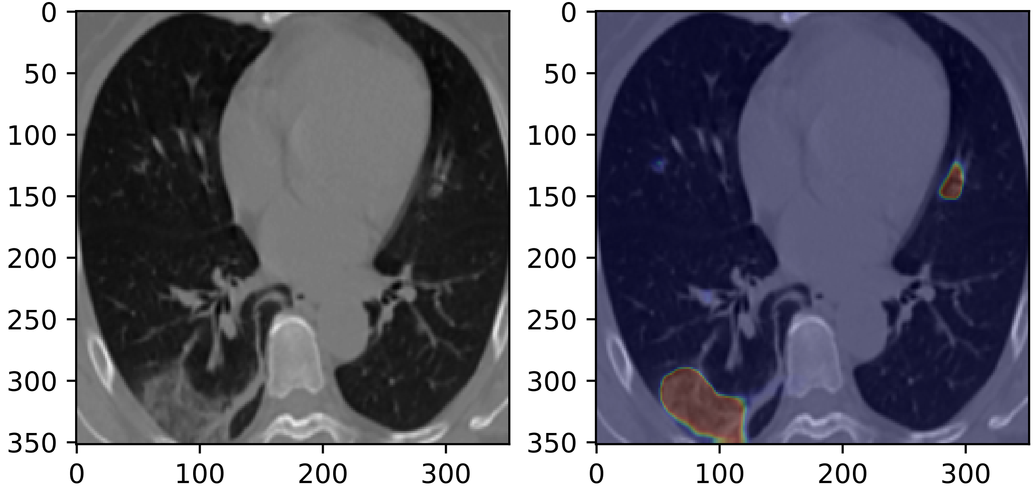

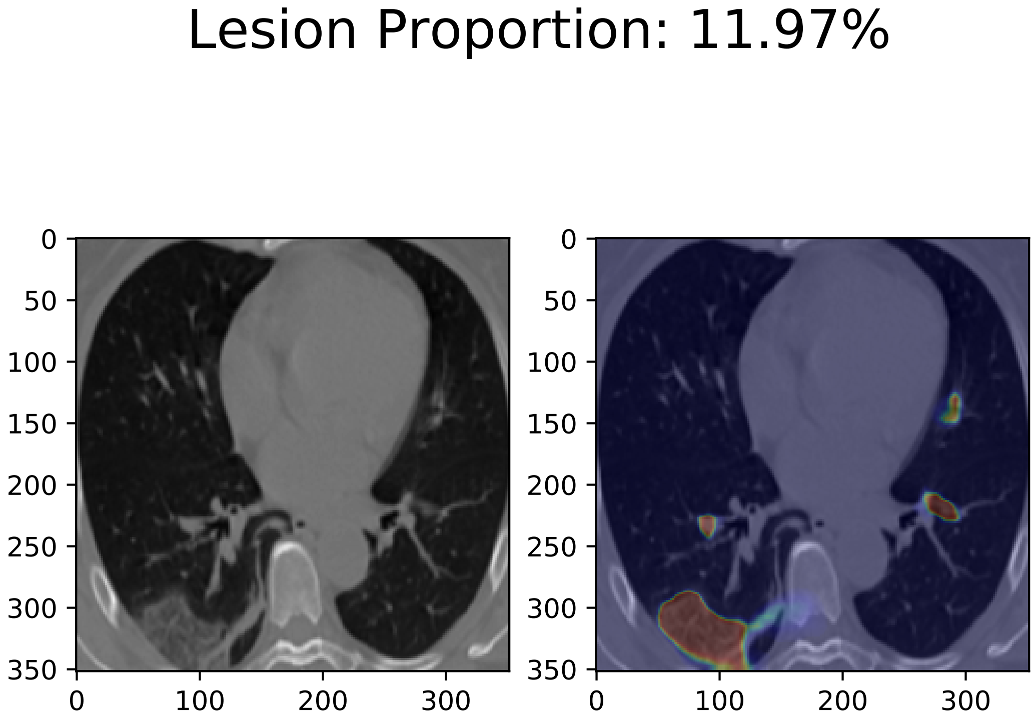
Lesion Proportion: 11.97%
0
50
100
150
200
250
300
350
0
100
200
300

Lesion Proportion: 10.41%

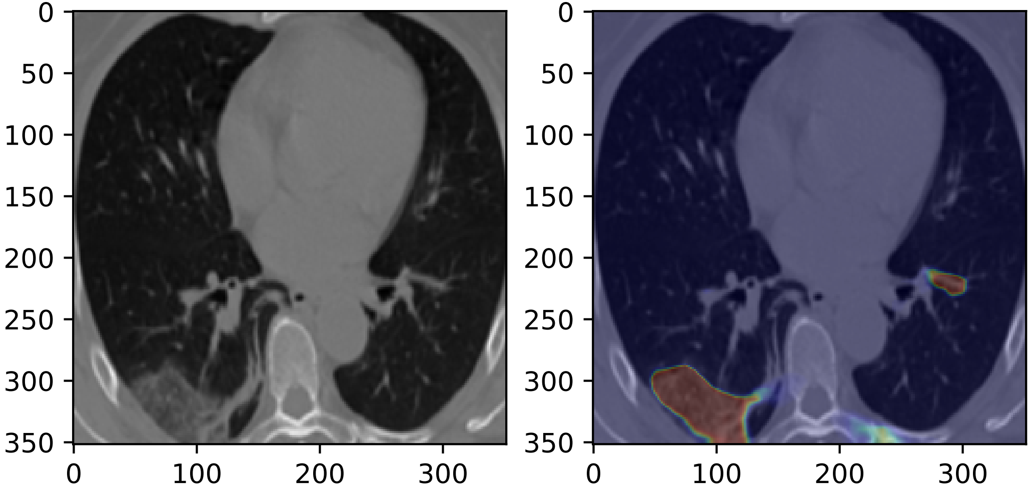

Lesion Proportion: 13.80%

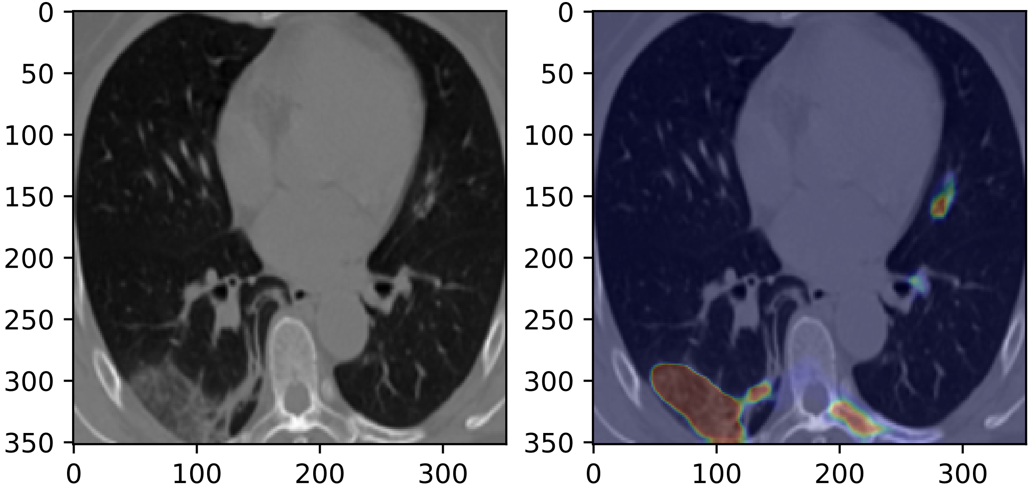

Lesion Proportion: 14.78%

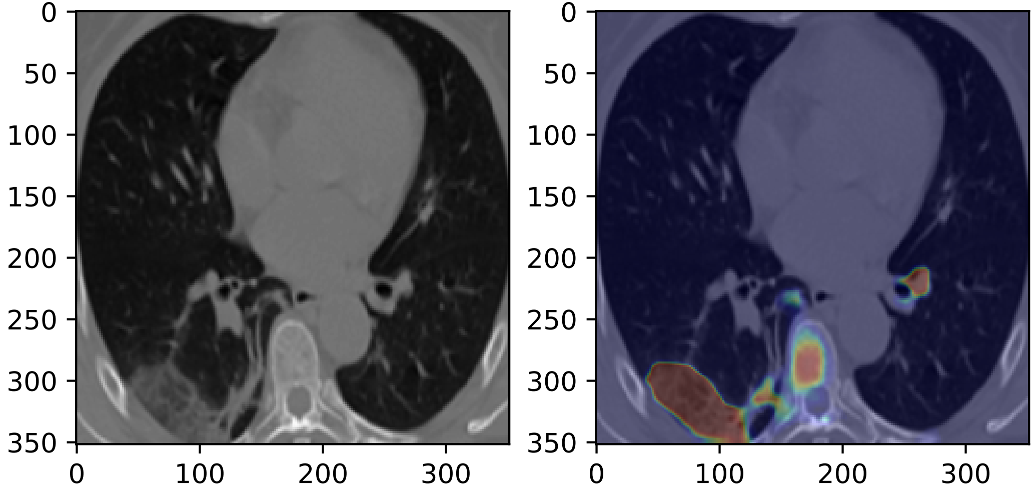

Lesion Proportion: 10.82%

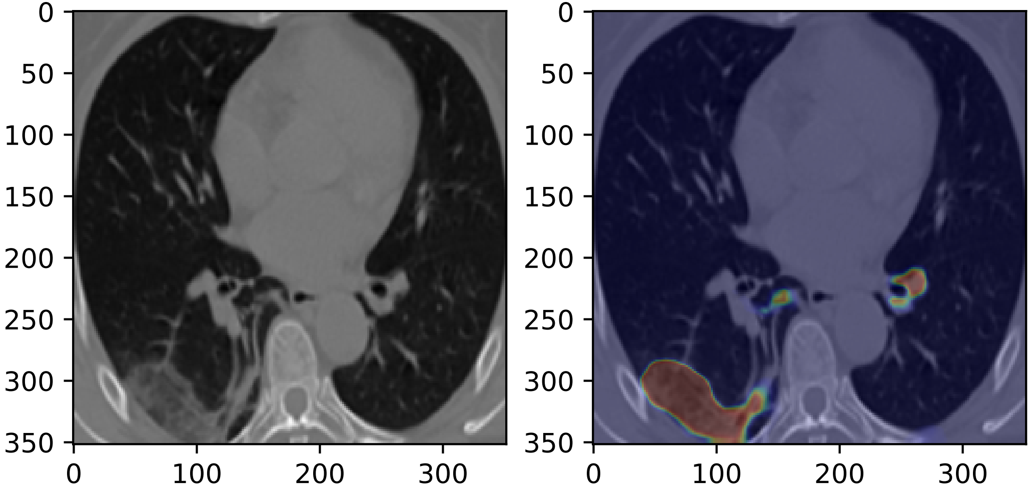

Lesion Proportion: 11.78%

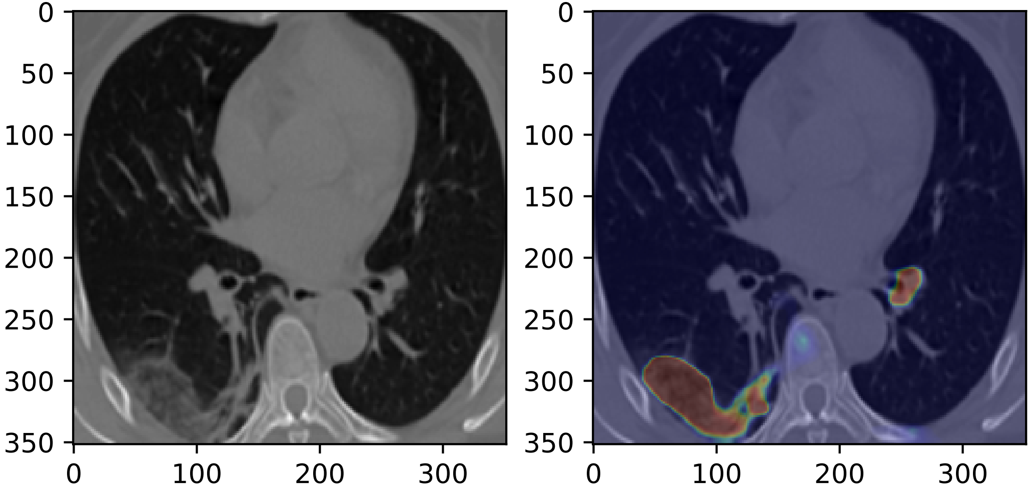

Lesion Proportion: 12.55%

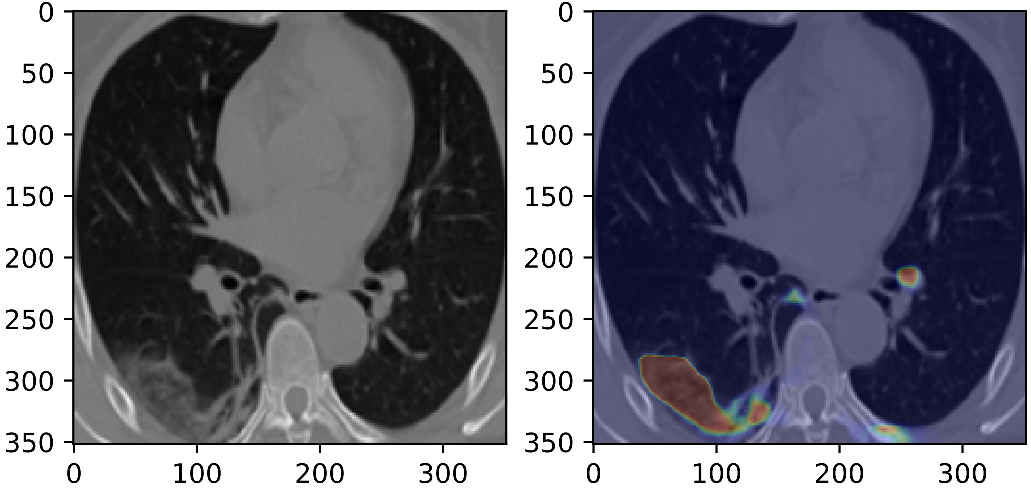

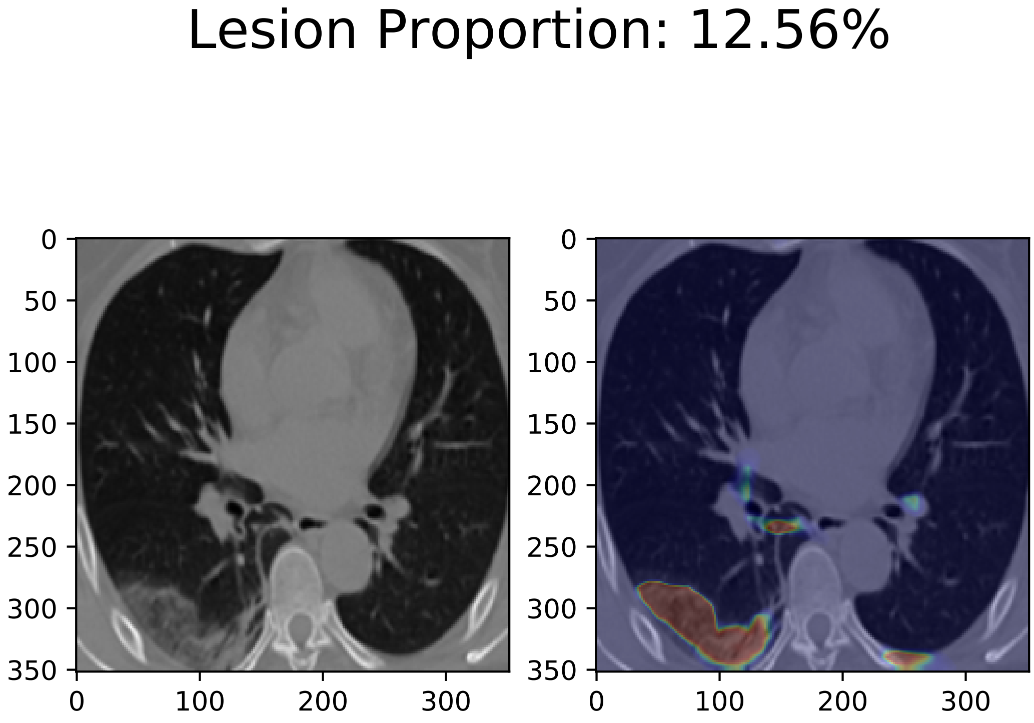

Lesion Proportion: 12.56%
0
50
100
150
200
250
300
350
0
100
200
300

Lesion Proportion: 10.46%

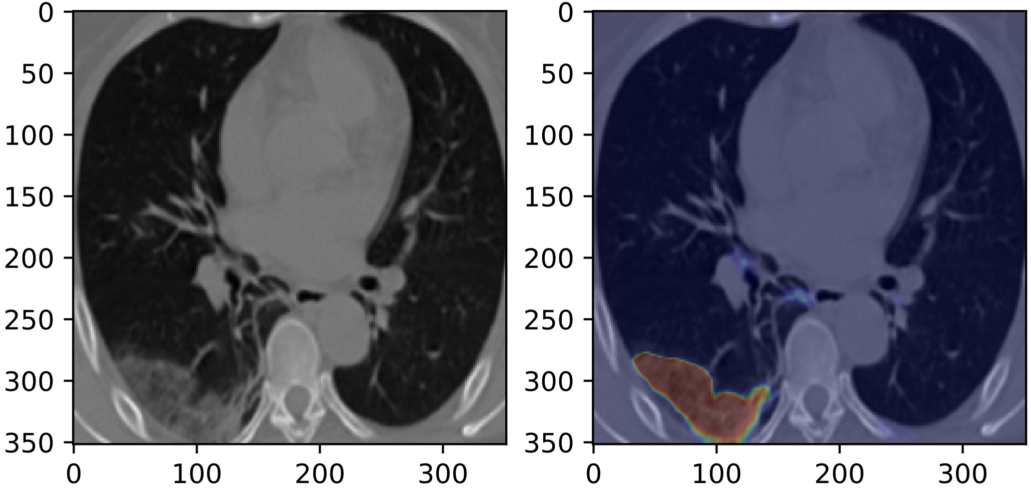

Lesion Proportion: 12.04%

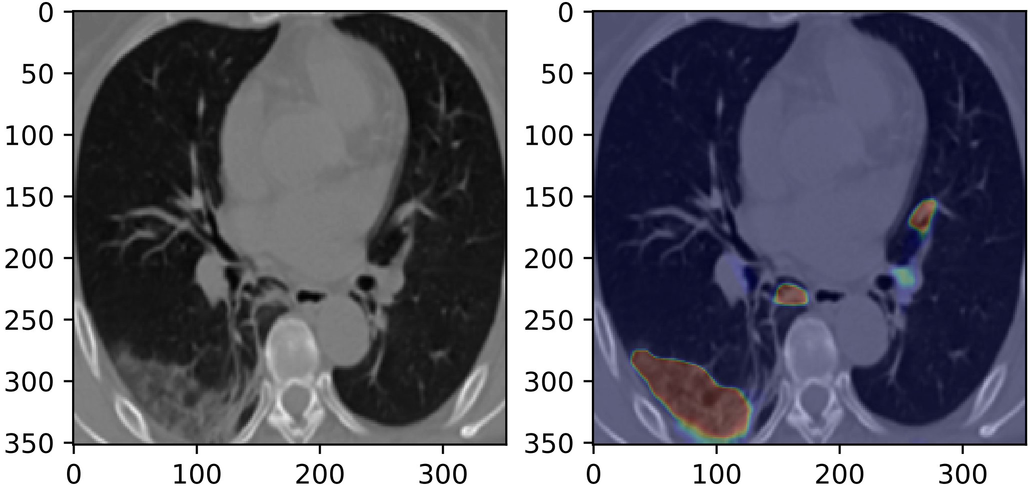

Lesion Proportion: 13.79%

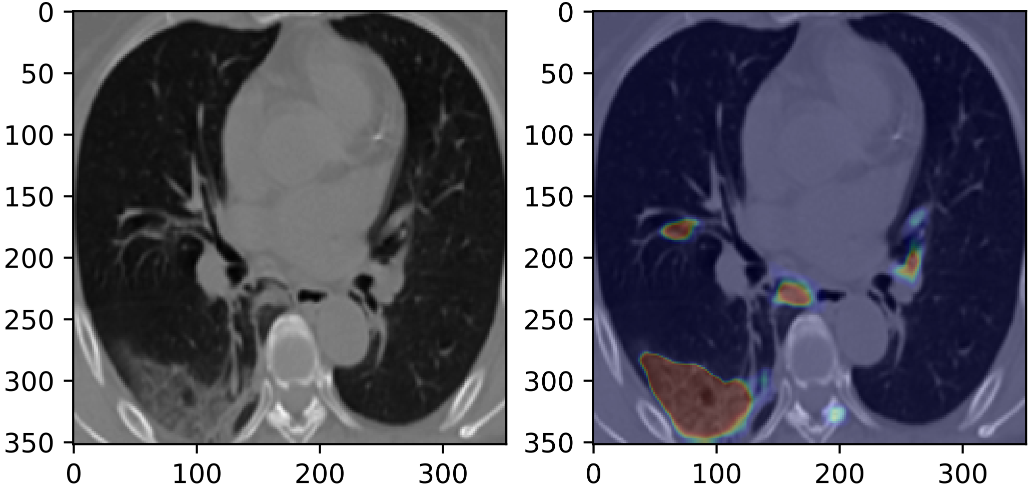

Lesion Proportion: 14.93%

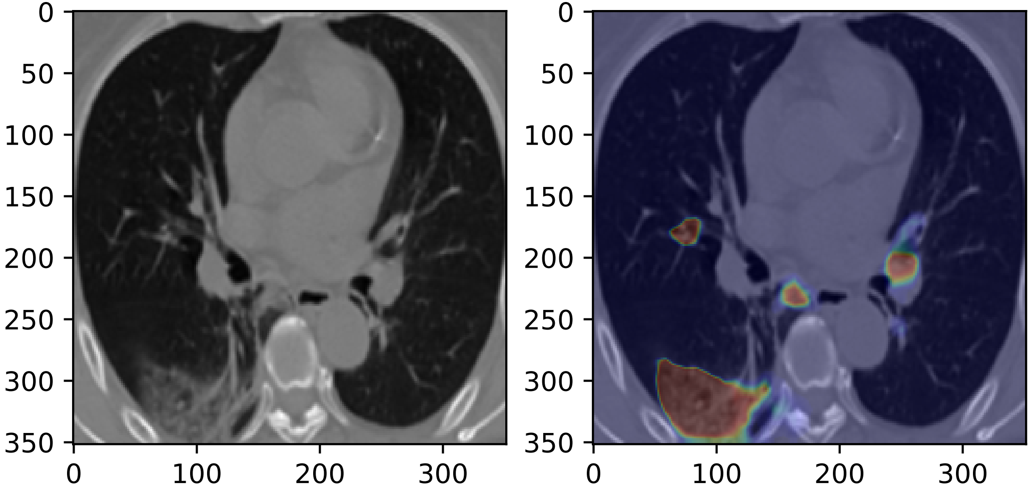

Lesion Proportion: 13.29%

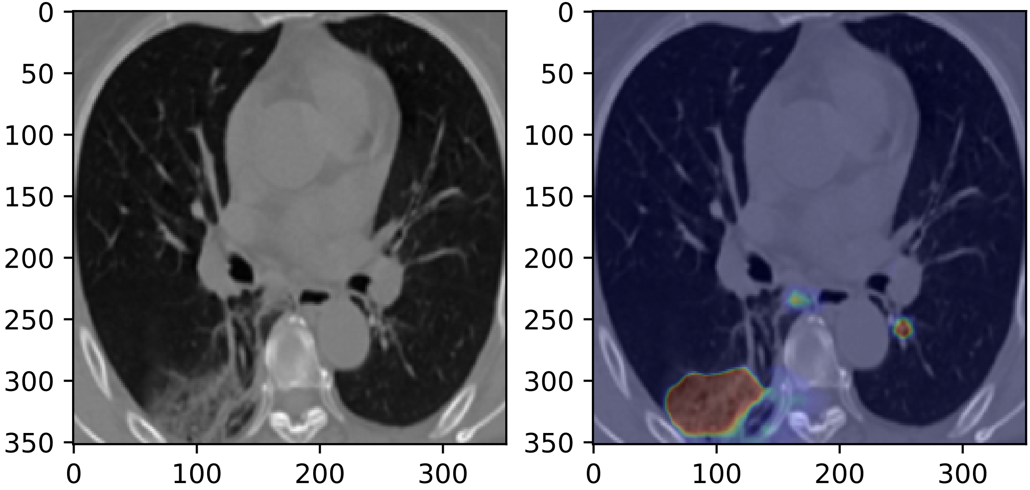

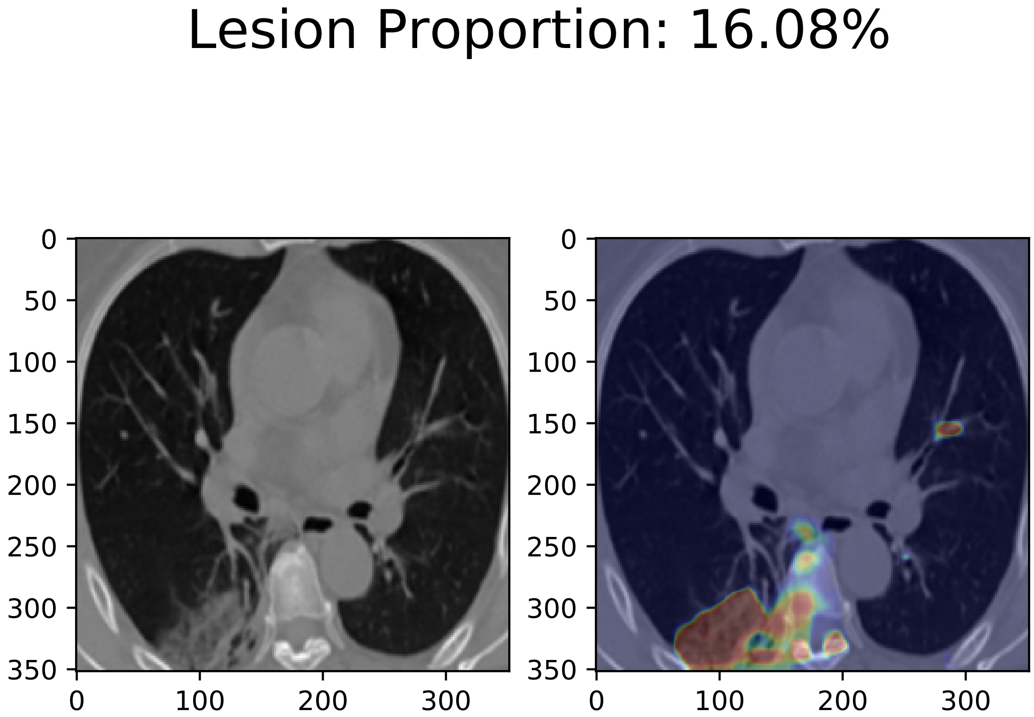

Lesion Proportion: 16.08%
0
50
100
150
200
250
300
350
0
100
200
300

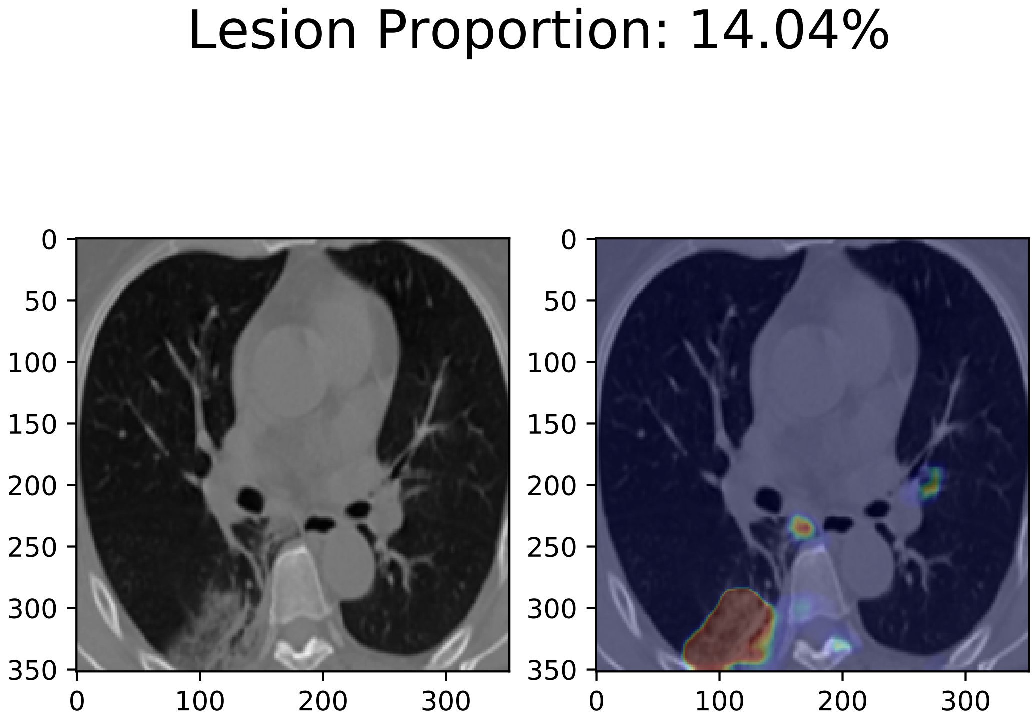
Lesion Proportion: 14.04%
0
50
100
150
200
250
300
350
0
100
200
300

Lesion Proportion: 9.26%

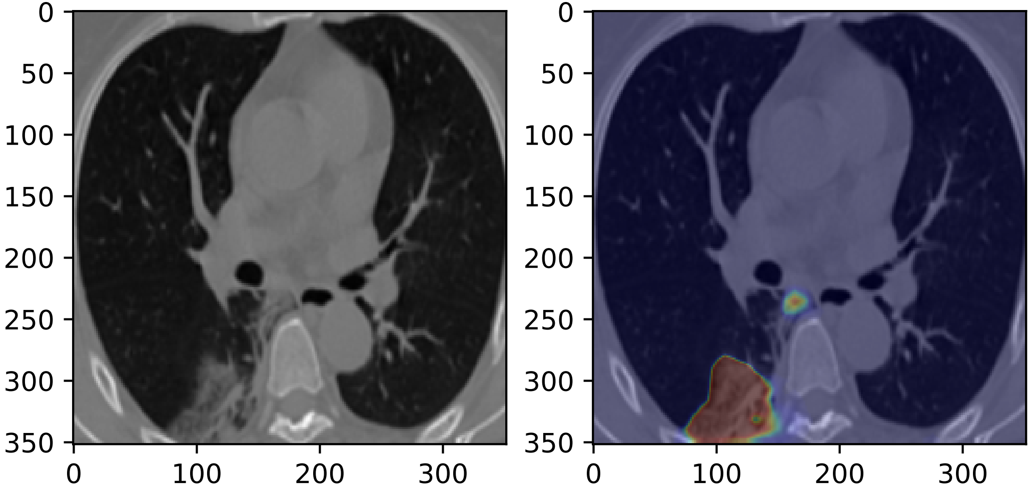

Lesion Proportion: 12.02%

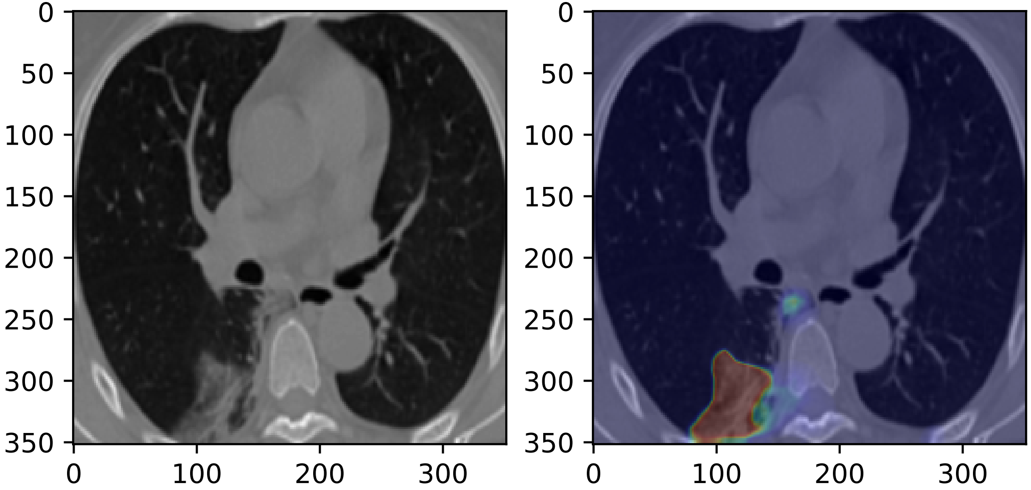

Lesion Proportion: 10.19%

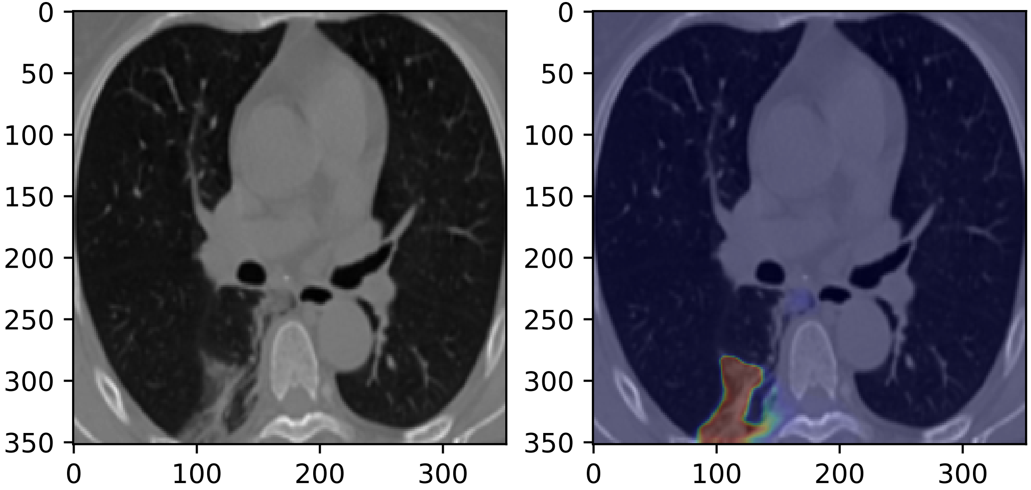

Lesion Proportion: 6.69%

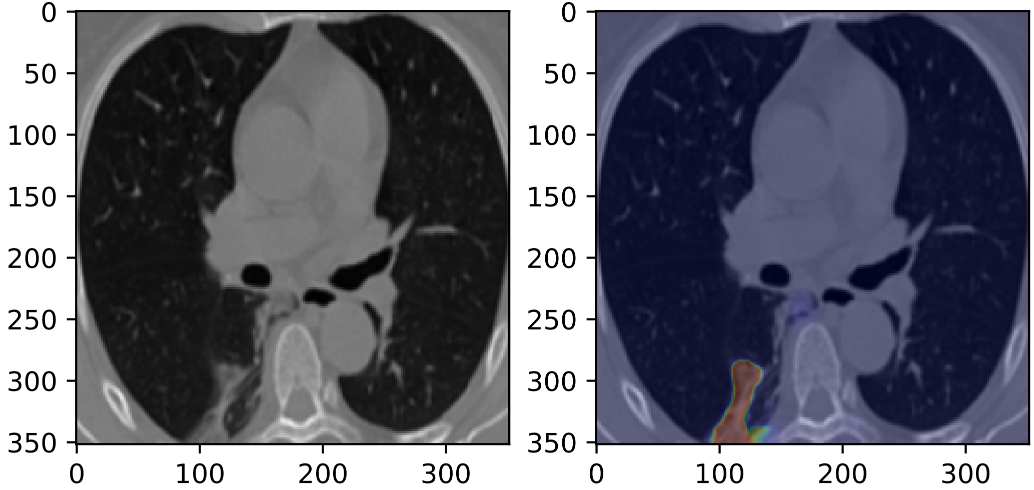

Lesion Proportion: 9.36%

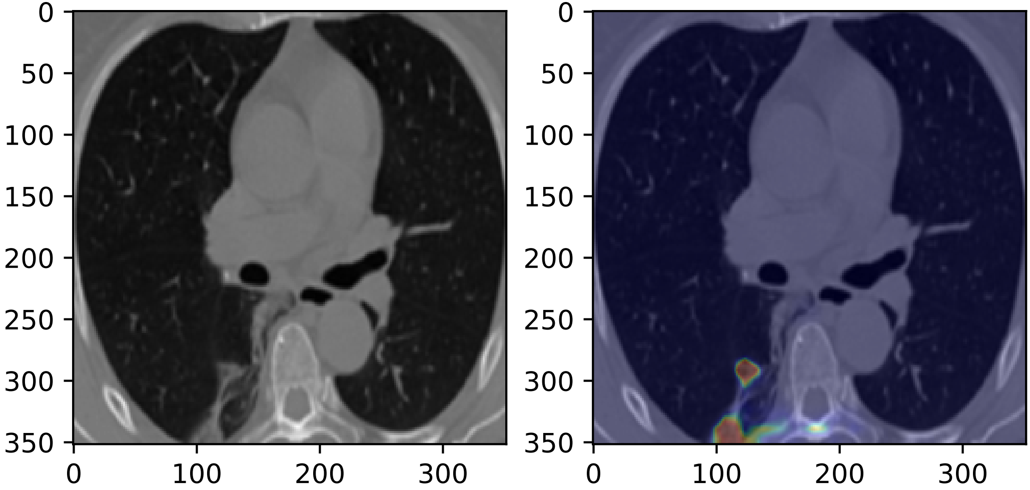

Lesion Proportion: 9.15%

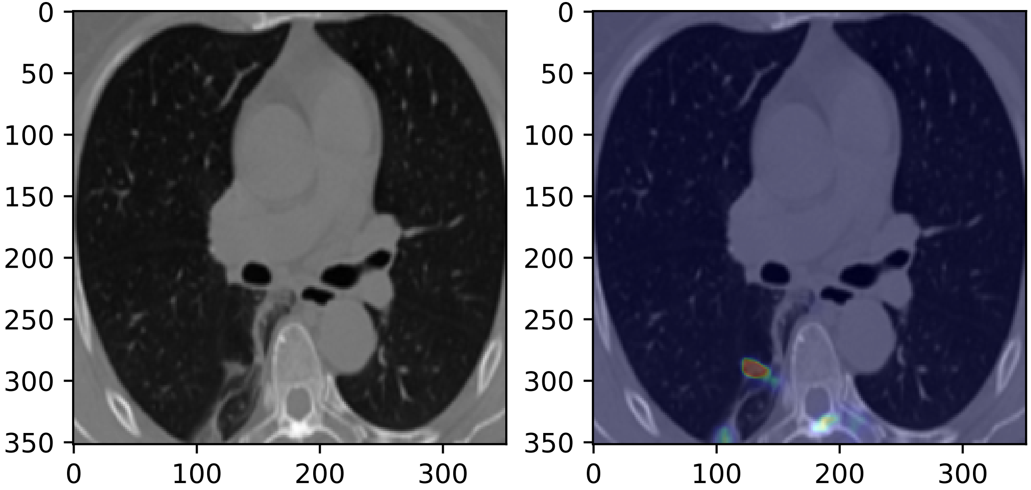

Lesion Proportion: 8.14%

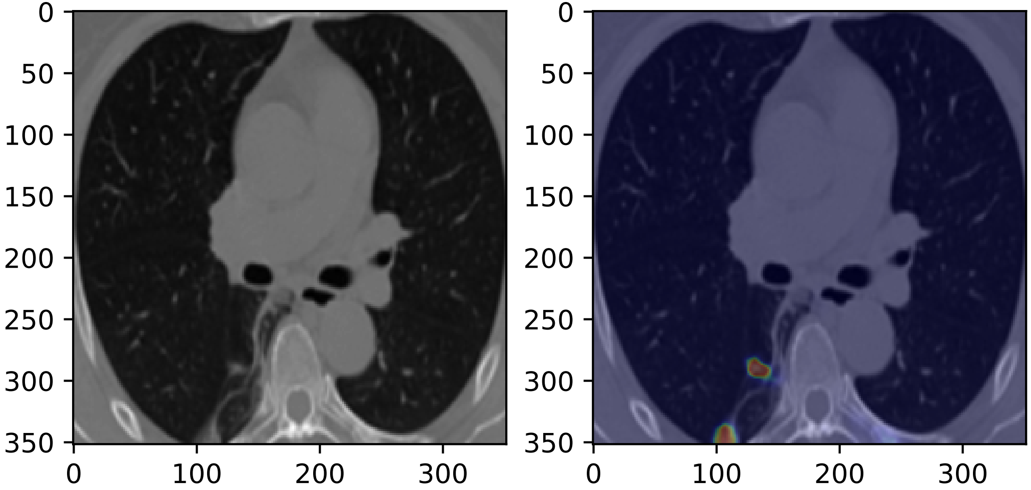

Lesion Proportion: 15.54%

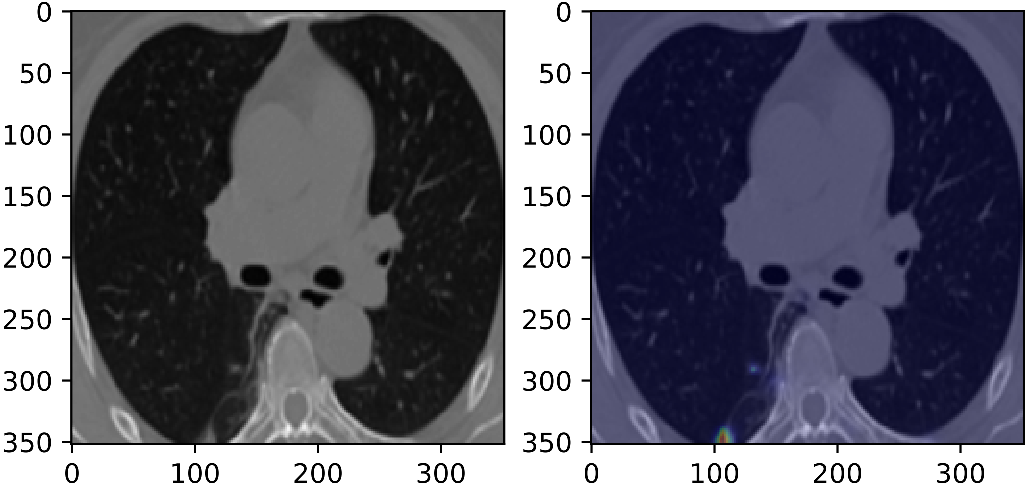

Lesion Proportion: 28.83%

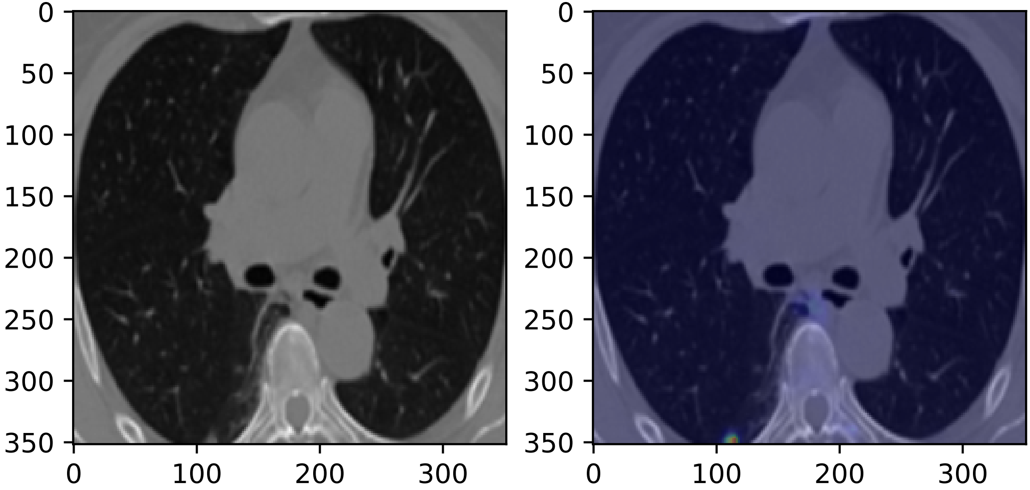

Lesion Proportion: 6.04%

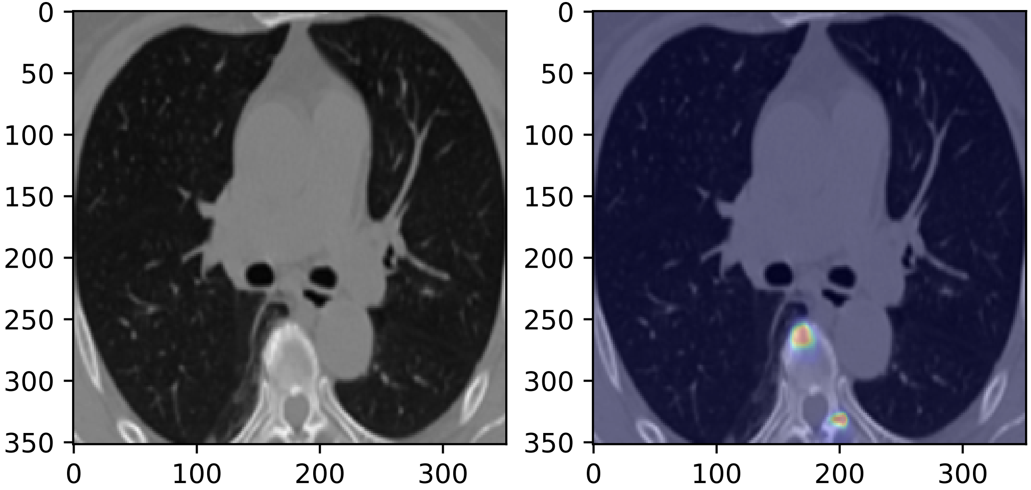

Lesion Proportion: 32.02%

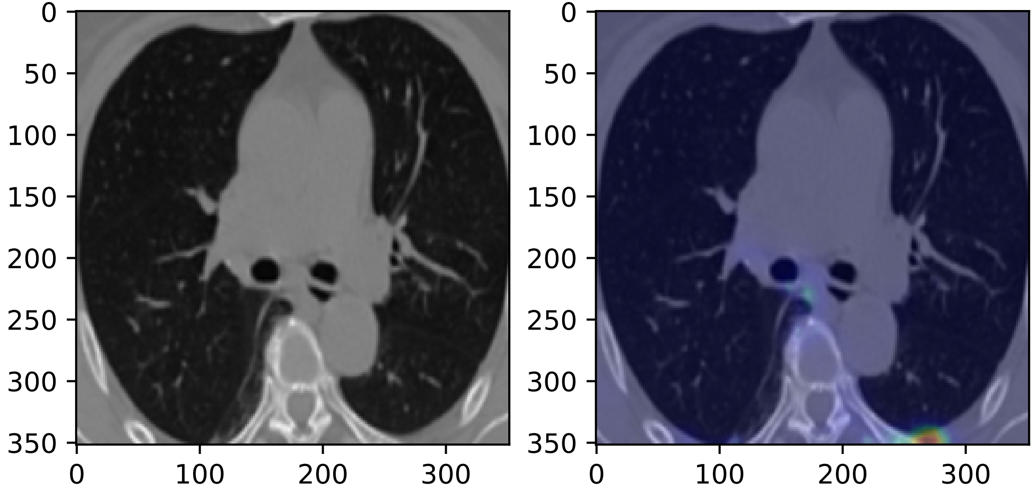

Lesion Proportion: 12.62%

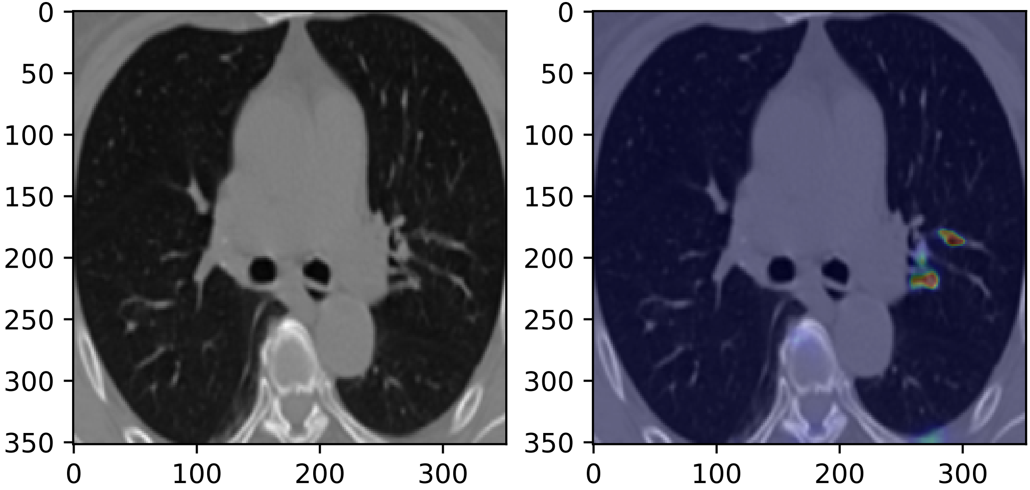

Lesion Proportion: 10.54%

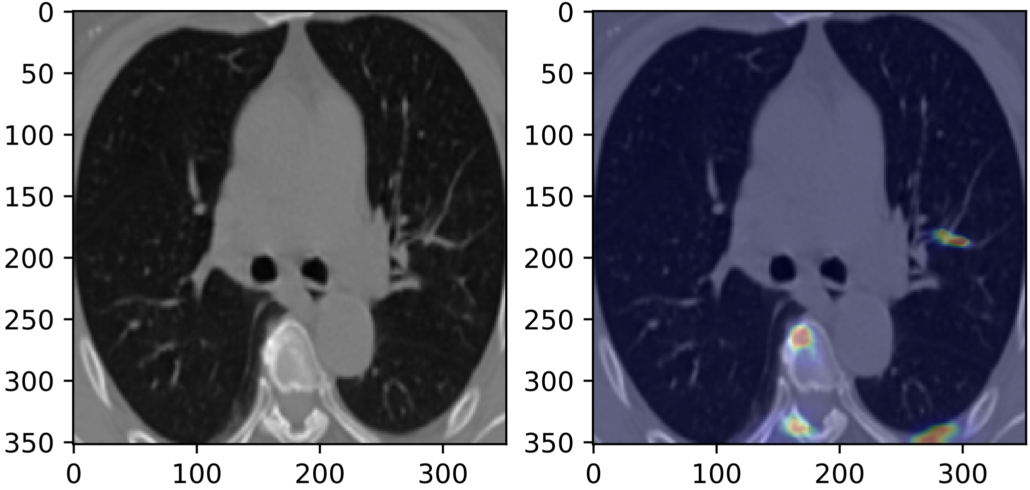

Lesion Proportion: 15.42%

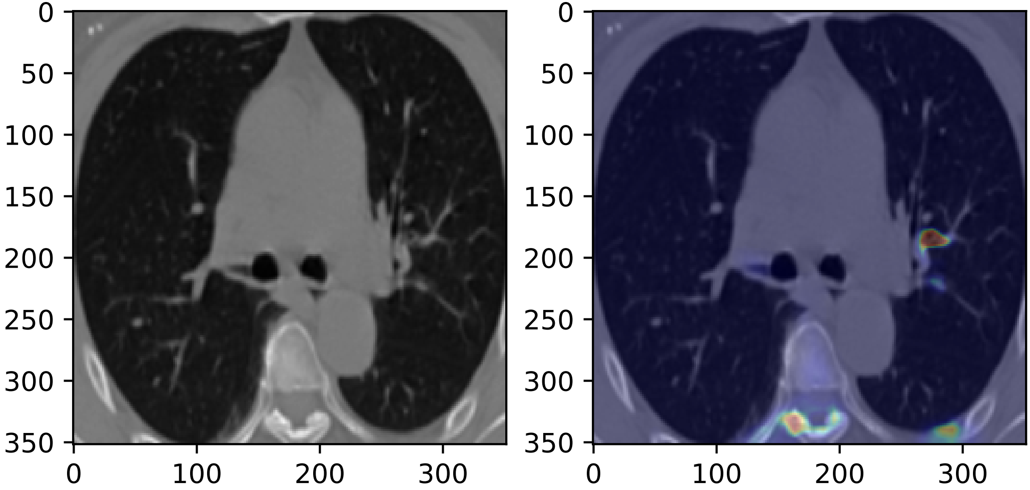

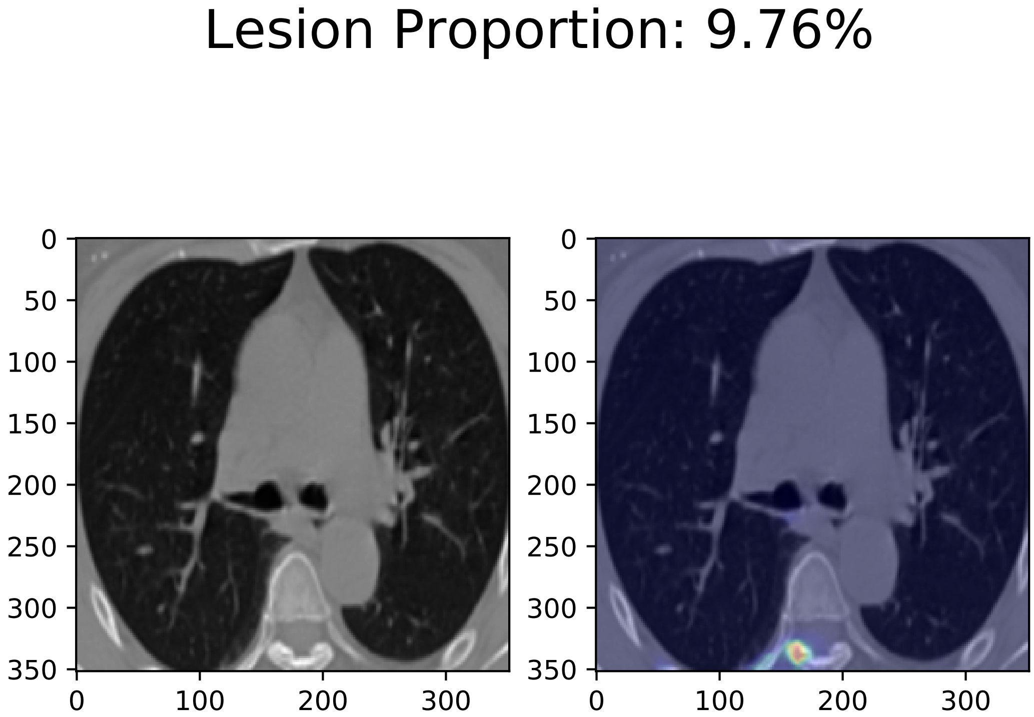
Lesion Proportion: 9.76%
0
50
100
150
200
250
300
350
0
100
200
300
0
50
100
150
200
250
300
350
0
100
200
300

Lesion Proportion: 12.31%

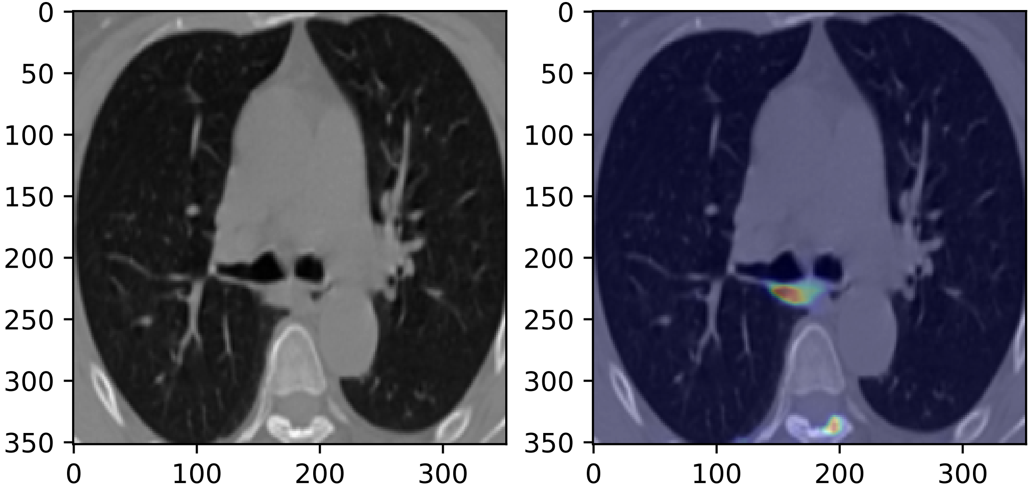

Lesion Proportion: 12.04%

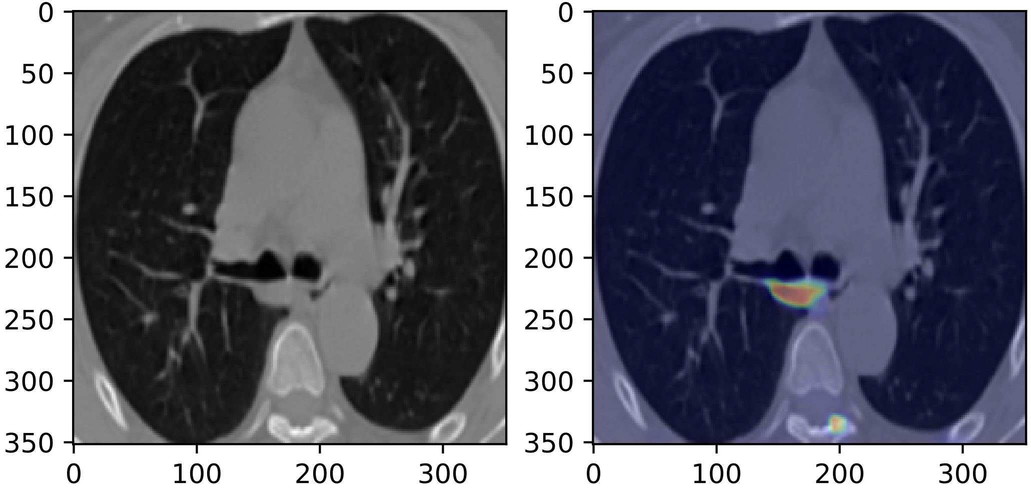

Lesion Proportion: 12.54%

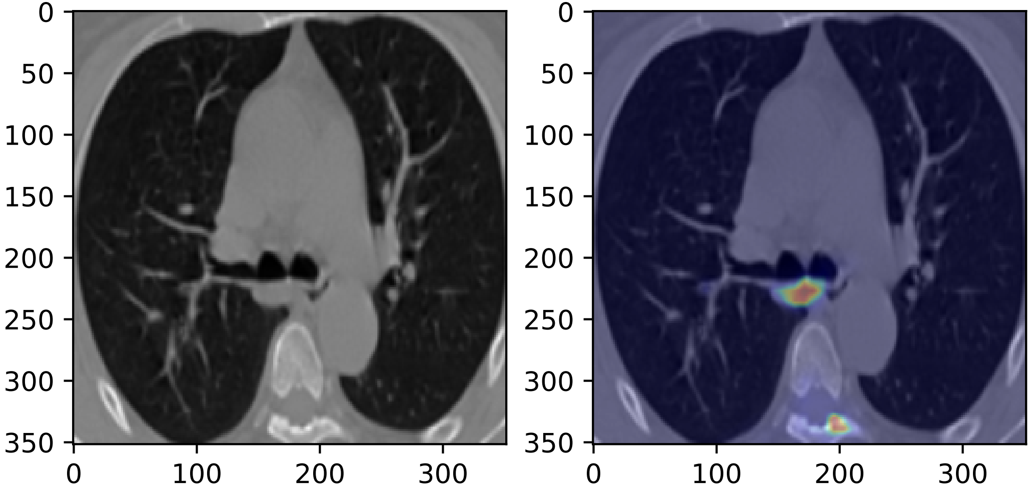

Lesion Proportion: 12.02%

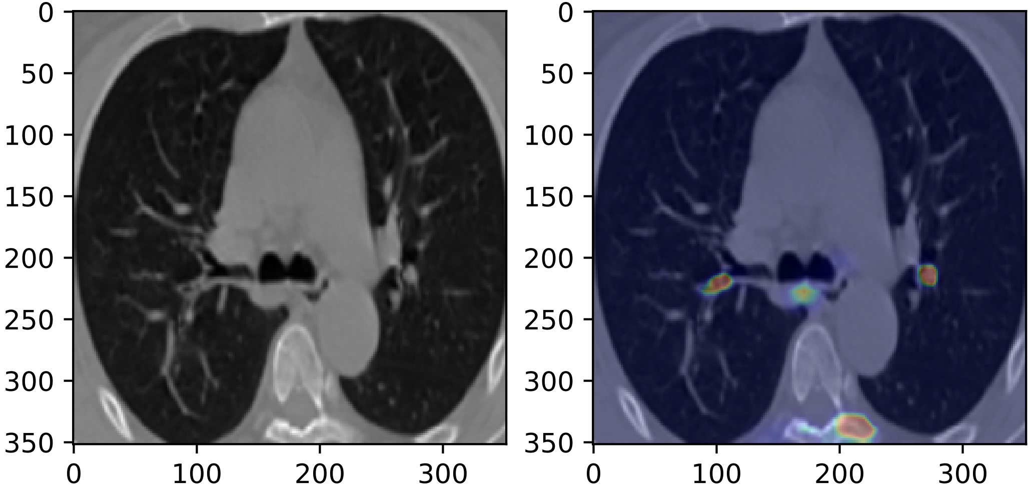

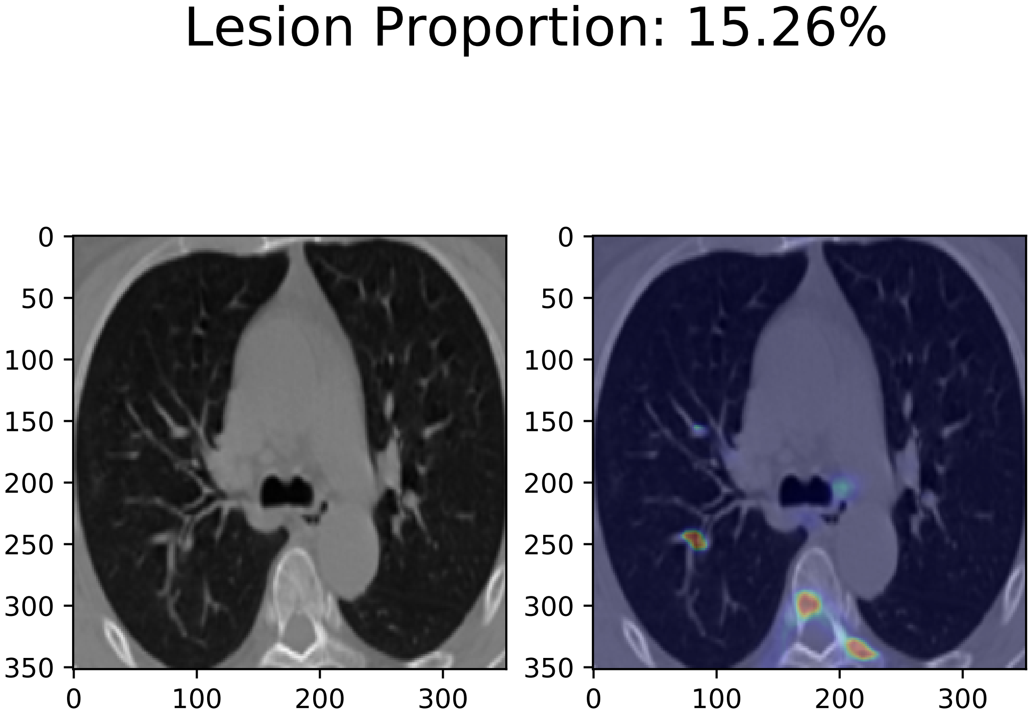
Lesion Proportion: 15.26%

Lesion Proportion: 18.03%

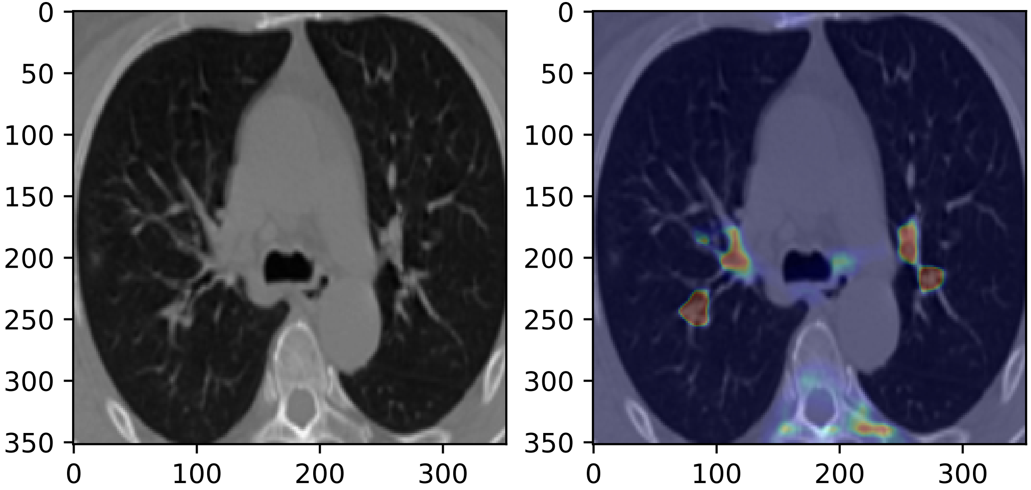

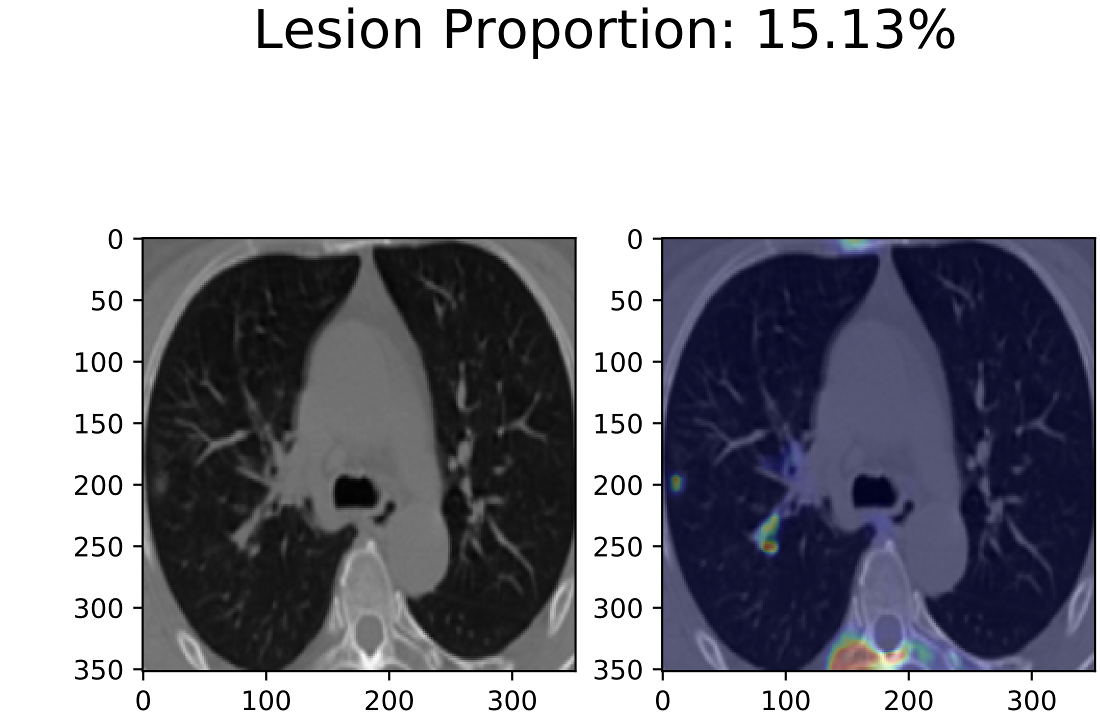

Lesion Proportion: 15.13%
0
50
100
150
200
250
300
350
0
100
200
300

Lesion Proportion: 12.84%

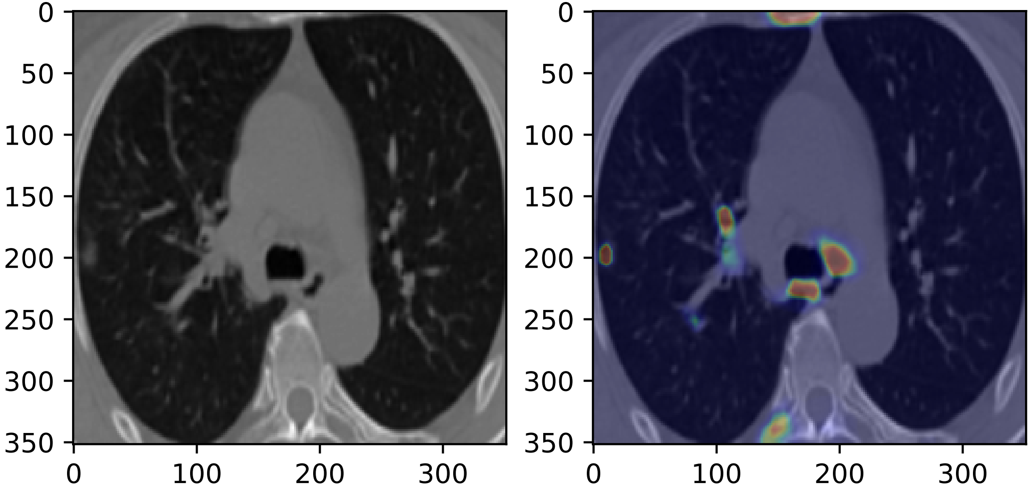

Lesion Proportion: 10.45%

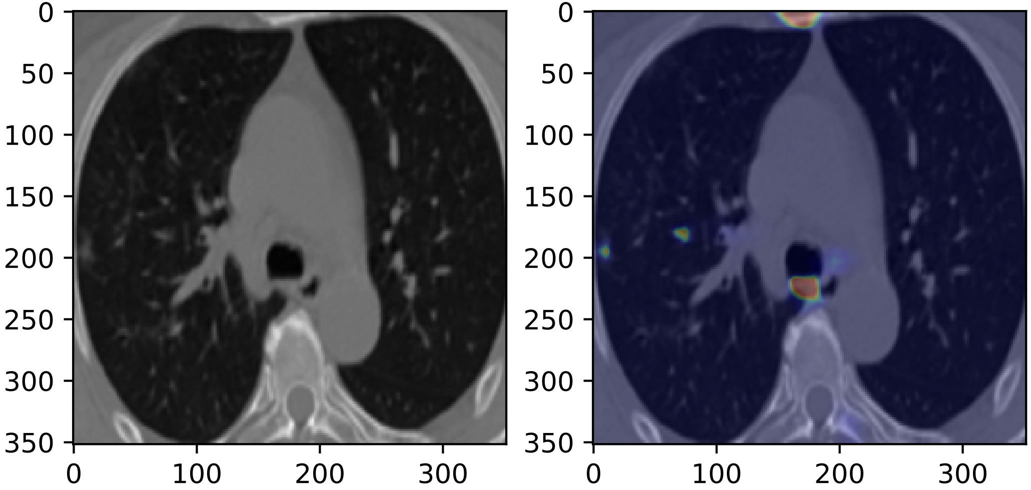

Lesion Proportion: 7.93%

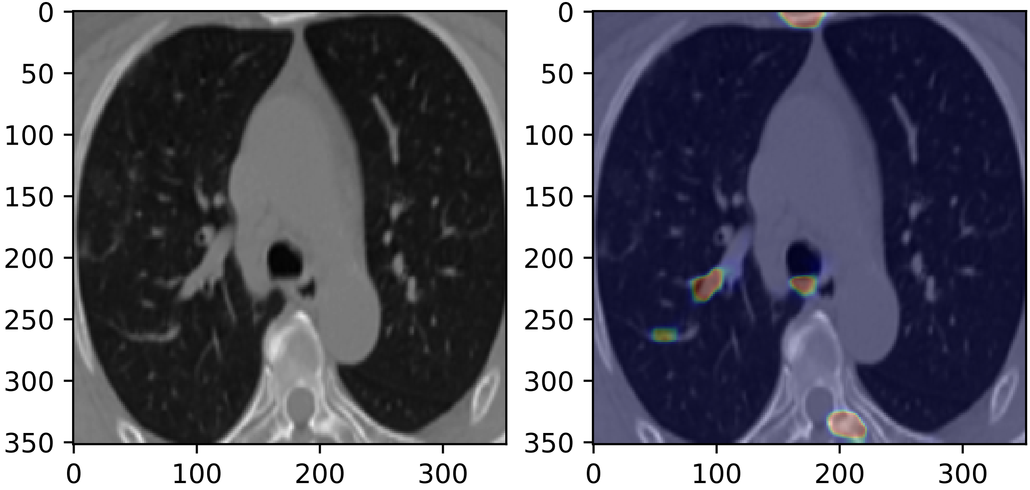

Lesion Proportion: 9.75%

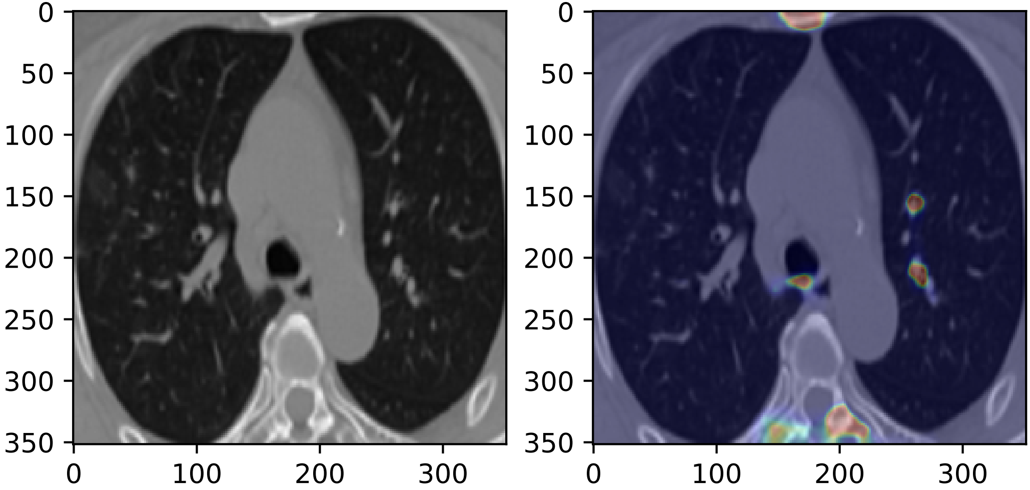

Lesion Proportion: 9.66%

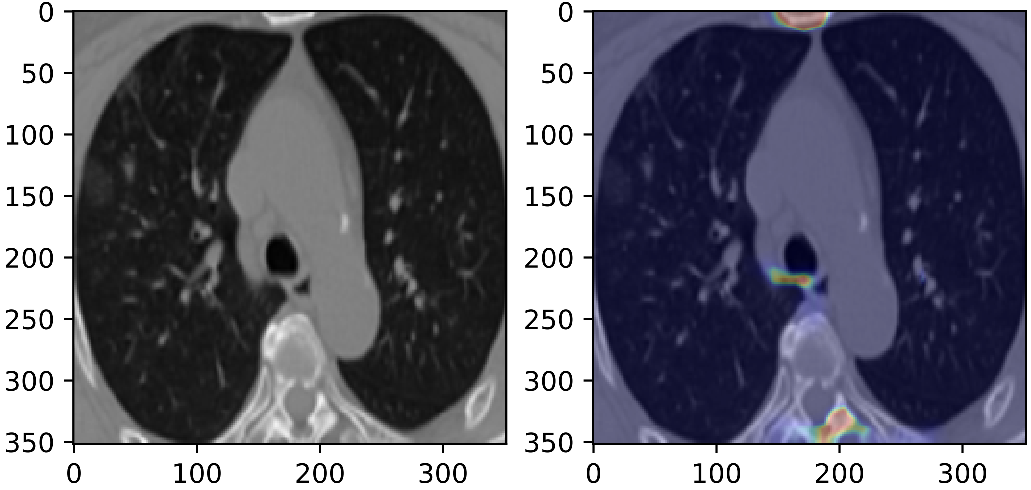

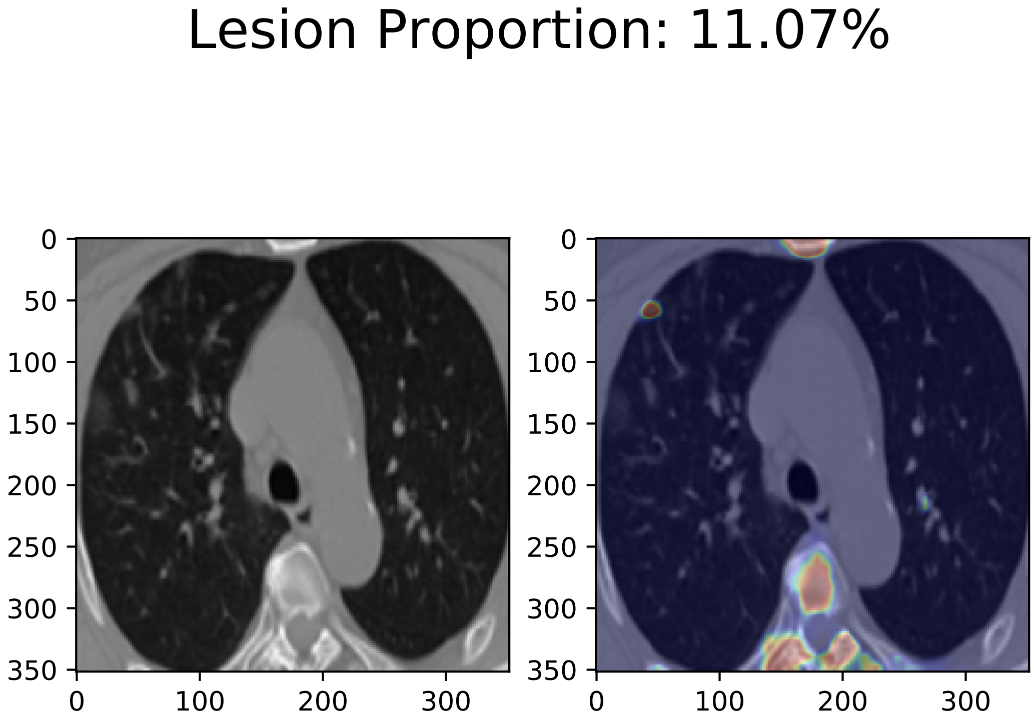
Lesion Proportion: 11.07%
0
50
100
150
200
250
300
350
0
100
200
300
0
50
100
150
200
250
300
350
0
100
200
300

Lesion Proportion: 11.35%

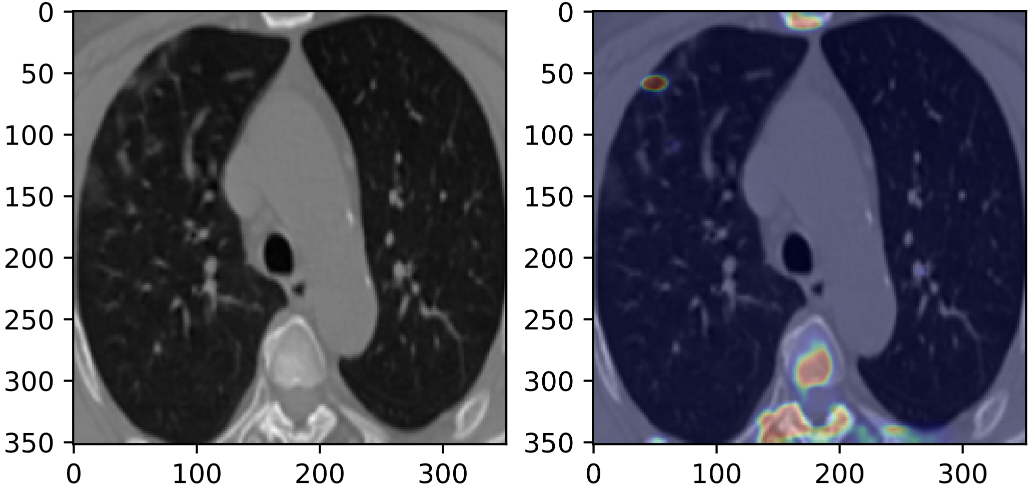

Lesion Proportion: 13.17%

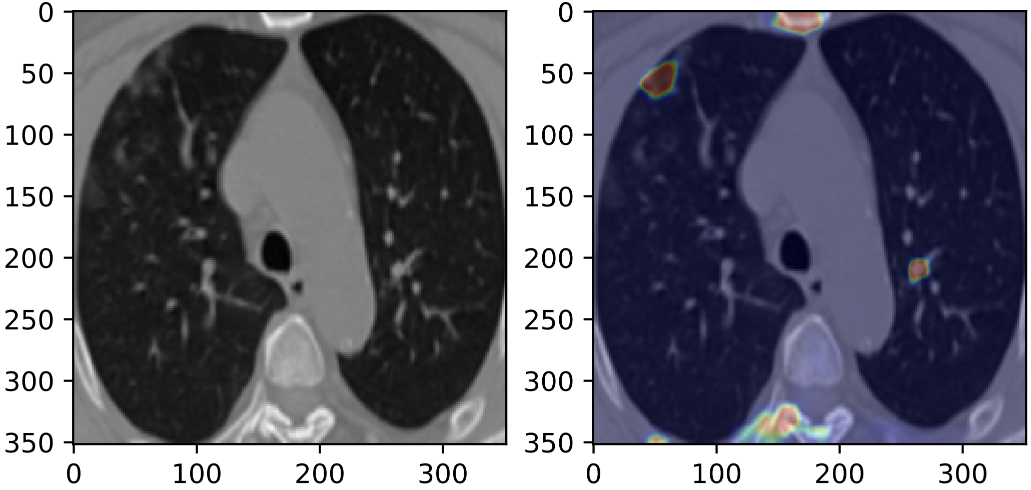

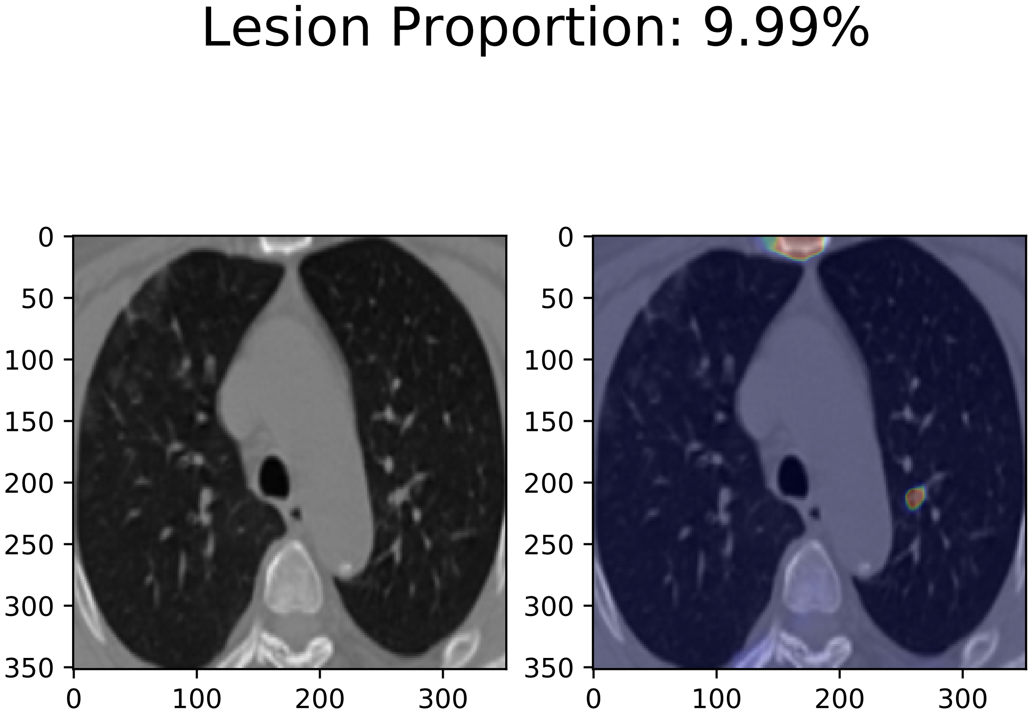
Lesion Proportion: 9.99%
0
50
100
150
200
250
300
350
0
100
200
300

Lesion Proportion: 7.66%

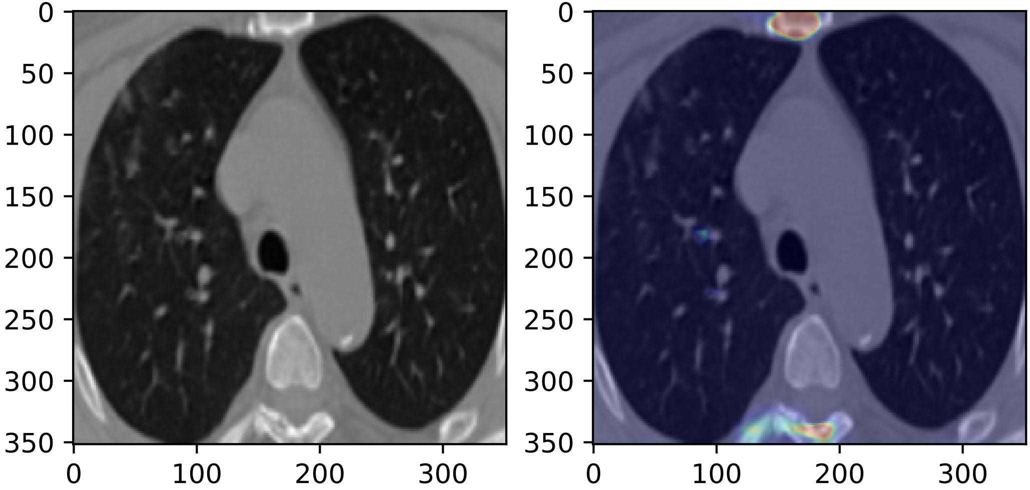

Lesion Proportion: 7.55%

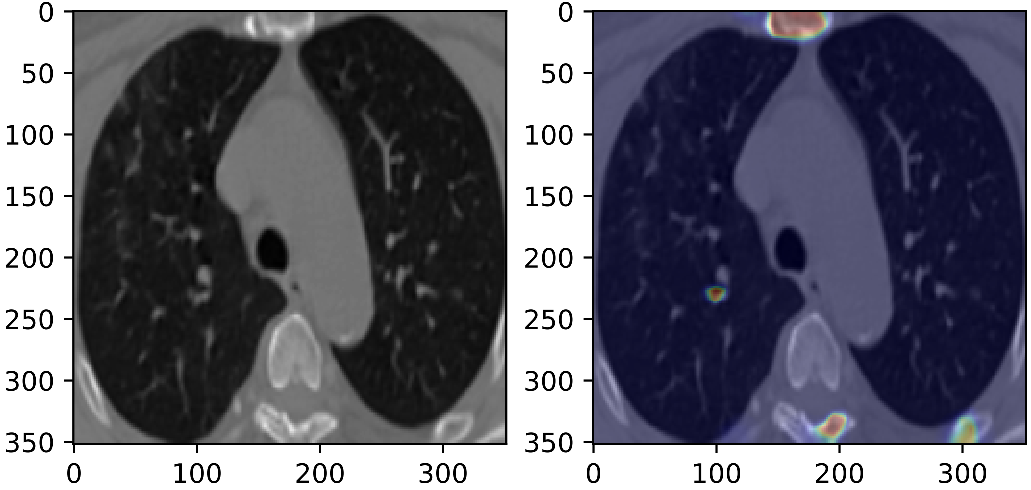

Supplement: Supplementary file 5 [file DataSheet5.pdf]
