# Supplementary material for: Medical decision support system using weakly-labeled lung CT scans
Source: Front Med Technol. 2022 Sep 28;4:980735. doi: 10.3389/fmedt.2022.980735 (PMC9554434; doi:10.3389/fmedt.2022.980735)

Lesion Proportion: 37.34%

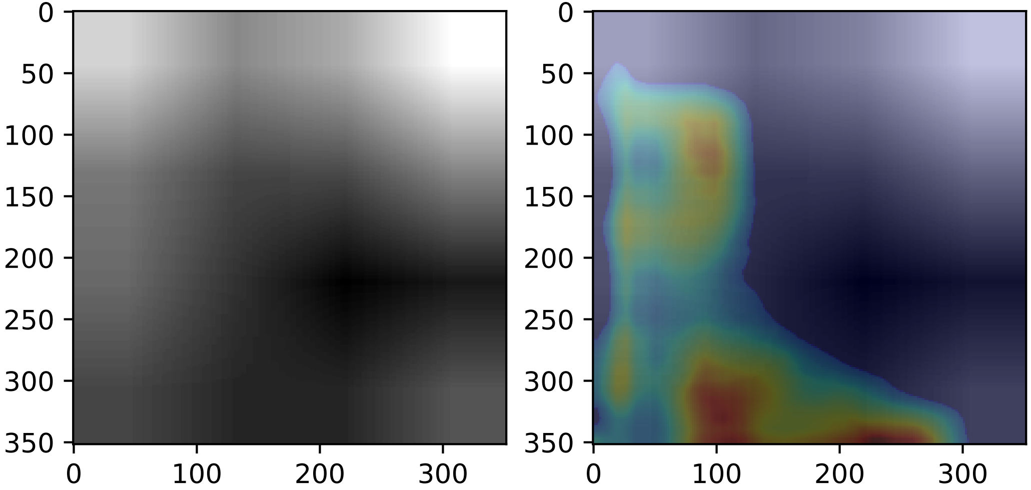

Lesion Proportion: 7.61%

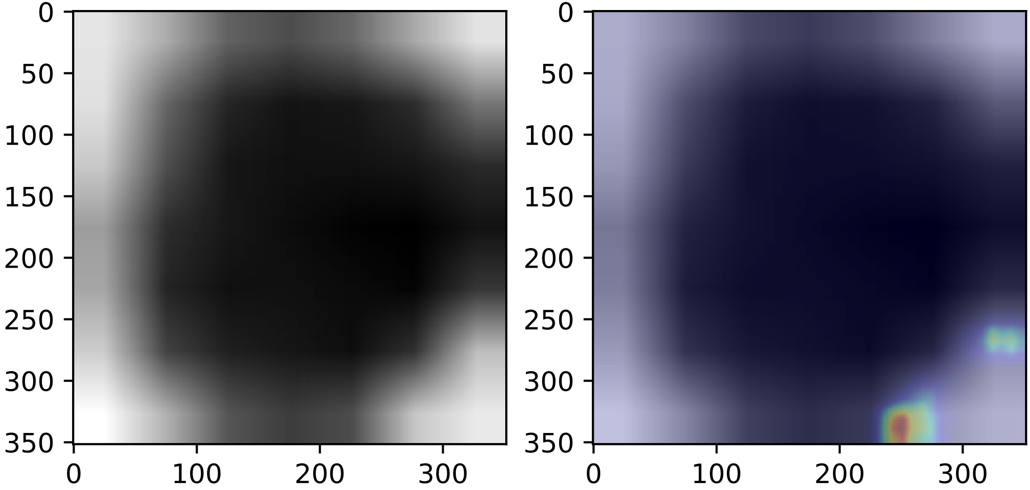

Lesion Proportion: 8.23%

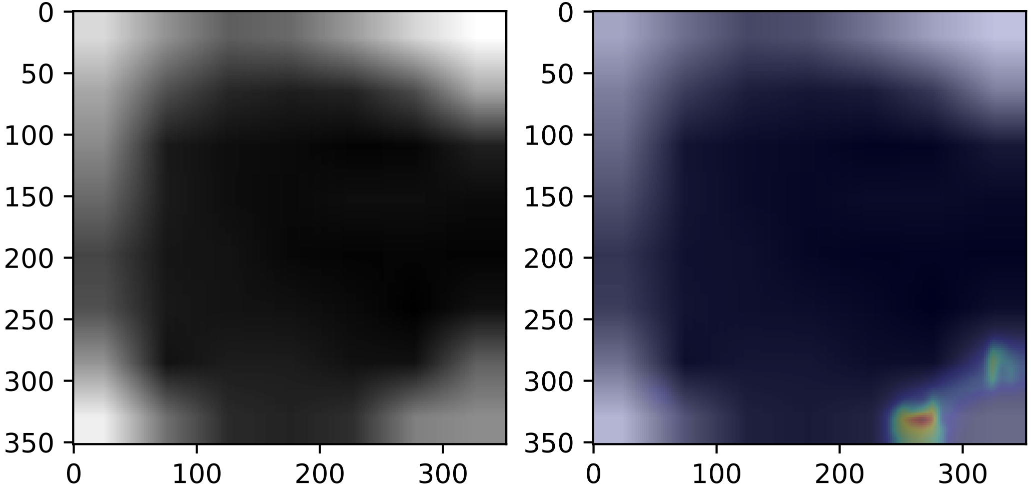

Lesion Proportion: 5.15%

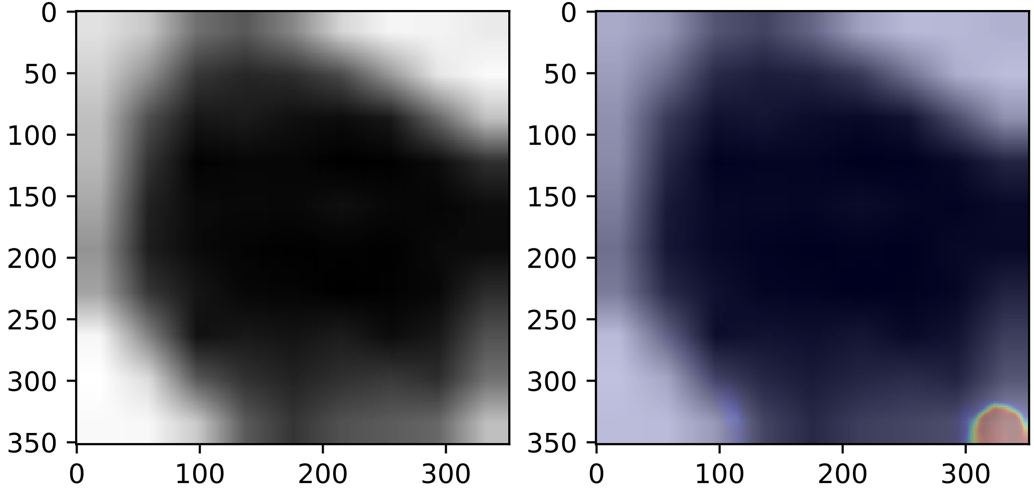

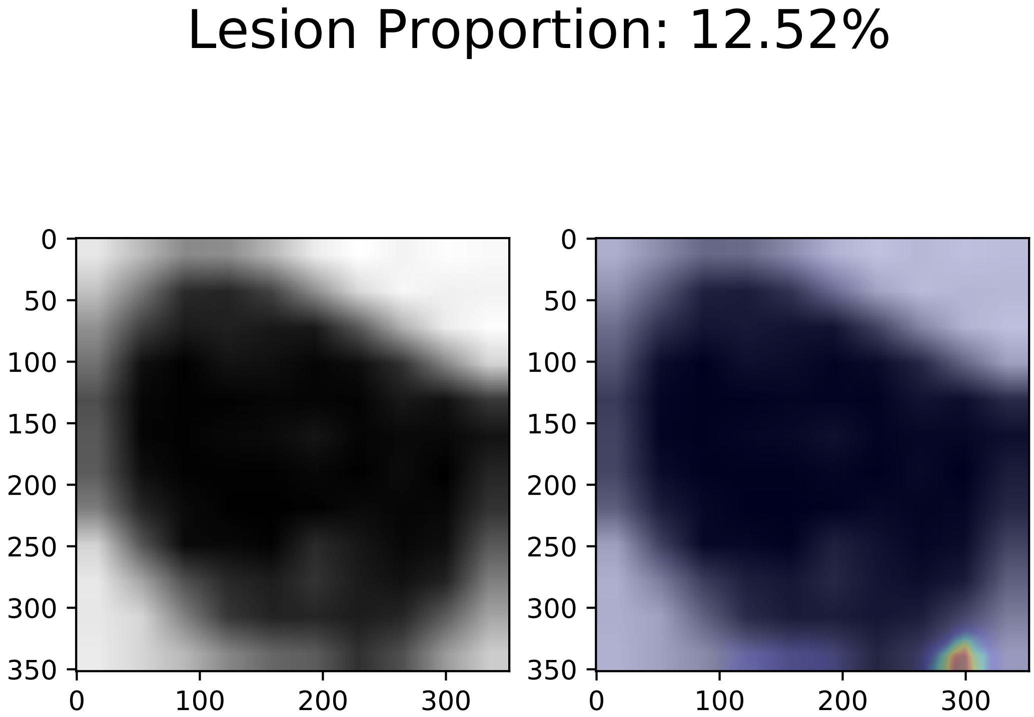
Lesion Proportion: 12.52%
0
50
100
150
200
250
300
350
0
100
200
300

Lesion Proportion: 17.41%

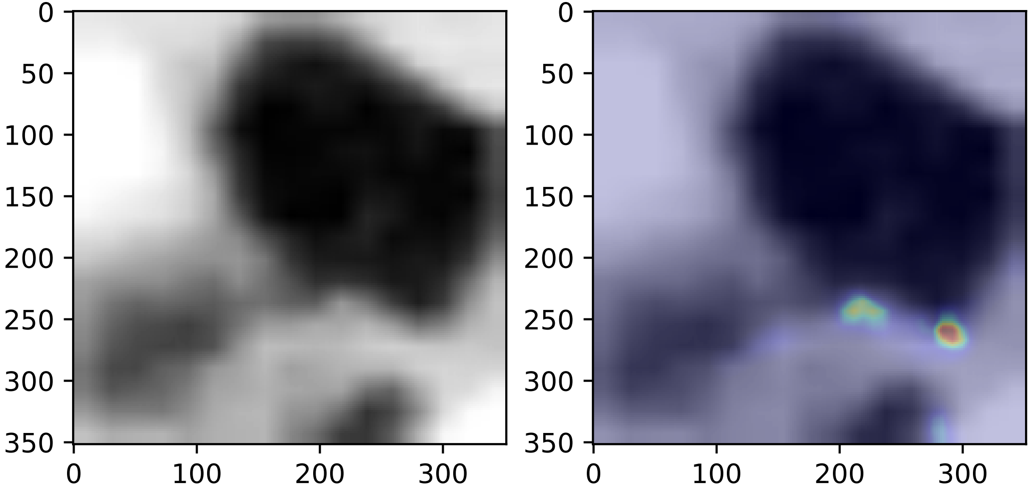

Lesion Proportion: 31.05%

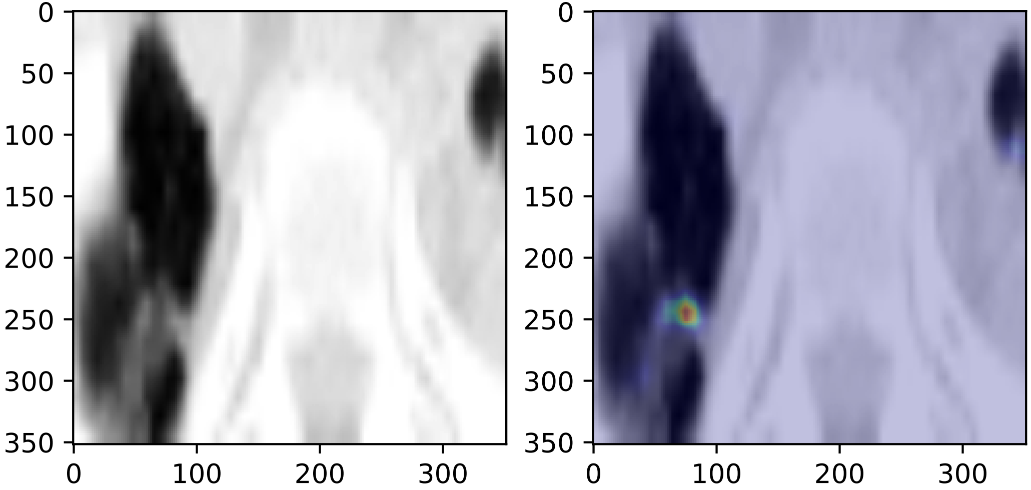

Lesion Proportion: 19.10%

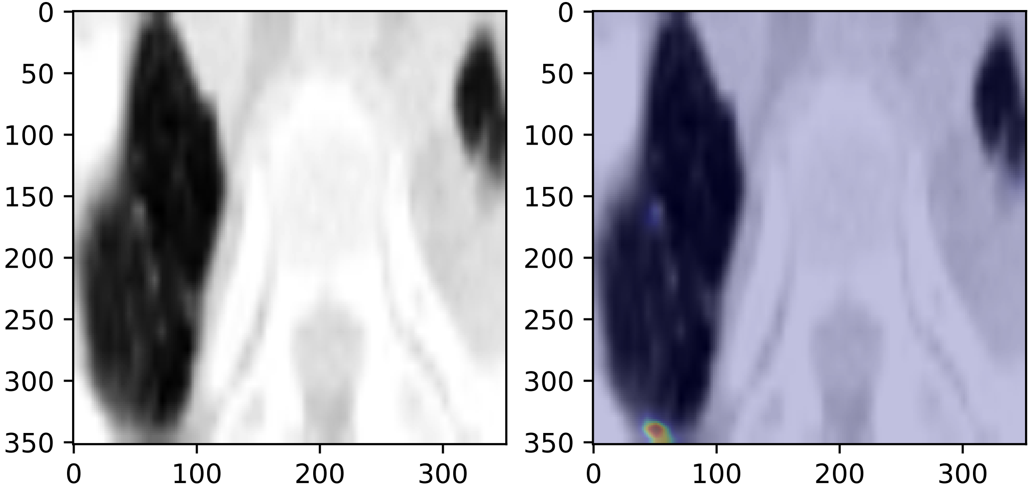

Lesion Proportion: 38.80%

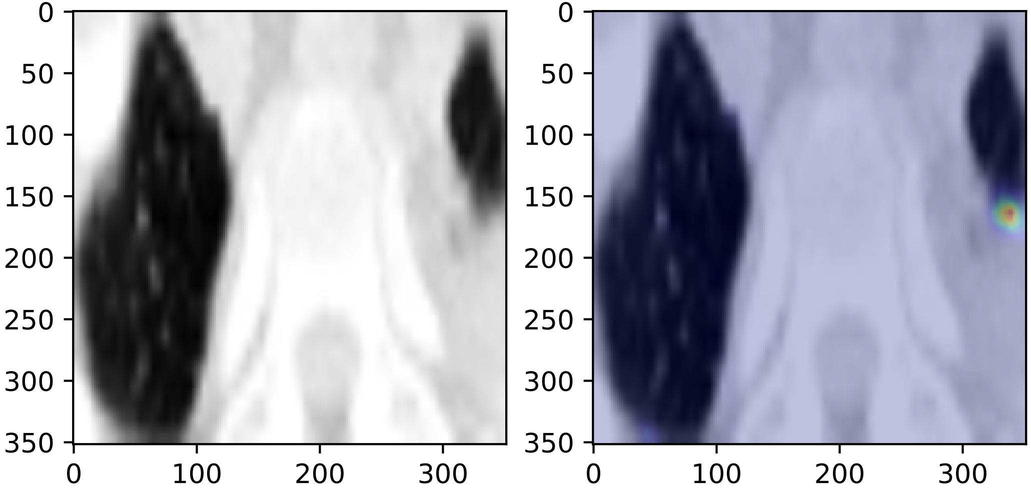

Lesion Proportion: 38.70%

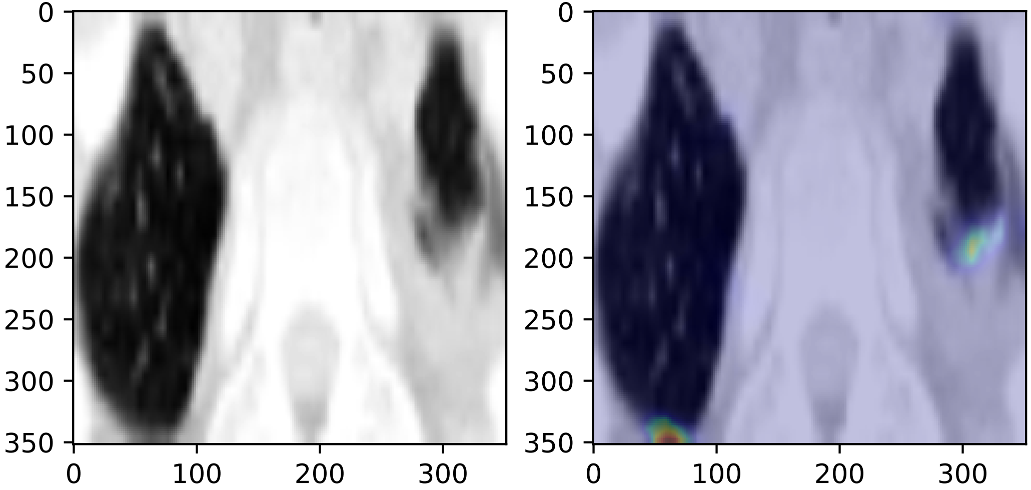

Lesion Proportion: 15.61%

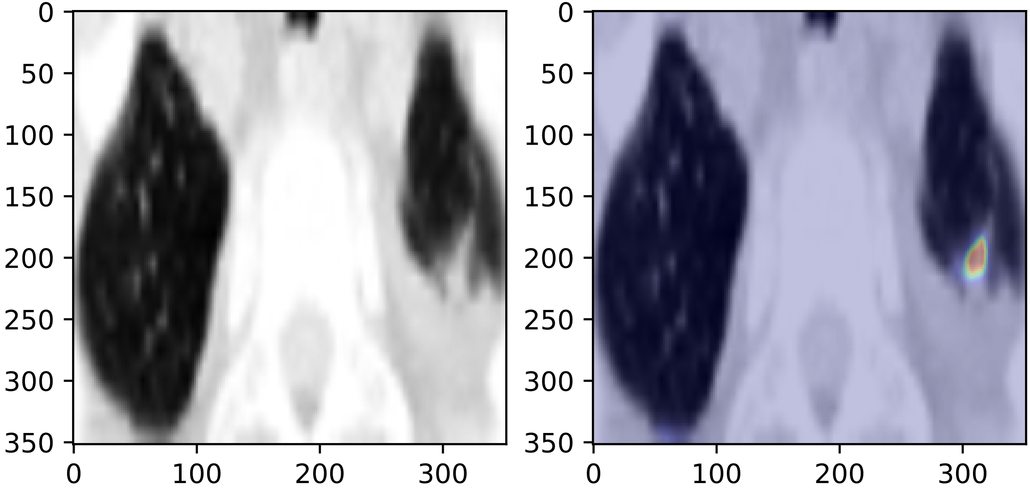

Lesion Proportion: 15.37%

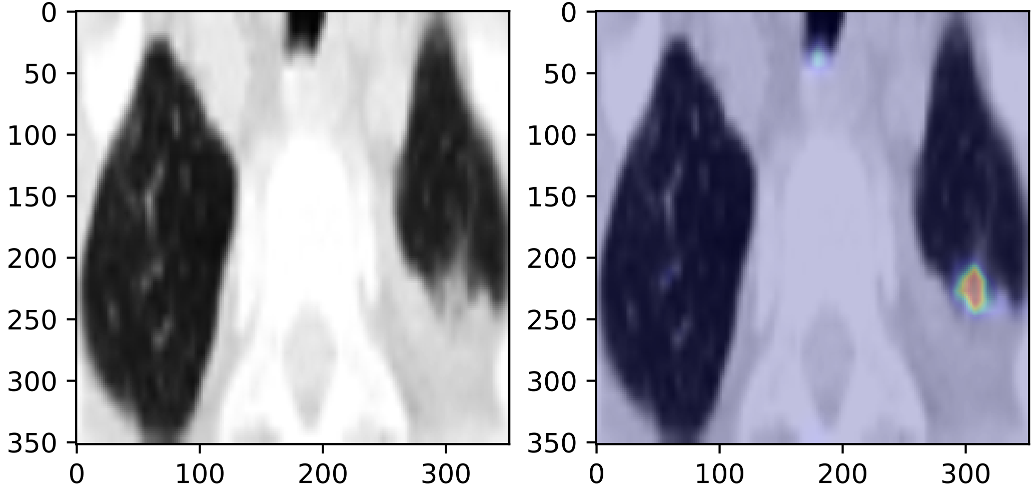

Lesion Proportion: 13.82%

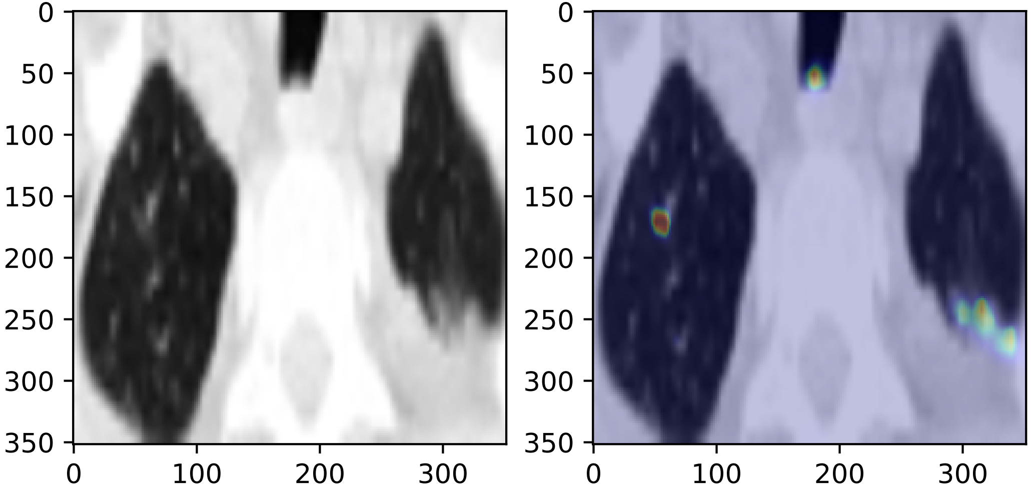

Lesion Proportion: 42.00%

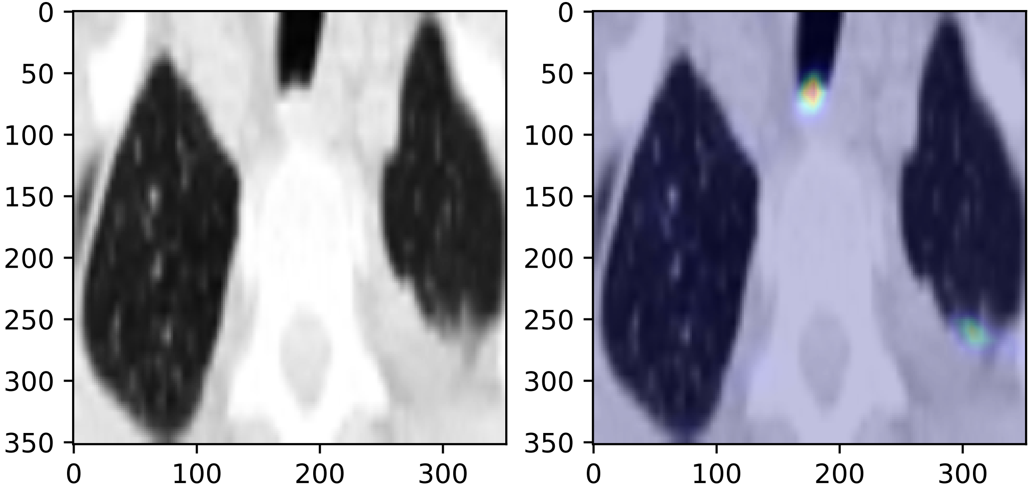

Lesion Proportion: 41.47%

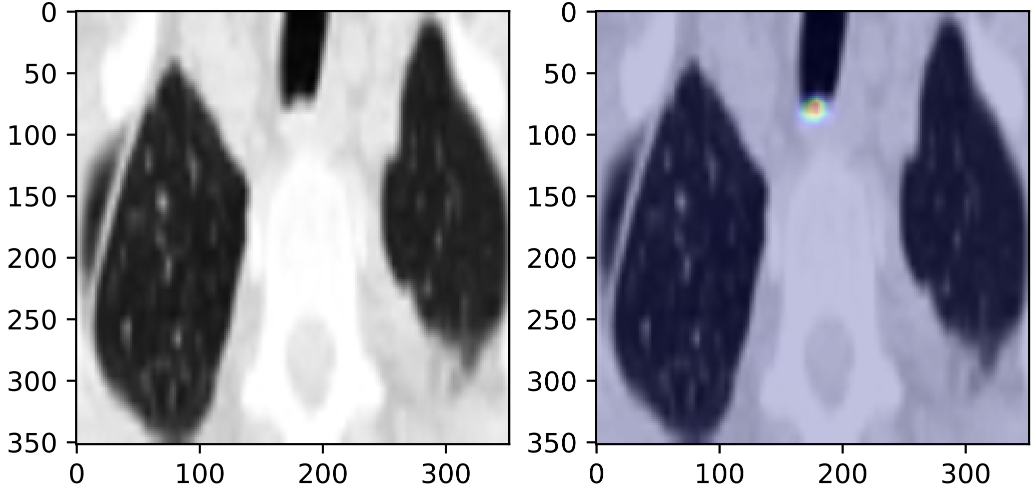

Lesion Proportion: 20.56%

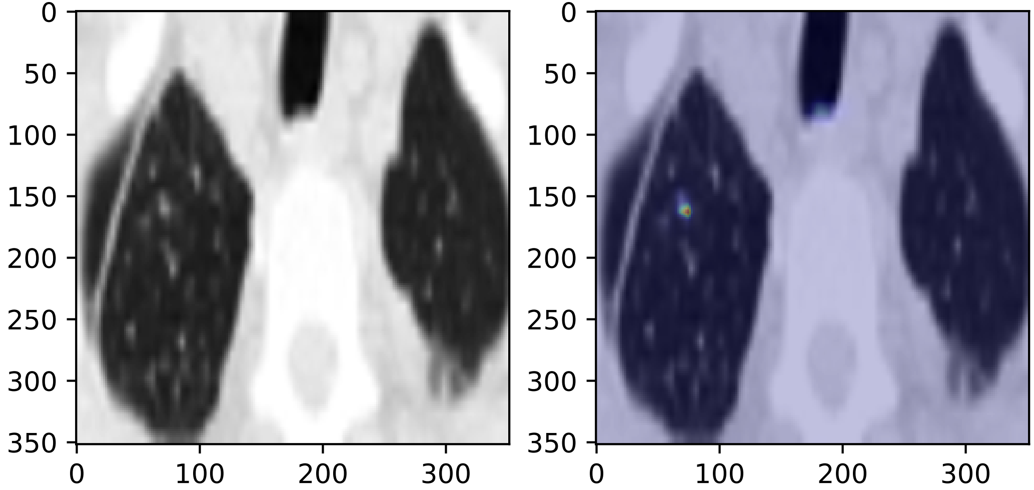

Lesion Proportion: 38.93%

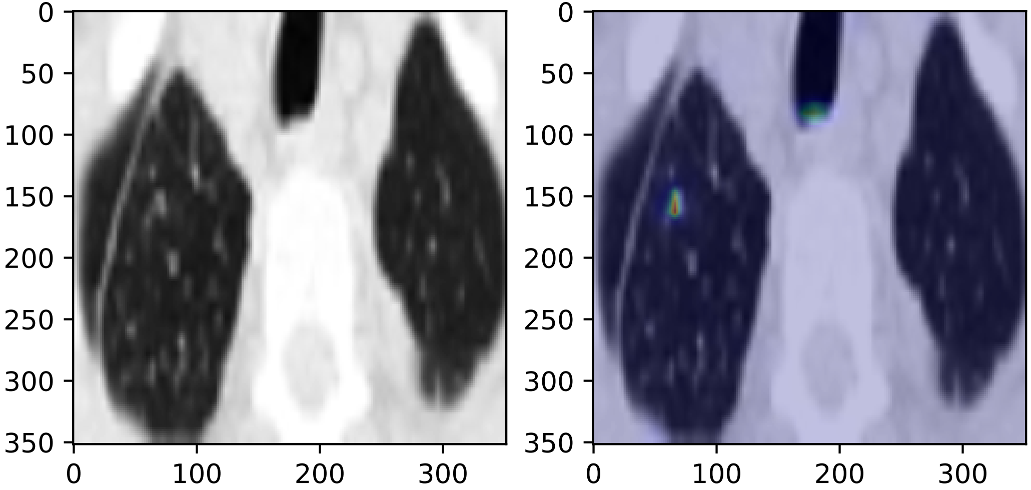

Lesion Proportion: 6.71%

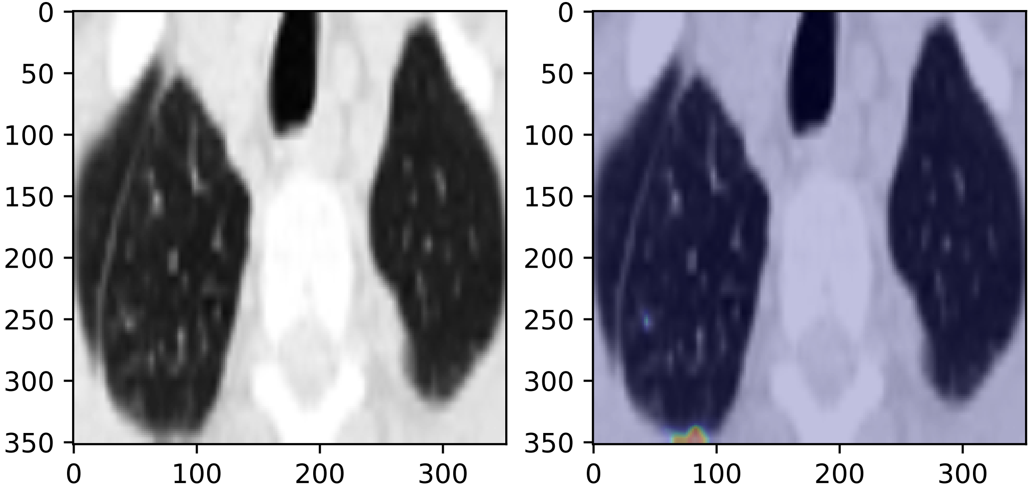

Lesion Proportion: 7.80%

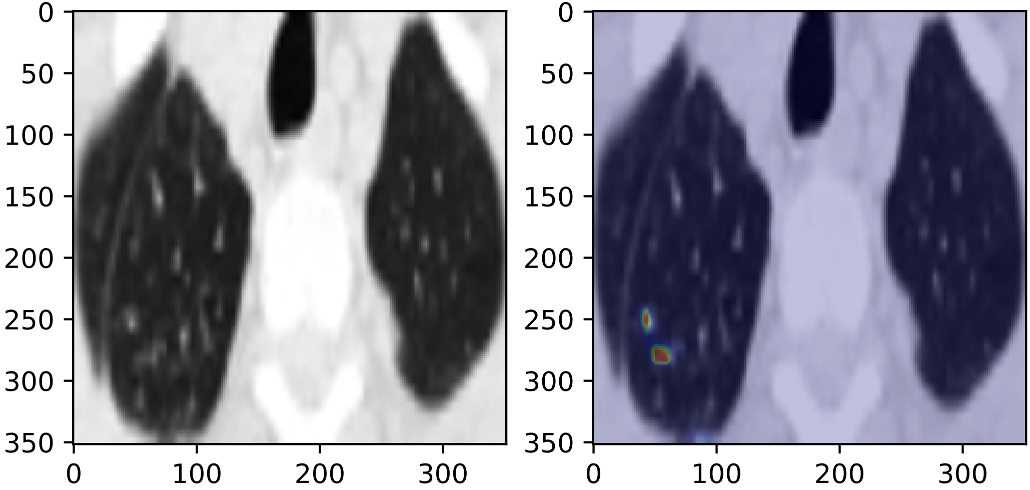

Lesion Proportion: 5.00%

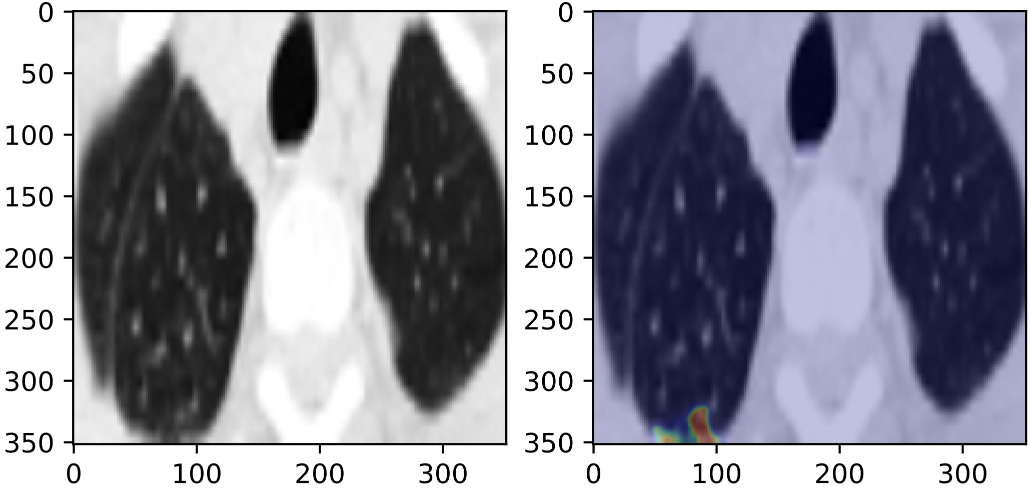

Lesion Proportion: 6.04%

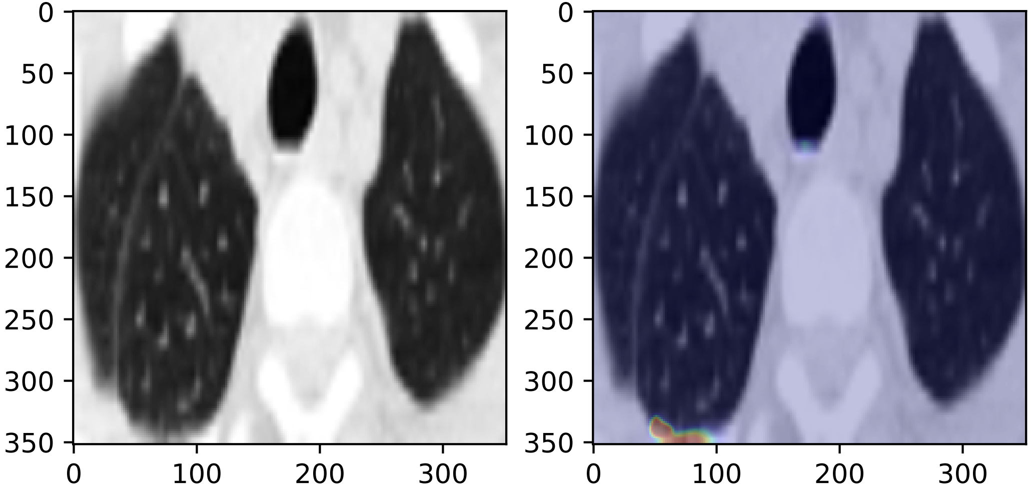

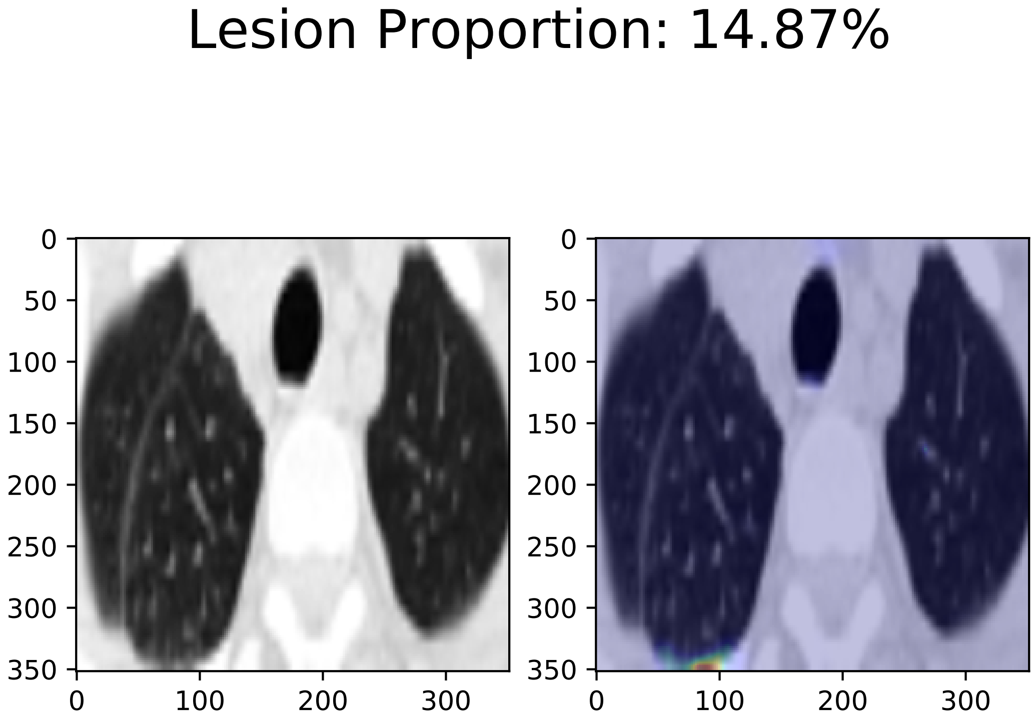

Lesion Proportion: 14.87%
0
50
100
150
200
250
300
350
0
100
200
300

Lesion Proportion: 8.10%

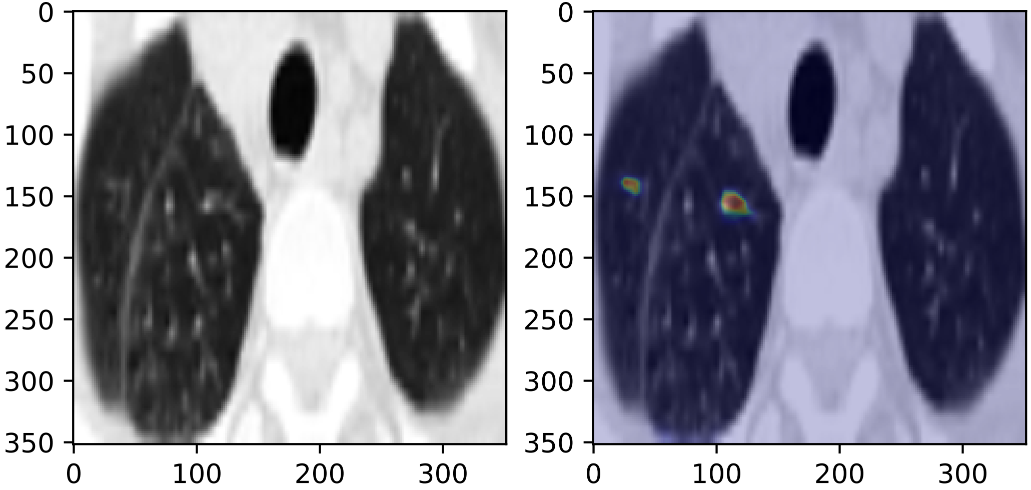

Lesion Proportion: 19.81%

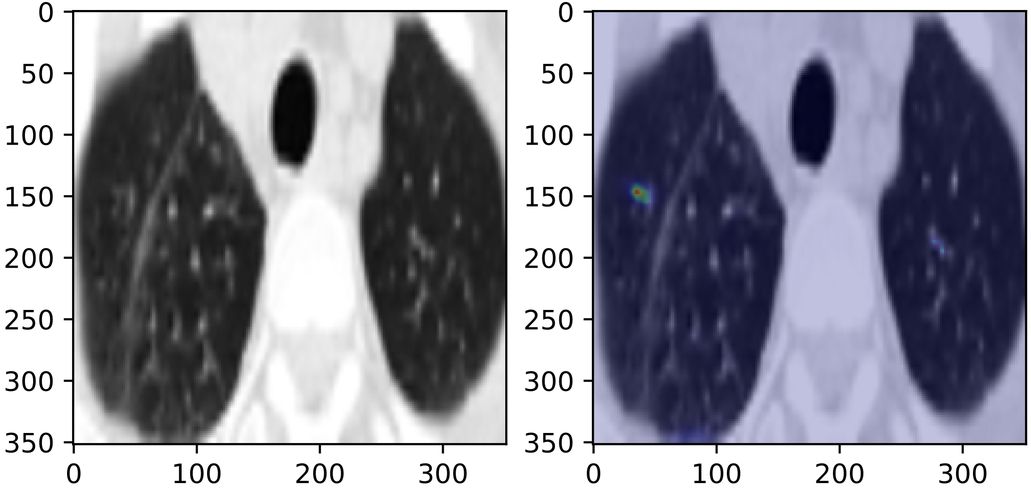

Lesion Proportion: 6.84%

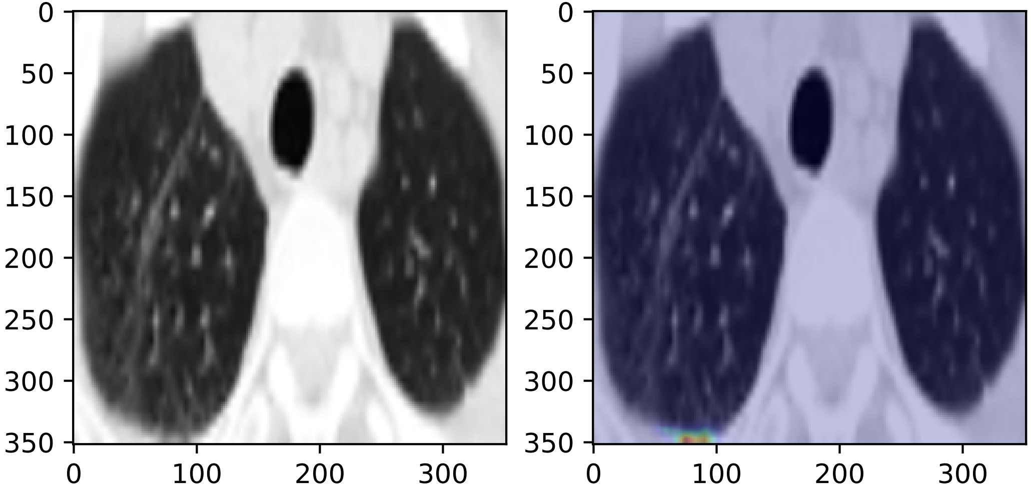

Lesion Proportion: 3.53%

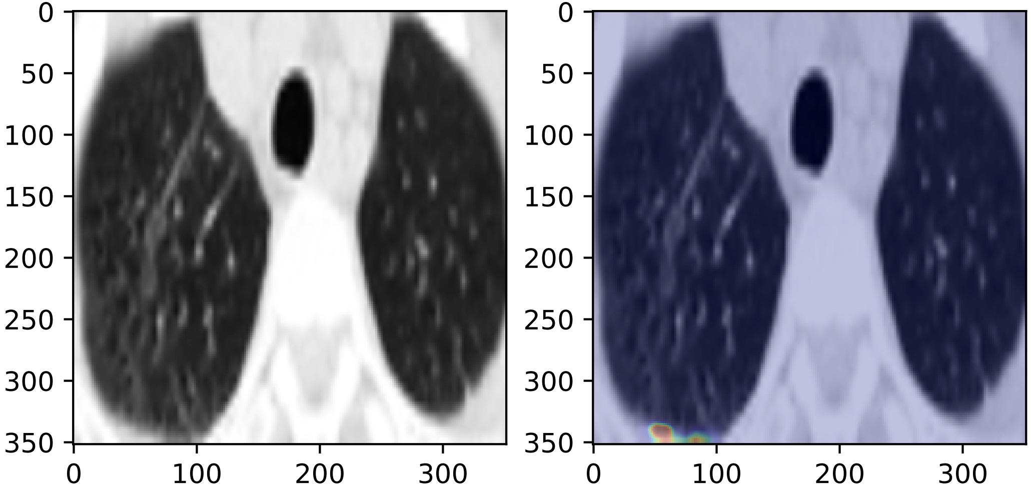

Lesion Proportion: 4.74%

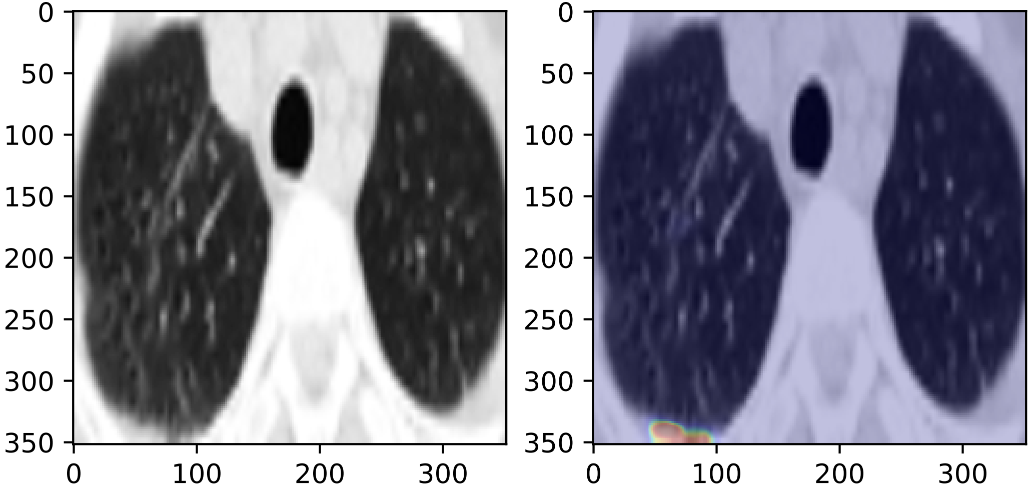

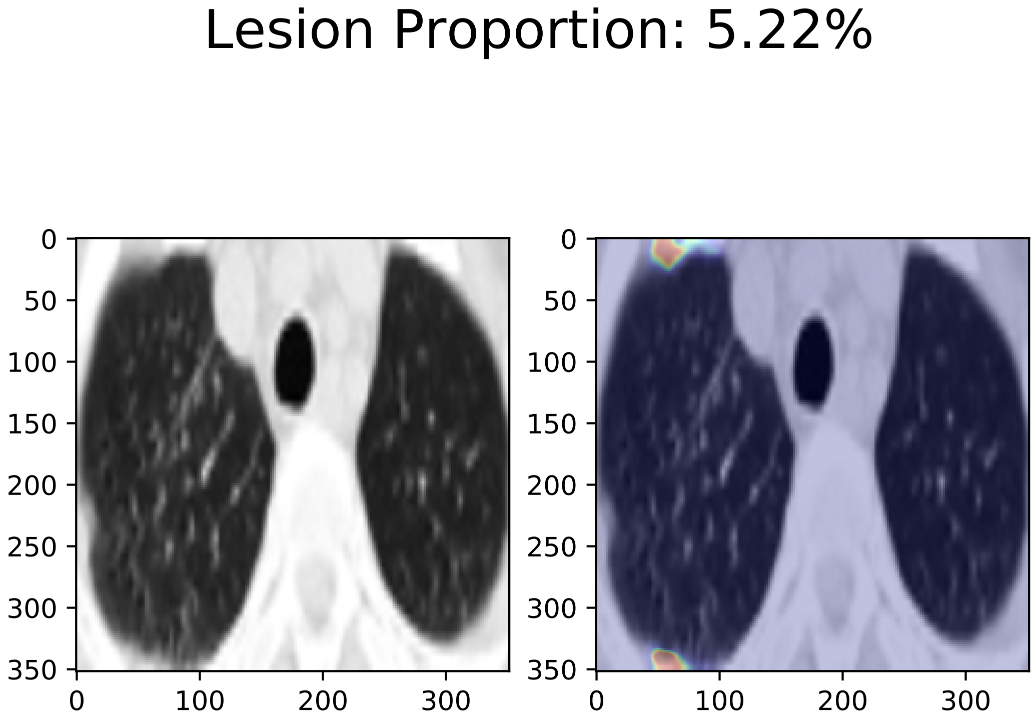
Lesion Proportion: 5.22%
0
50
100
150
200
250
300
350
0
100
200
300

Lesion Proportion: 5.13%

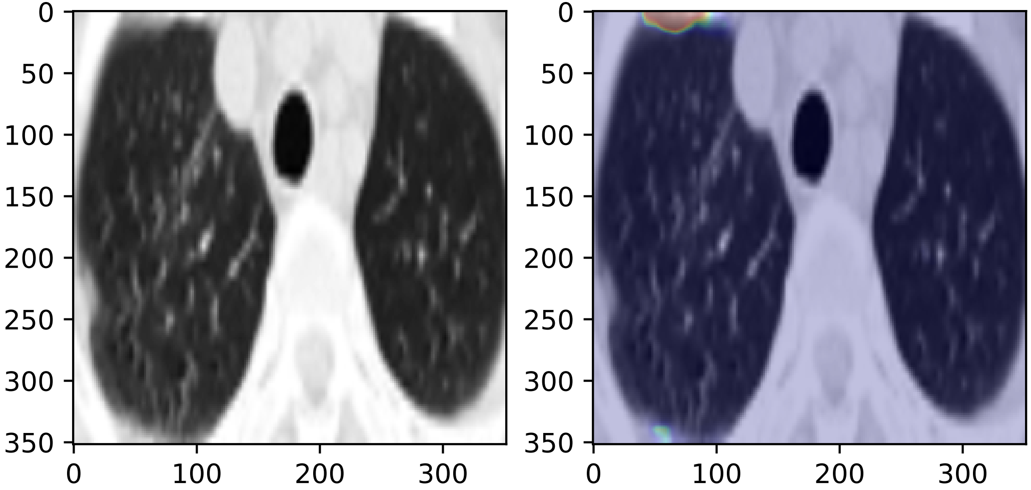

Lesion Proportion: 4.13%

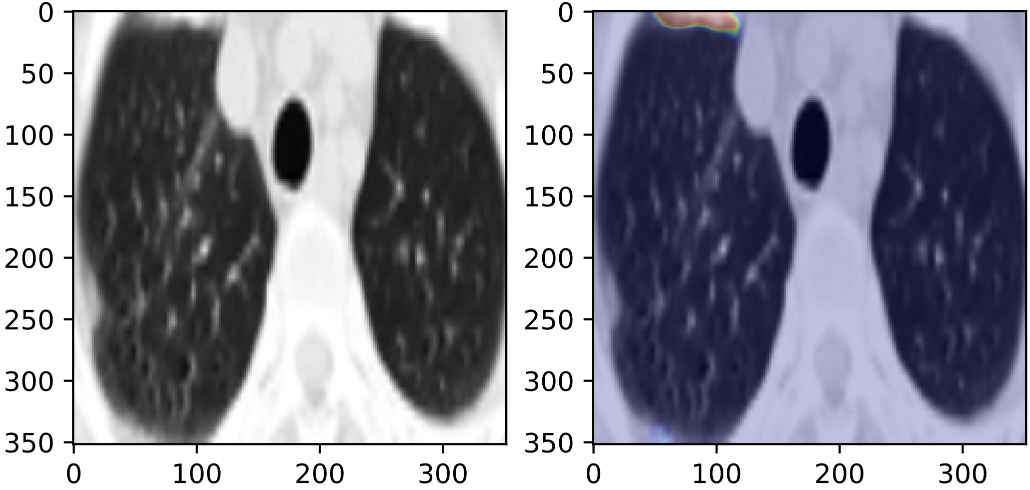

Lesion Proportion: 4.89%

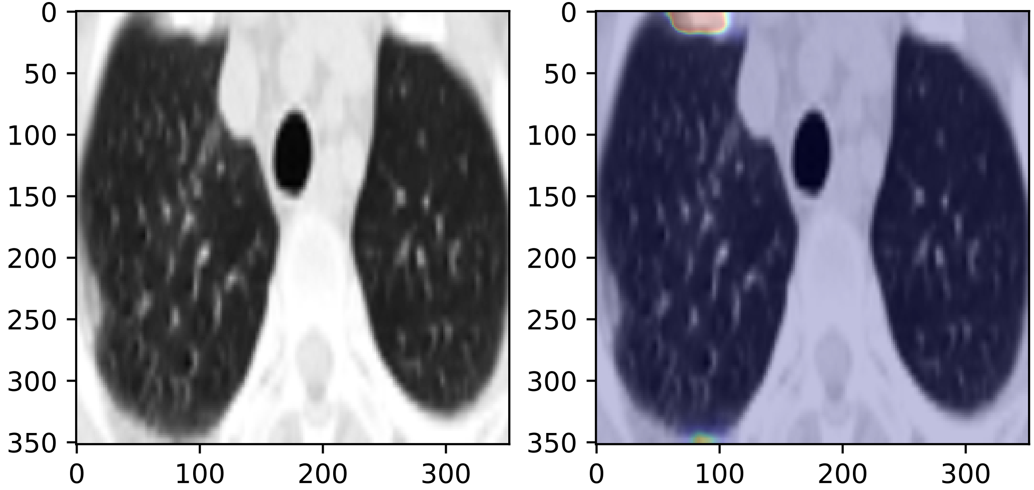

Lesion Proportion: 10.41%

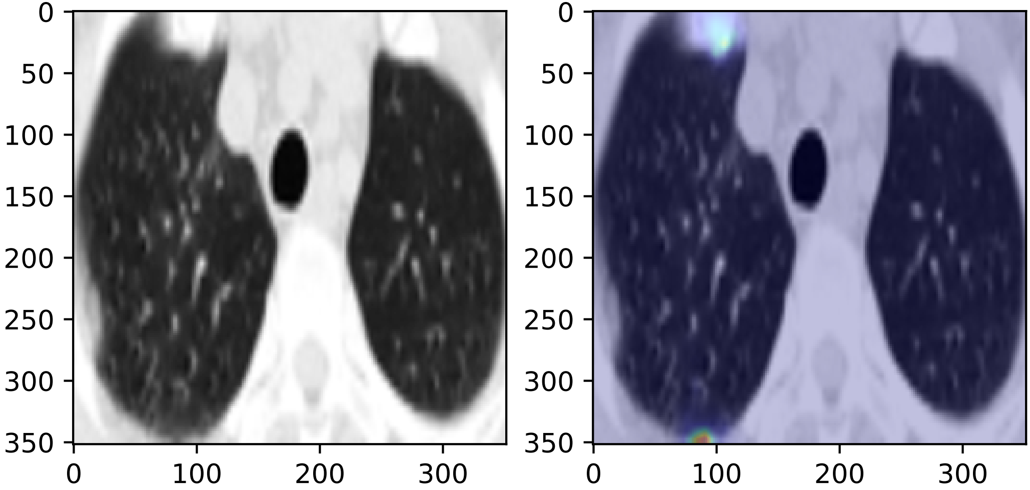

Lesion Proportion: 7.93%

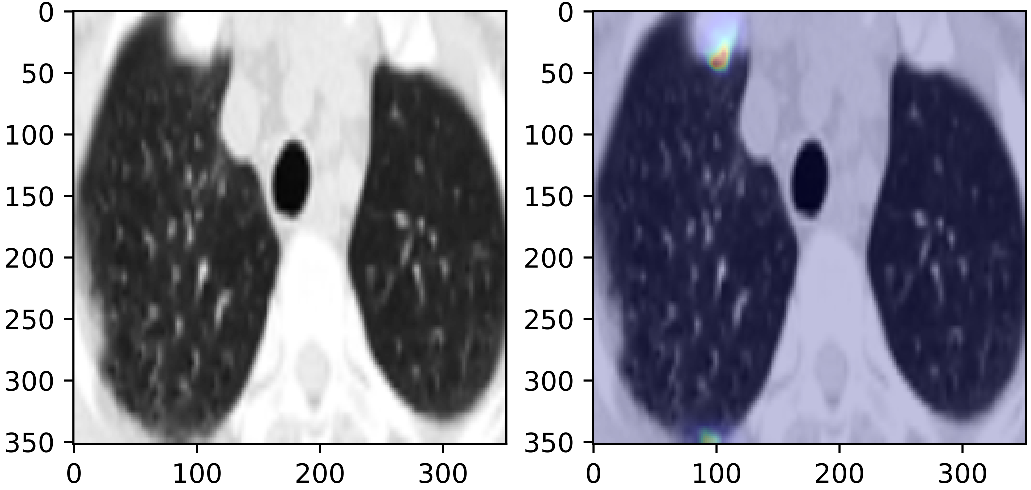

Lesion Proportion: 9.32%

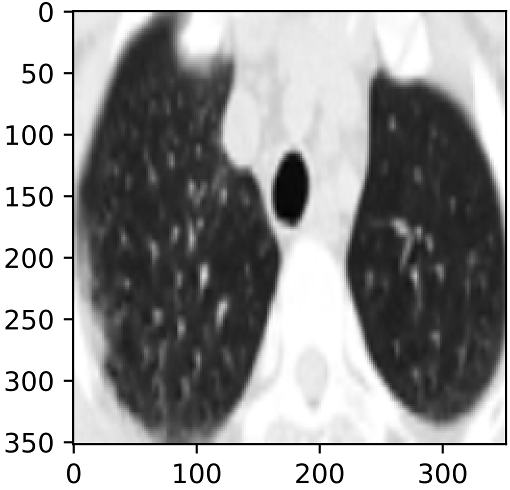

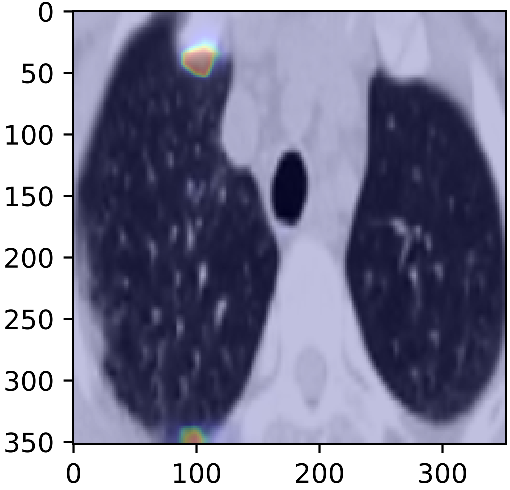

Lesion Proportion: 11.50%

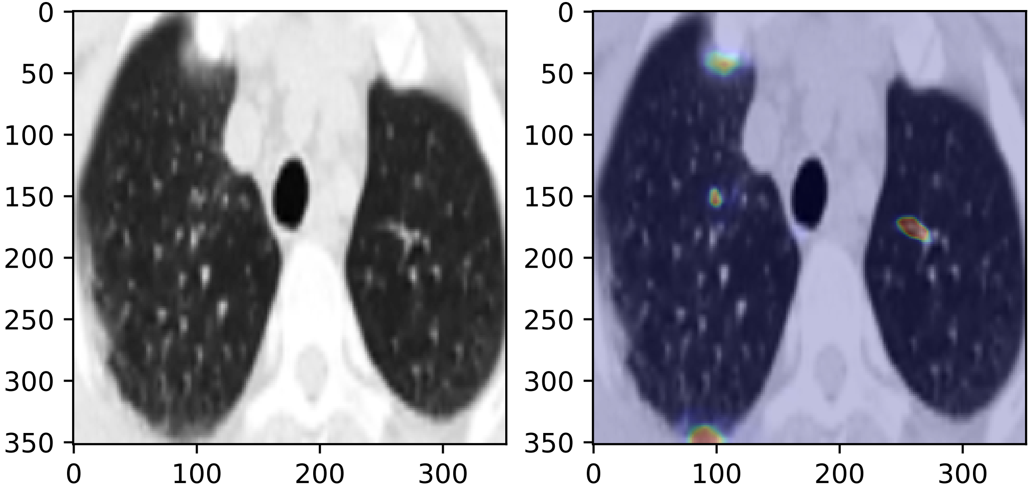

Lesion Proportion: 9.62%

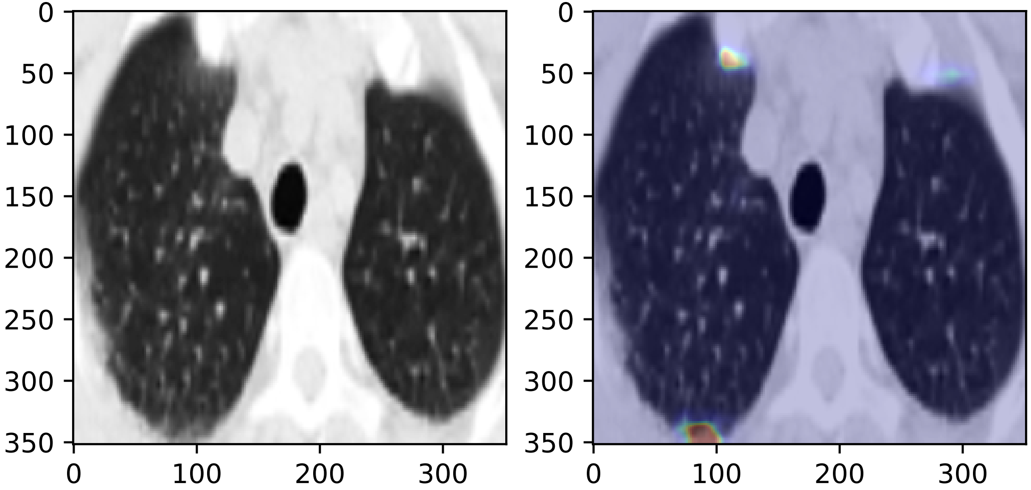

Lesion Proportion: 11.58%

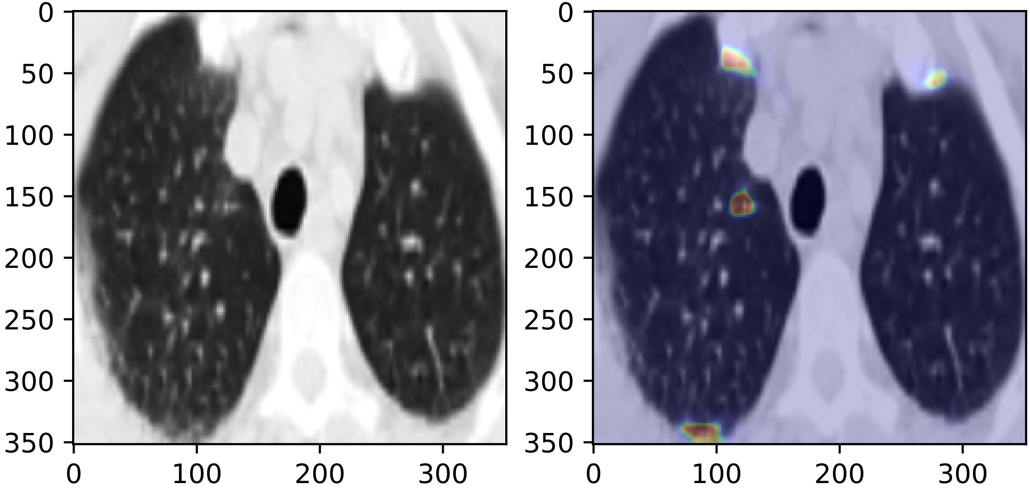

Lesion Proportion: 13.48%

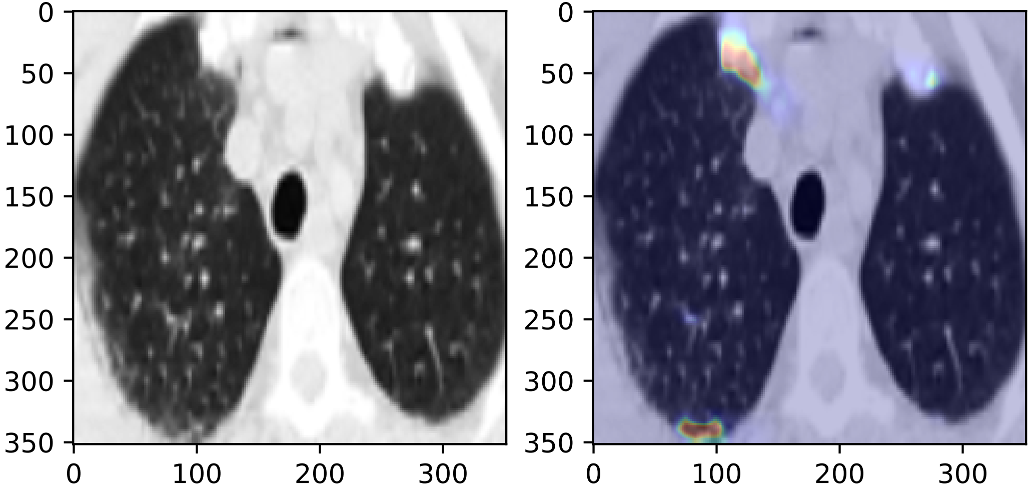

Lesion Proportion: 17.92%

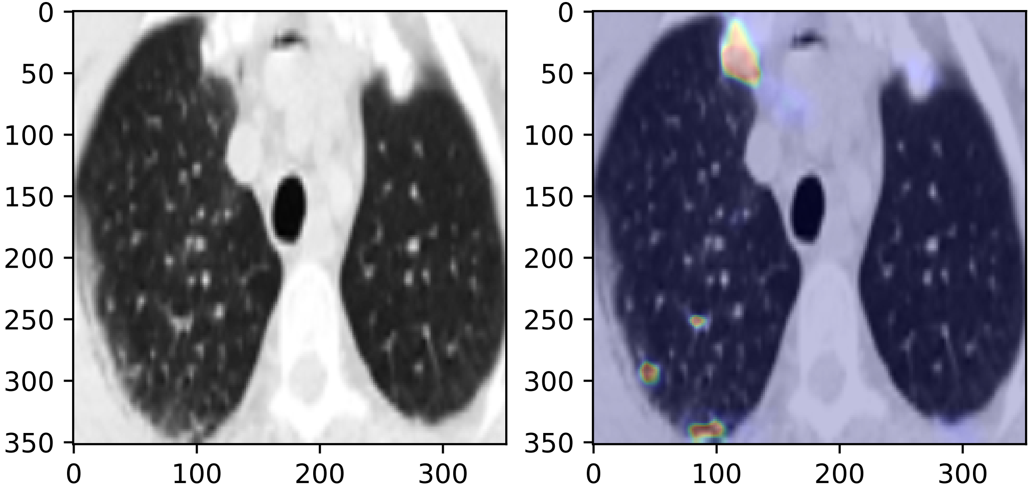

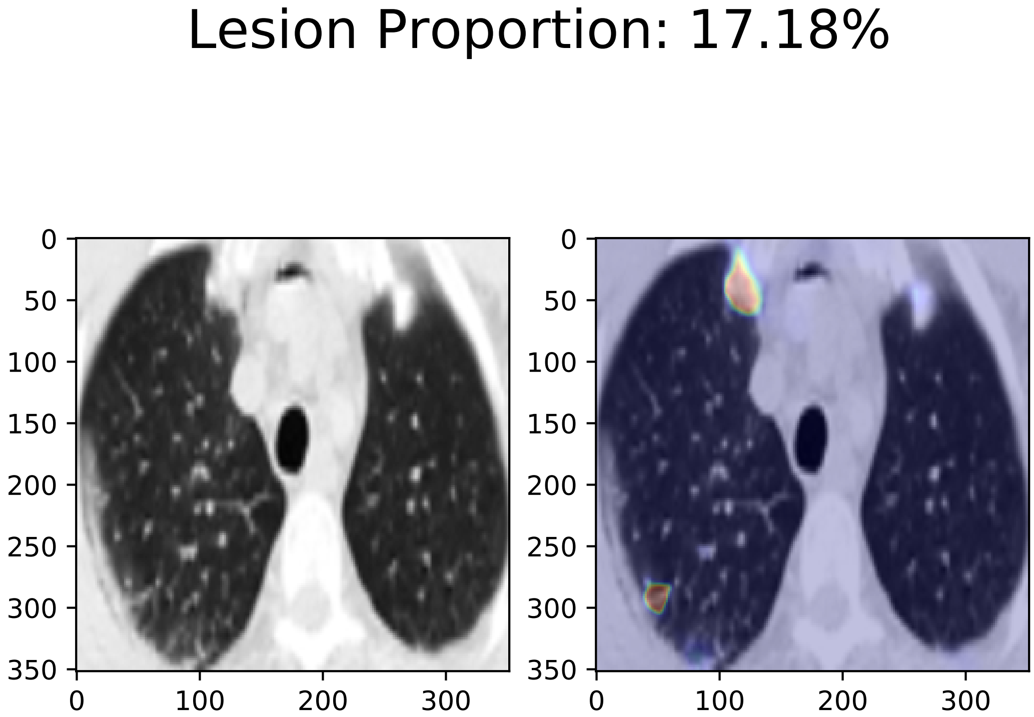
Lesion Proportion: 17.18%
0
50
100
150
200
250
300
350
0
100
200
300
0
50
100
150
200
250
300
350
0
100
200
300

Lesion Proportion: 13.55%

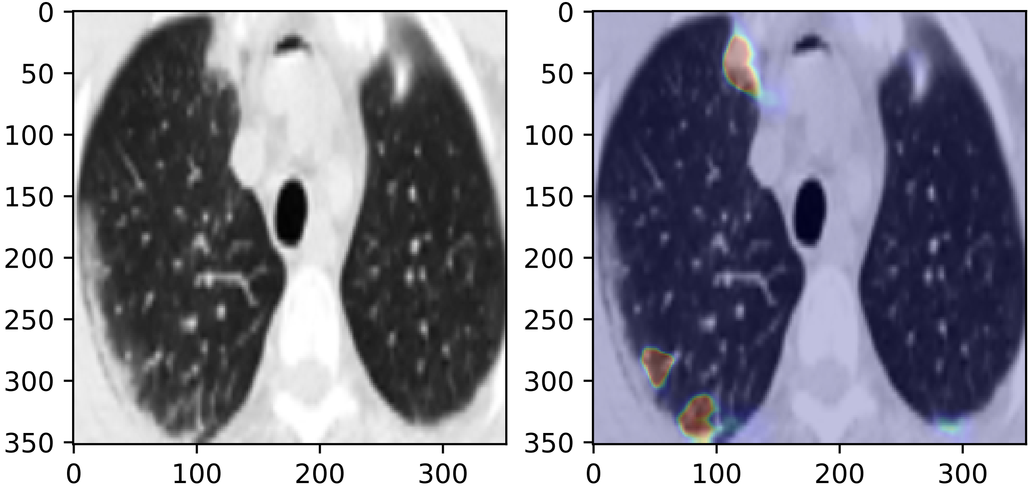

Lesion Proportion: 17.06%

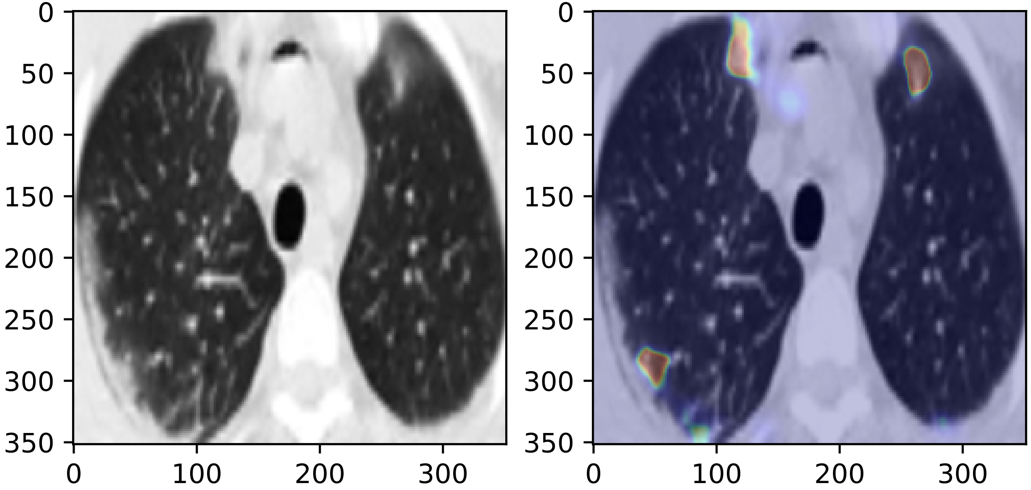

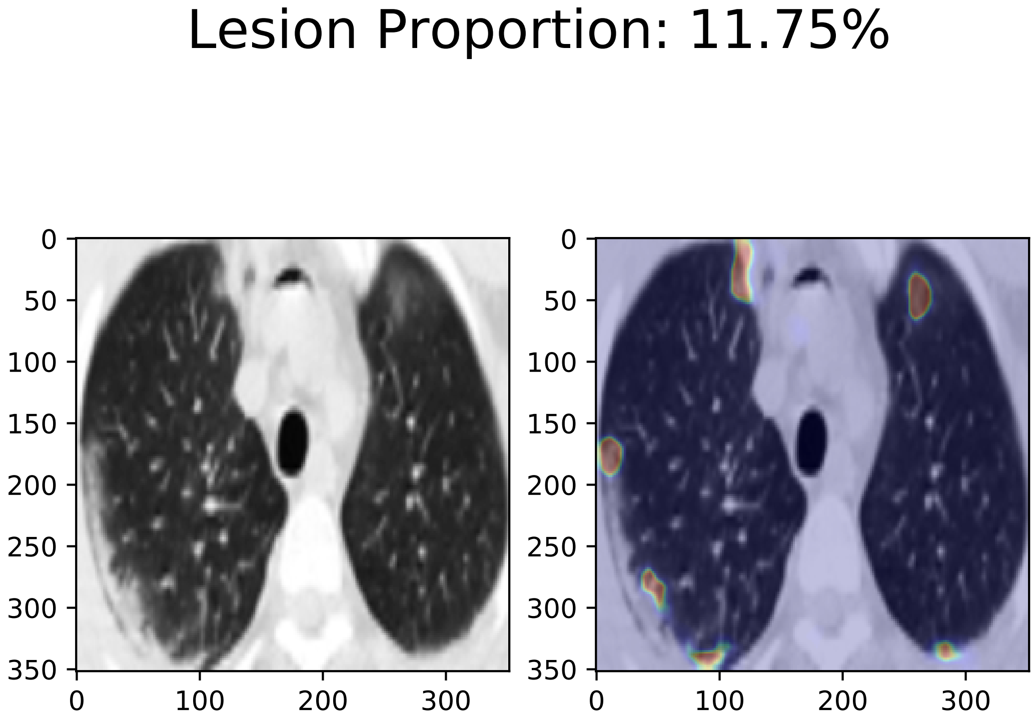

Lesion Proportion: 11.75%
0
50
100
150
200
250
300
350
0
100
200
300

Lesion Proportion: 12.40%

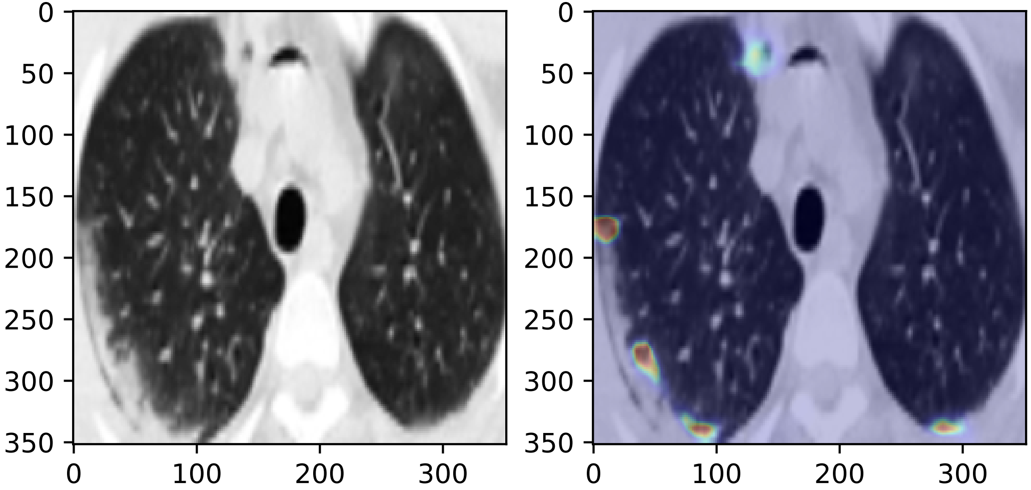

Lesion Proportion: 6.96%

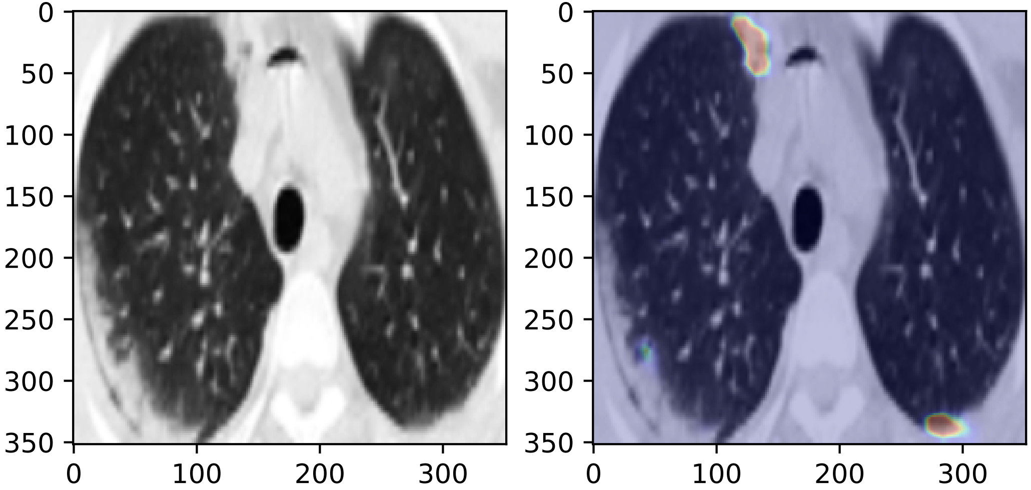

Lesion Proportion: 8.02%

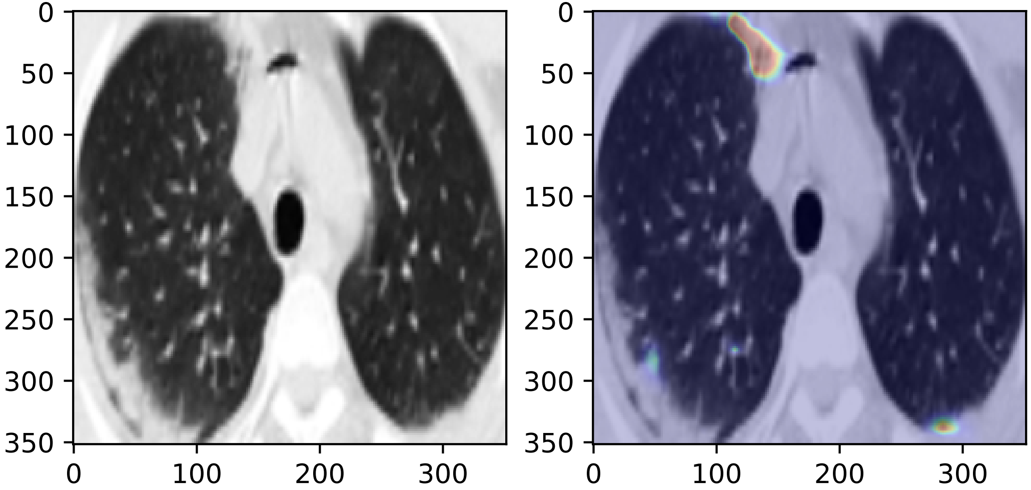

Lesion Proportion: 10.07%

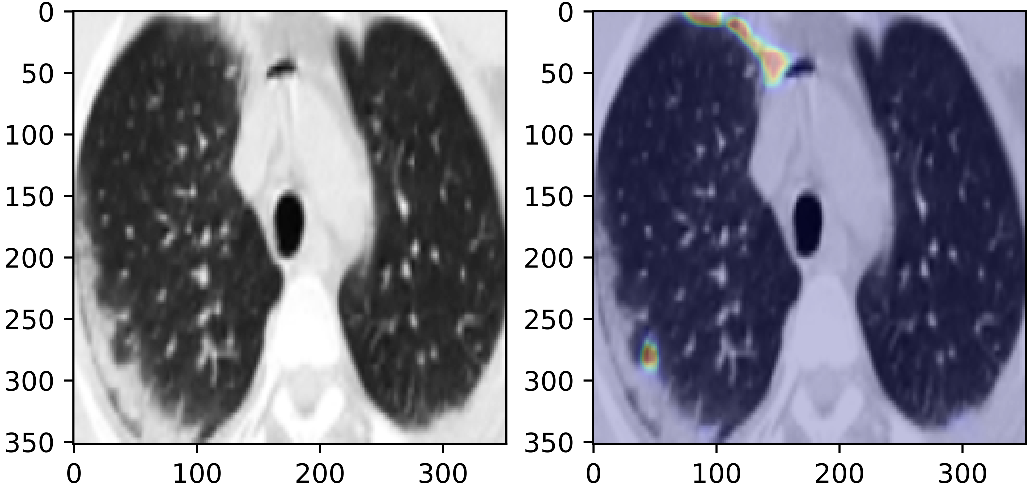

Lesion Proportion: 13.00%

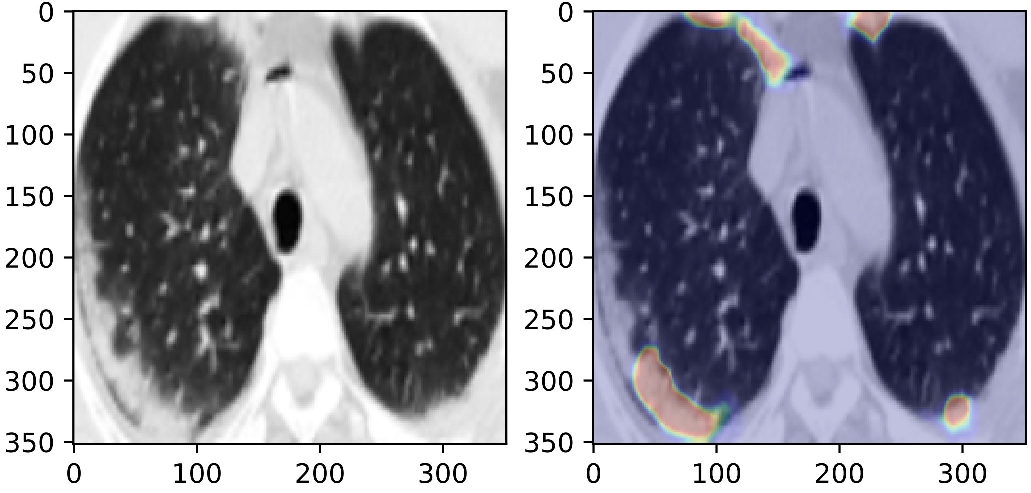

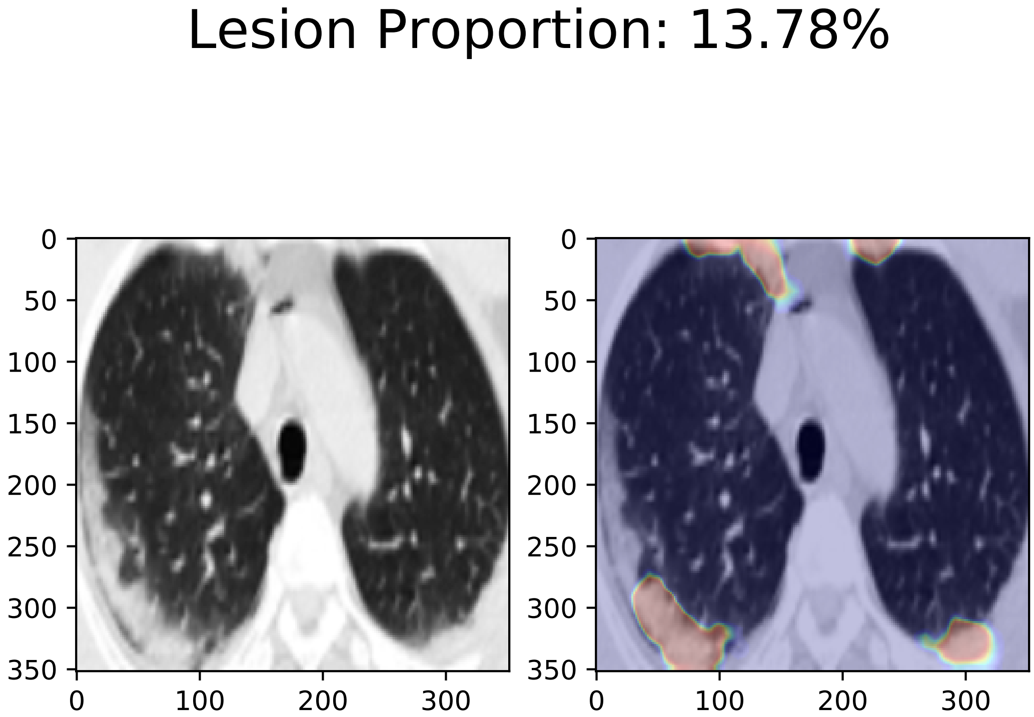

Lesion Proportion: 13.78%
0
50
100
150
200
250
300
350
0
100
200
300

Lesion Proportion: 14.49%

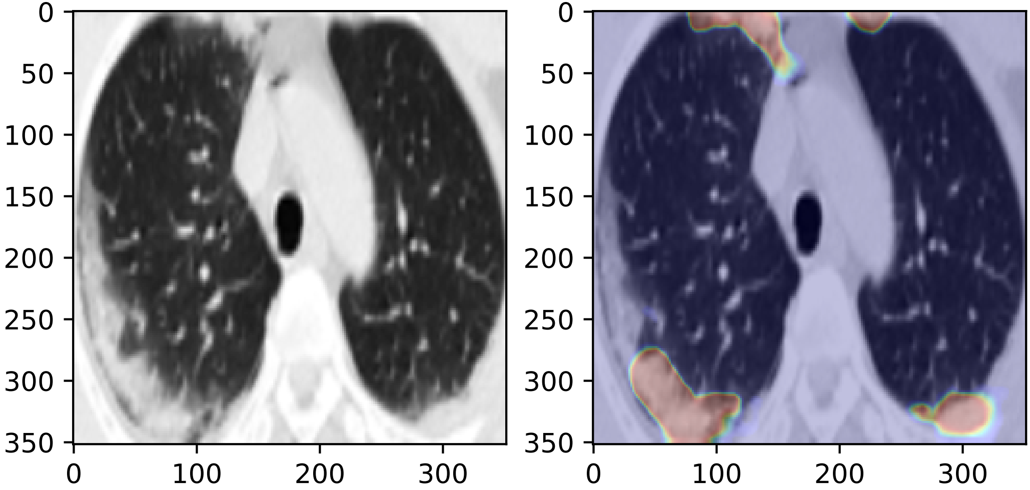

Lesion Proportion: 15.37%

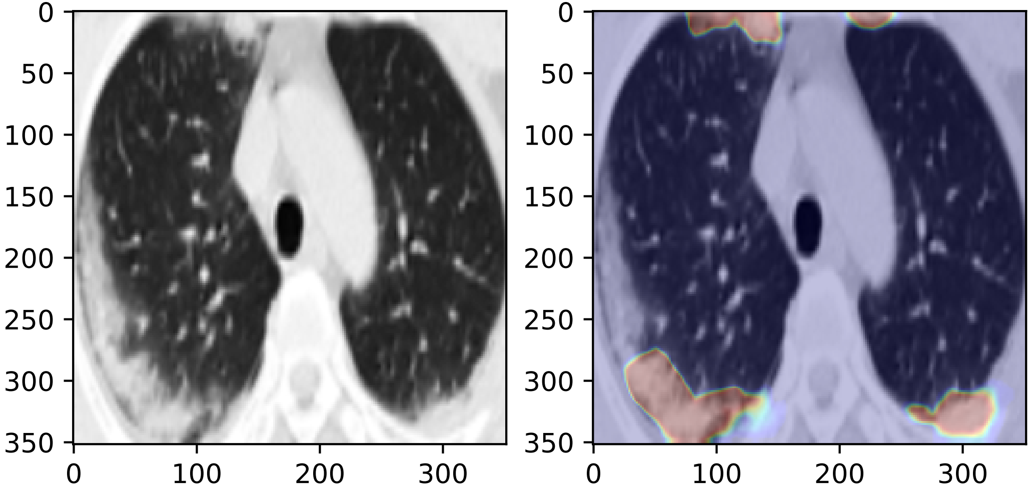

Lesion Proportion: 14.42%

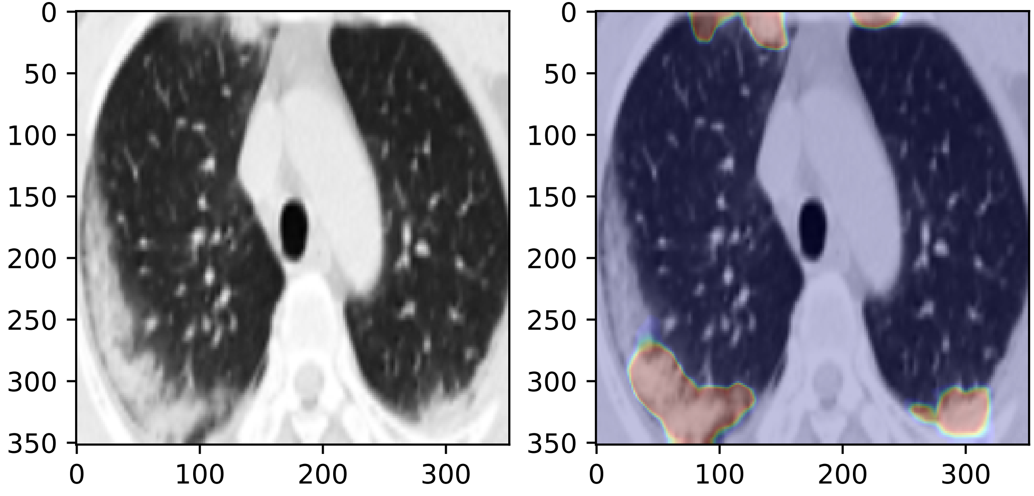

Lesion Proportion: 15.56%

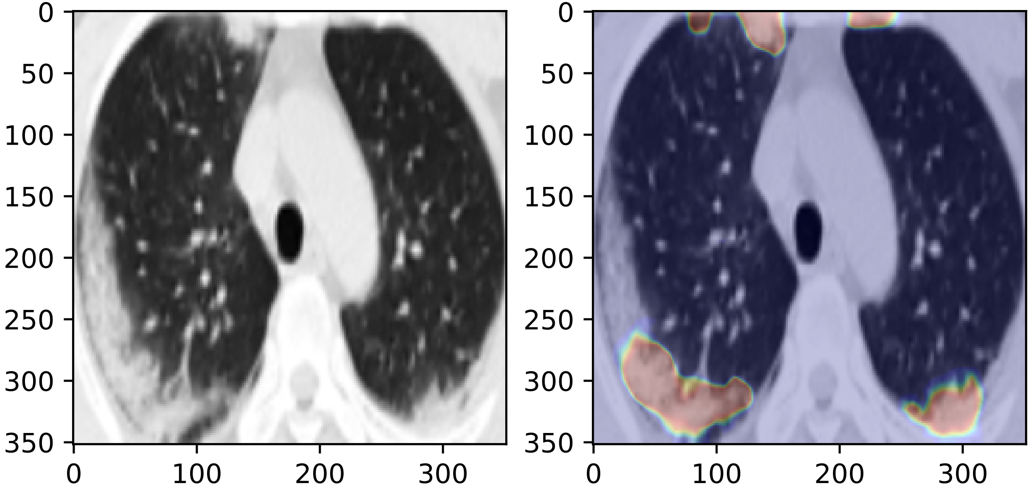

Lesion Proportion: 17.11%

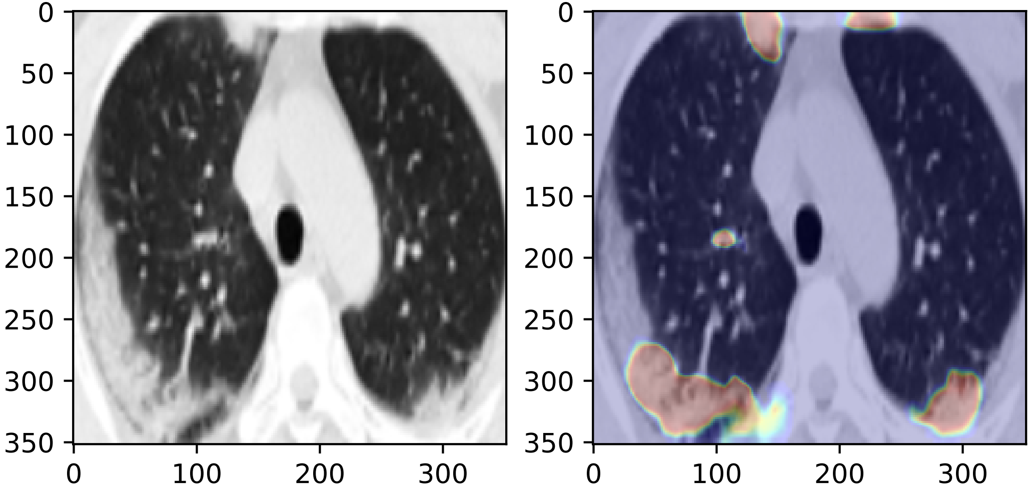

Lesion Proportion: 17.43%

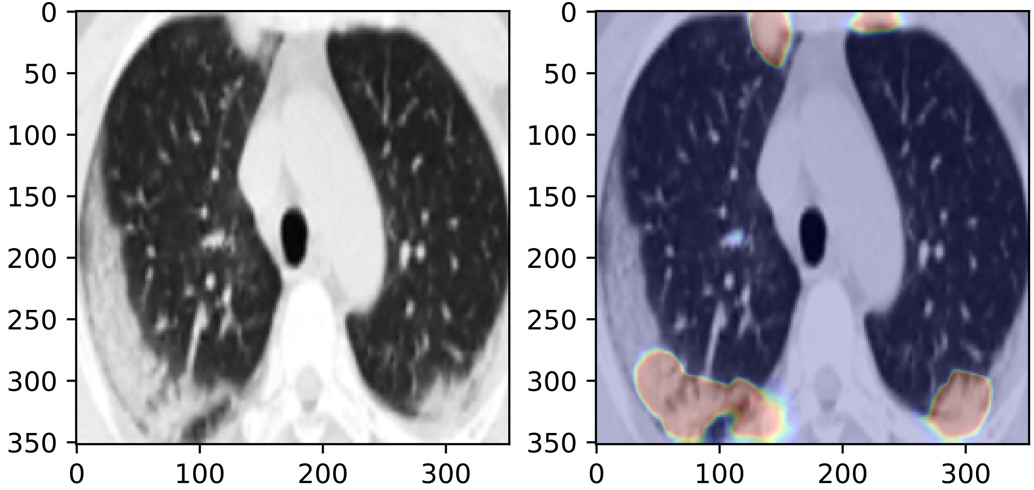

Lesion Proportion: 18.93%

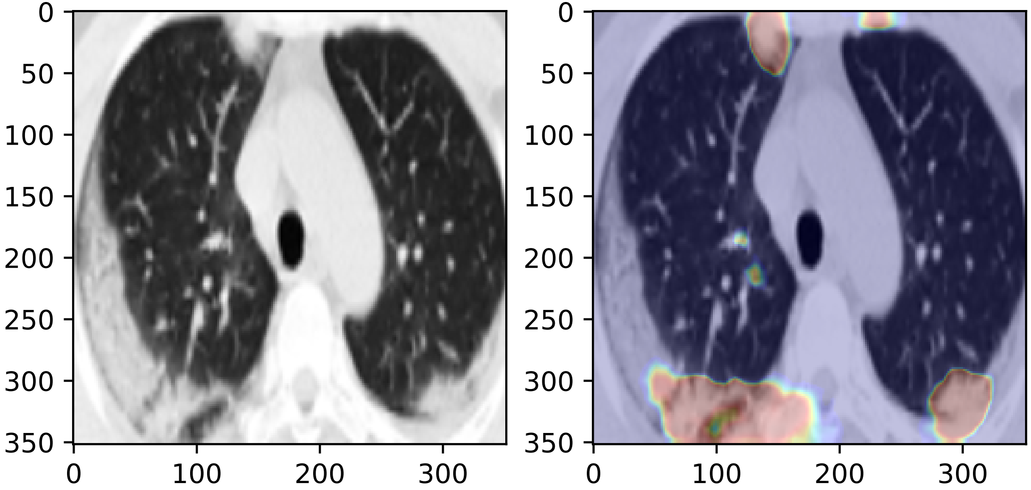

Lesion Proportion: 18.02%

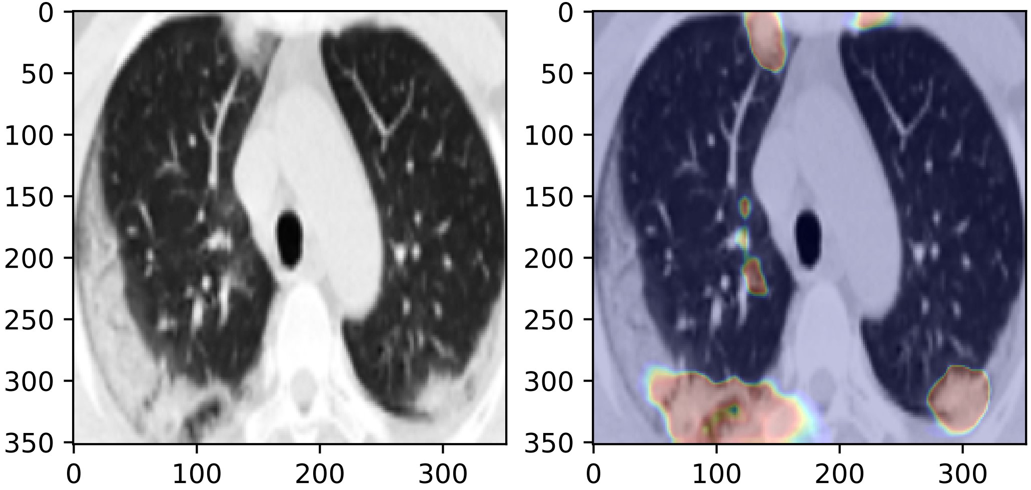

Lesion Proportion: 16.34%

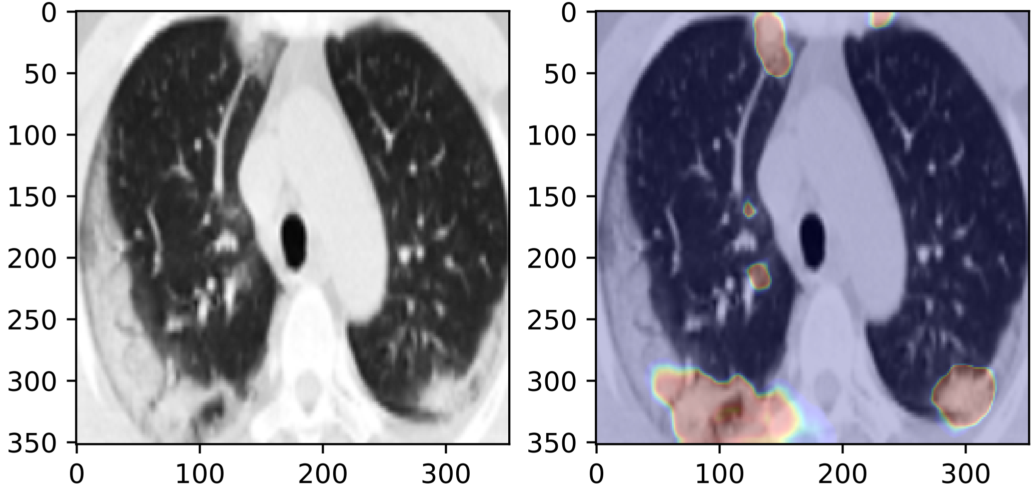

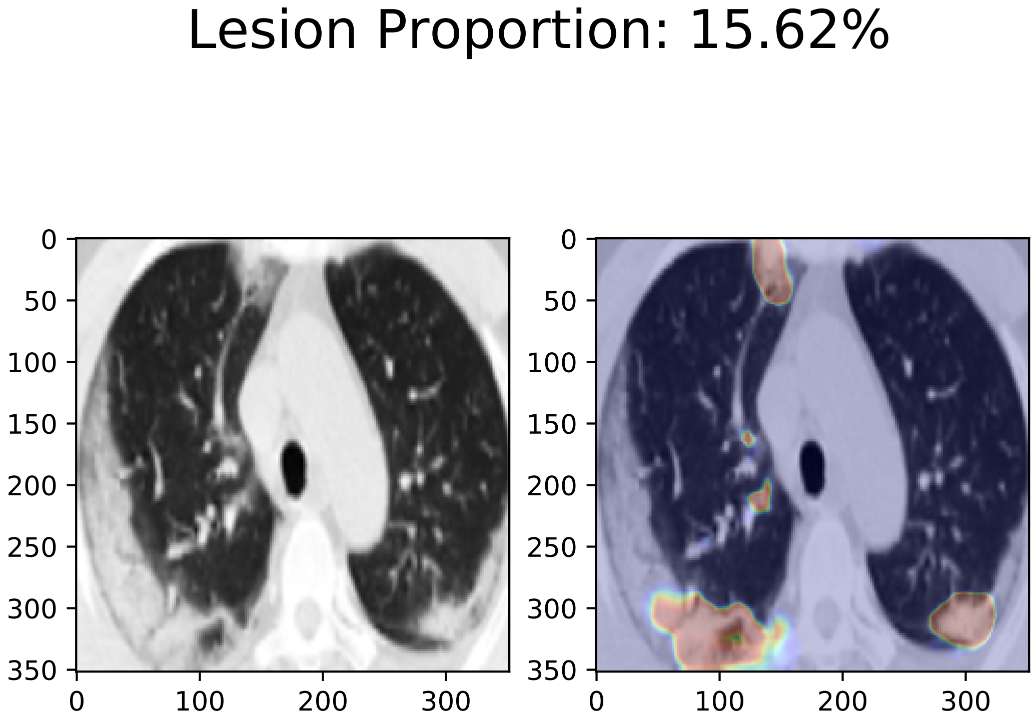
Lesion Proportion: 15.62%
0
50
100
150
200
250
300
350
0
100
200
300
0
50
100
150
200
250
300
350
0
100
200
300

Lesion Proportion: 13.89%

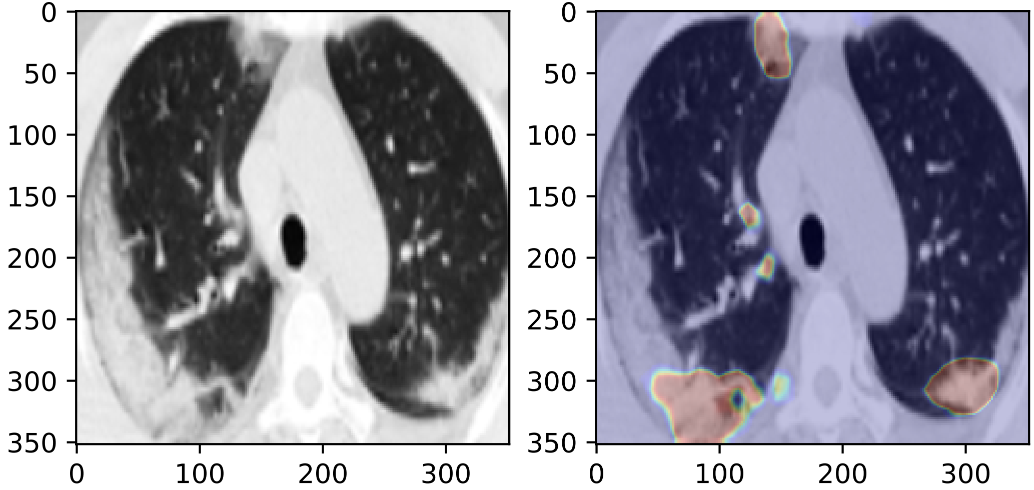

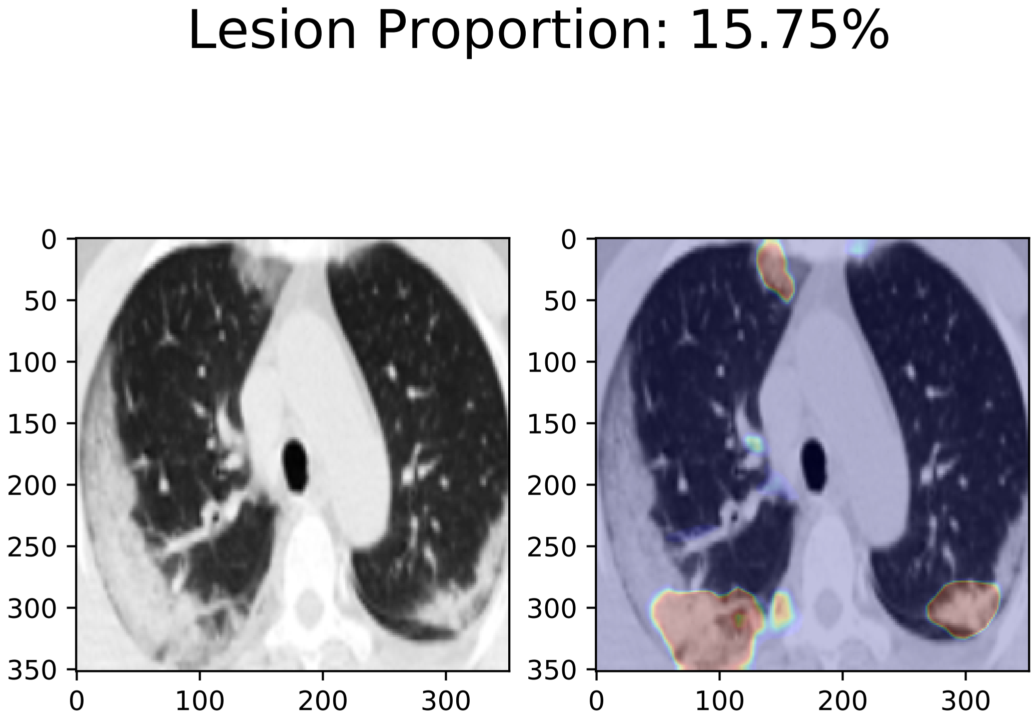
Lesion Proportion: 15.75%
0
50
100
150
200
250
300
350
0
100
200
300

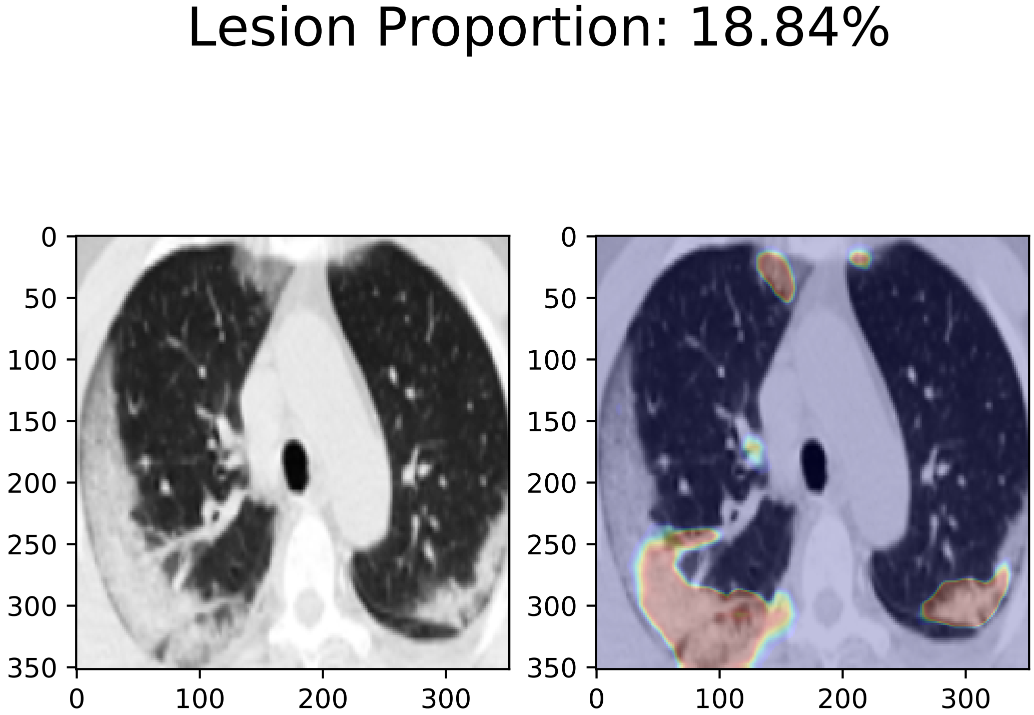
Lesion Proportion: 18.84%
0
50
100
150
200
250
300
350
0
100
200
300

Lesion Proportion: 19.46%

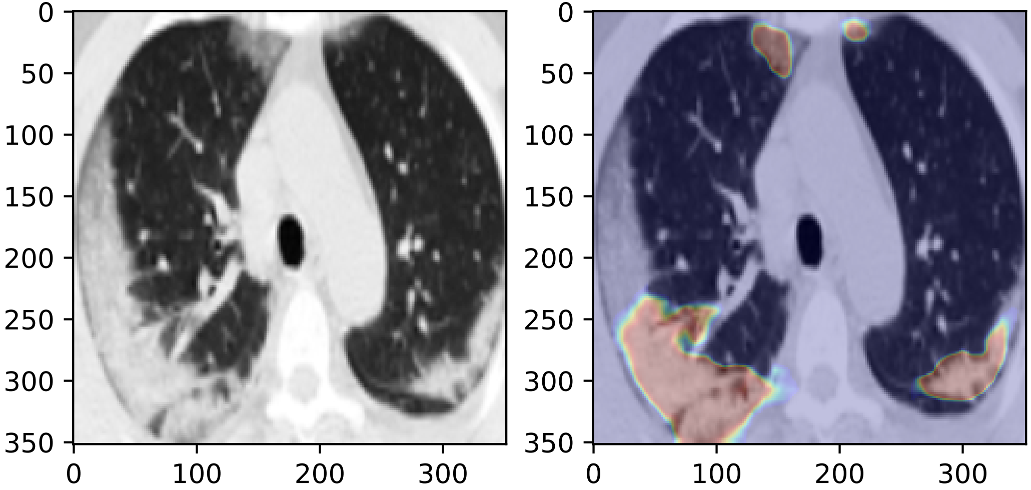

Lesion Proportion: 18.06%

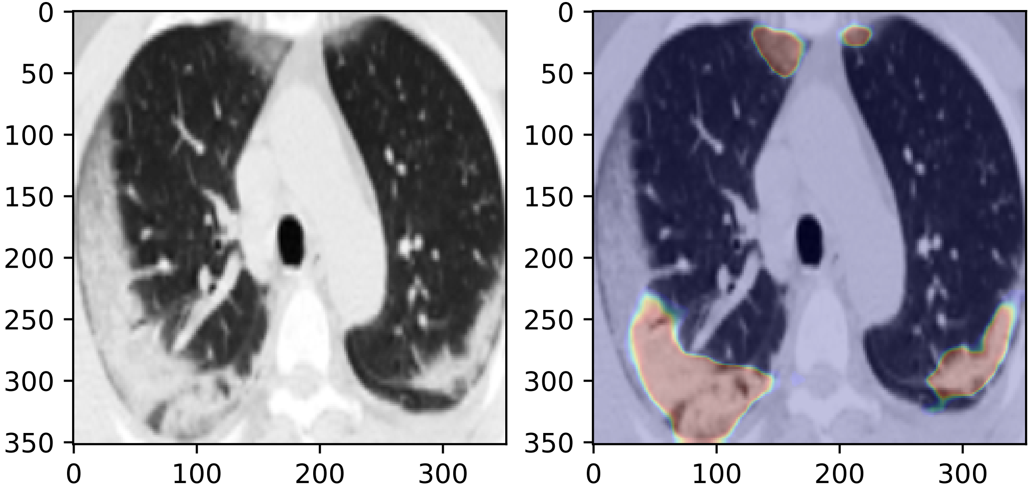

Lesion Proportion: 17.36%

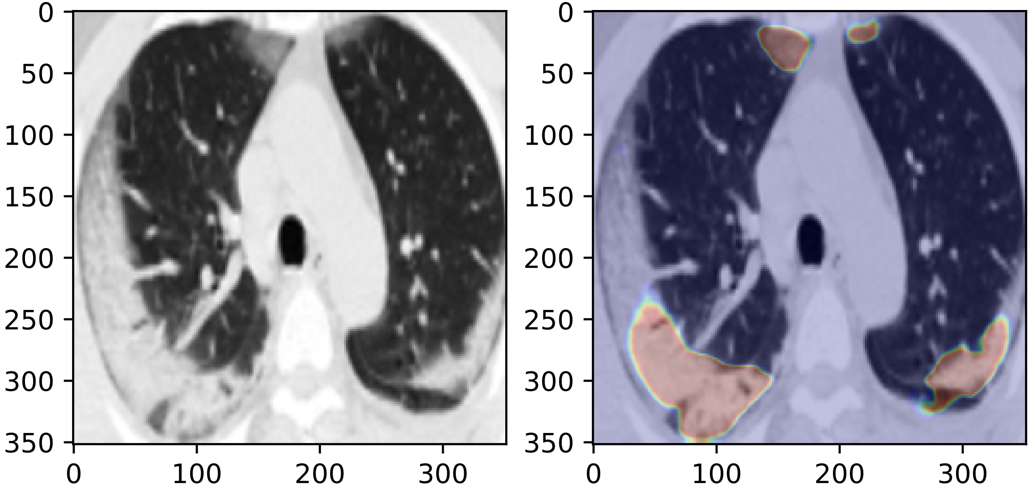

Lesion Proportion: 21.81%

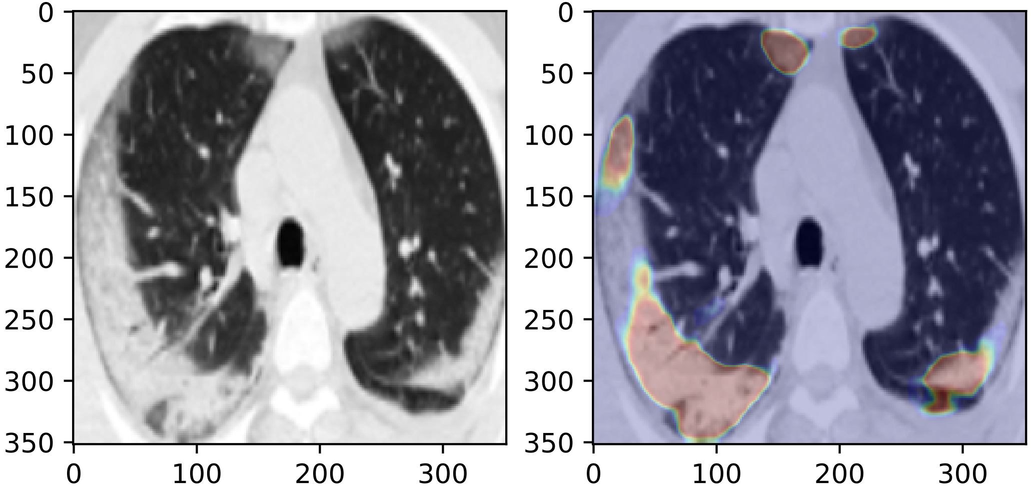

Lesion Proportion: 21.58%

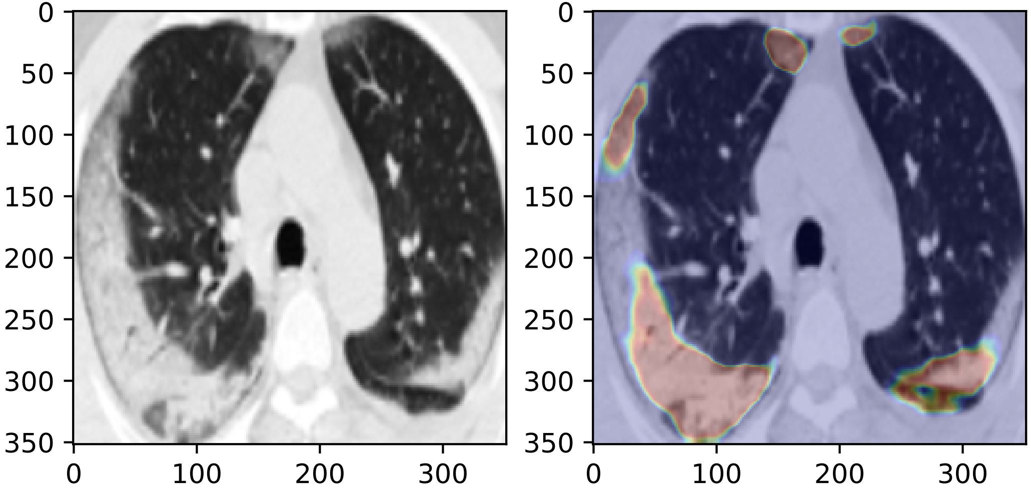

Lesion Proportion: 23.83%

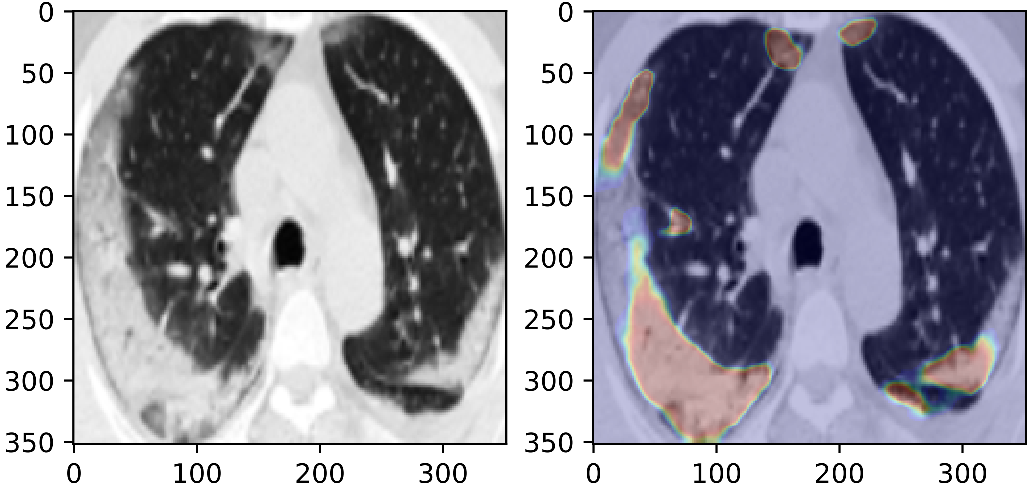

Lesion Proportion: 24.01%

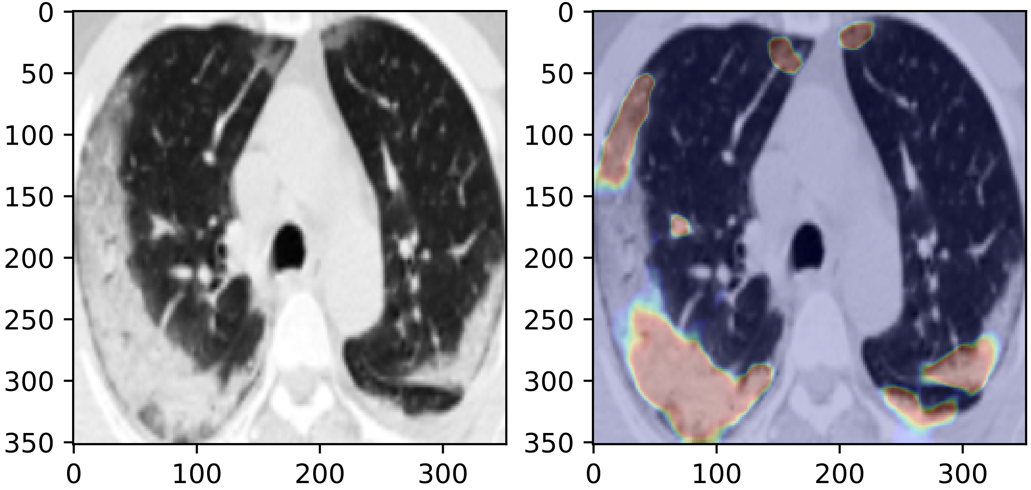

Lesion Proportion: 26.82%

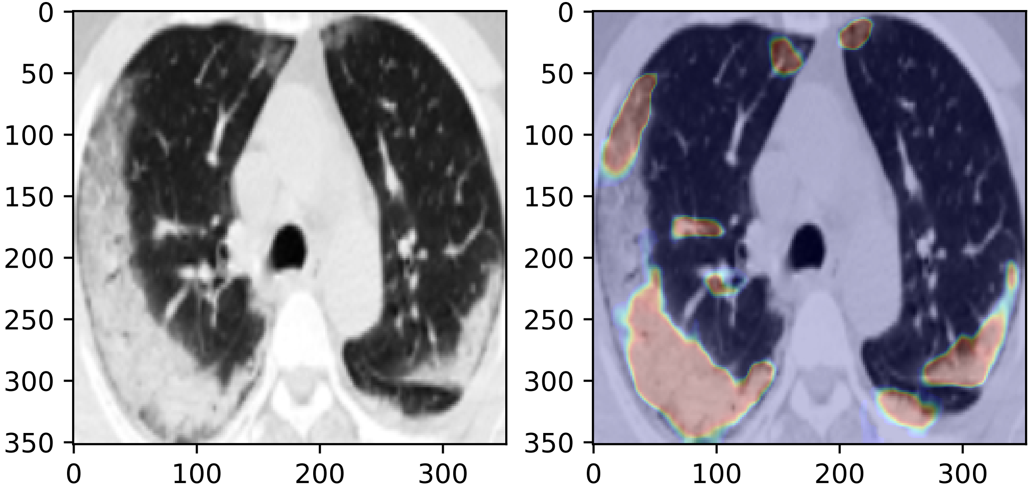

Lesion Proportion: 23.10%

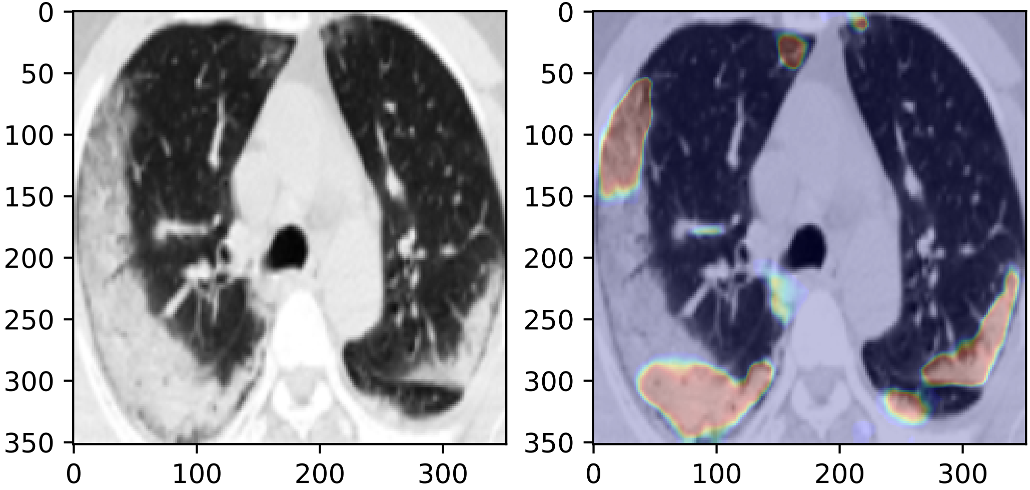

Lesion Proportion: 25.49%

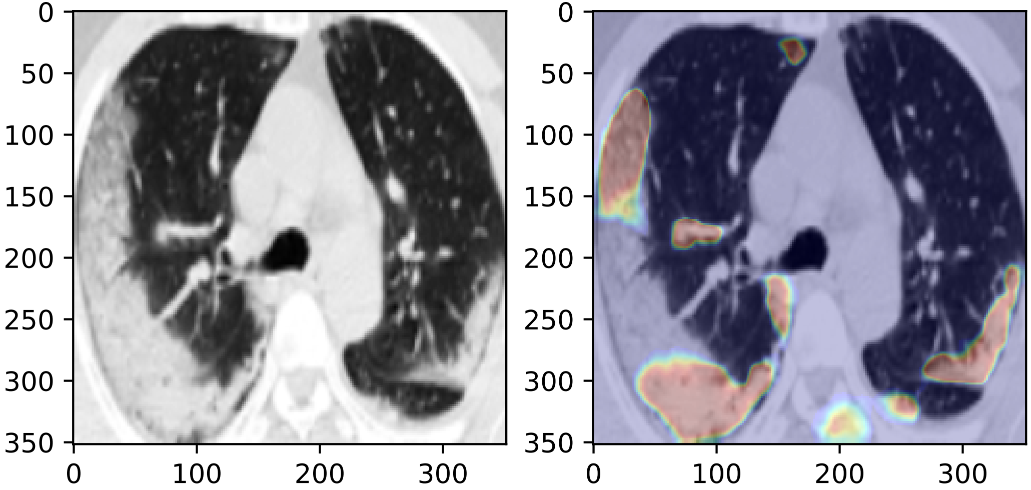

Lesion Proportion: 20.28%

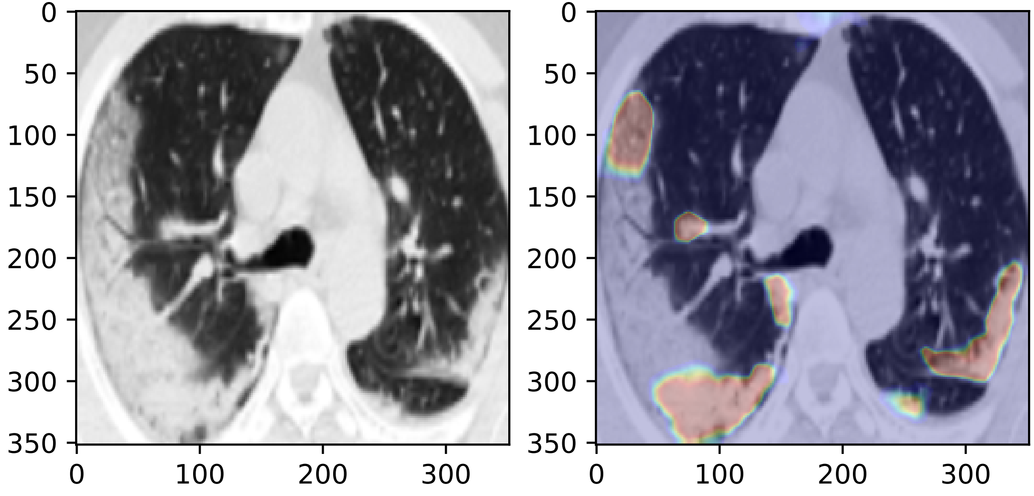

Lesion Proportion: 17.13%

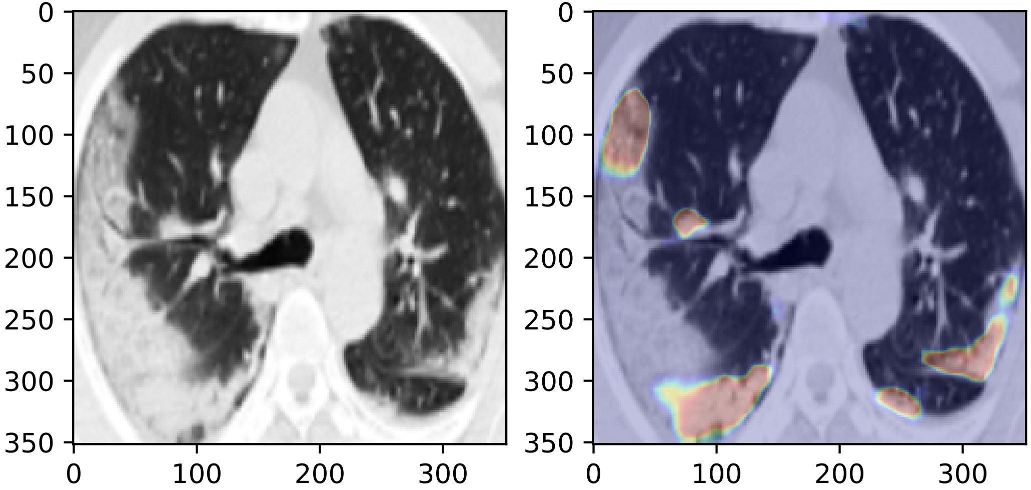

Lesion Proportion: 17.82%

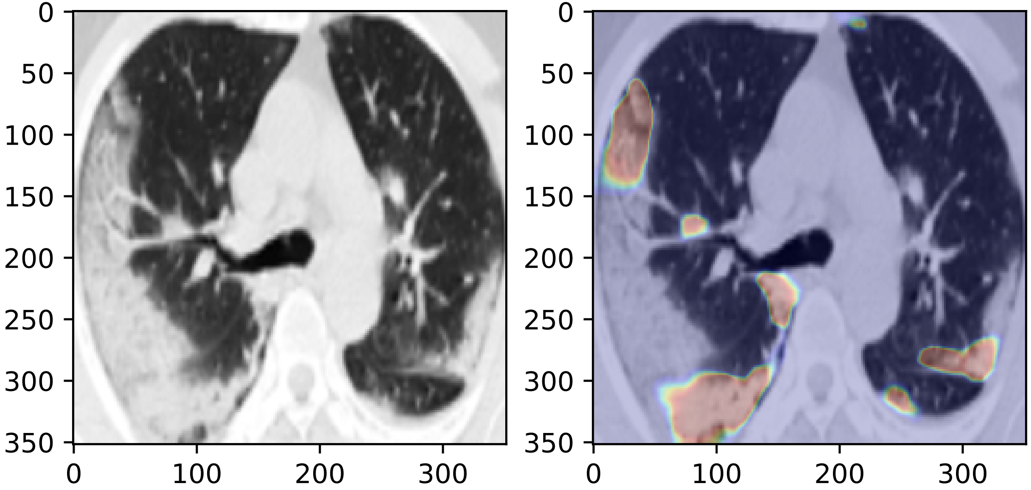

Lesion Proportion: 16.49%

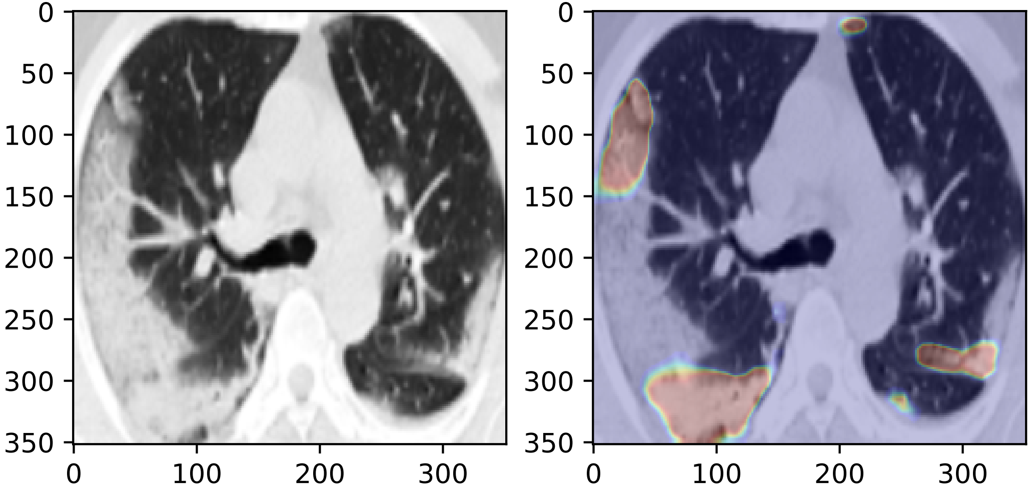

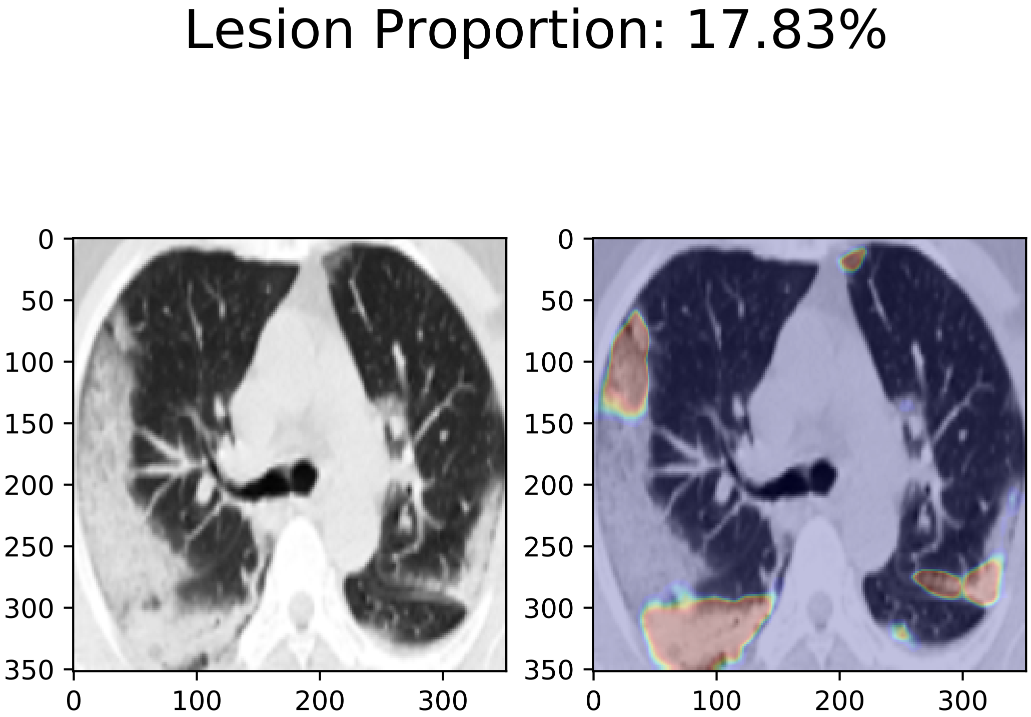
Lesion Proportion: 17.83%
0
50
100
150
200
250
300
350
0
100
200
300

Lesion Proportion: 19.26%

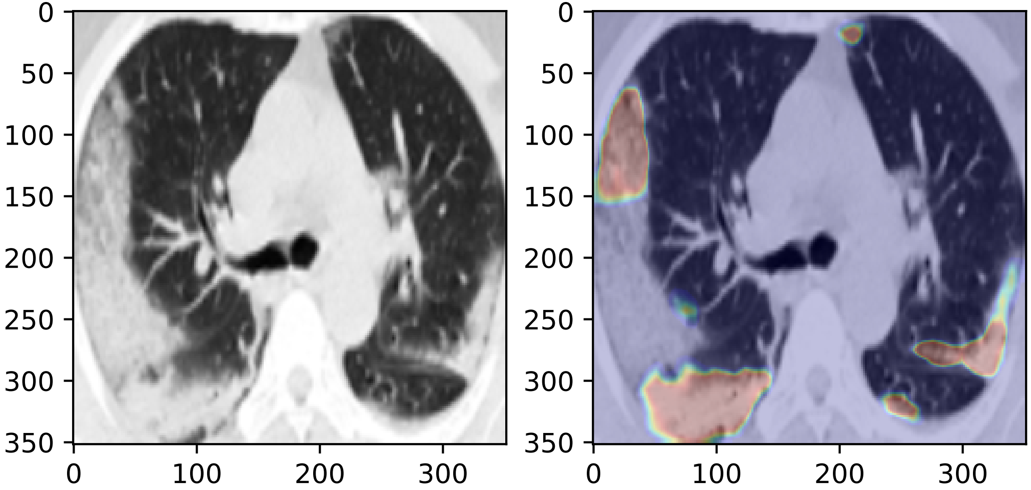

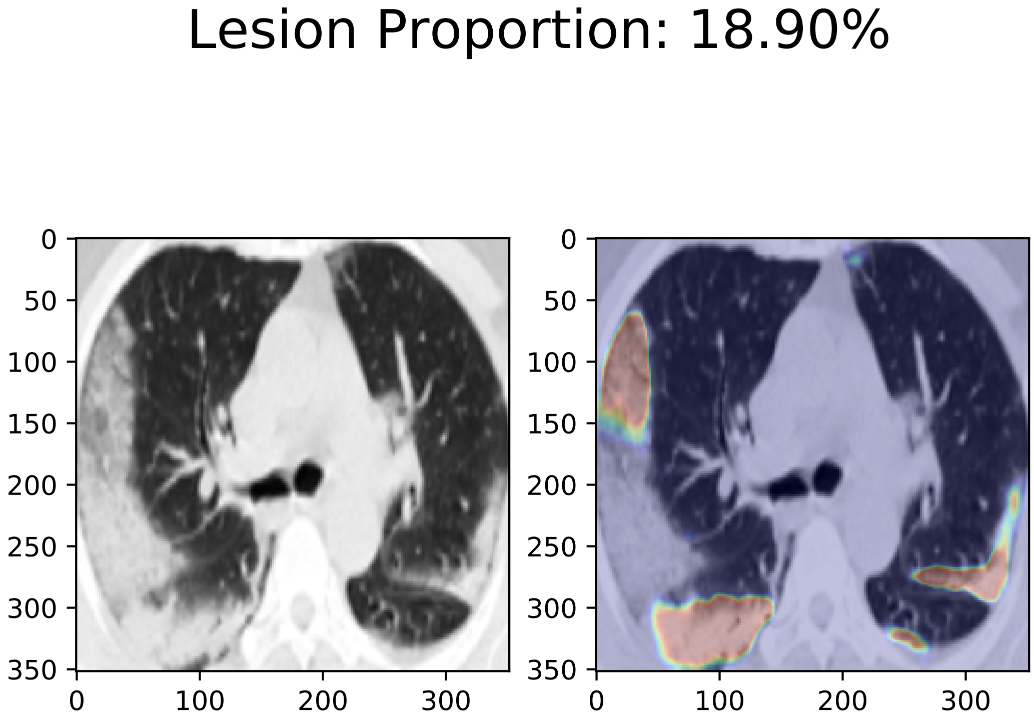
Lesion Proportion: 18.90%
0
50
100
150
200
250
300
350
0
100
200
300

Lesion Proportion: 22.68%

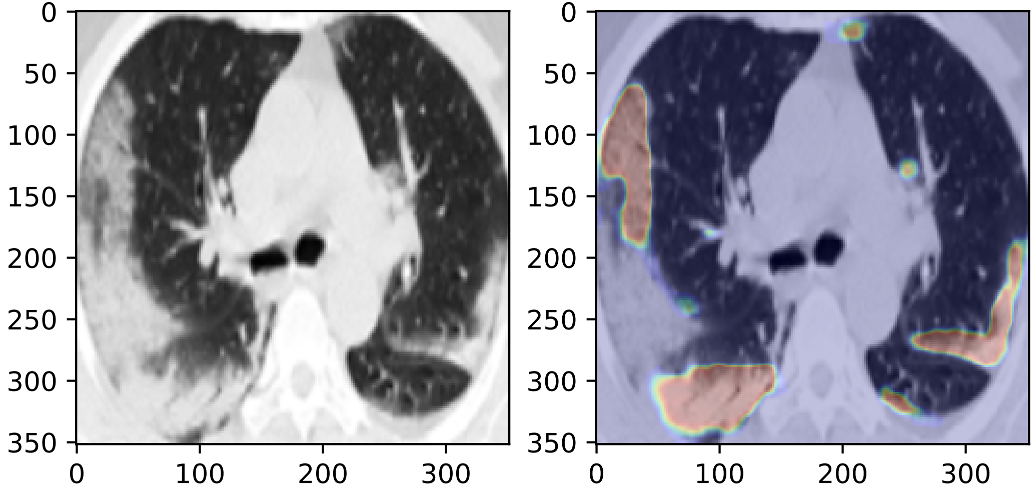

Lesion Proportion: 23.15%

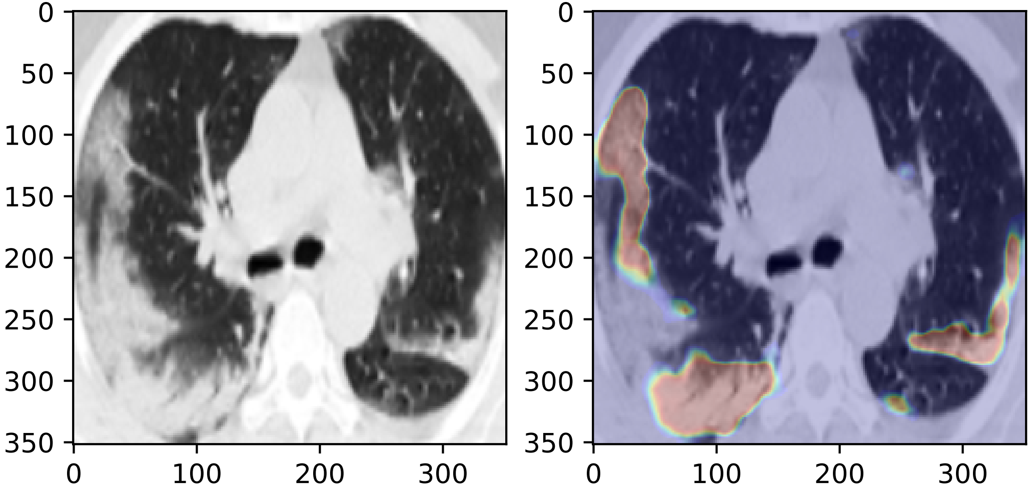

Lesion Proportion: 25.56%

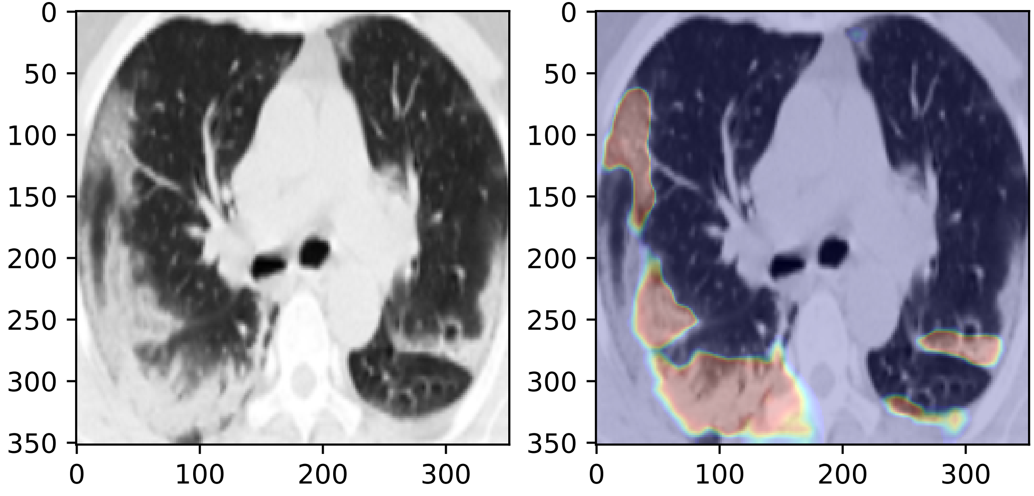

Lesion Proportion: 24.27%

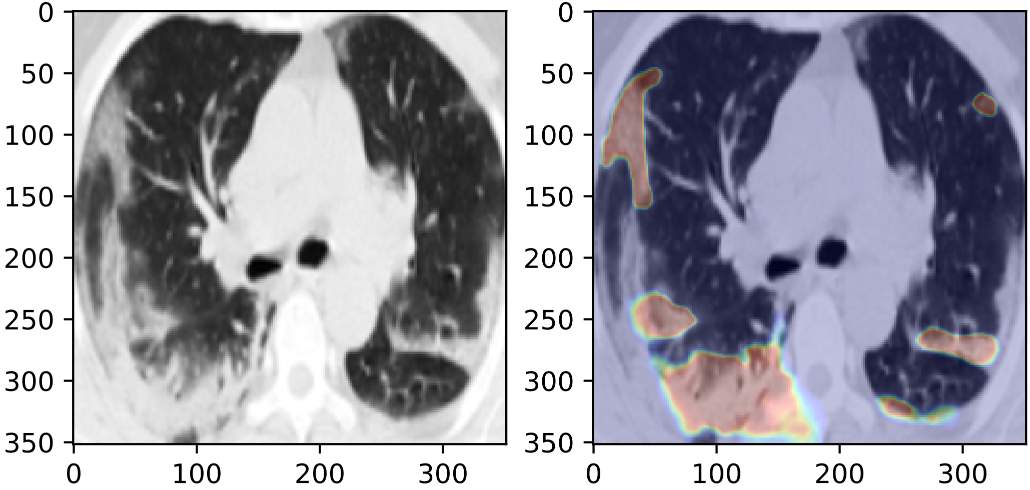

Lesion Proportion: 25.79%

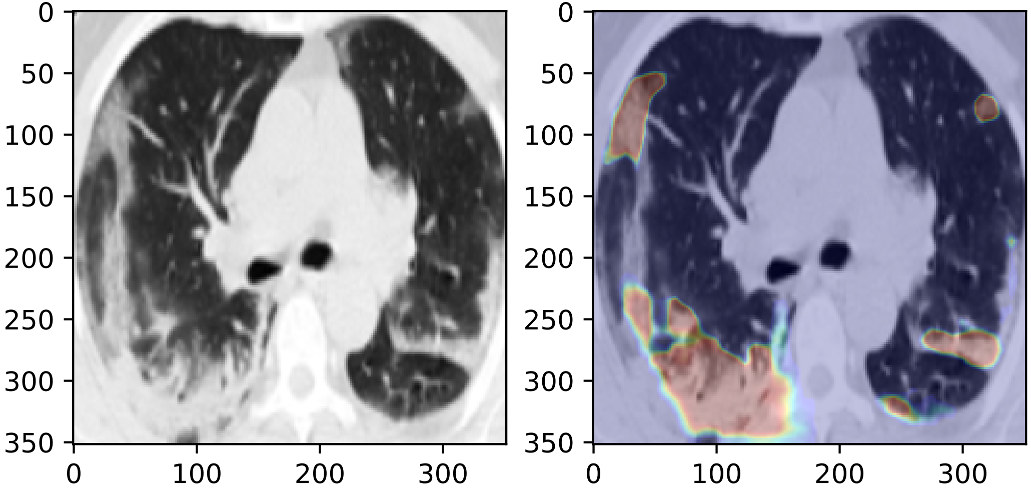

Lesion Proportion: 28.38%

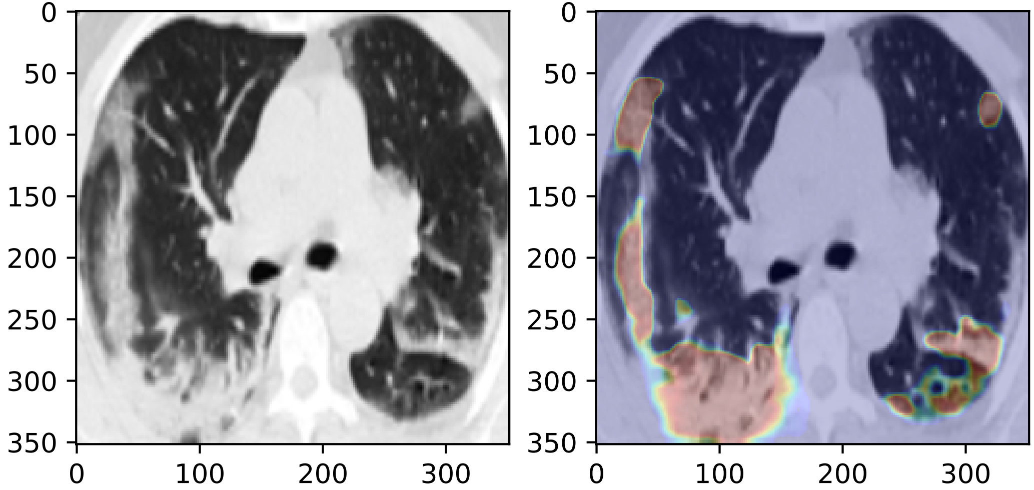

Lesion Proportion: 31.64%

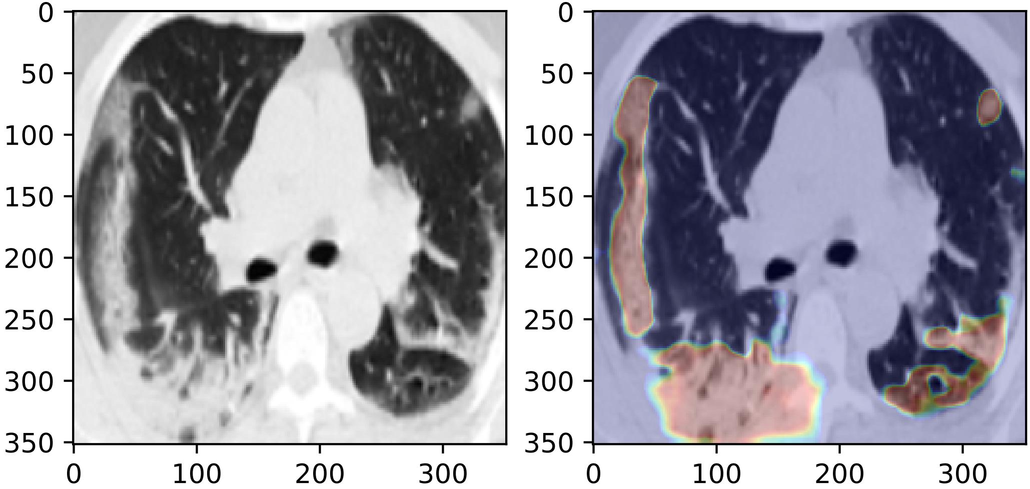

Lesion Proportion: 27.24%

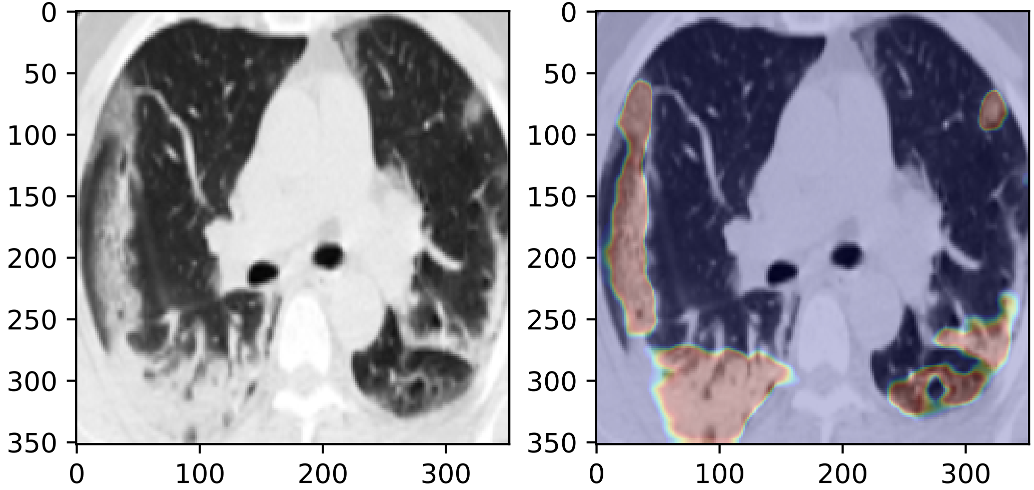

Lesion Proportion: 28.18%

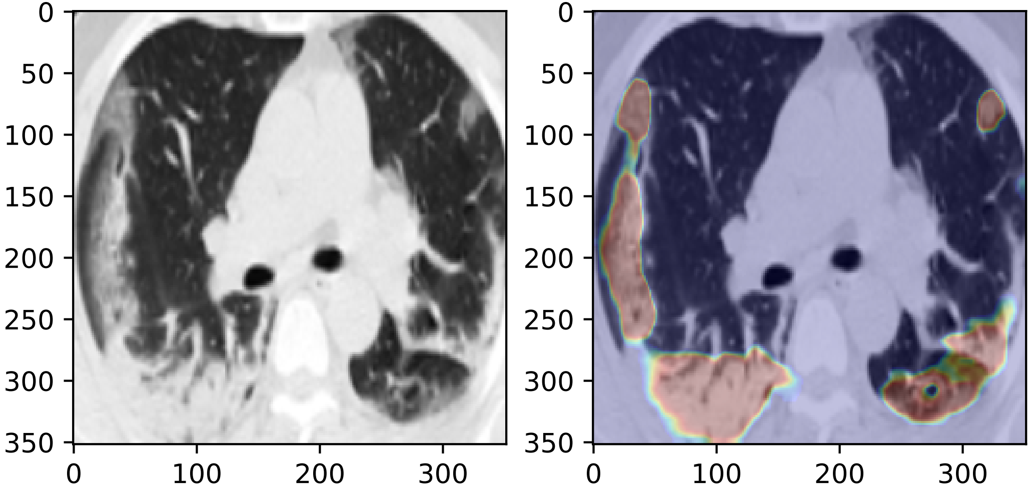

Lesion Proportion: 27.69%

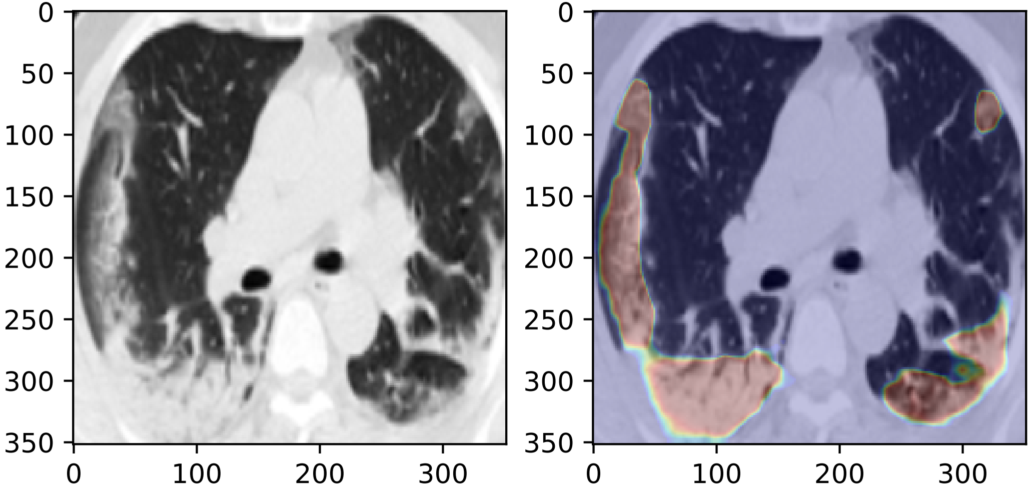

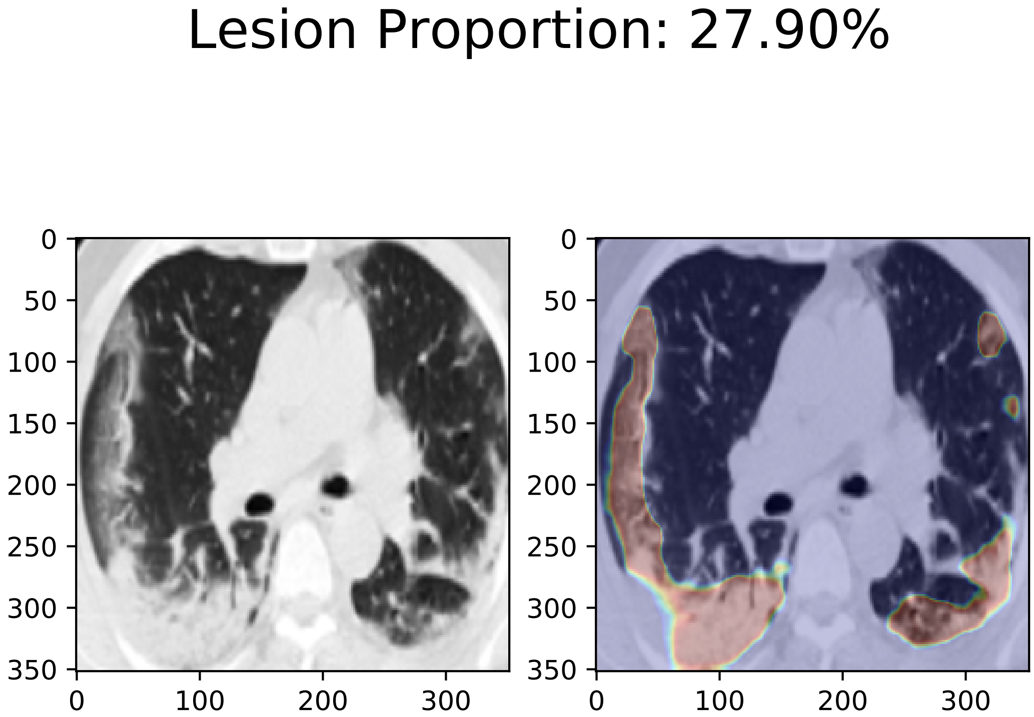

Lesion Proportion: 27.90%
0
50
100
150
200
250
300
350
0
100
200
300

Lesion Proportion: 31.99%

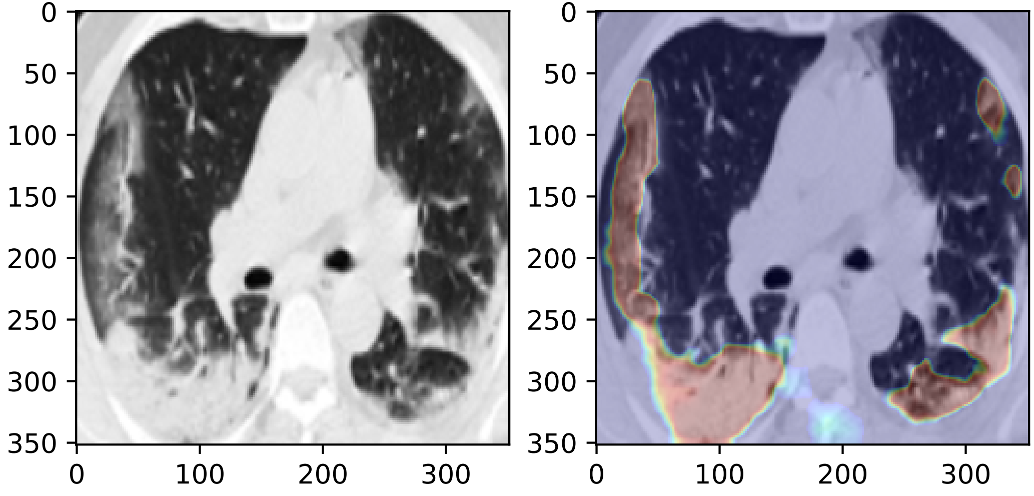

Lesion Proportion: 37.76%

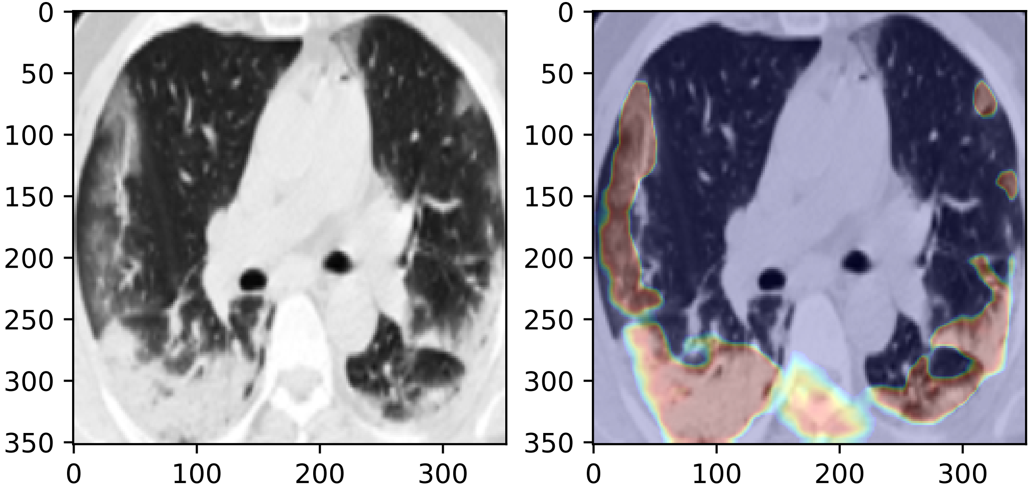

Lesion Proportion: 35.92%

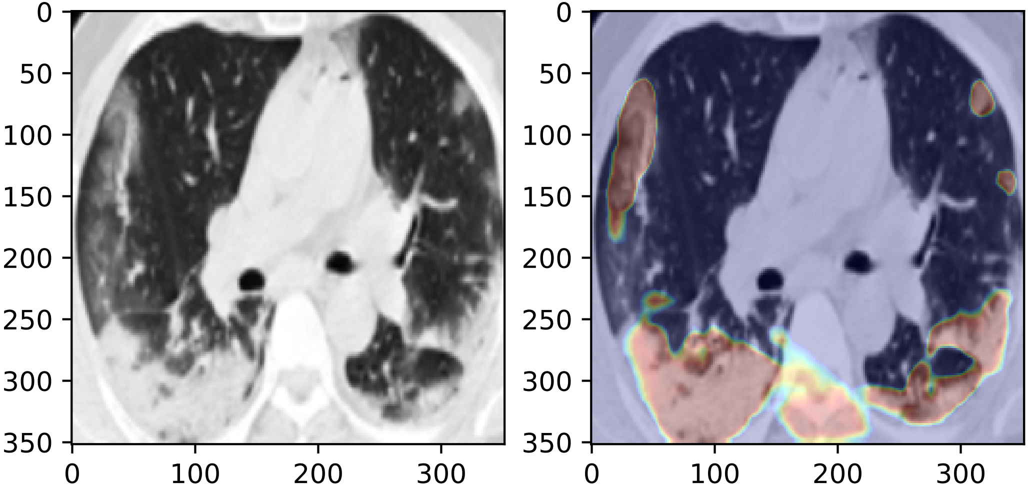

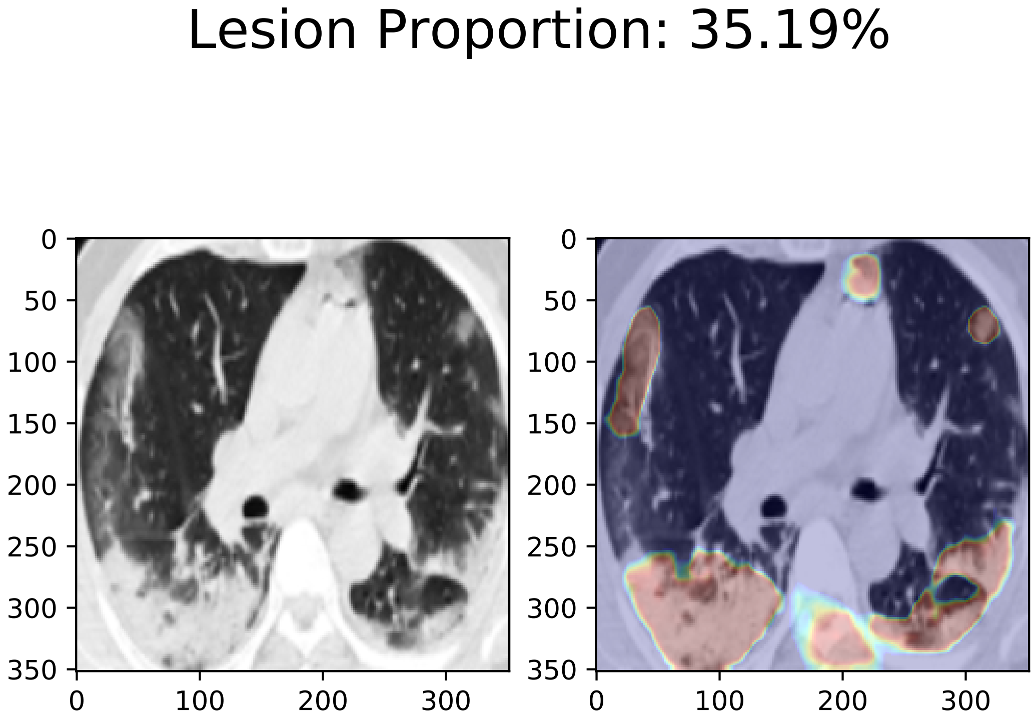
Lesion Proportion: 35.19%
0
50
100
150
200
250
300
350
0
100
200
300

Lesion Proportion: 34.63%

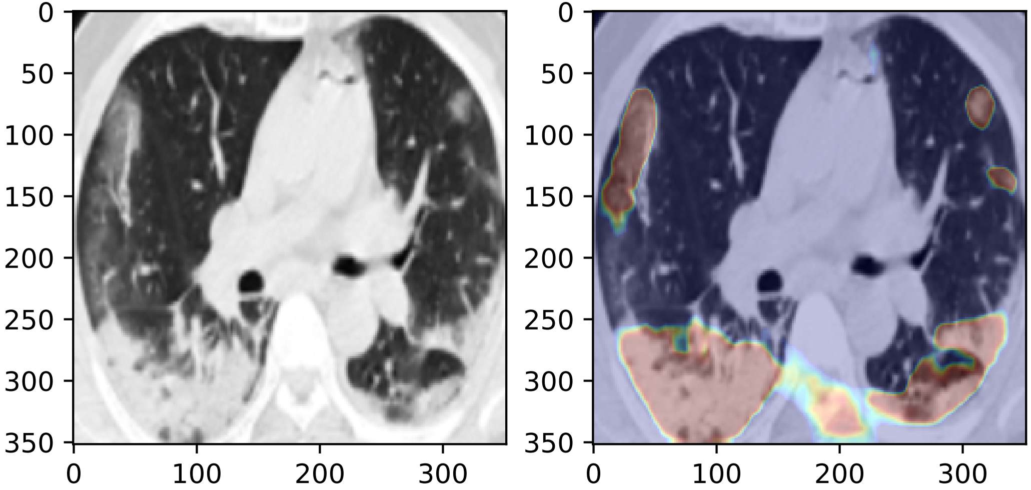

Lesion Proportion: 31.43%

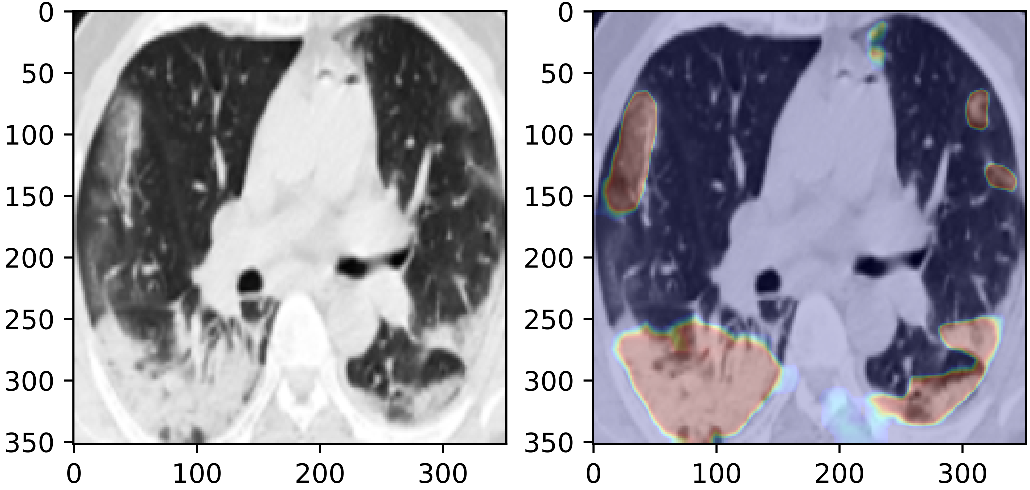

Lesion Proportion: 33.10%

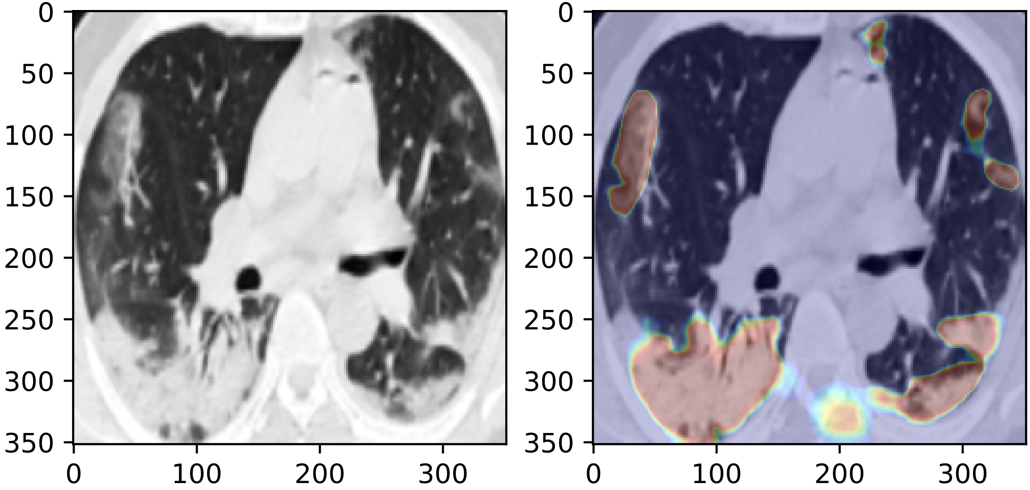

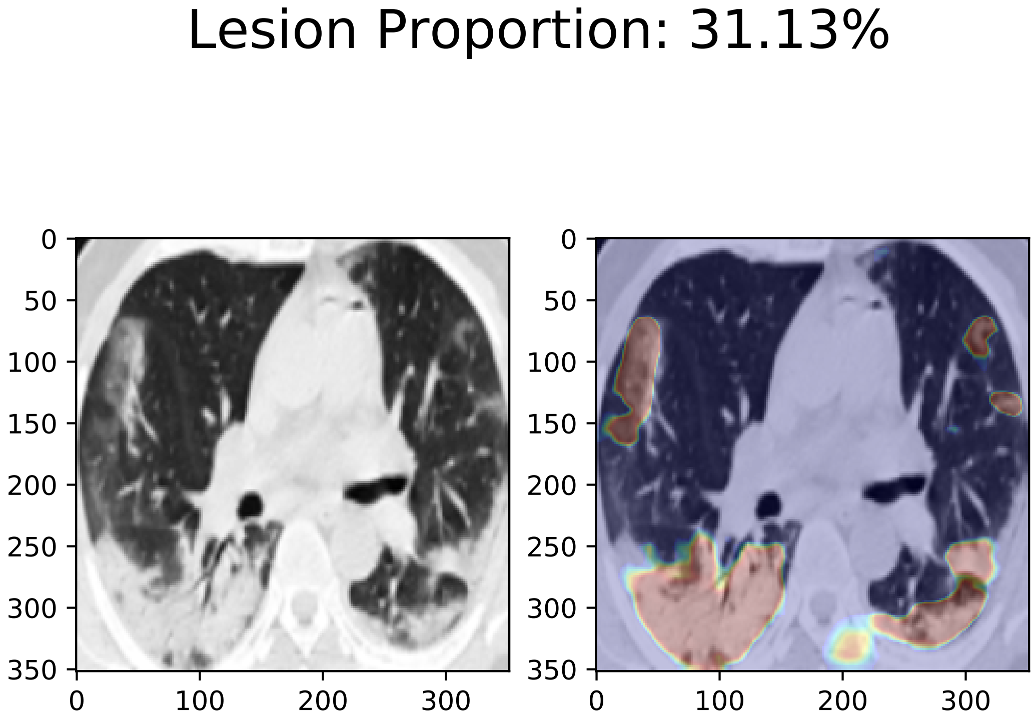
Lesion Proportion: 31.13%
0
50
100
150
200
250
300
350
0
100
200
300

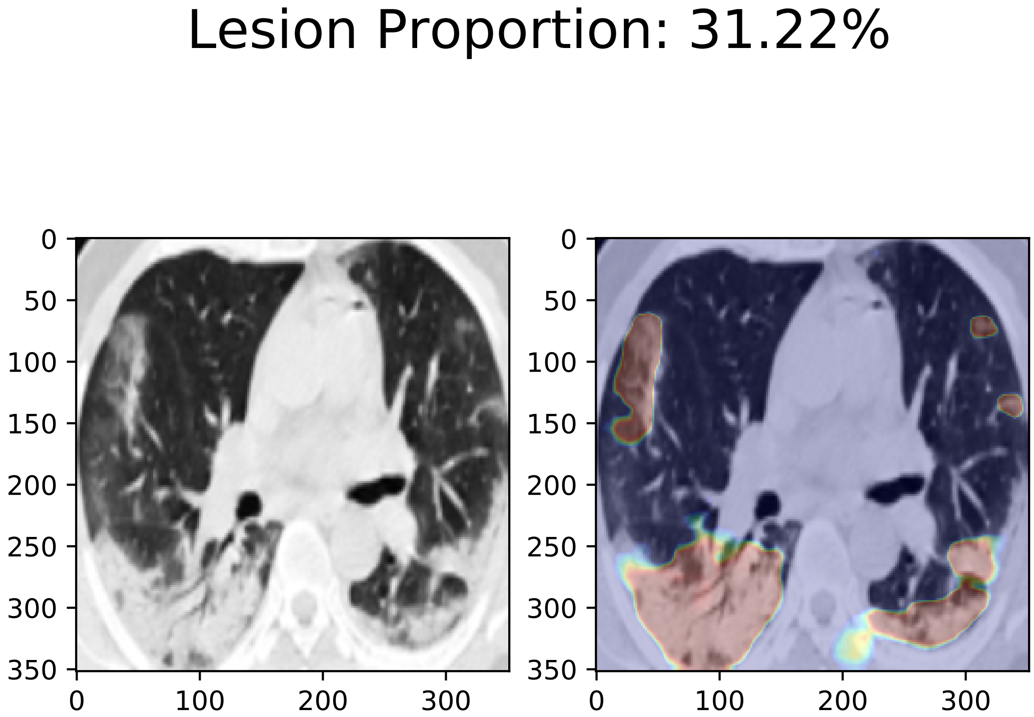
Lesion Proportion: 31.22%
0
50
100
150
200
250
300
350
0
100
200
300
0
50
100
150
200
250
300
350
0
100
200
300

Lesion Proportion: 32.80%

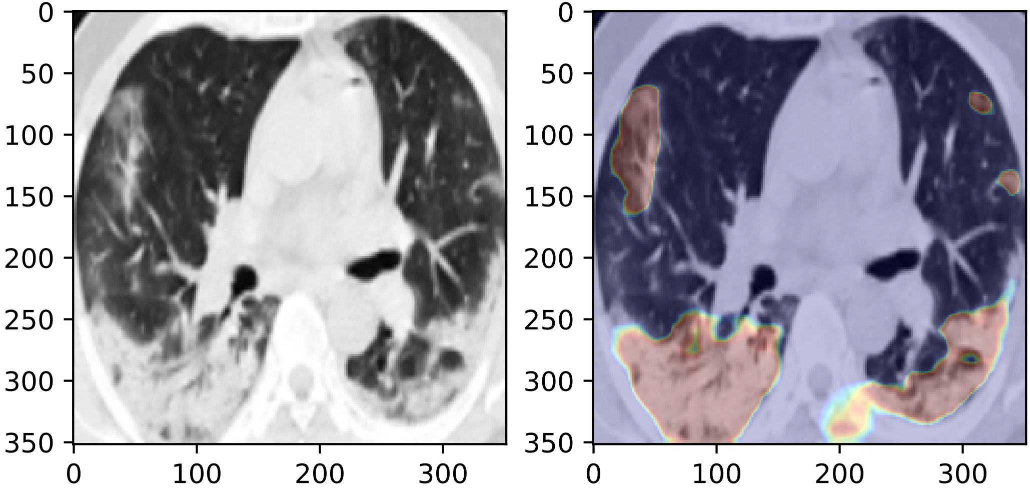

Lesion Proportion: 29.55%

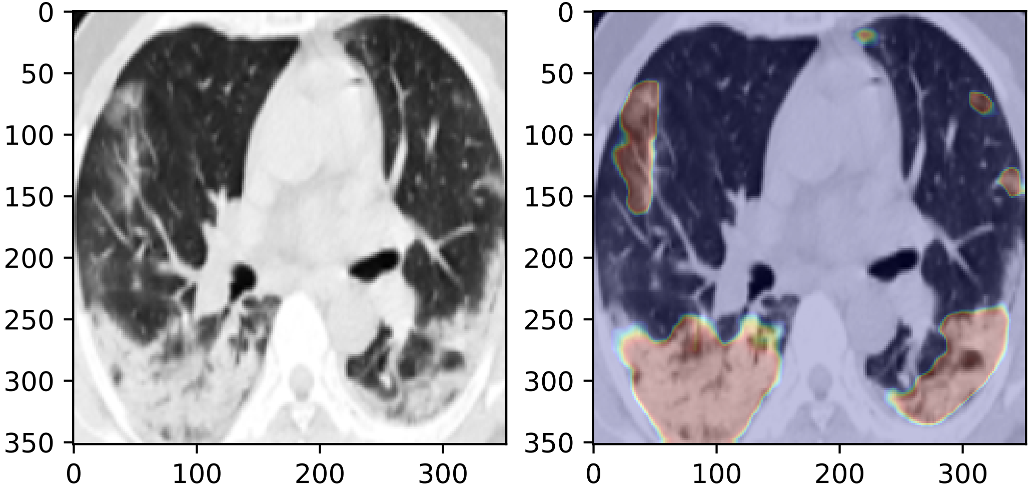

Lesion Proportion: 29.56%

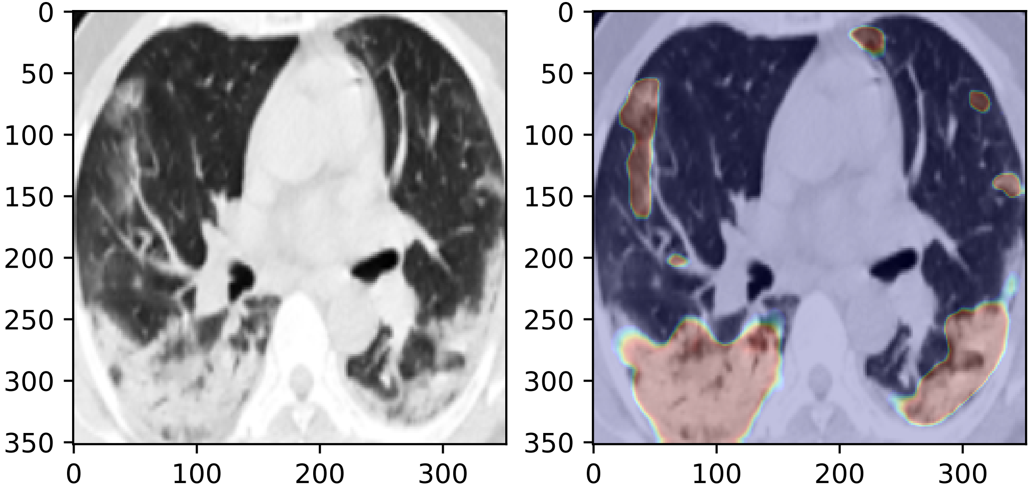

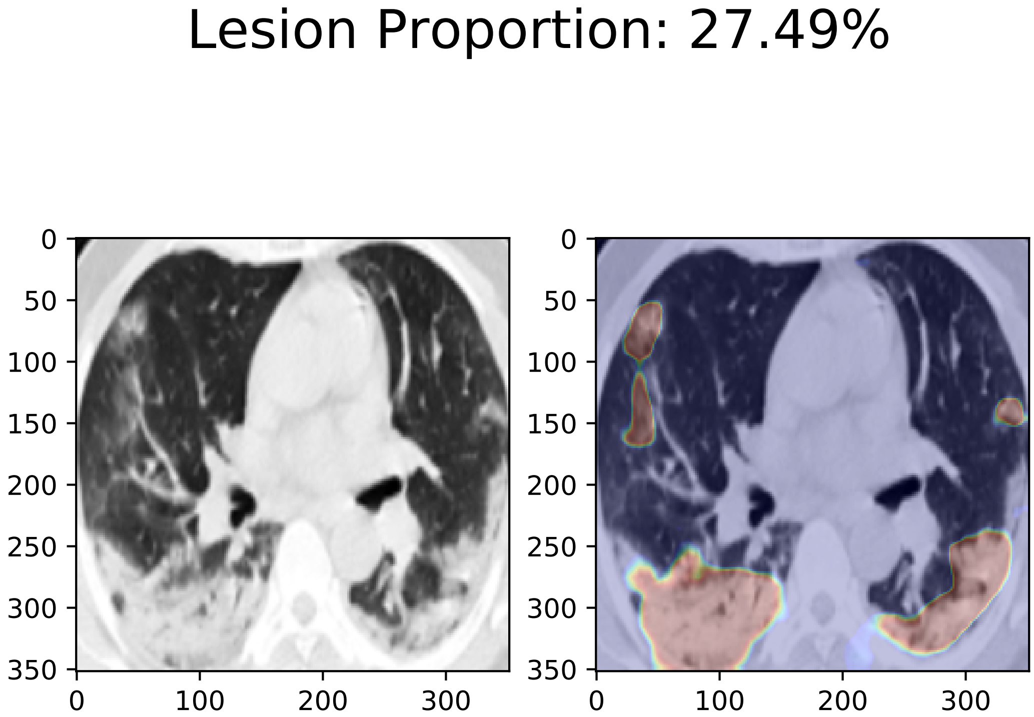
Lesion Proportion: 27.49%
0
50
100
150
200
250
300
350
0
100
200
300
0
50
100
150
200
250
300
350
0
100
200
300

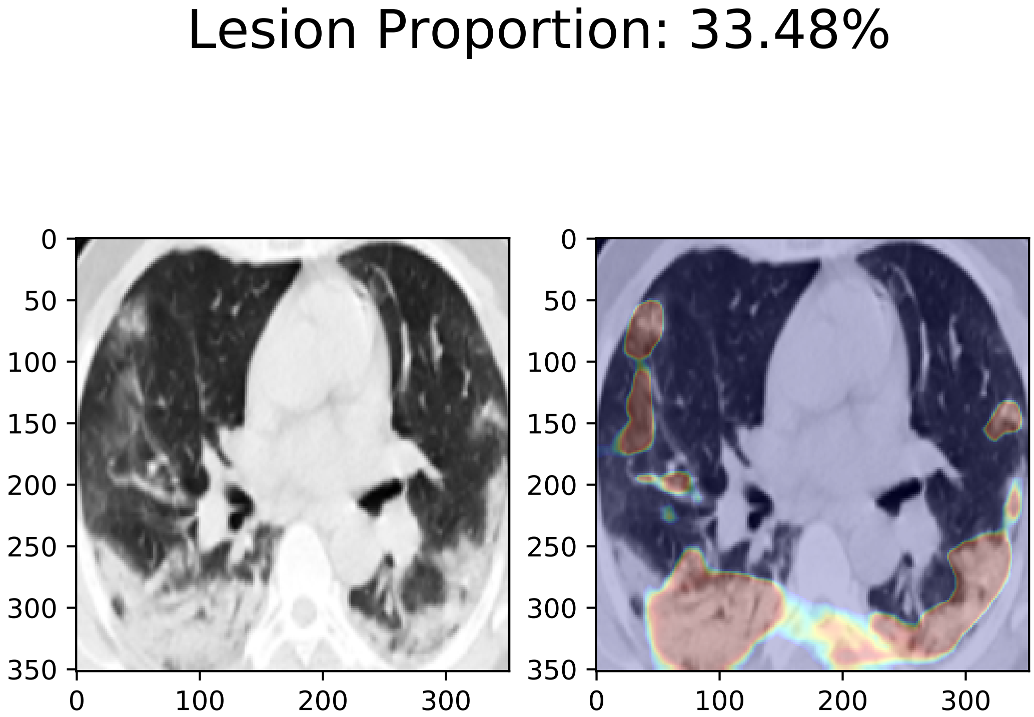
Lesion Proportion: 33.48%
0
50
100
150
200
250
300
350
0
100
200
300
0
50
100
150
200
250
300
350
0
100
200
300

Lesion Proportion: 34.11%

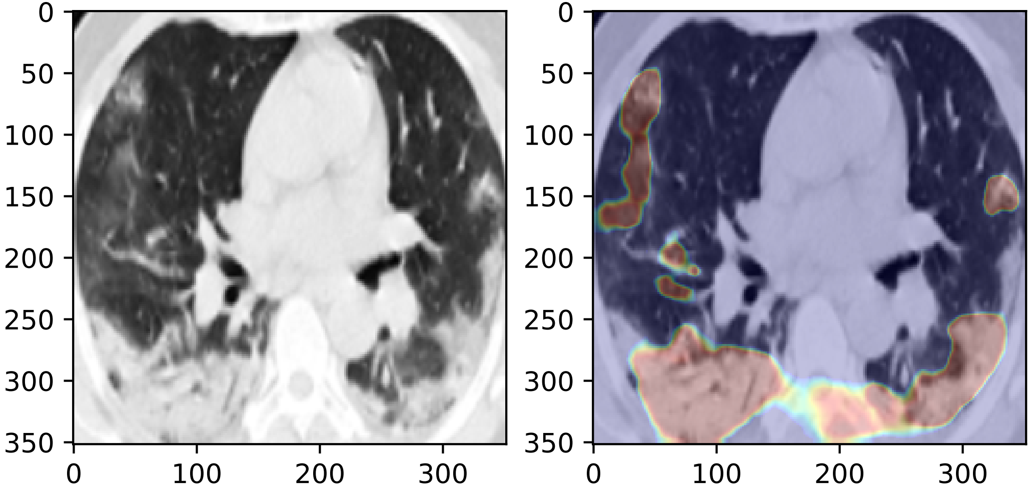

Lesion Proportion: 36.44%

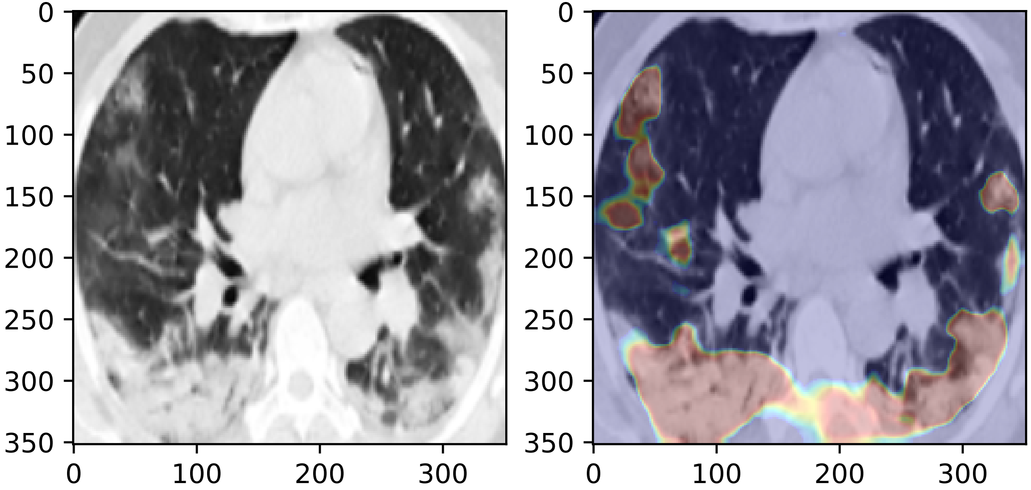

Lesion Proportion: 33.38%

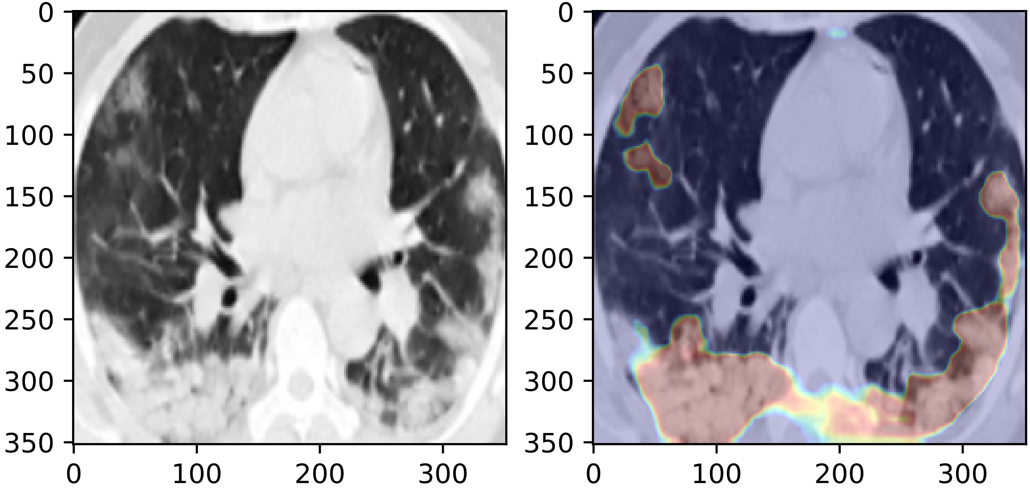

Lesion Proportion: 32.69%

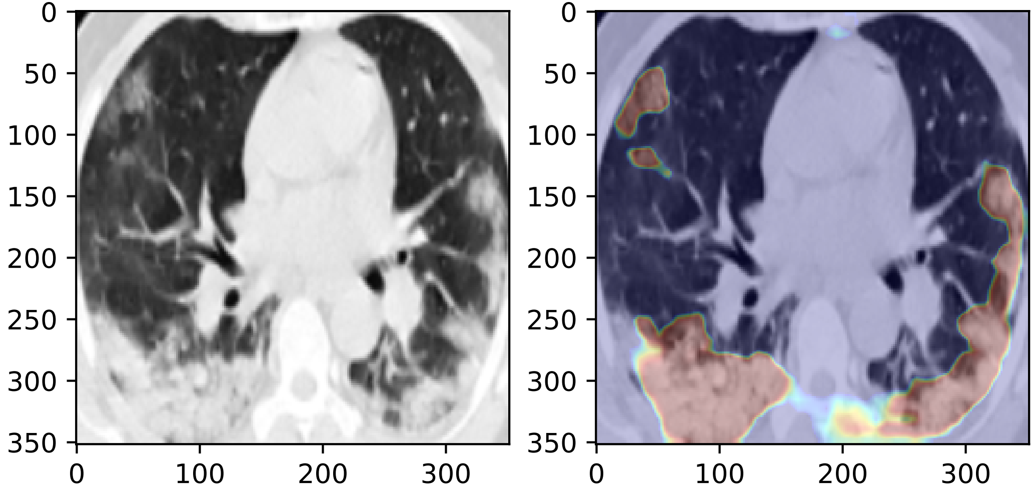

Lesion Proportion: 33.36%

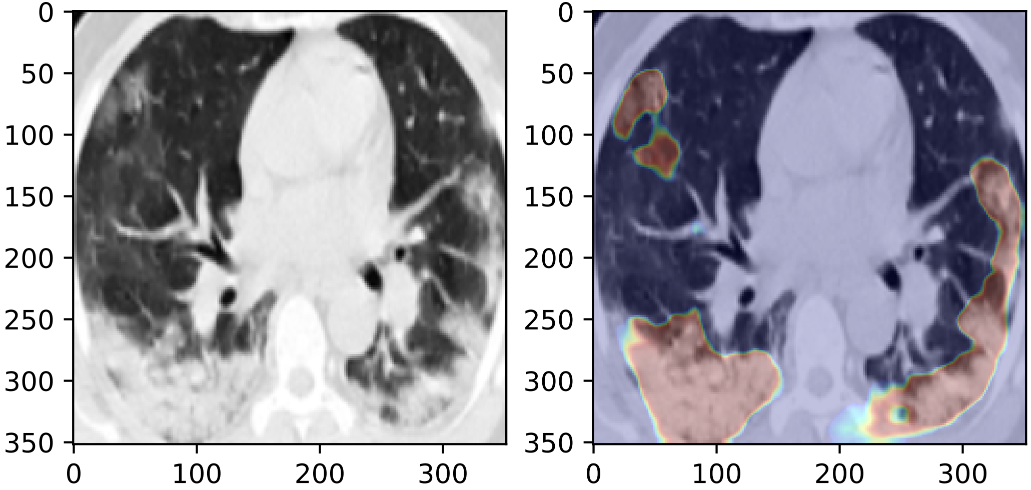

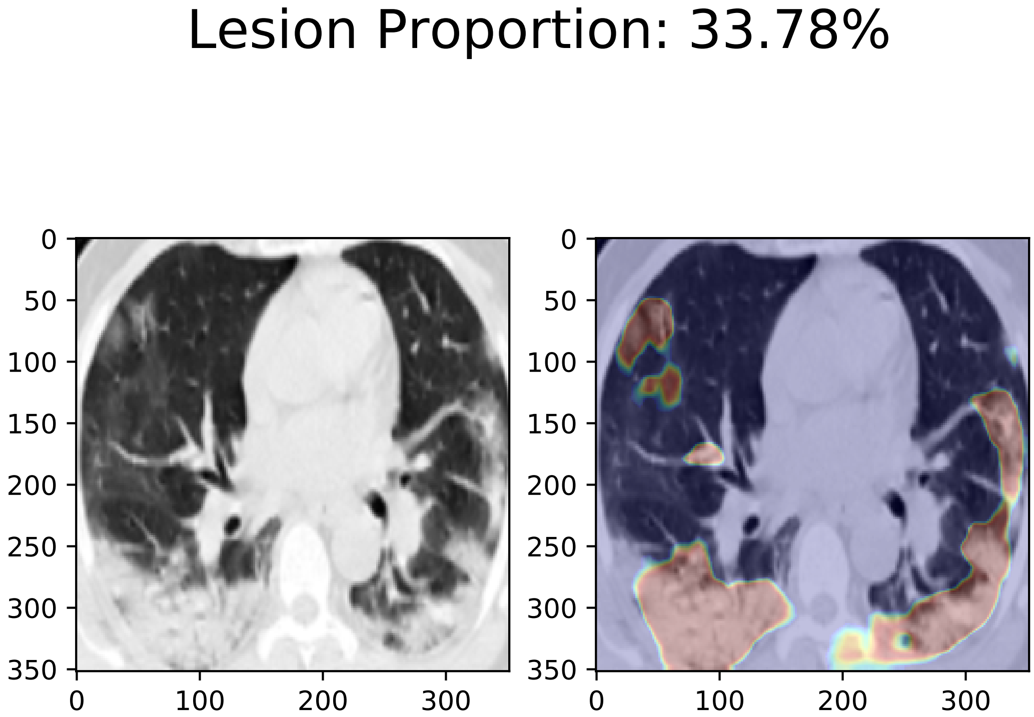
Lesion Proportion: 33.78%
0
50
100
150
200
250
300
350
0
100
200
300

Lesion Proportion: 32.17%

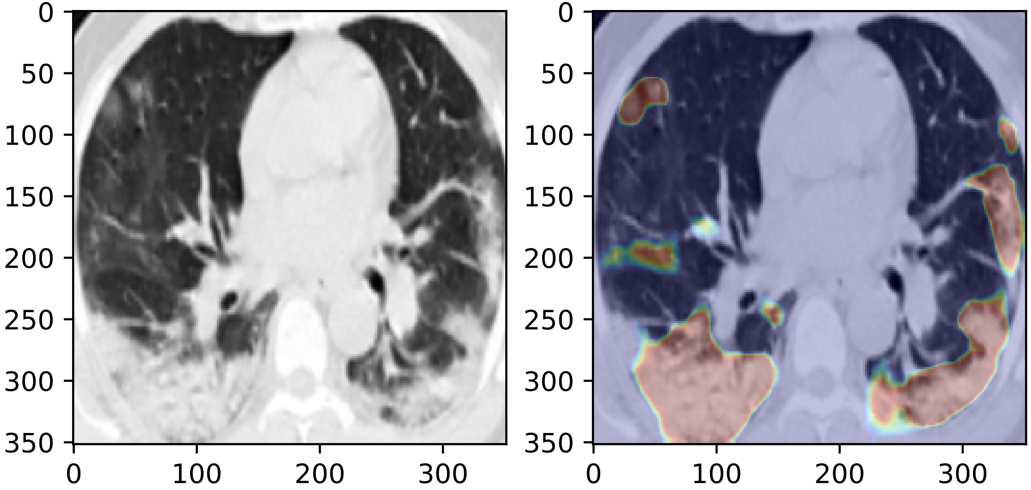

Lesion Proportion: 37.23%

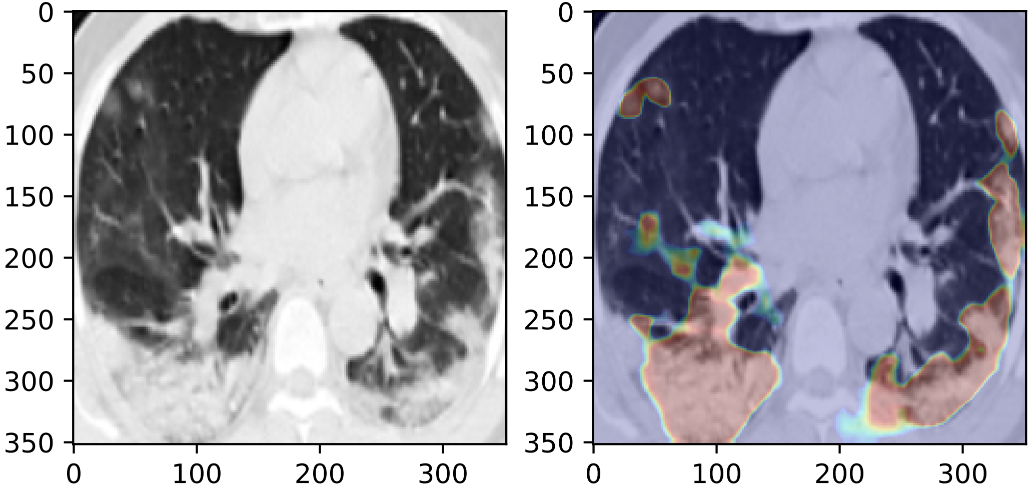

Lesion Proportion: 39.09%

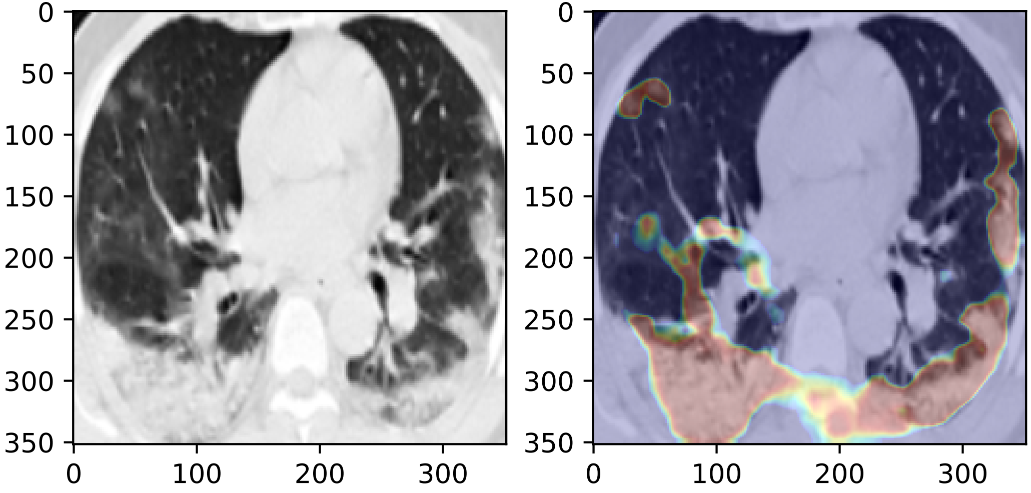

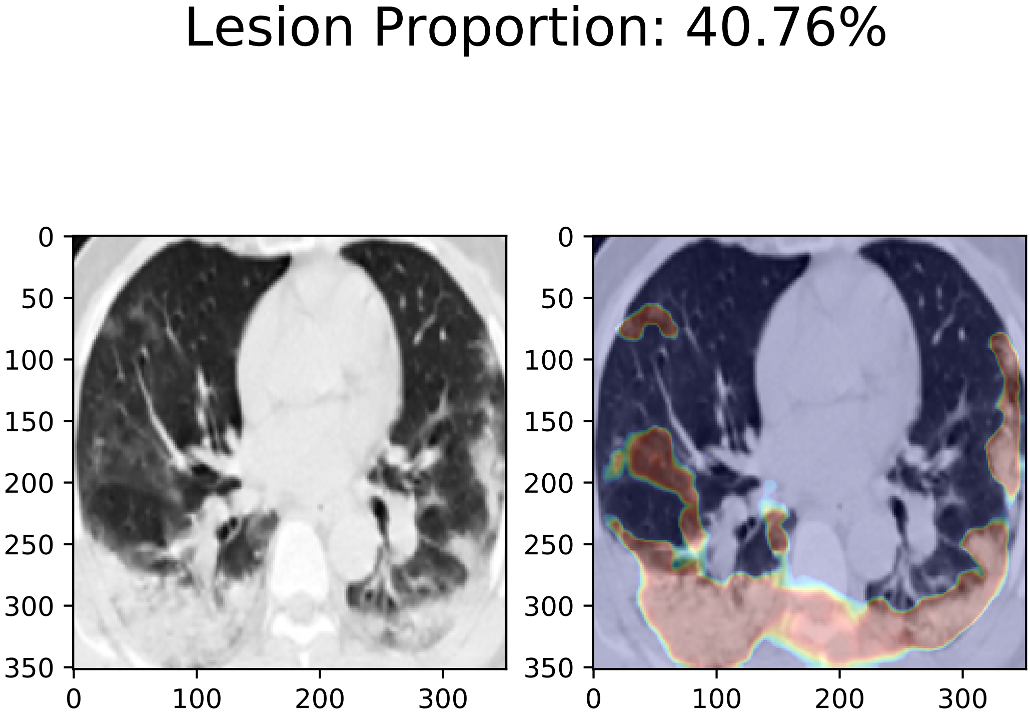
Lesion Proportion: 40.76%
0
50
100
150
200
250
300
350
0
100
200
300

Lesion Proportion: 35.20%

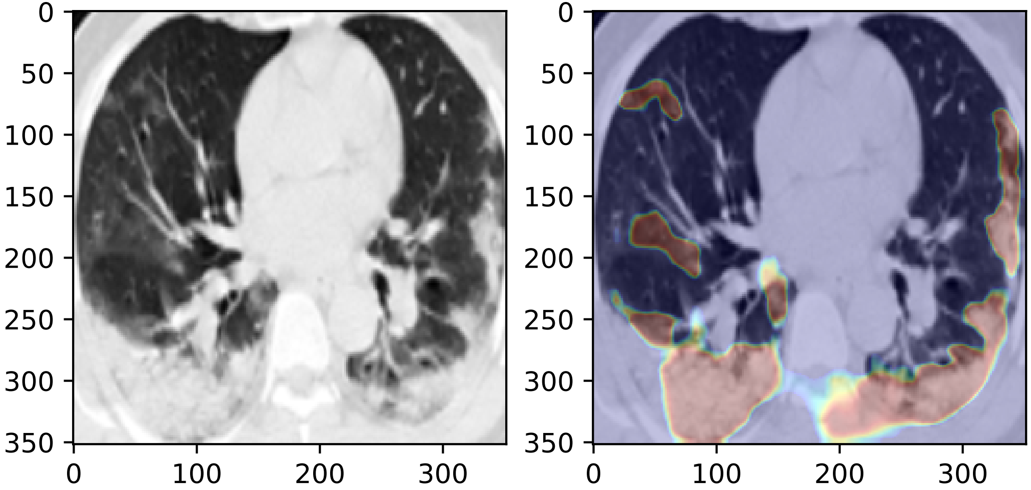

Lesion Proportion: 37.78%

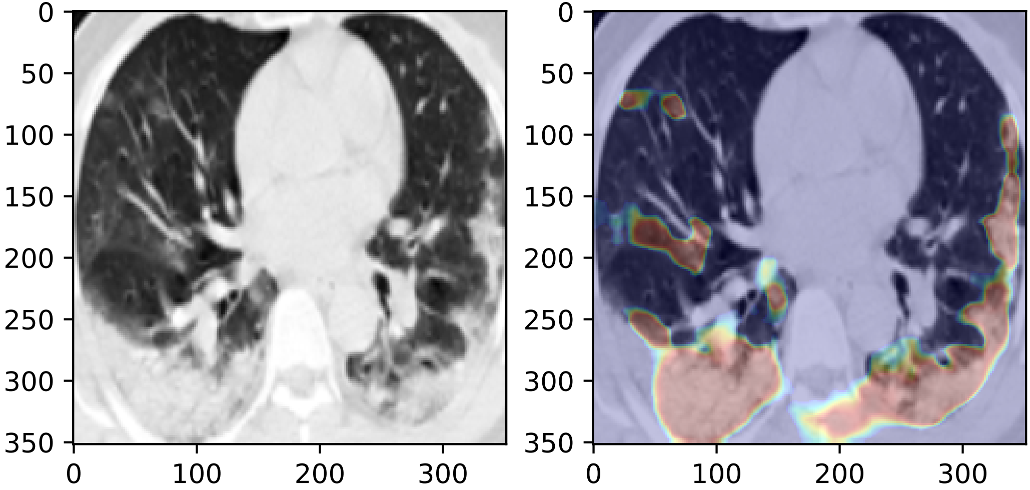

Lesion Proportion: 34.49%

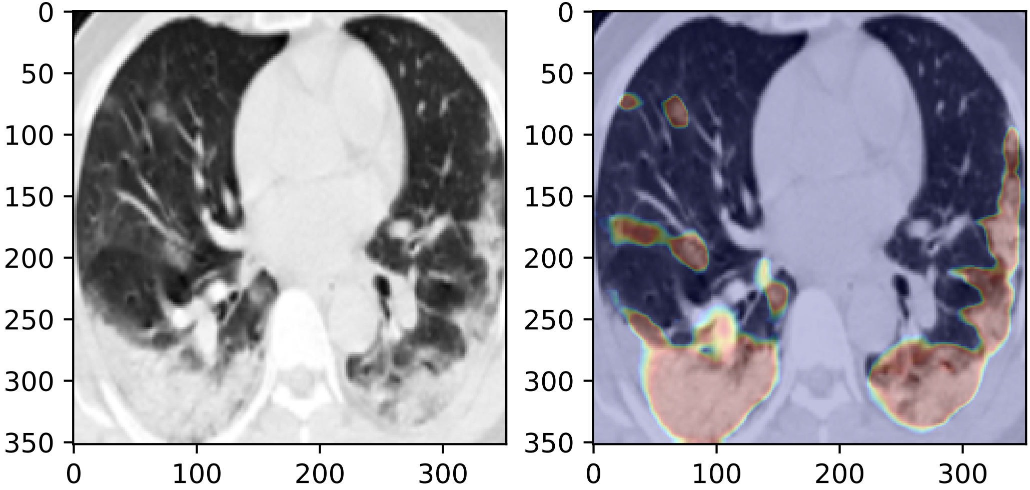

Lesion Proportion: 33.08%

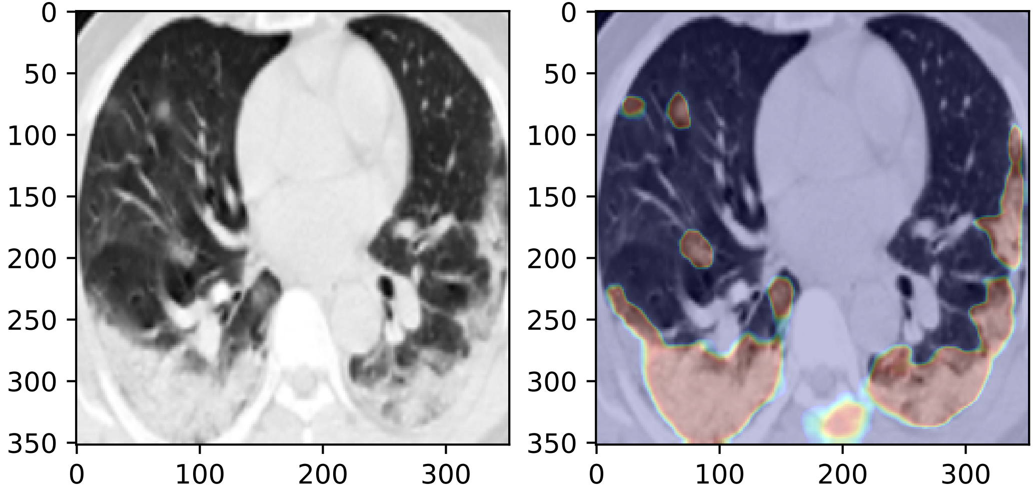

Lesion Proportion: 35.13%

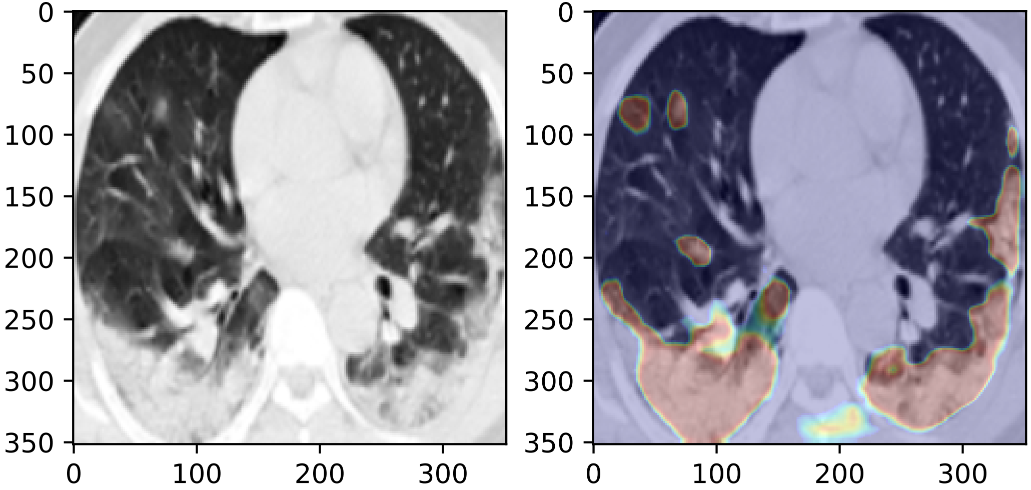

Lesion Proportion: 33.15%

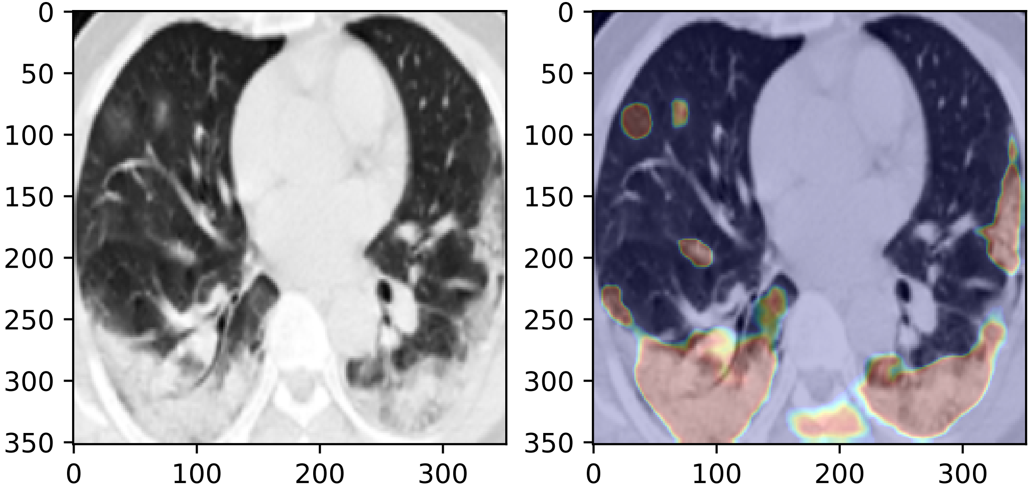

Lesion Proportion: 33.19%

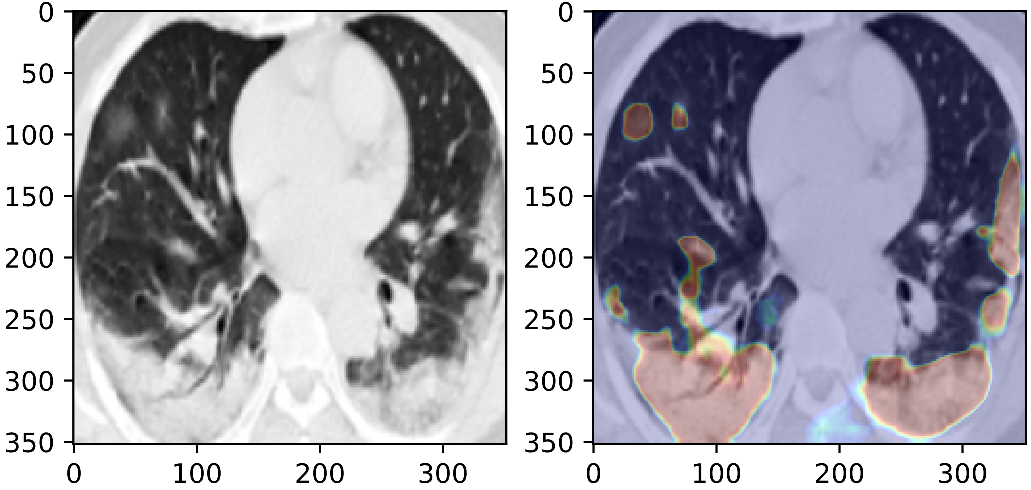

Lesion Proportion: 32.00%

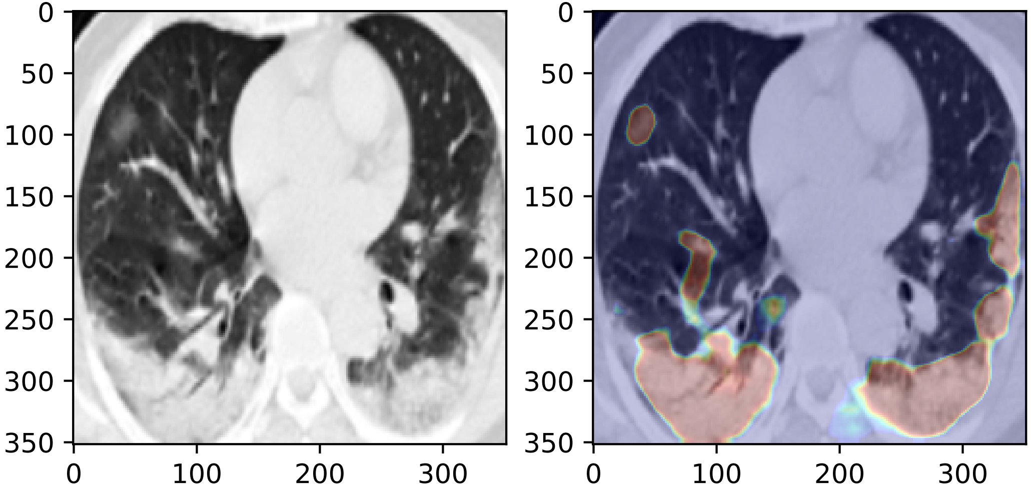

Lesion Proportion: 30.85%

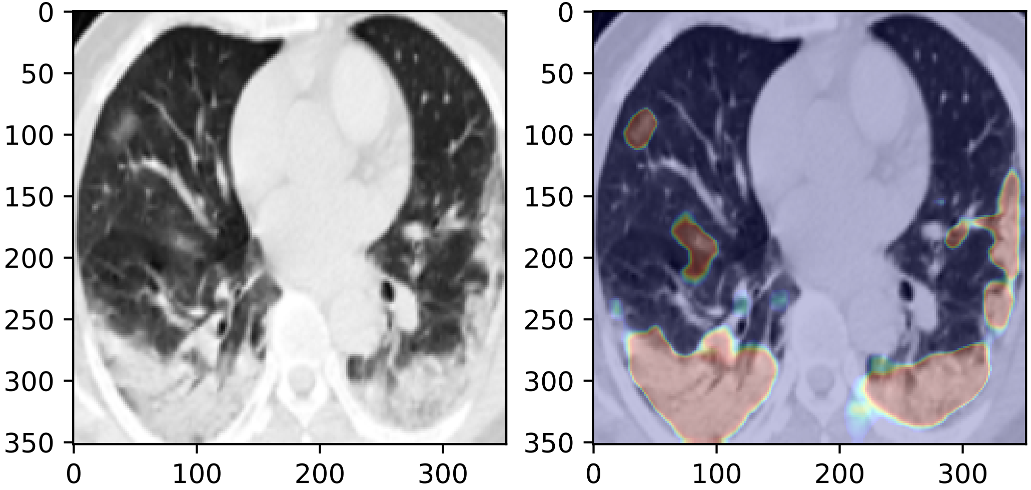

Lesion Proportion: 34.62%

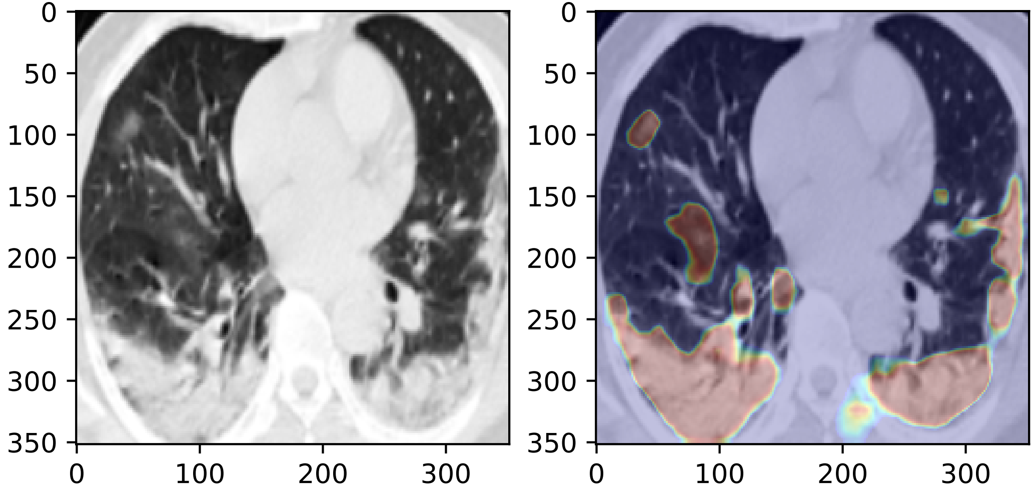

Lesion Proportion: 36.74%

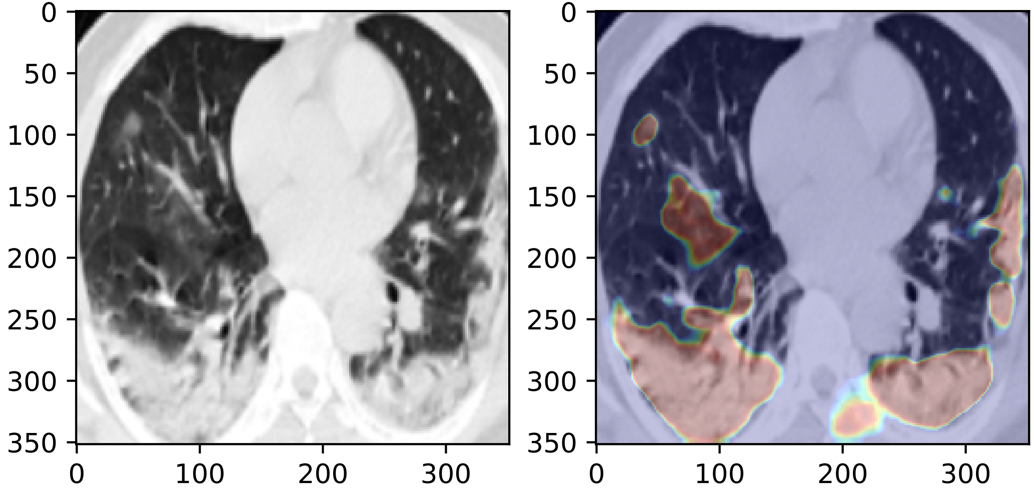

Lesion Proportion: 34.68%

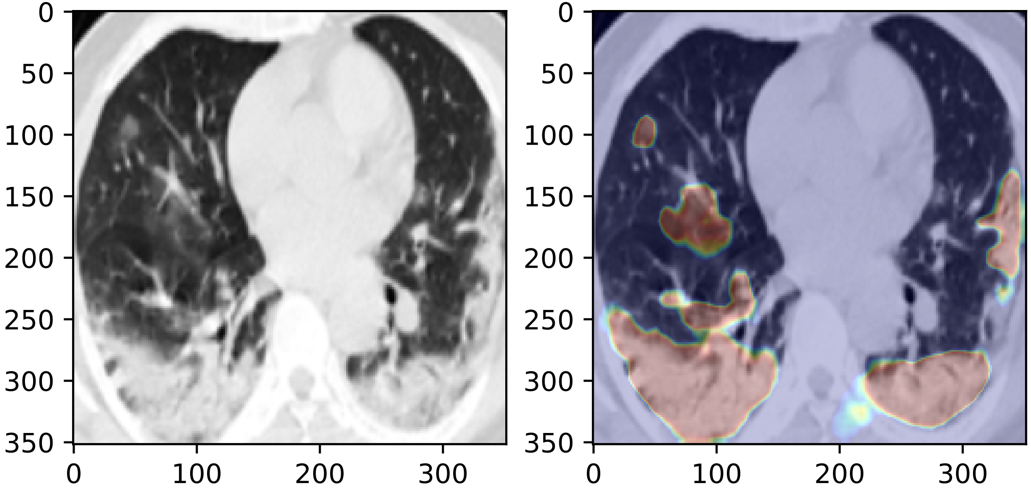

Lesion Proportion: 33.45%

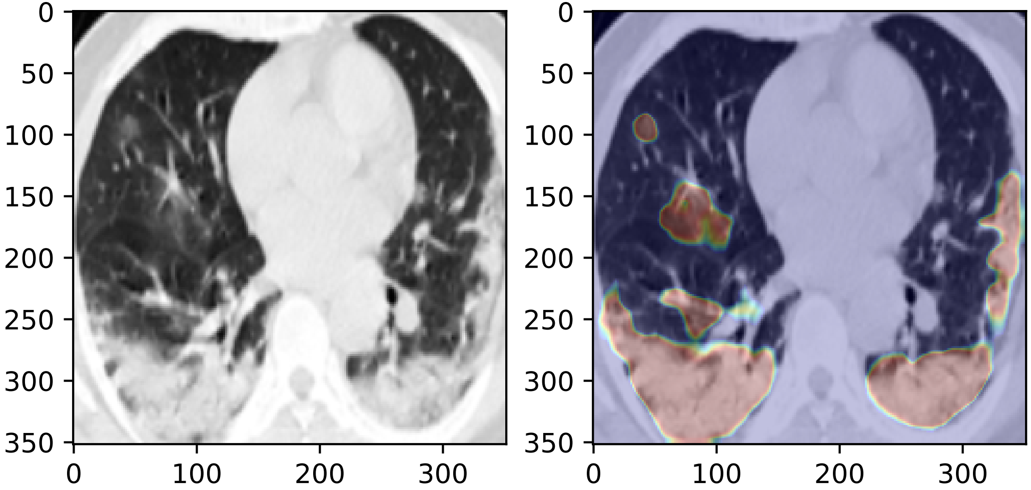

Lesion Proportion: 34.47%

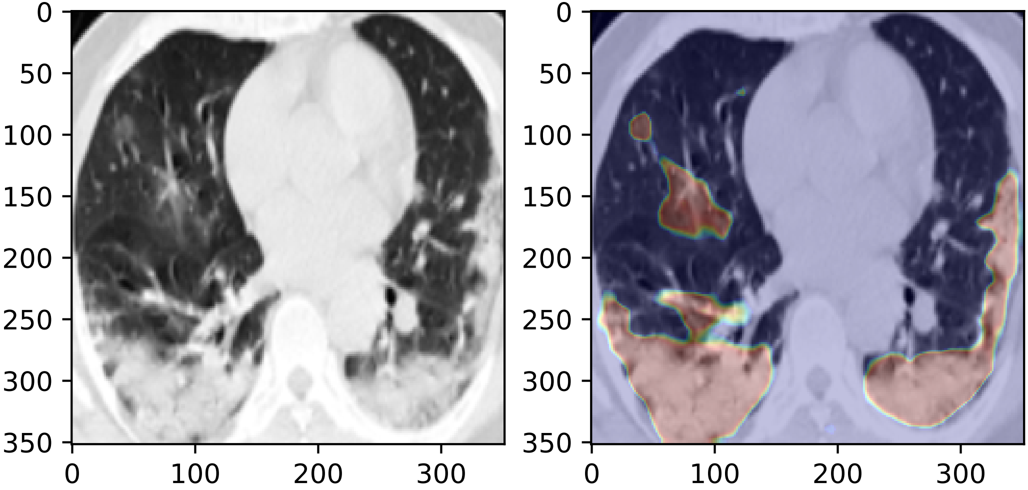

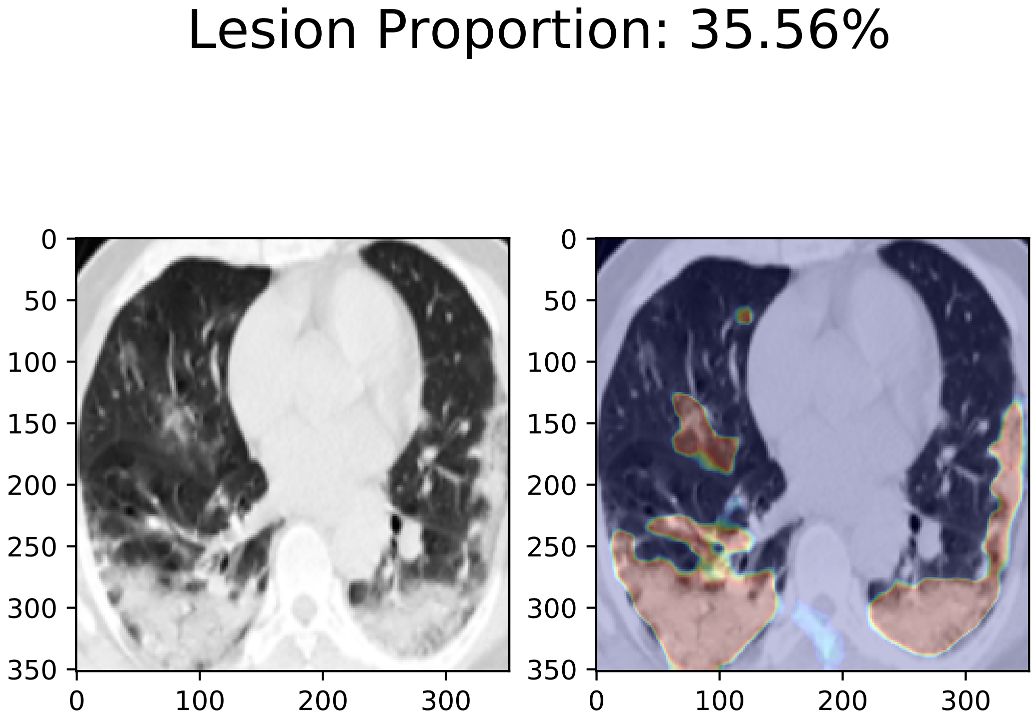
Lesion Proportion: 35.56%
0
50
100
150
200
250
300
350
0
100
200
300

Lesion Proportion: 35.93%

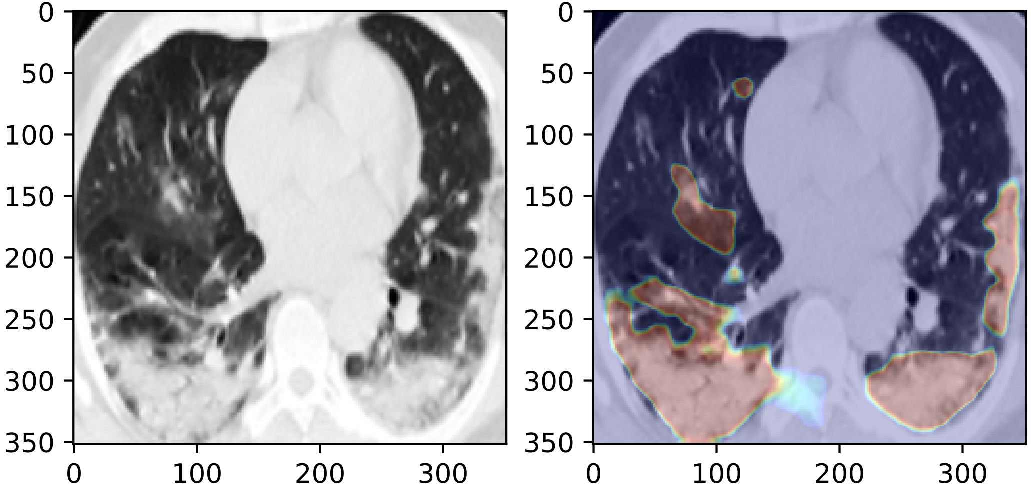

Lesion Proportion: 37.14%

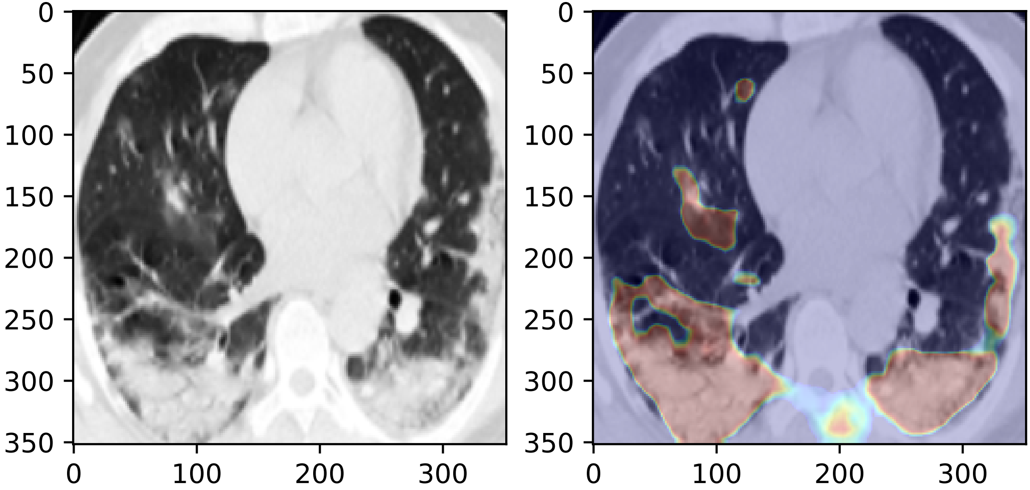

Lesion Proportion: 36.90%

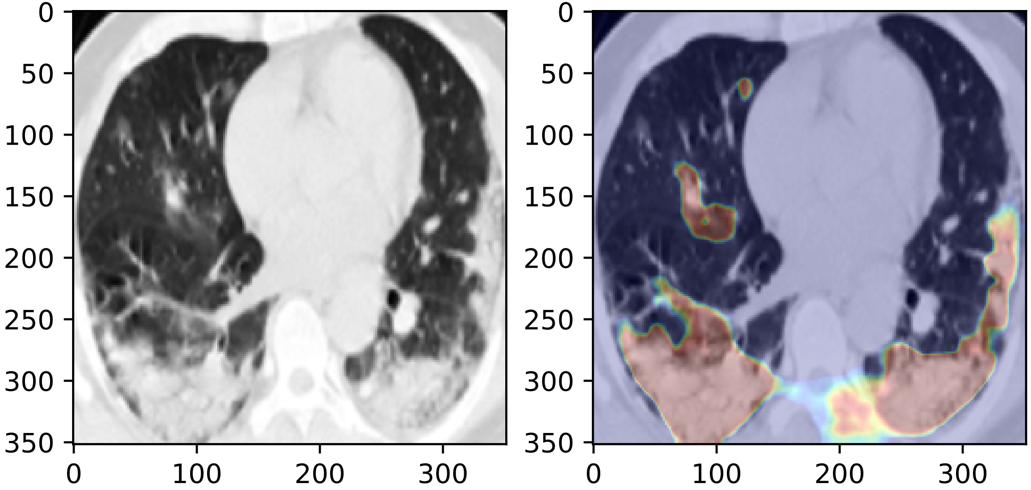

Lesion Proportion: 32.95%

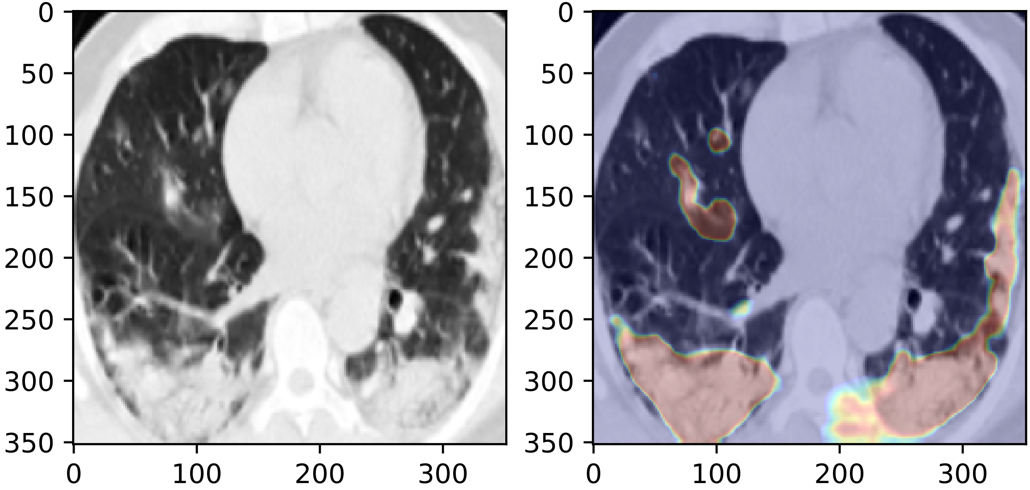

Lesion Proportion: 32.65%

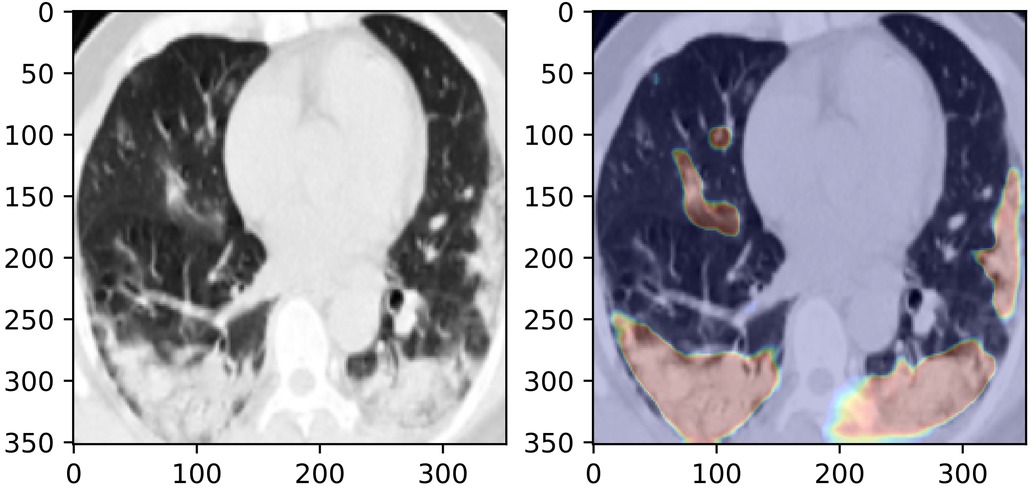

Lesion Proportion: 32.77%

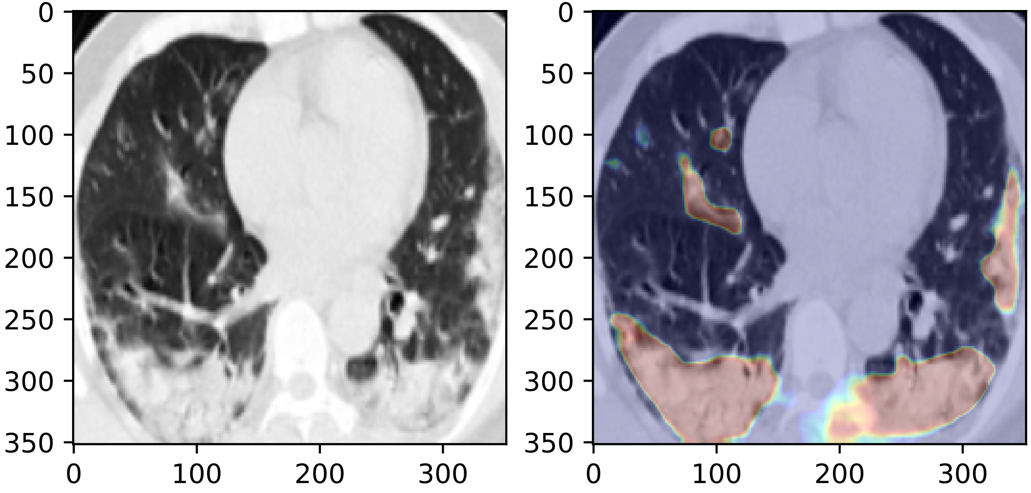

Lesion Proportion: 29.39%

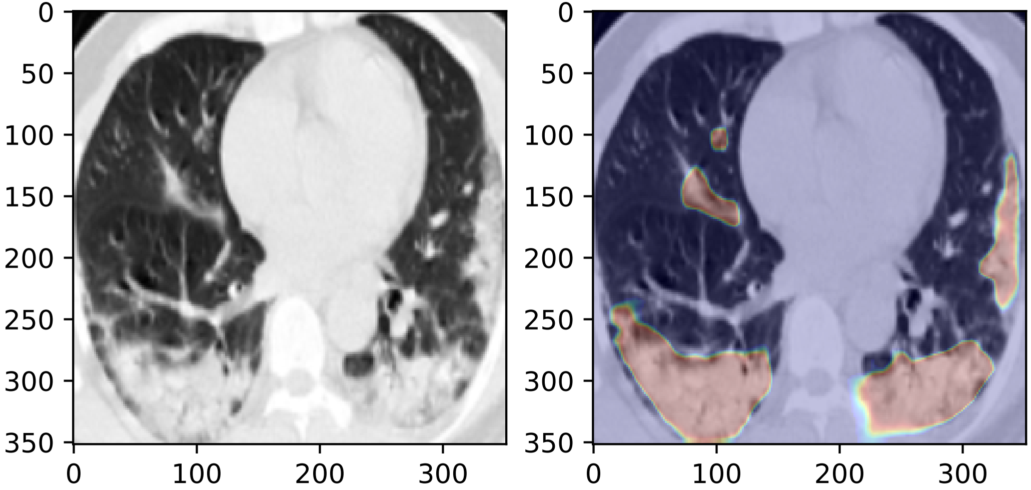

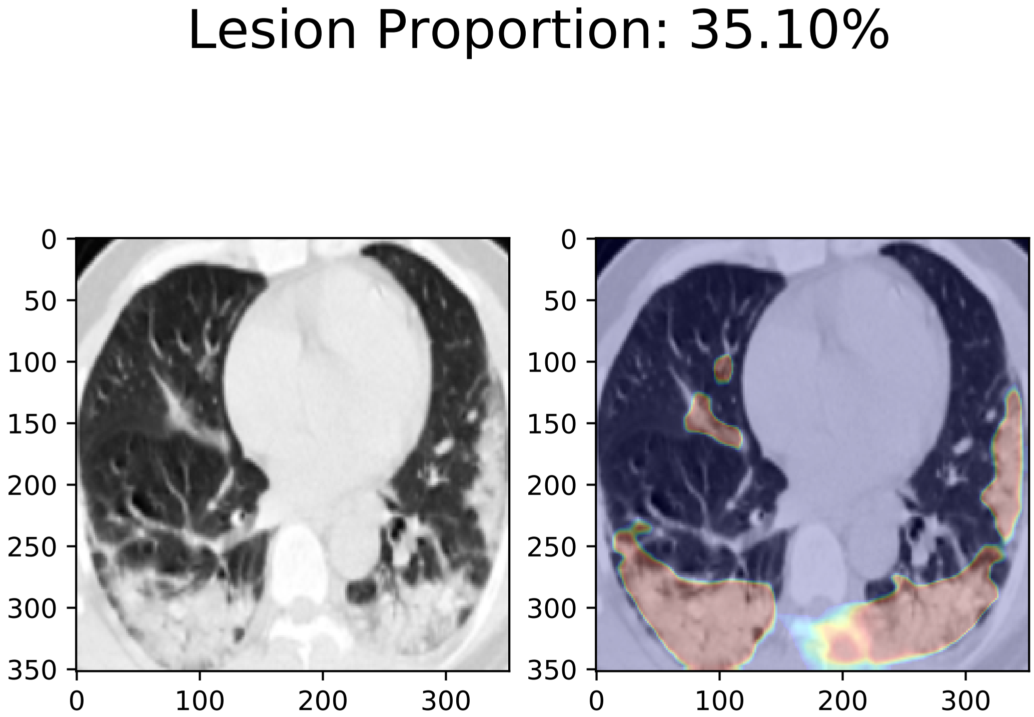
Lesion Proportion: 35.10%
0
50
100
150
200
250
300
350
0
100
200
300

Lesion Proportion: 39.19%

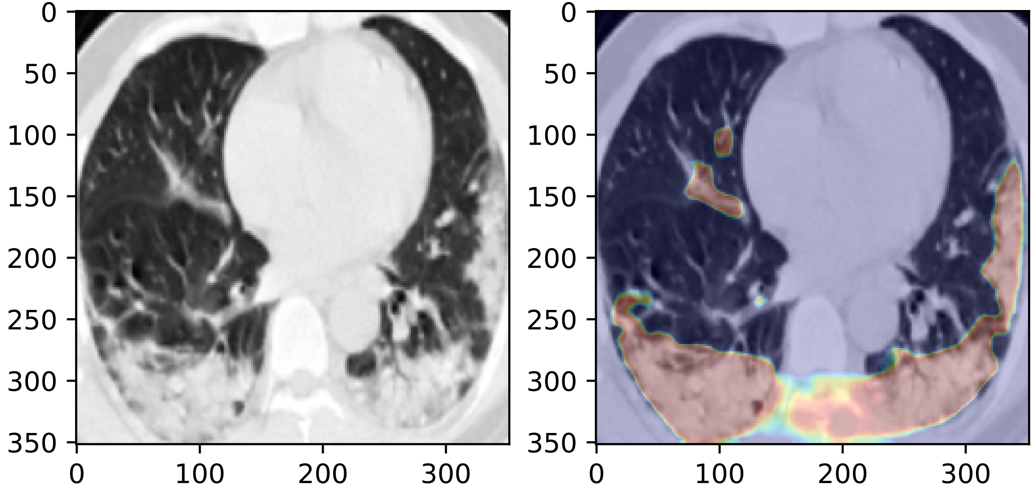

Lesion Proportion: 35.84%

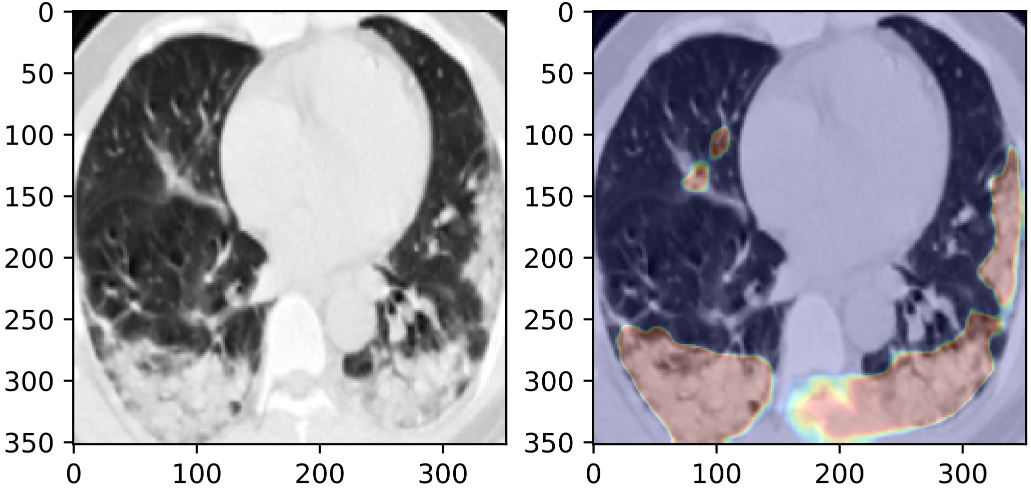

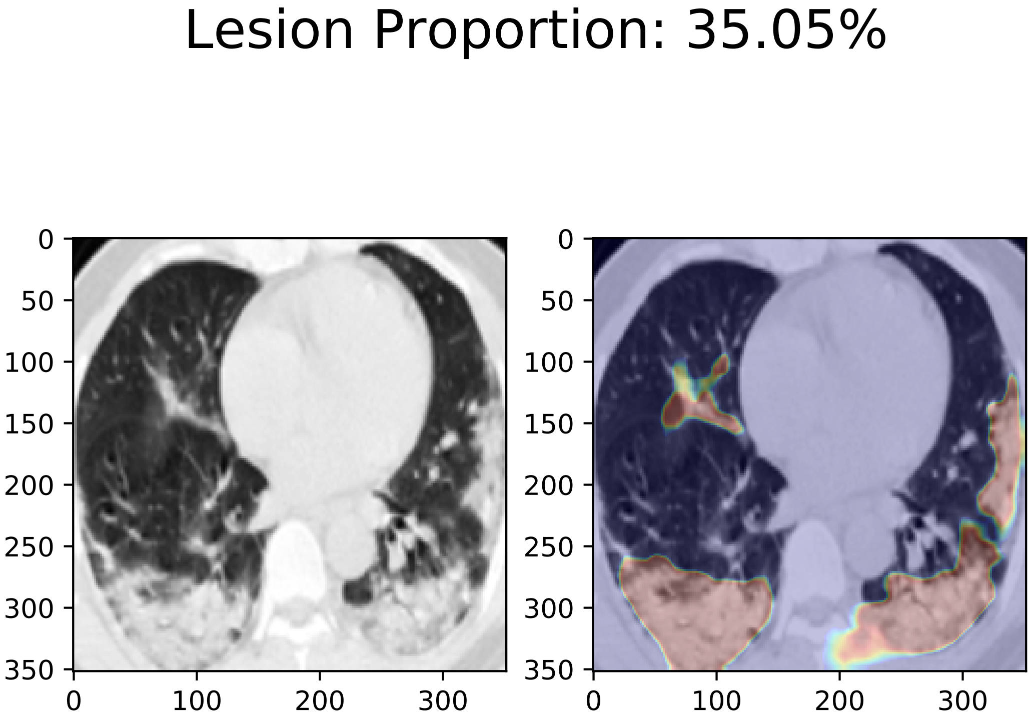
Lesion Proportion: 35.05%
0
50
100
150
200
250
300
350
0
100
200
300

Lesion Proportion: 33.03%

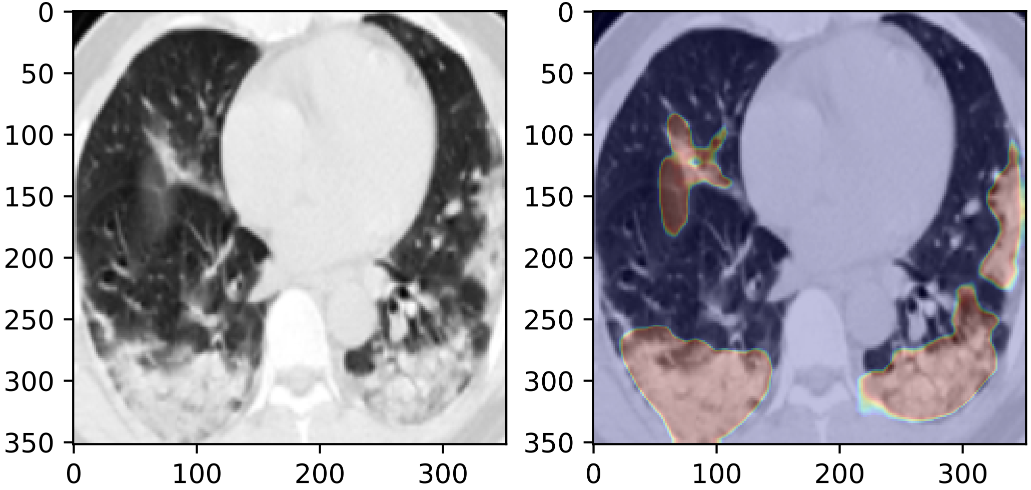

Lesion Proportion: 34.05%

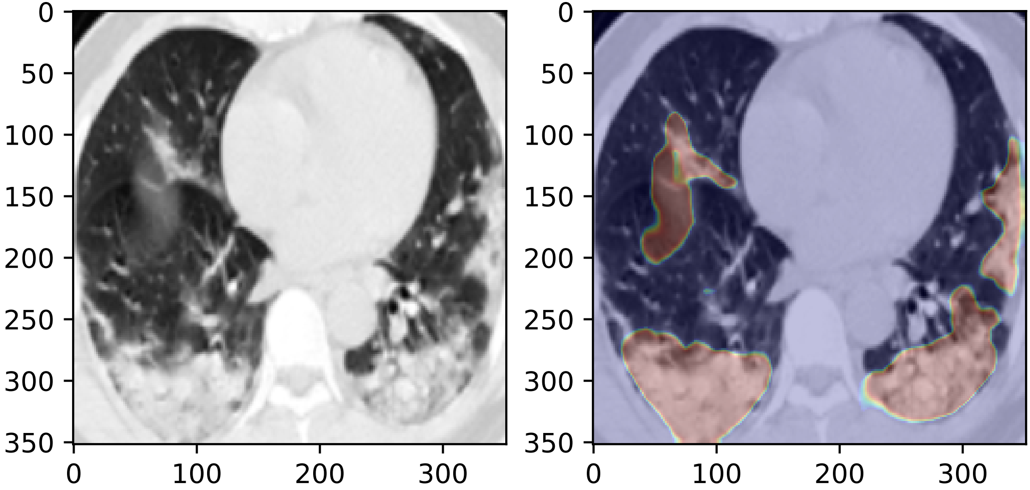

Lesion Proportion: 37.79%

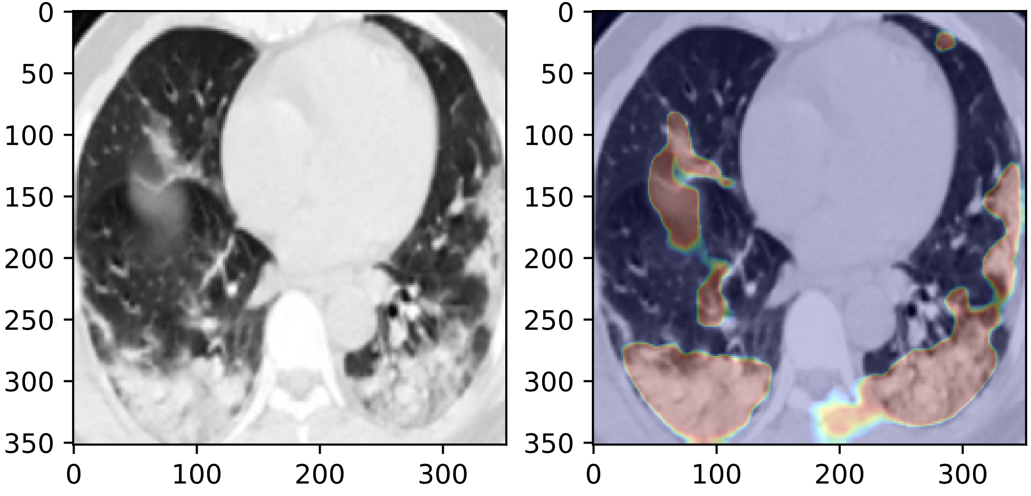

Lesion Proportion: 34.99%

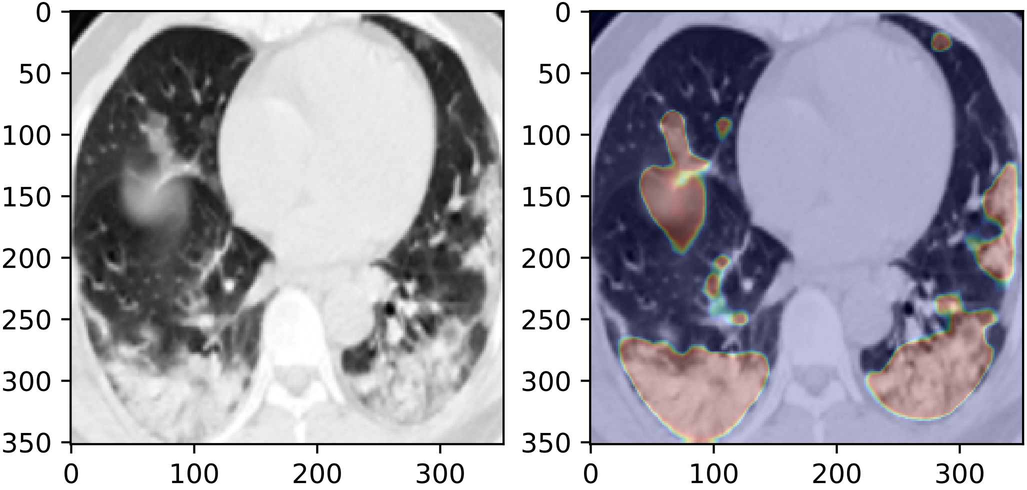

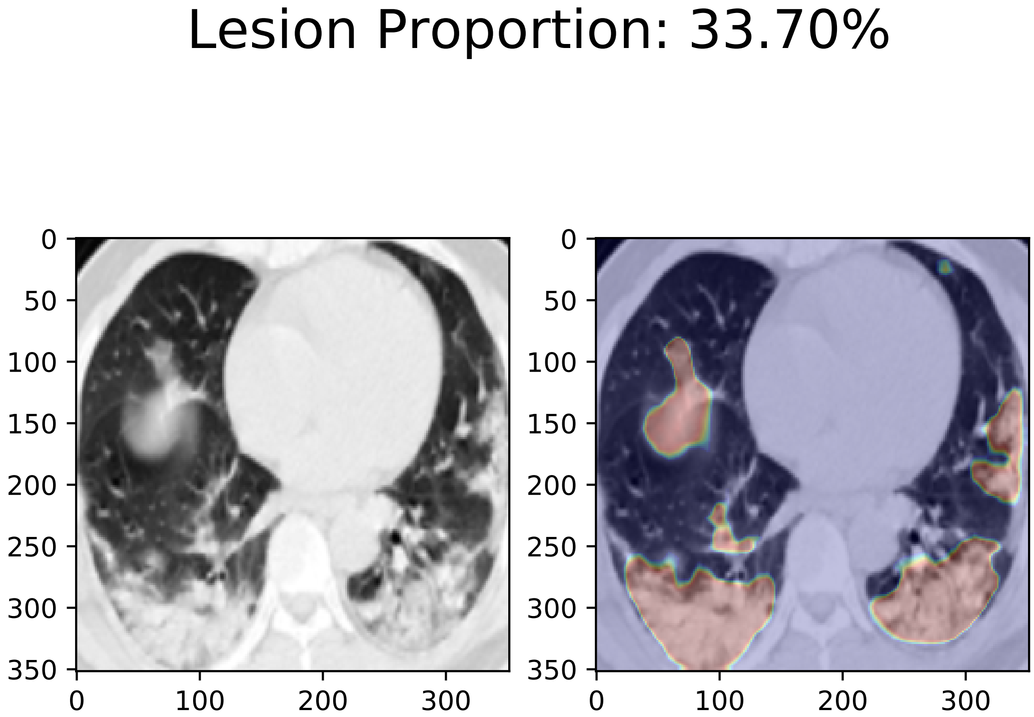
Lesion Proportion: 33.70%
0
50
100
150
200
250
300
350
0
100
200
300
0
50
100
150
200
250
300
350
0
100
200
300

Lesion Proportion: 33.76%

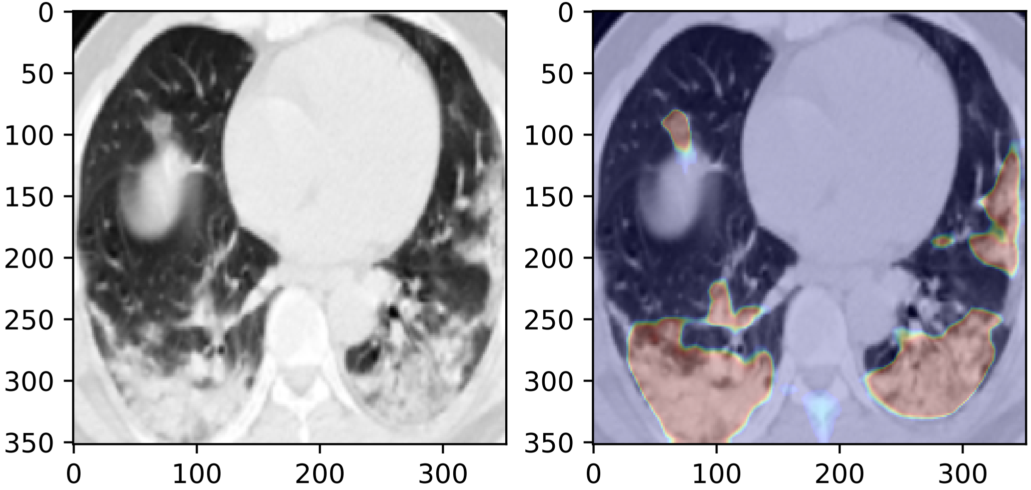

Lesion Proportion: 37.22%

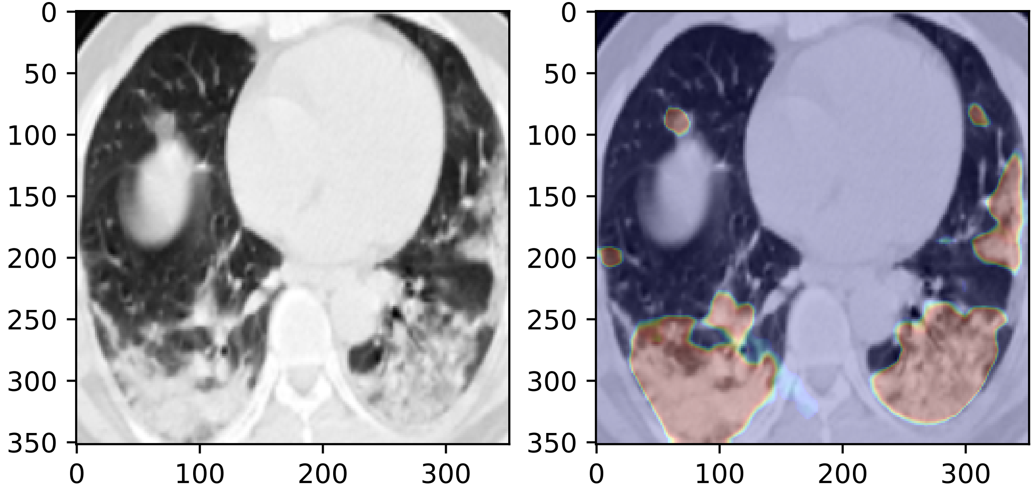

Lesion Proportion: 42.08%

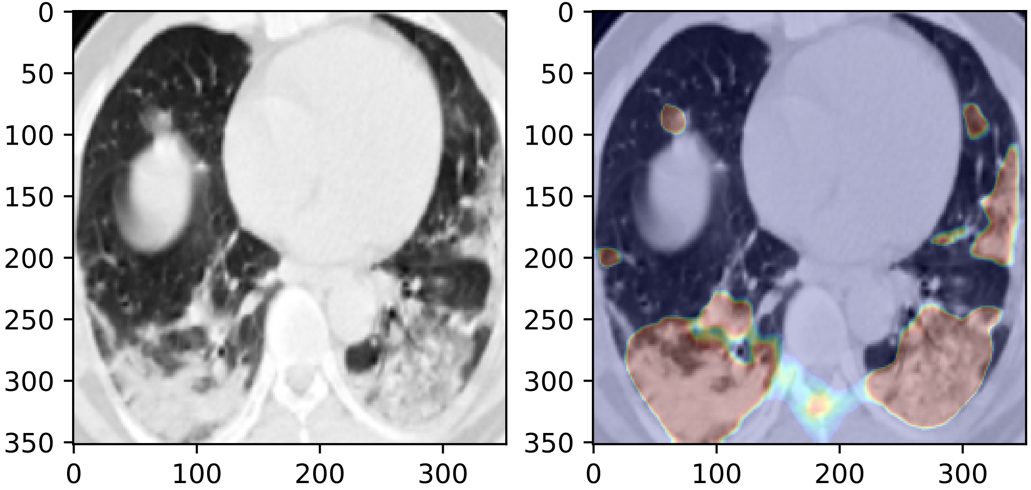

Lesion Proportion: 42.82%

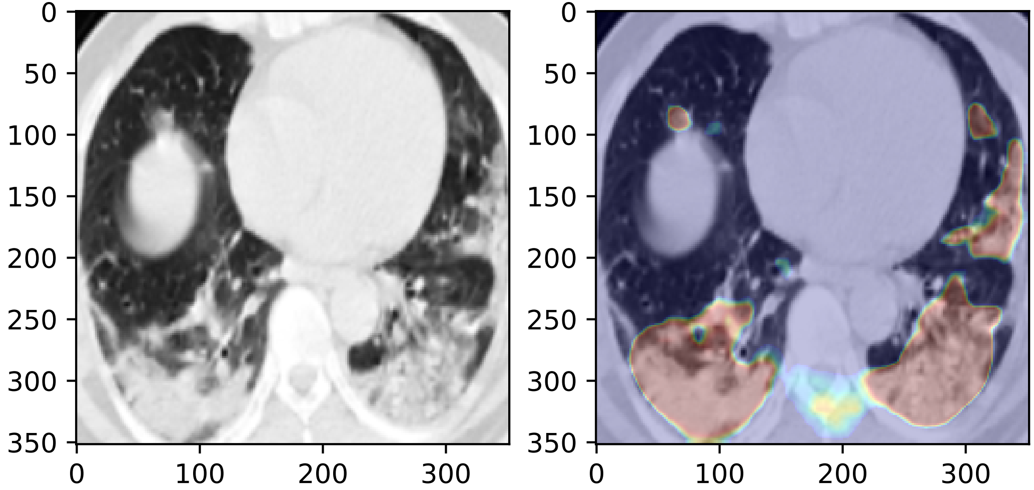

Lesion Proportion: 36.01%

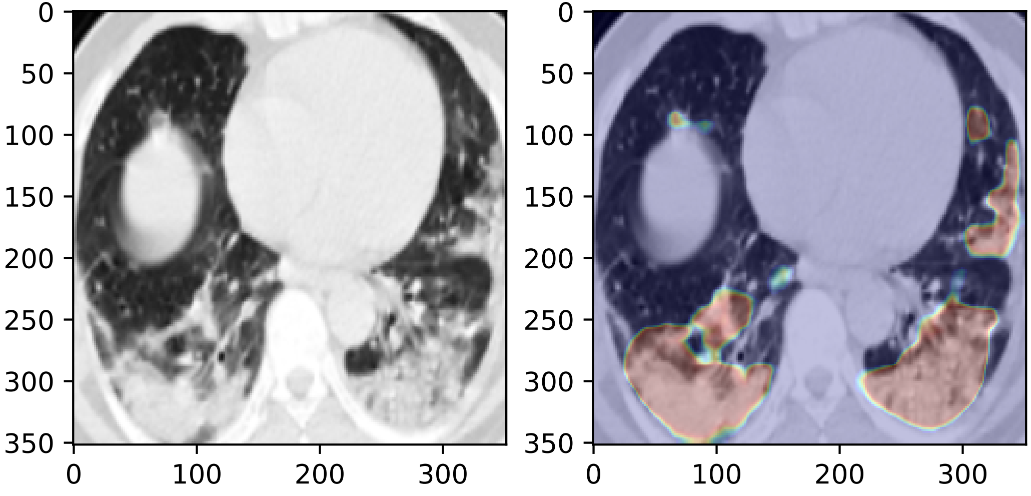

Lesion Proportion: 36.93%

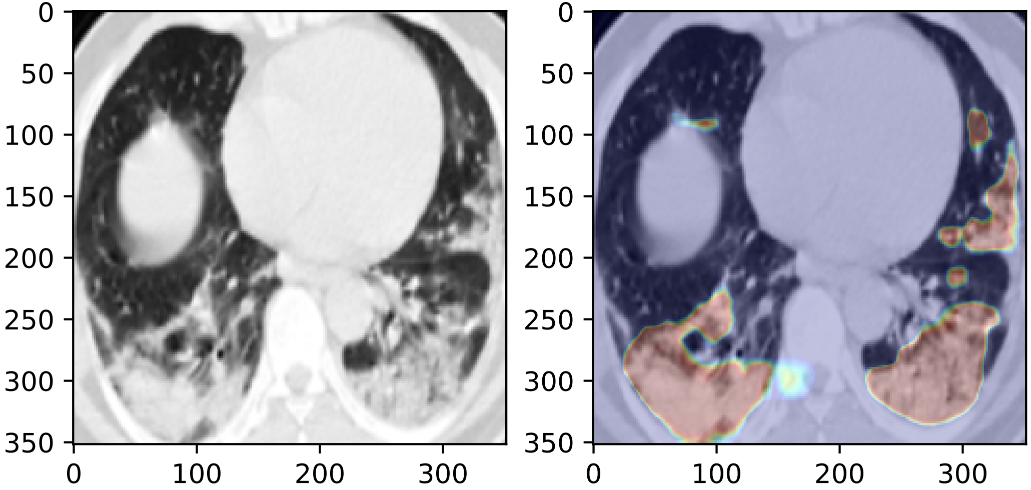

Lesion Proportion: 38.04%

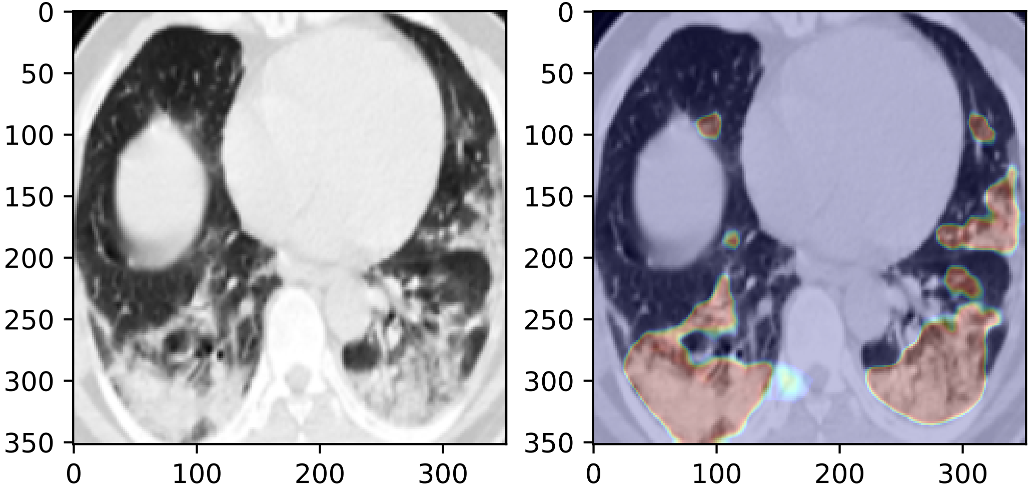

Lesion Proportion: 41.53%

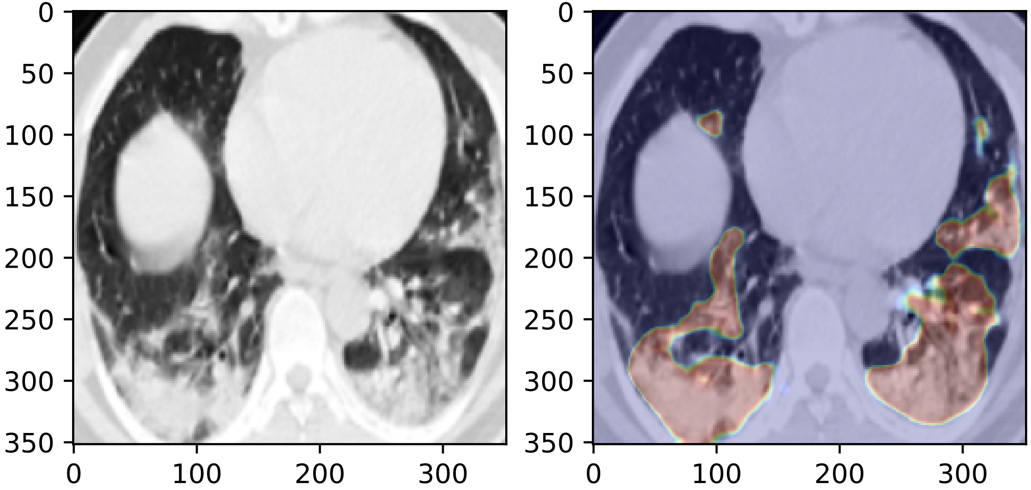

Lesion Proportion: 42.42%

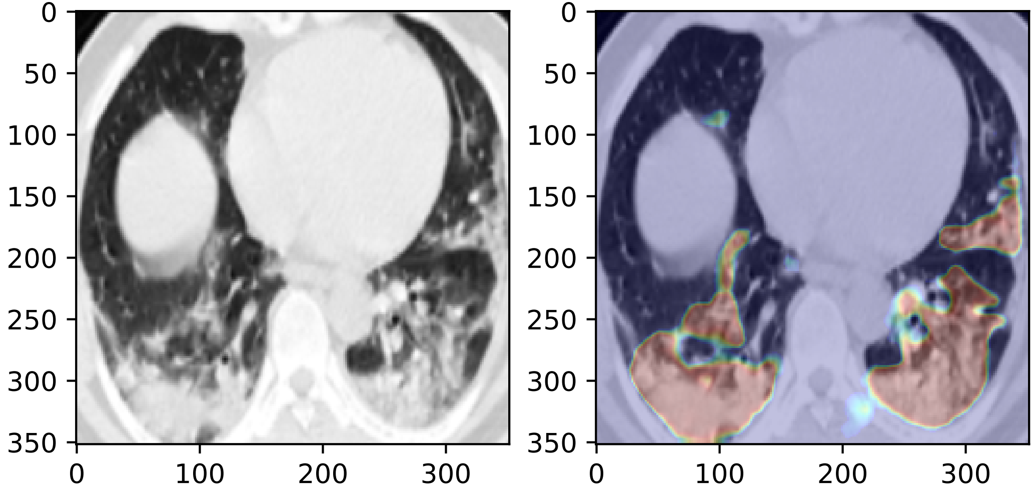

Lesion Proportion: 36.83%

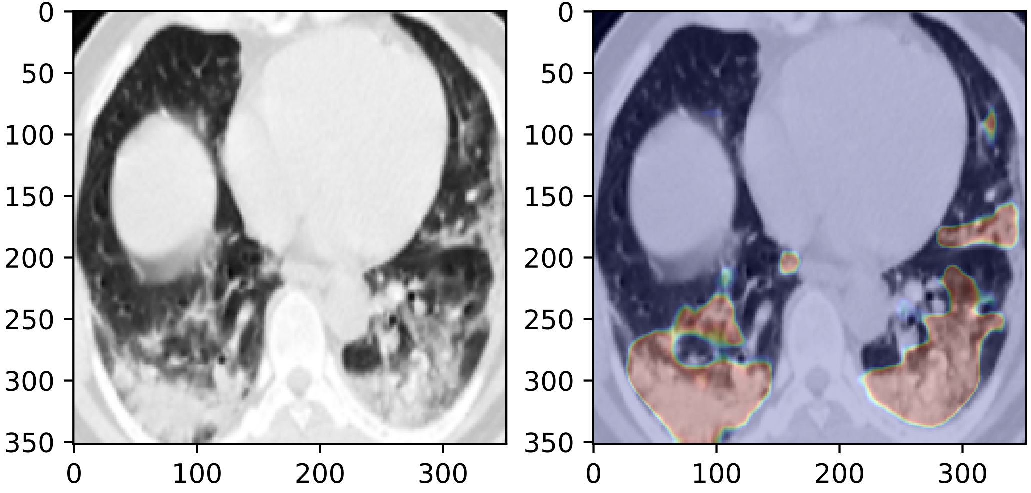

Lesion Proportion: 39.11%

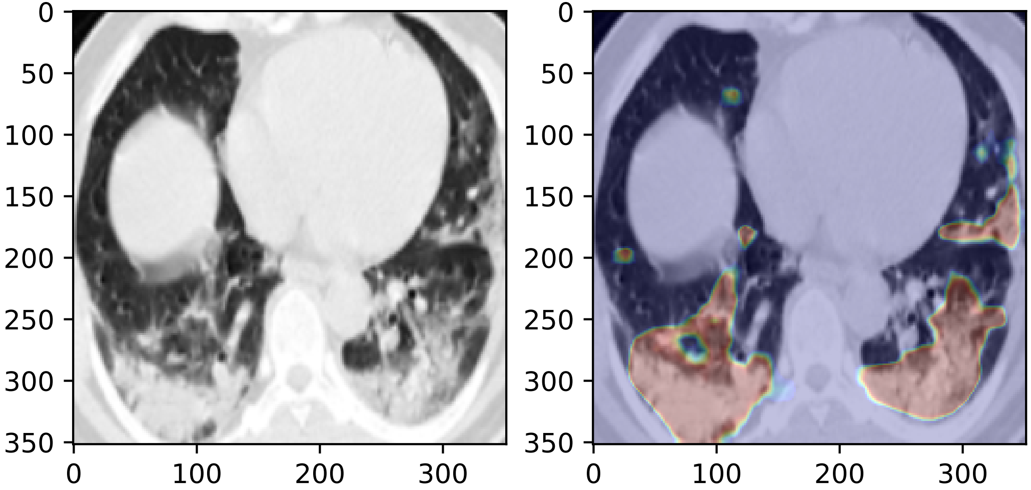

Lesion Proportion: 38.62%

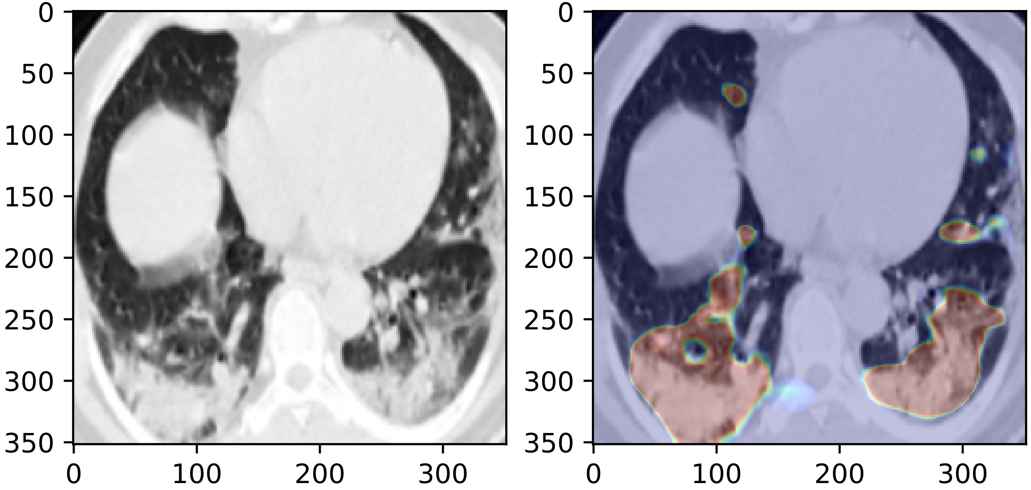

Lesion Proportion: 39.82%

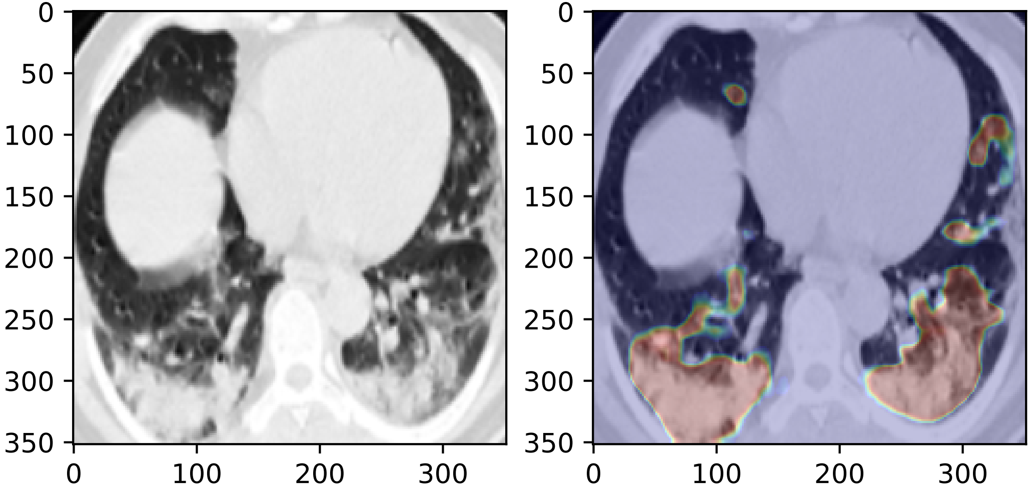

Lesion Proportion: 37.86%

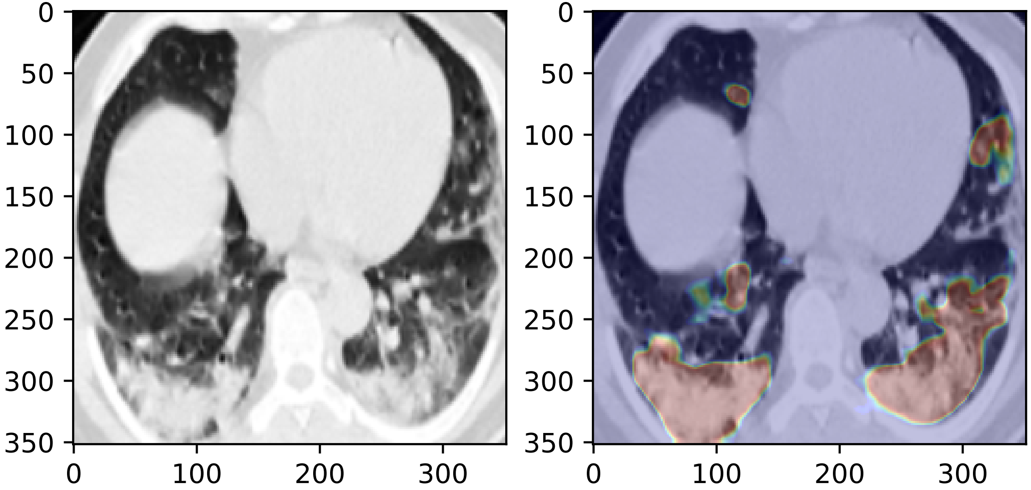

Lesion Proportion: 46.81%

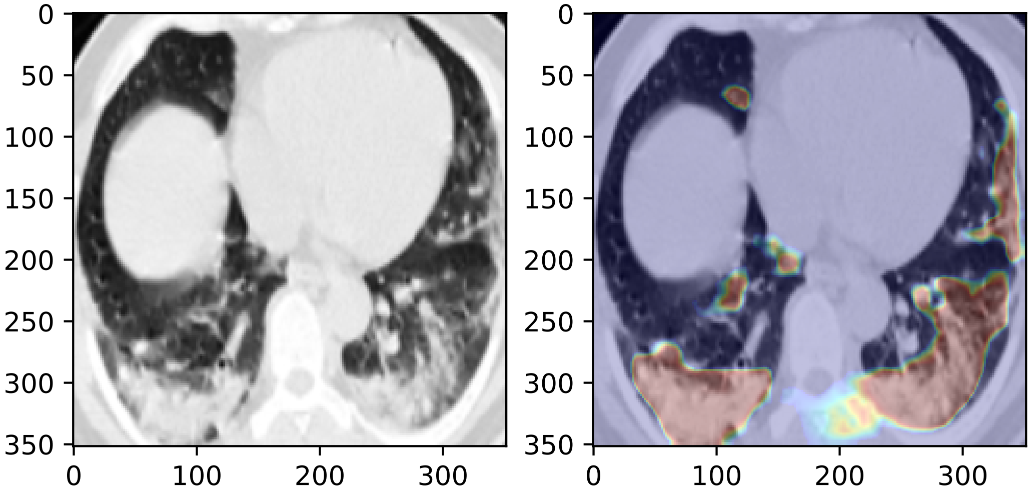

Lesion Proportion: 40.72%

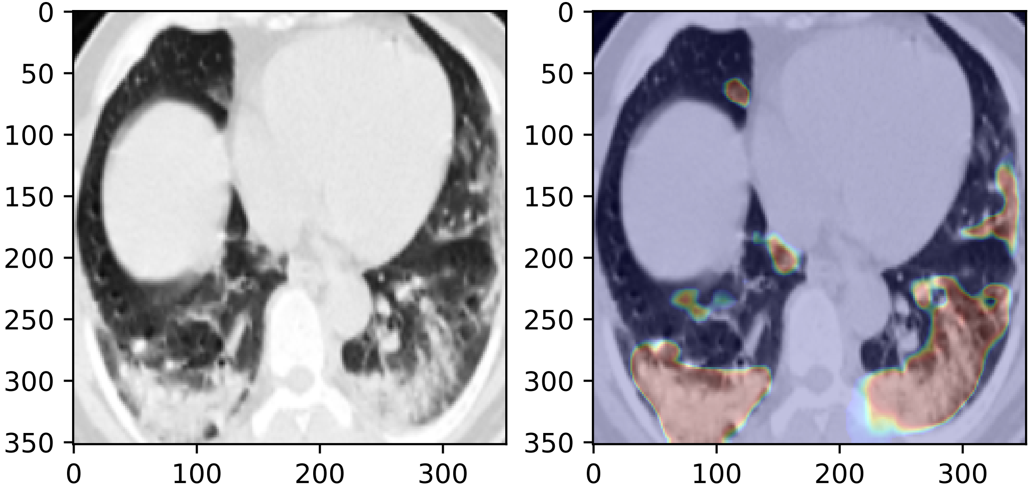

Lesion Proportion: 42.05%

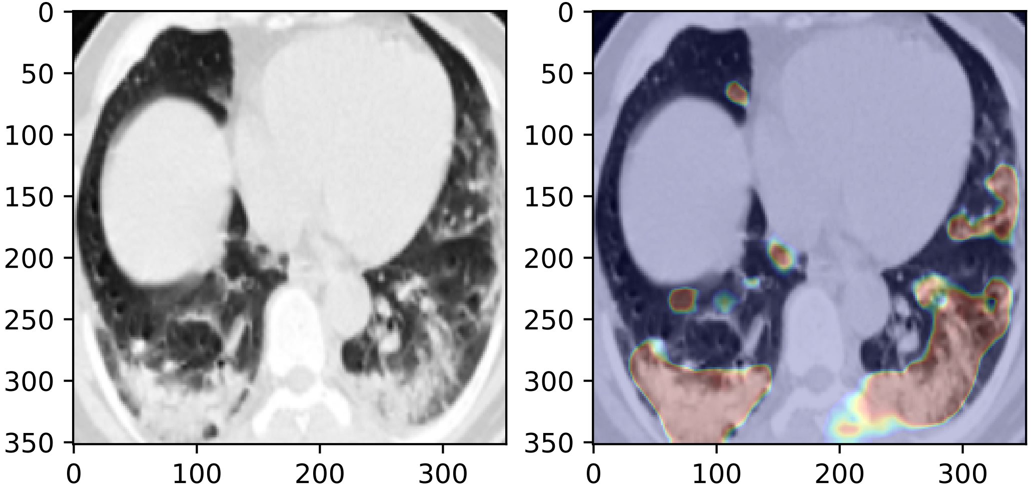

Lesion Proportion: 38.18%

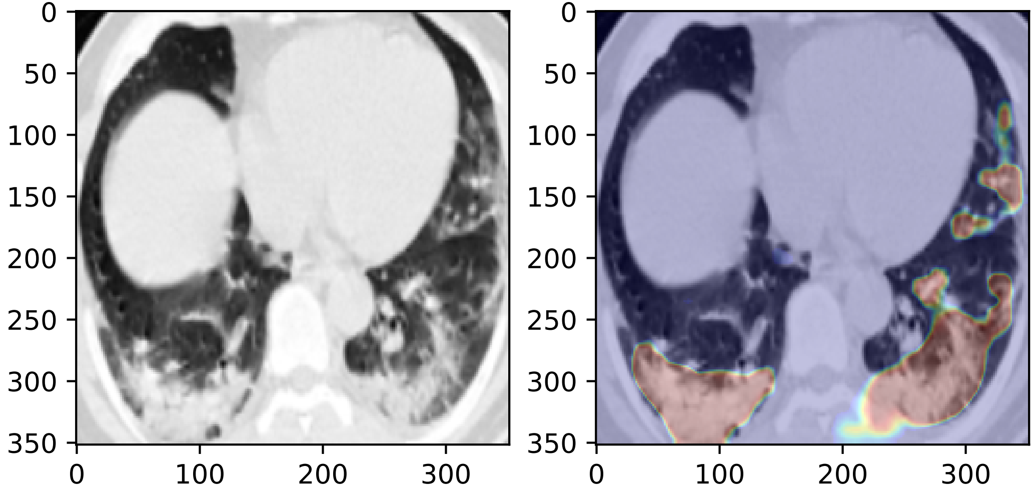

Lesion Proportion: 40.04%

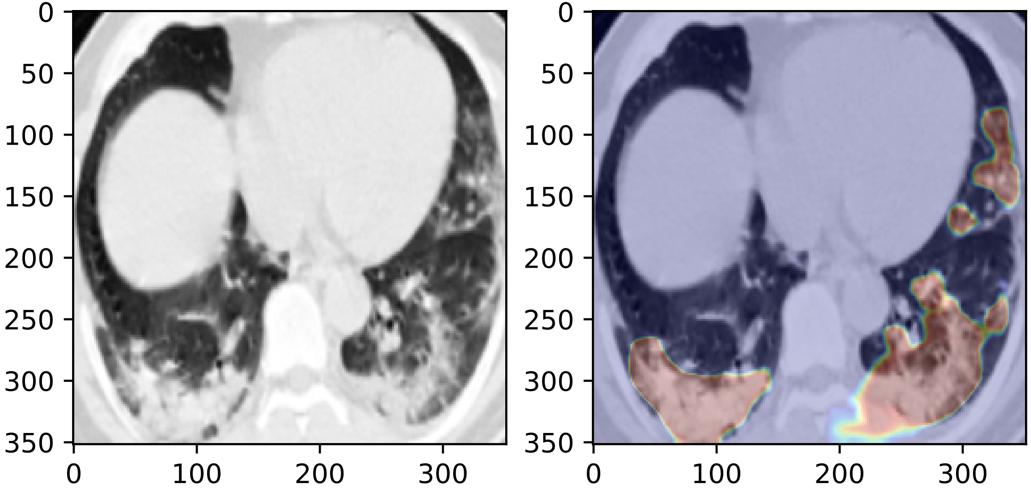

Lesion Proportion: 46.39%

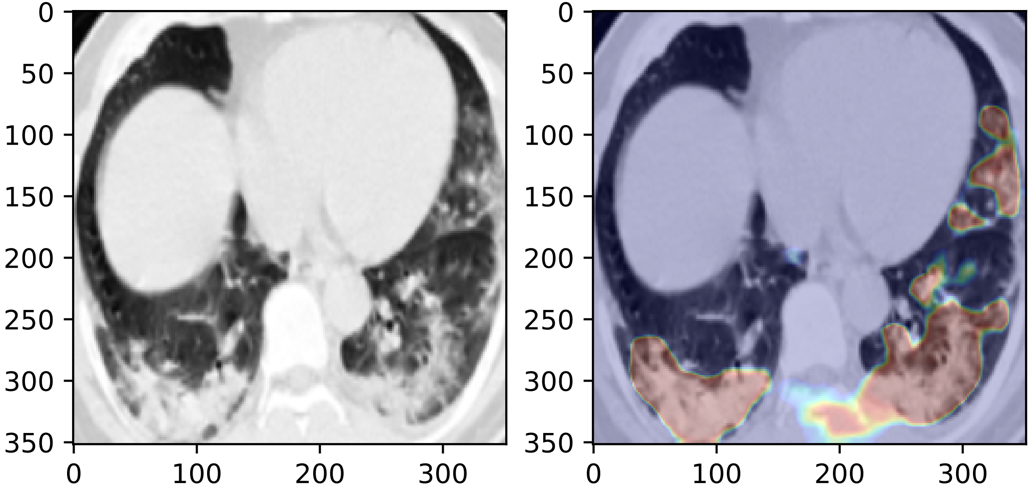

Lesion Proportion: 50.05%

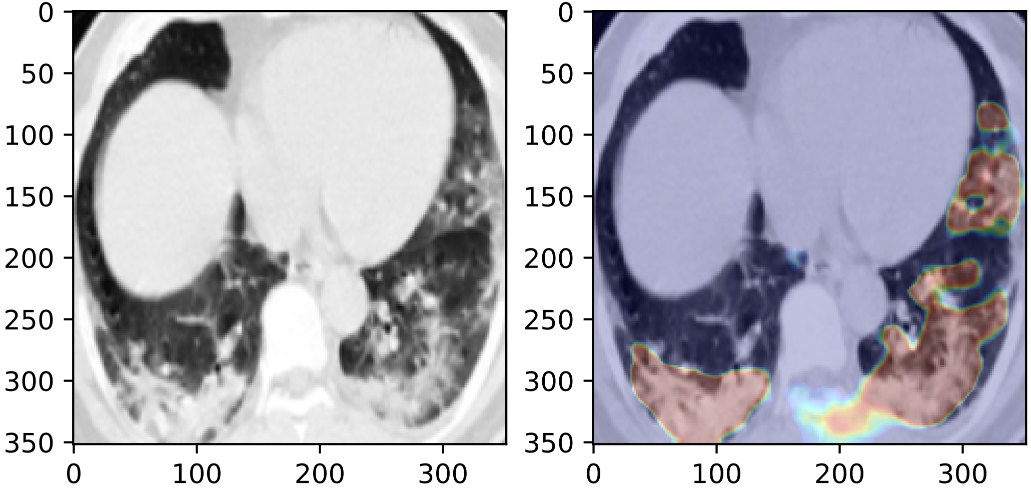

Lesion Proportion: 50.71%

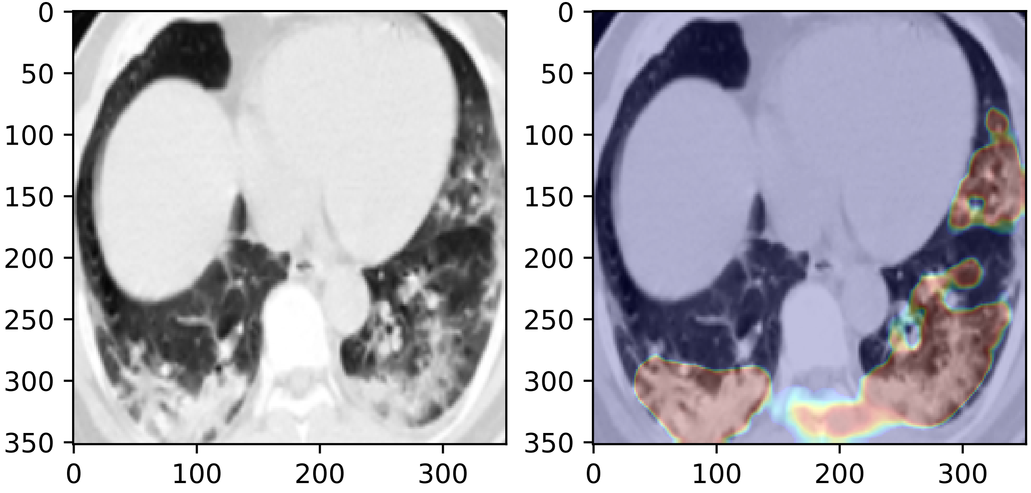

Lesion Proportion: 48.91%

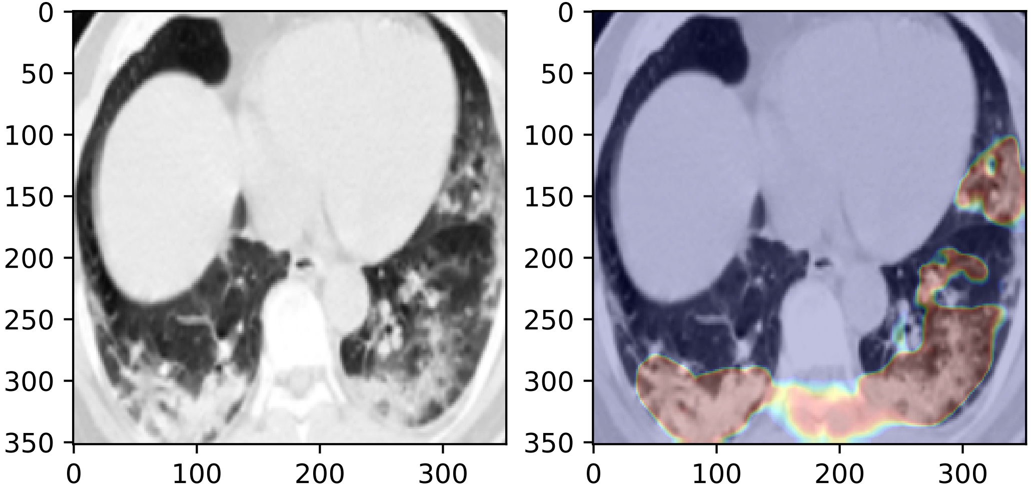

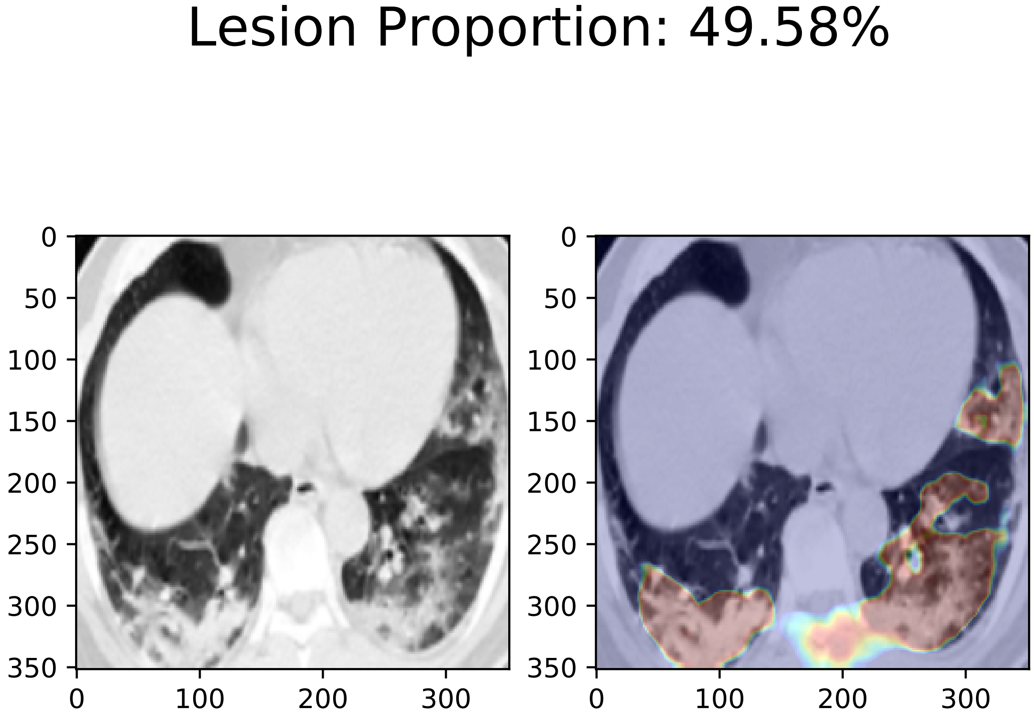
Lesion Proportion: 49.58%
0
50
100
150
200
250
300
350
0
100
200
300

Lesion Proportion: 42.22%

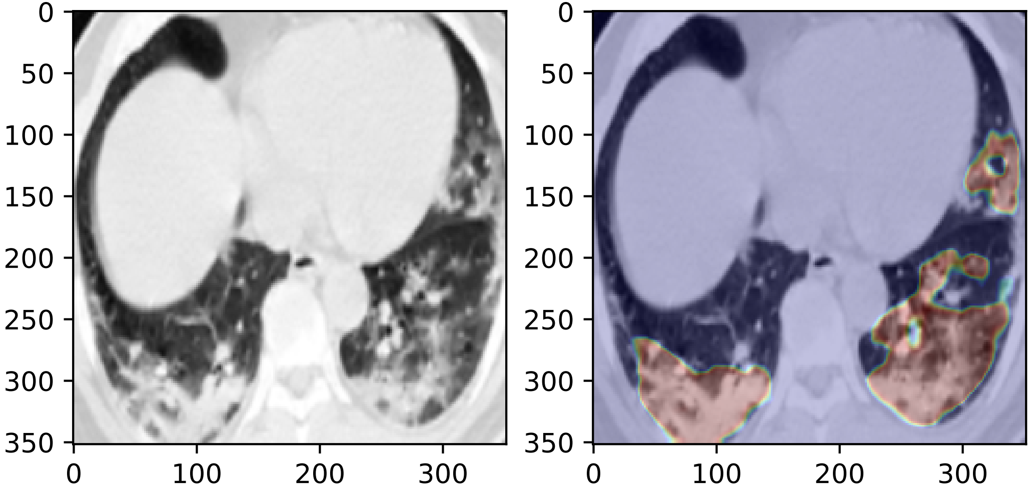

Lesion Proportion: 42.68%

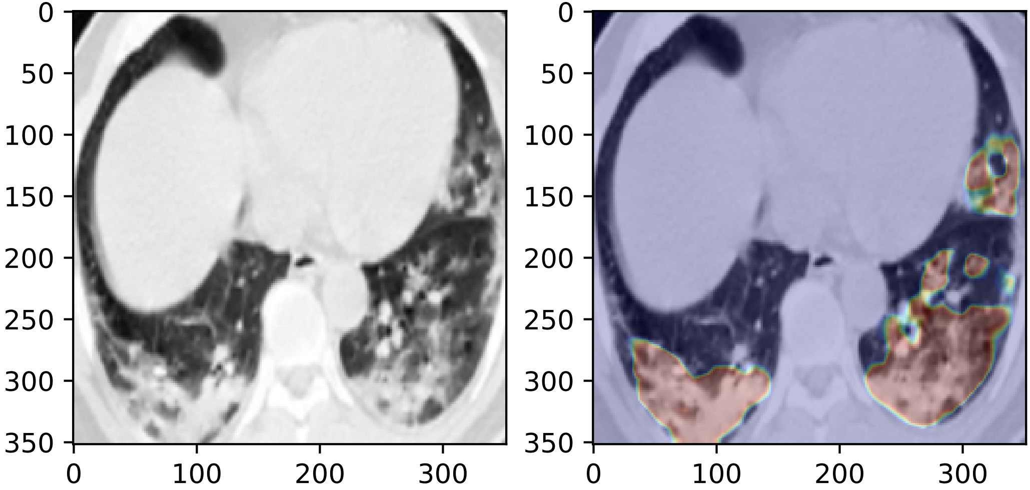

Lesion Proportion: 42.80%

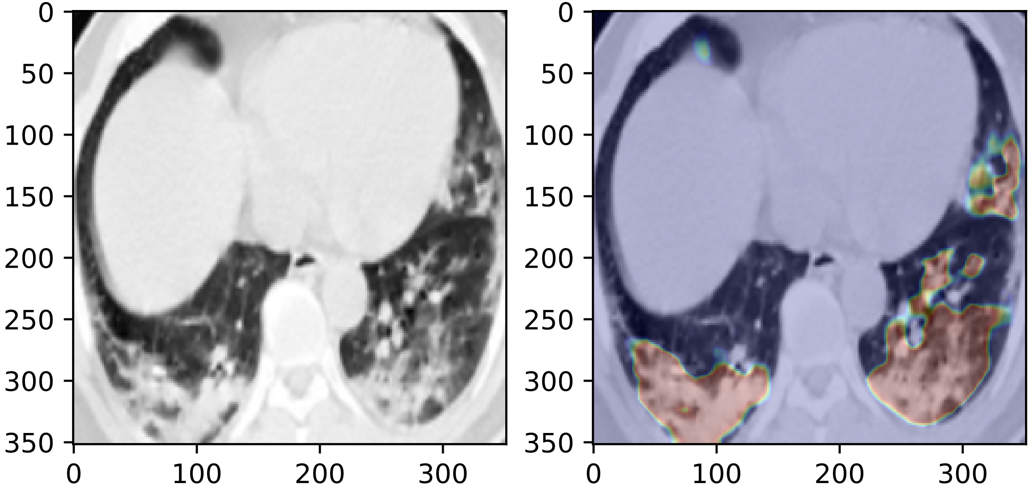

Lesion Proportion: 41.77%

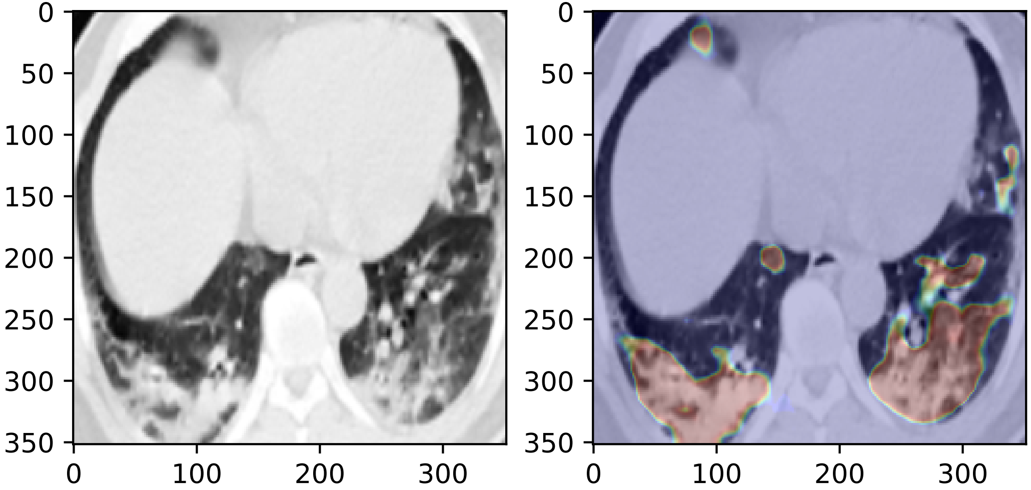

Lesion Proportion: 43.17%

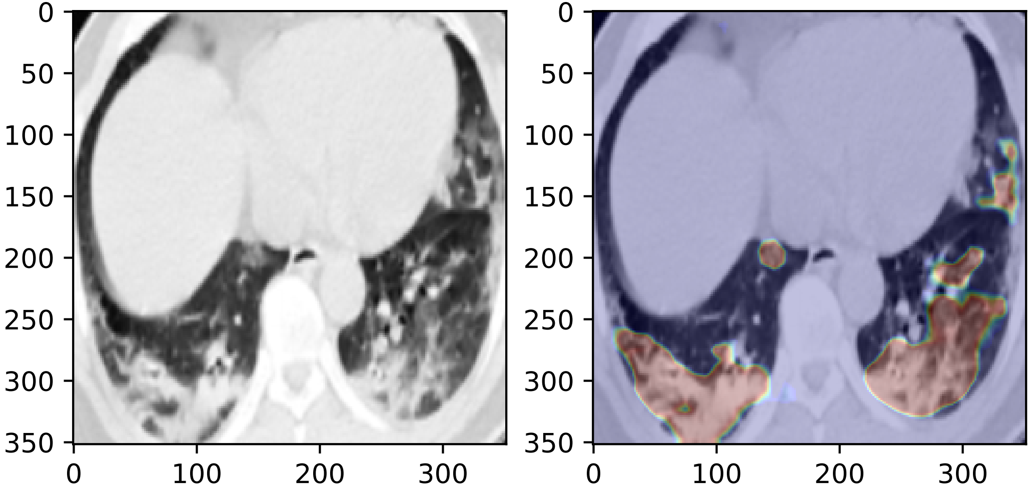

Lesion Proportion: 43.43%

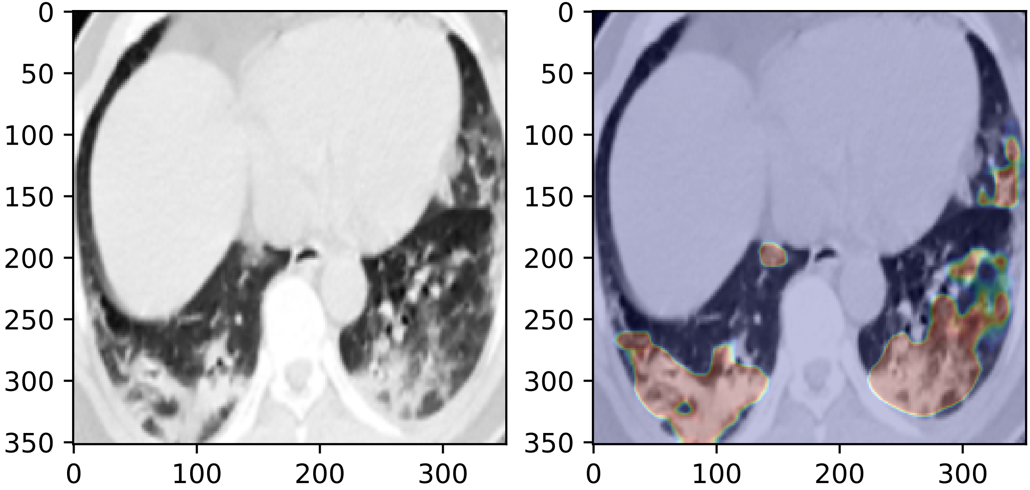

Lesion Proportion: 43.94%

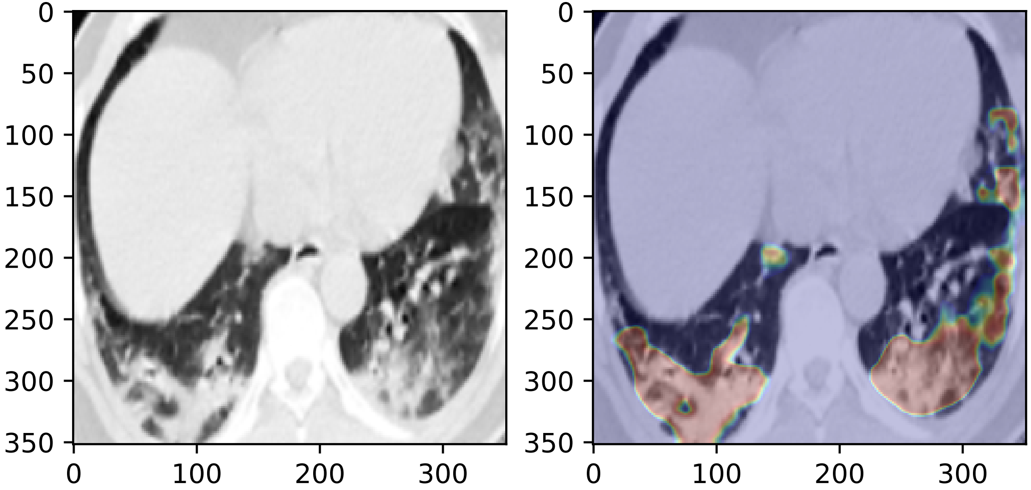

Lesion Proportion: 40.26%

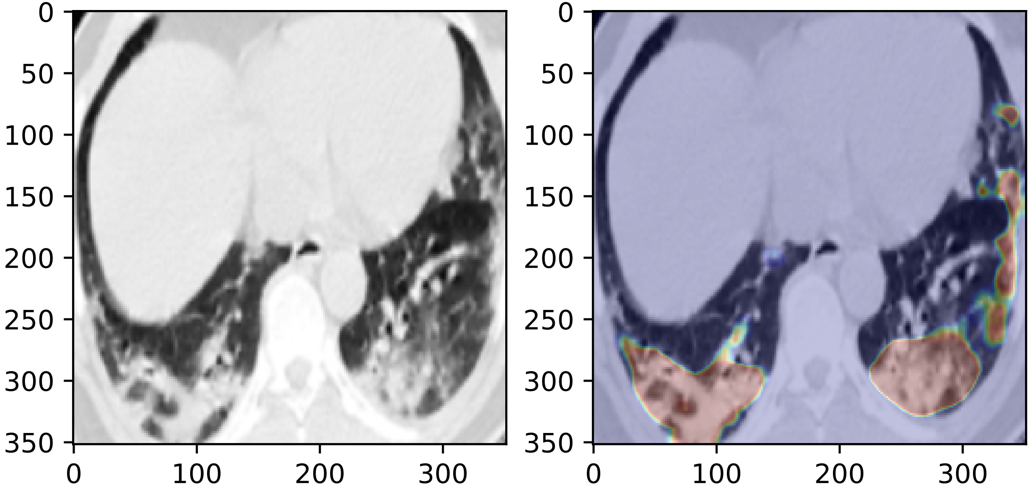

Lesion Proportion: 39.84%

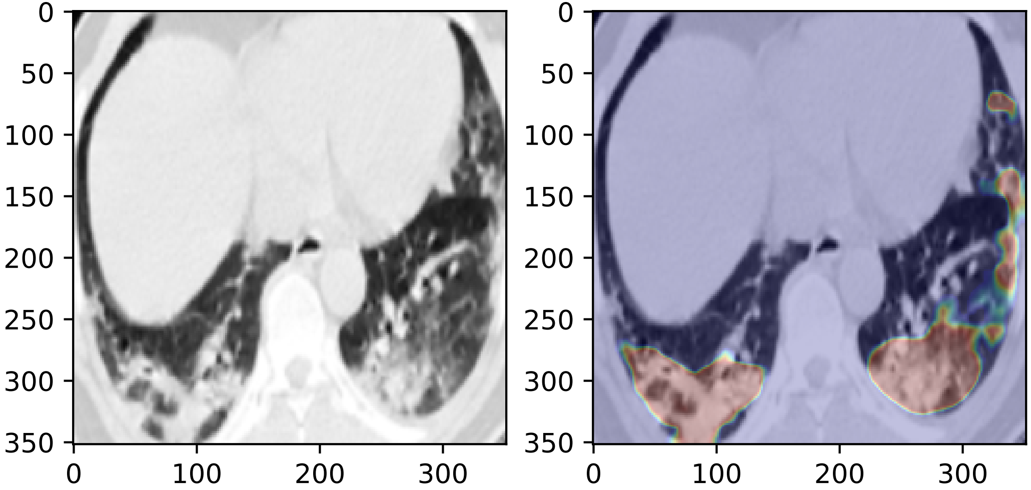

Lesion Proportion: 33.06%

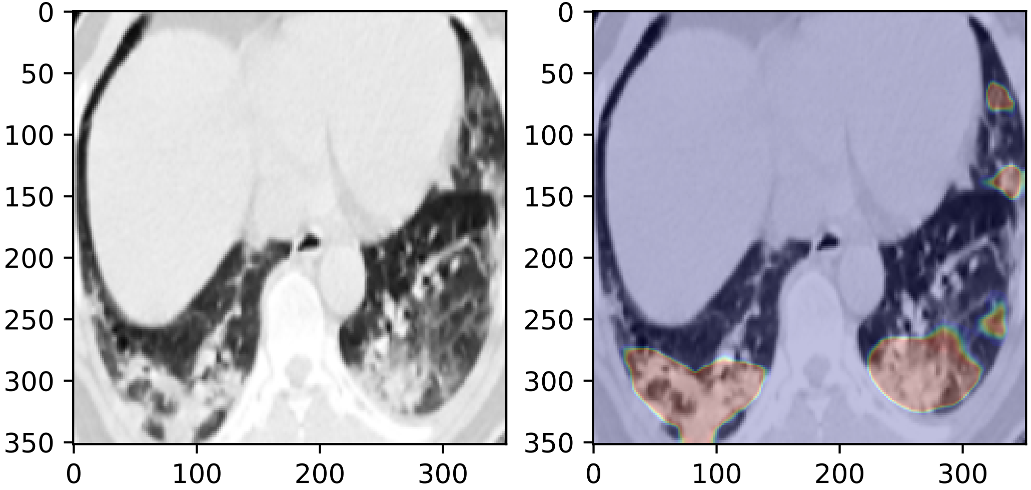

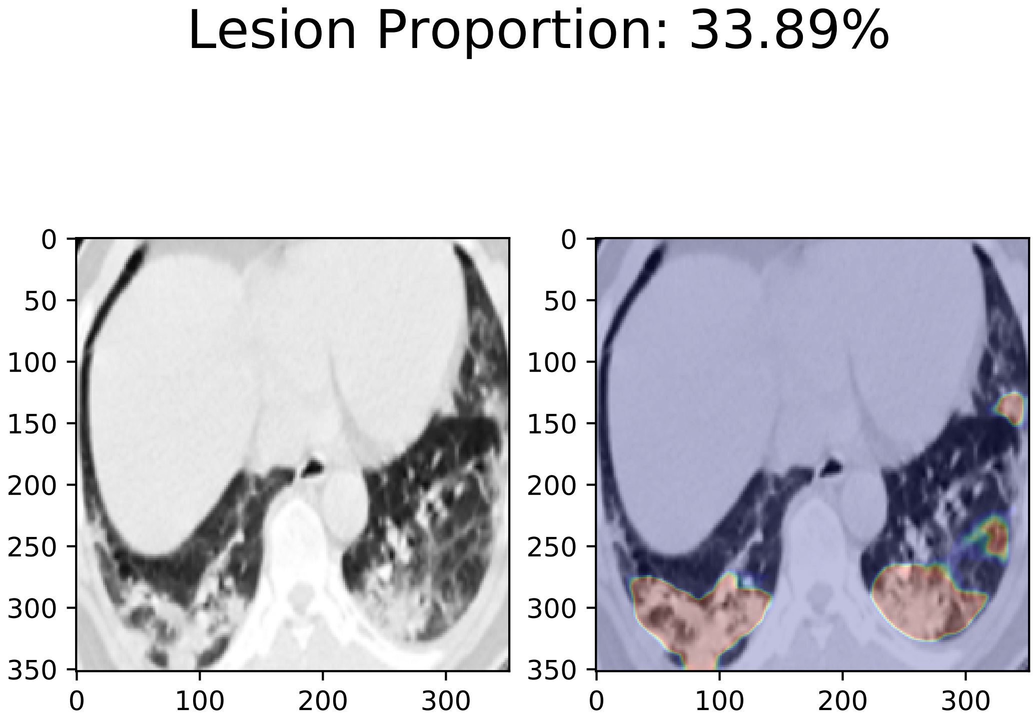

Lesion Proportion: 33.89%
0
50
100
150
200
250
300
350
0
100
200
300

Lesion Proportion: 35.81%

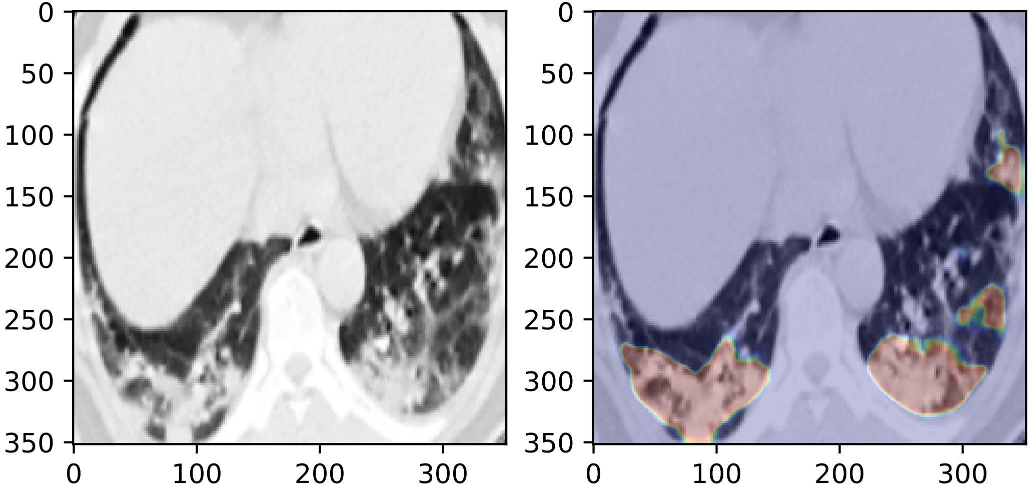

Lesion Proportion: 39.50%

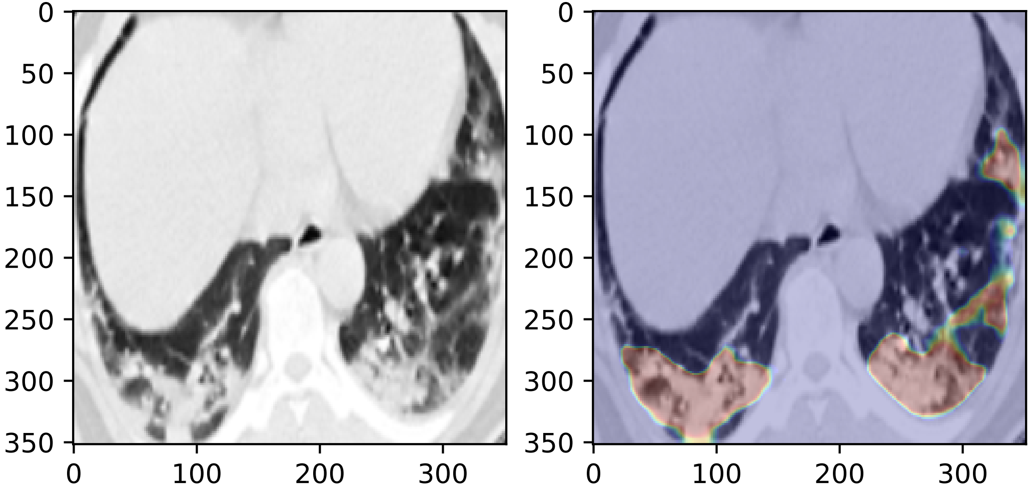

Lesion Proportion: 39.12%

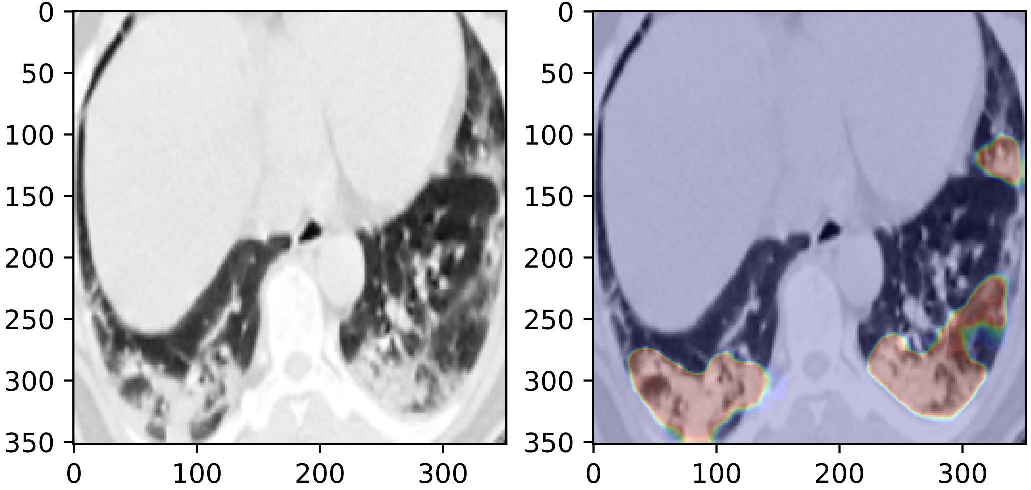

Lesion Proportion: 41.39%

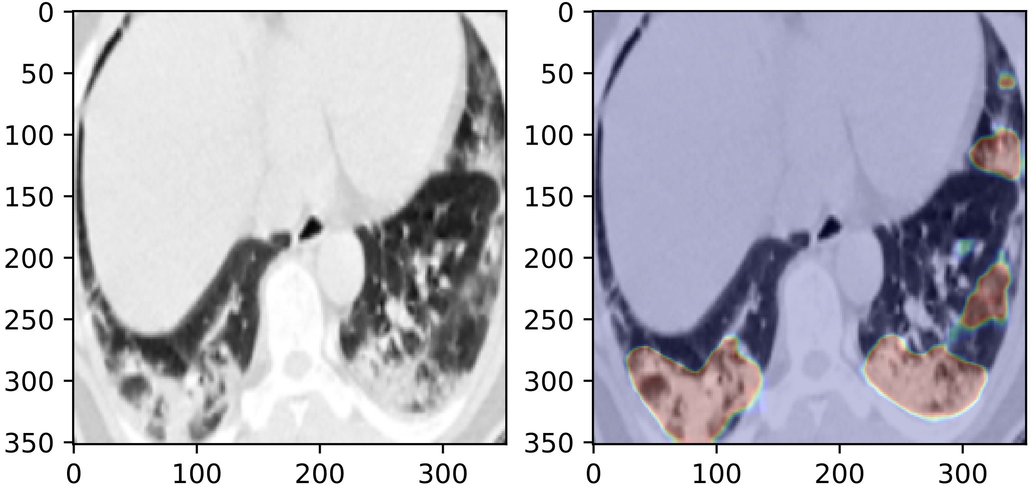

Lesion Proportion: 47.83%

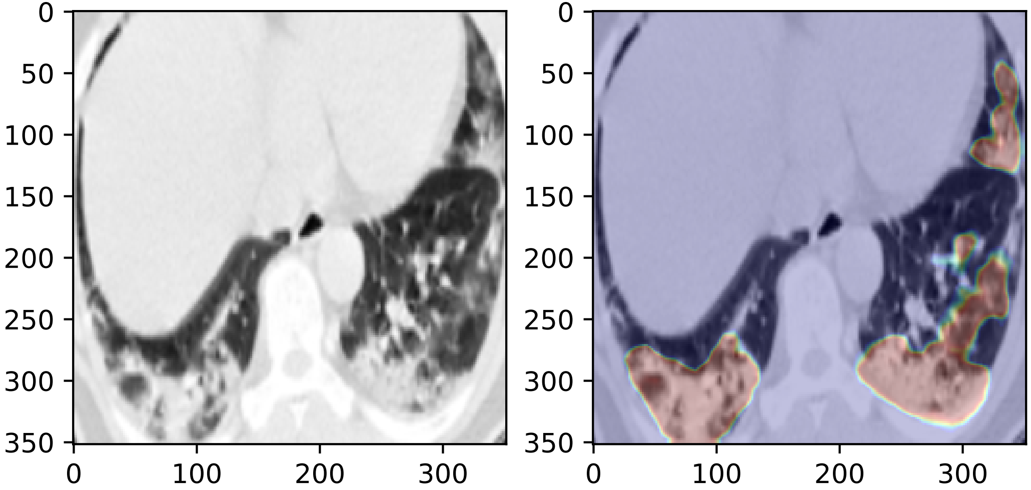

Lesion Proportion: 48.53%

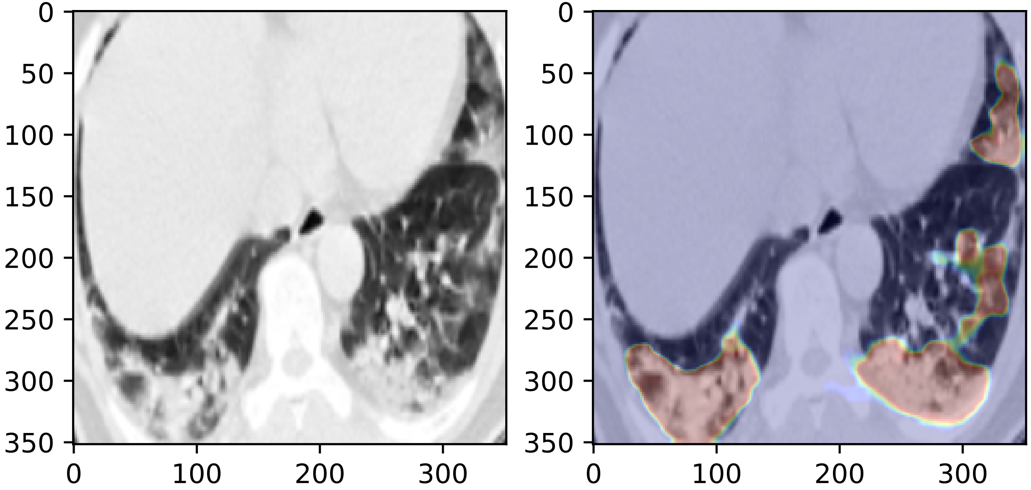

Lesion Proportion: 53.55%

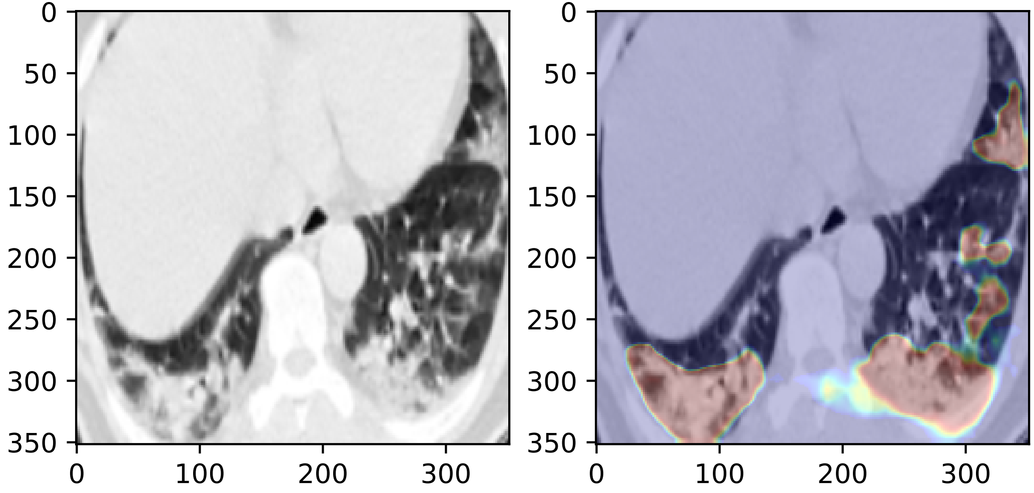

Lesion Proportion: 49.77%

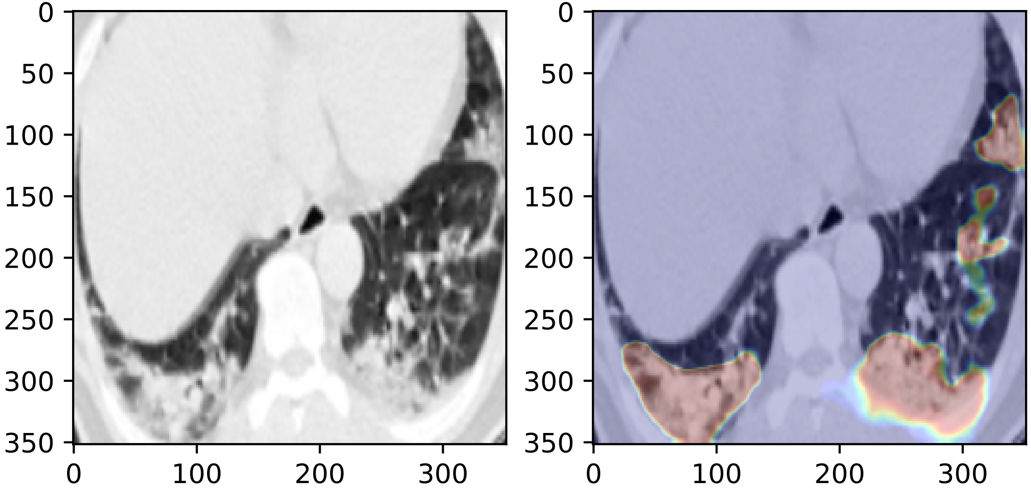

Lesion Proportion: 56.19%

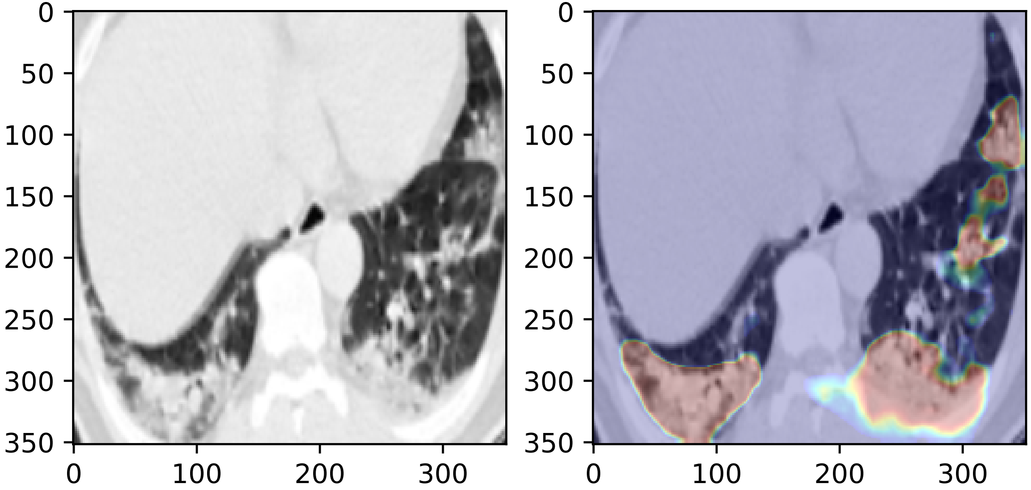

Lesion Proportion: 53.68%

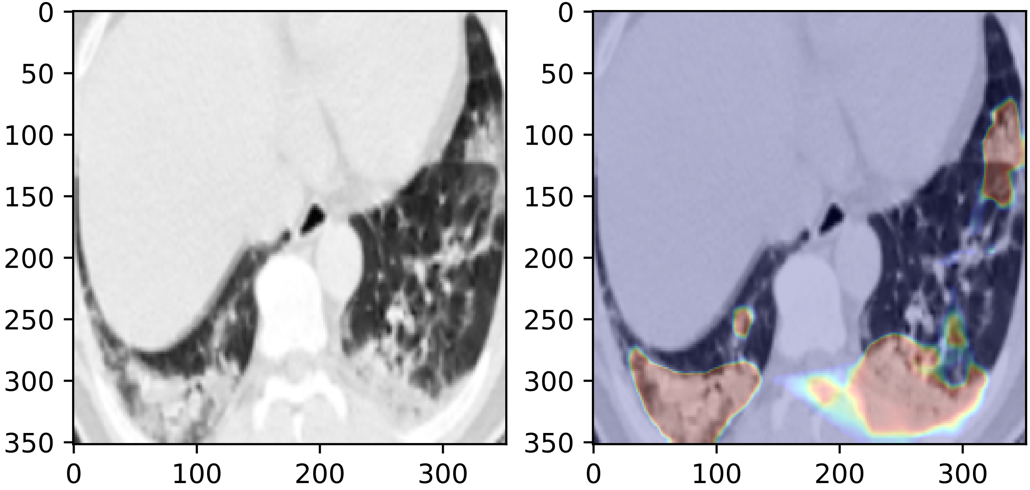

Lesion Proportion: 59.35%

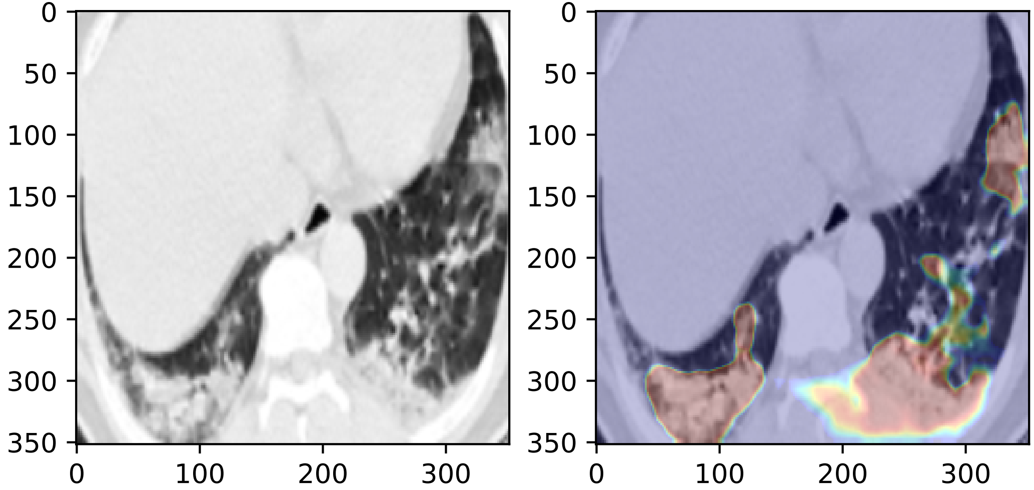

Lesion Proportion: 61.43%

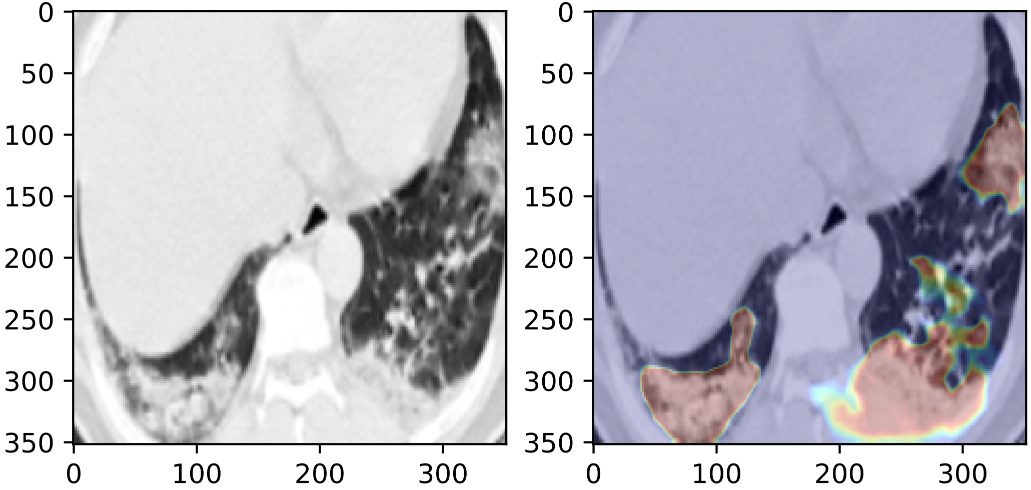

Lesion Proportion: 63.50%

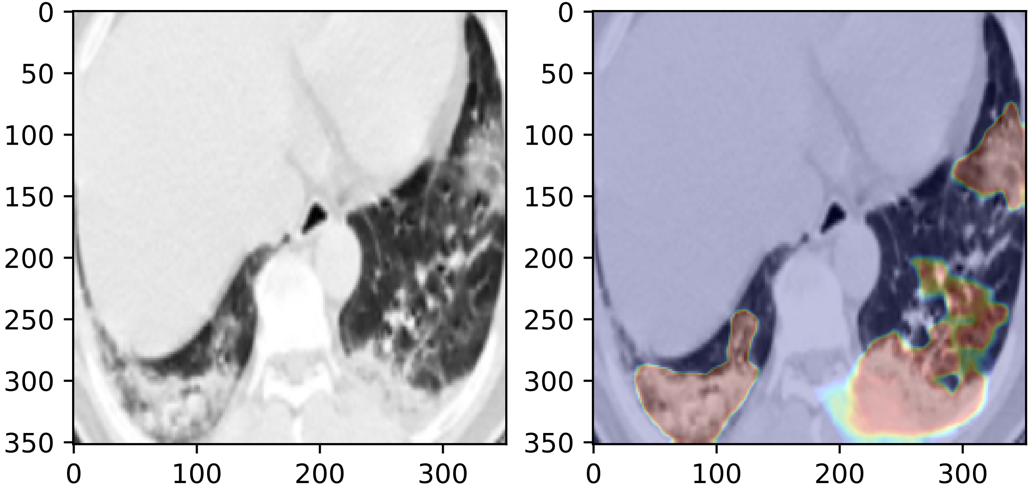

Lesion Proportion: 69.71%

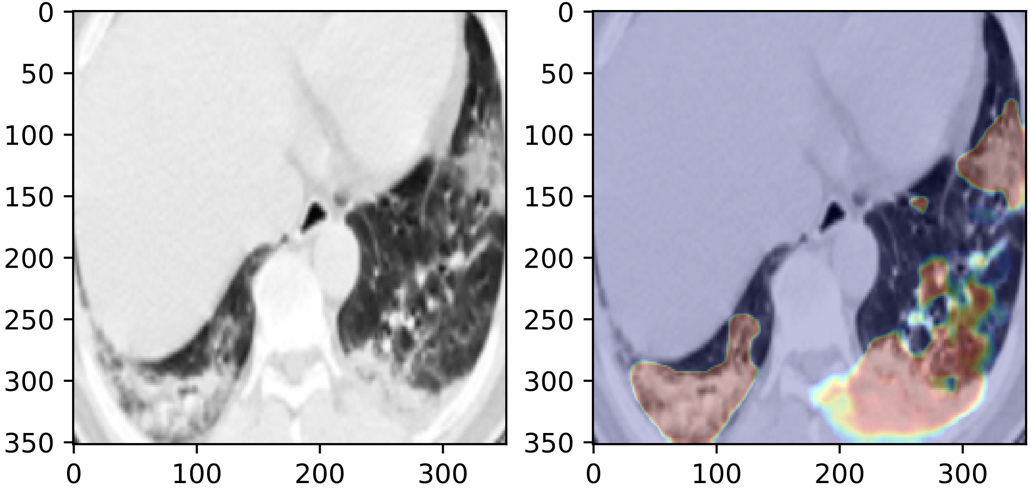

Lesion Proportion: 63.79%

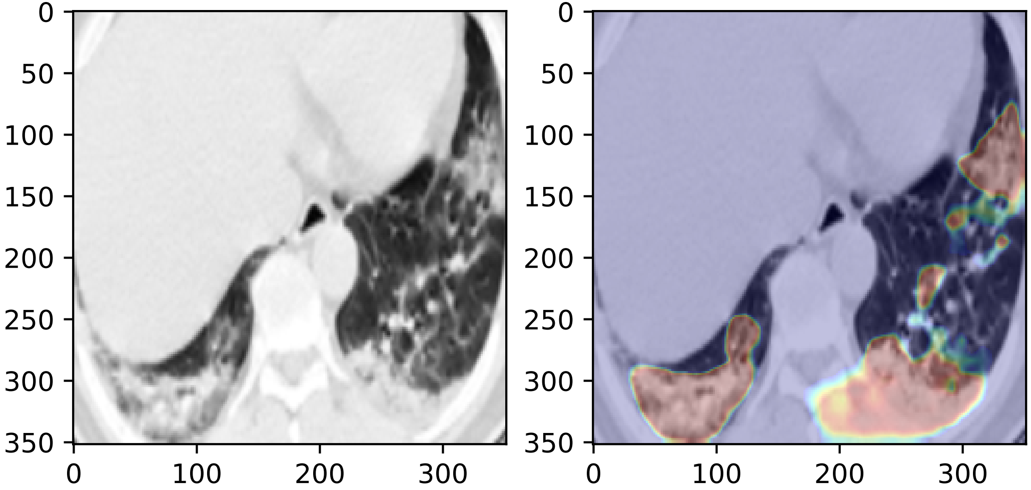

Lesion Proportion: 55.78%

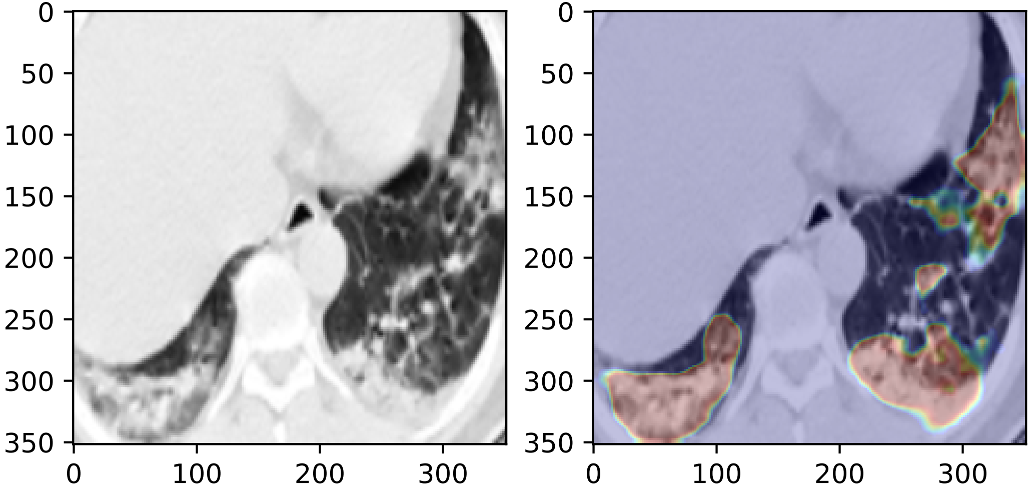

Lesion Proportion: 51.81%

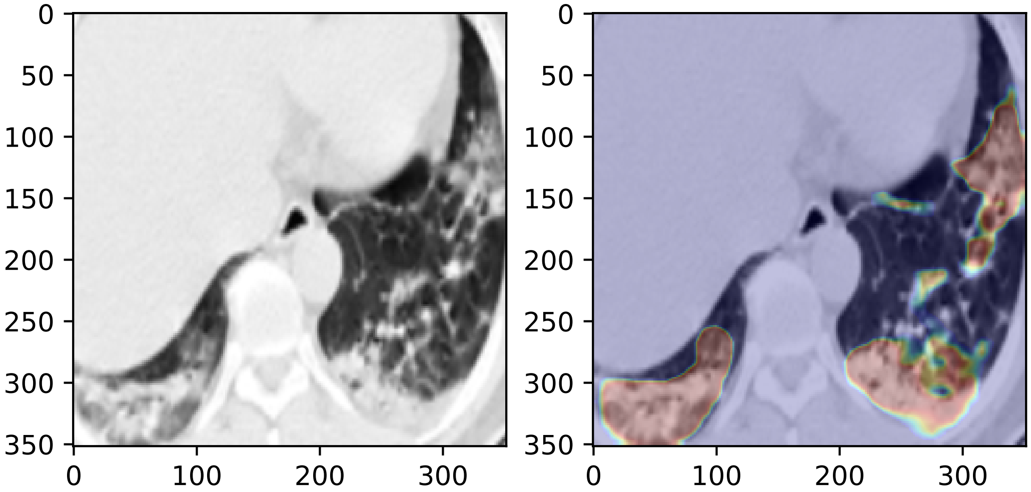

Lesion Proportion: 53.15%

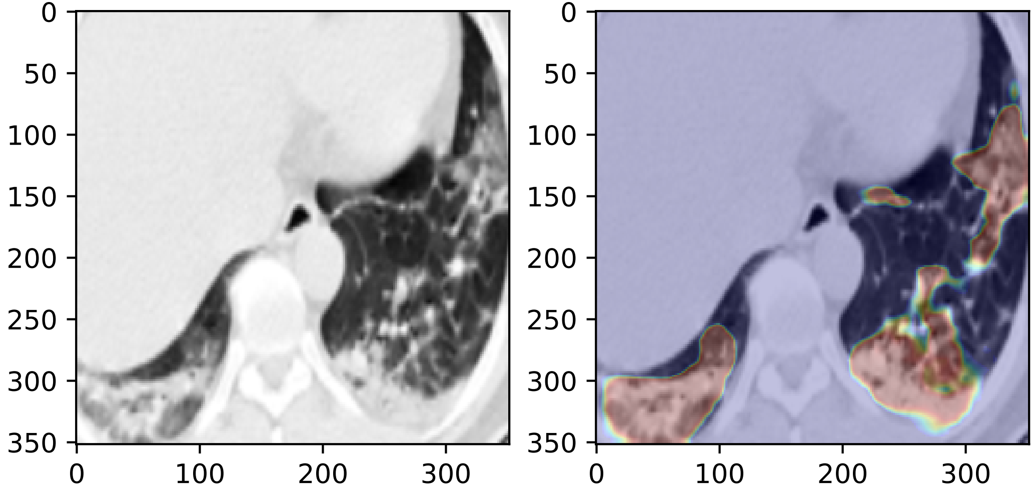

Lesion Proportion: 51.13%

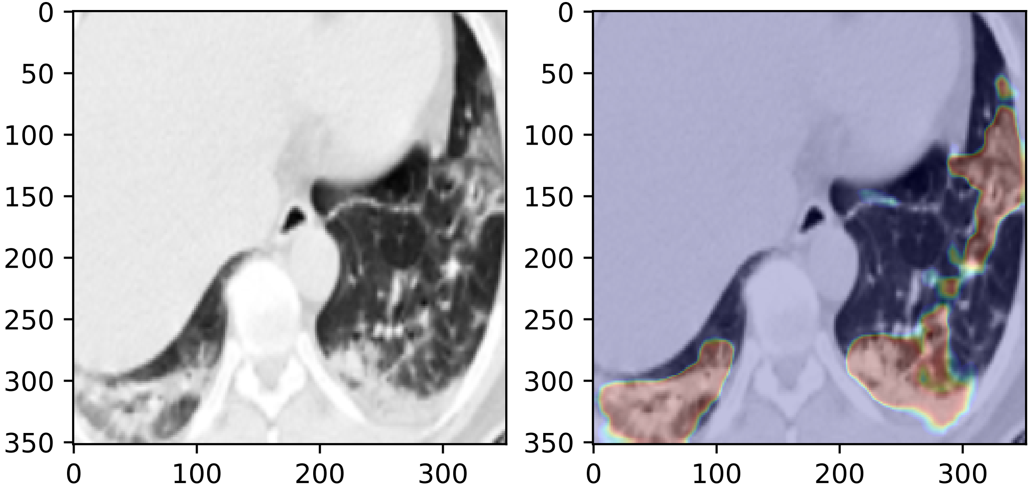

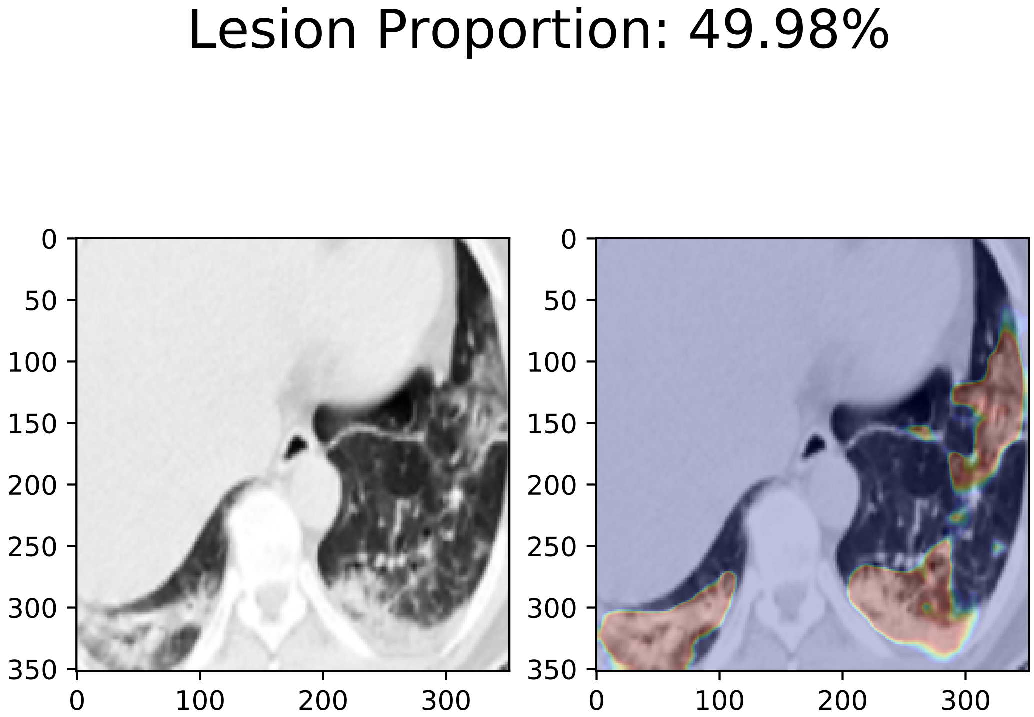
Lesion Proportion: 49.98%
0
50
100
150
200
250
300
350
0
100
200
300

Lesion Proportion: 48.92%

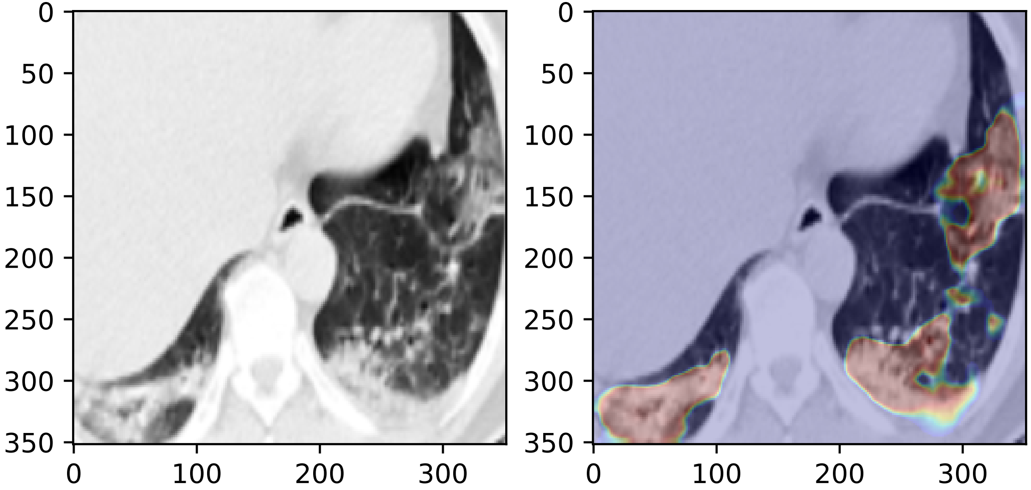

Lesion Proportion: 45.88%

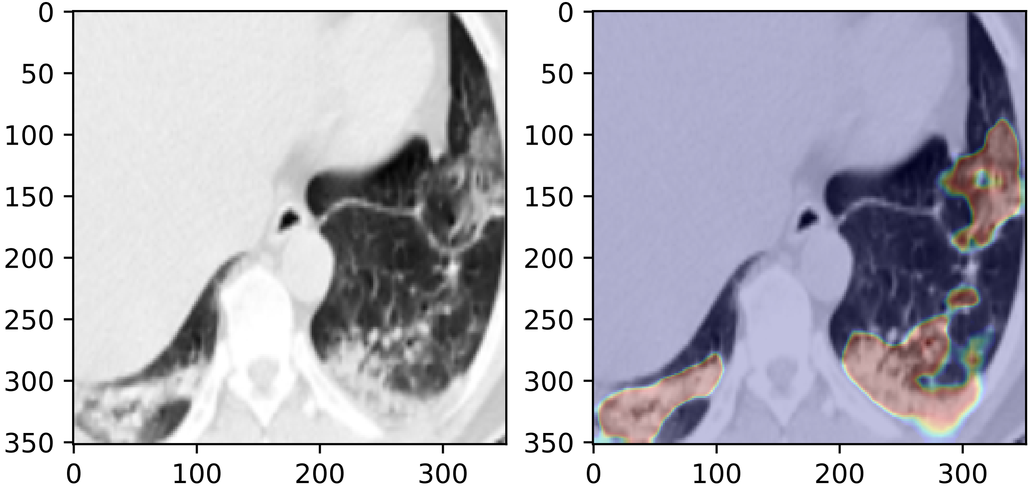

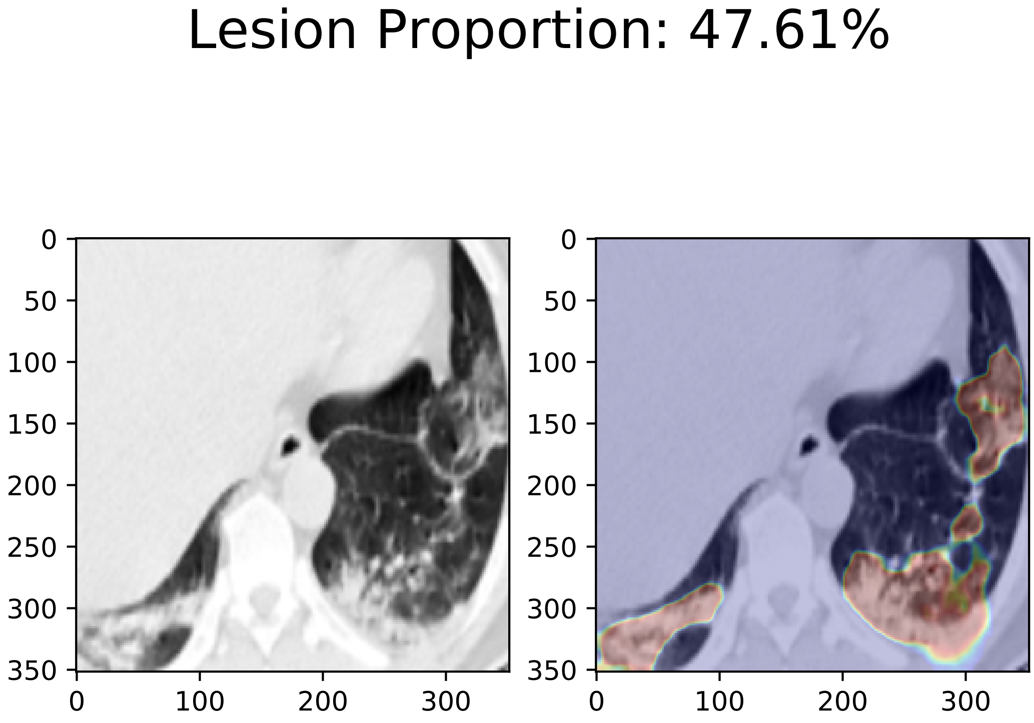
Lesion Proportion: 47.61%
0
50
100
150
200
250
300
350
0
100
200
300
0
50
100
150
200
250
300
350
0
100
200
300

Lesion Proportion: 44.91%

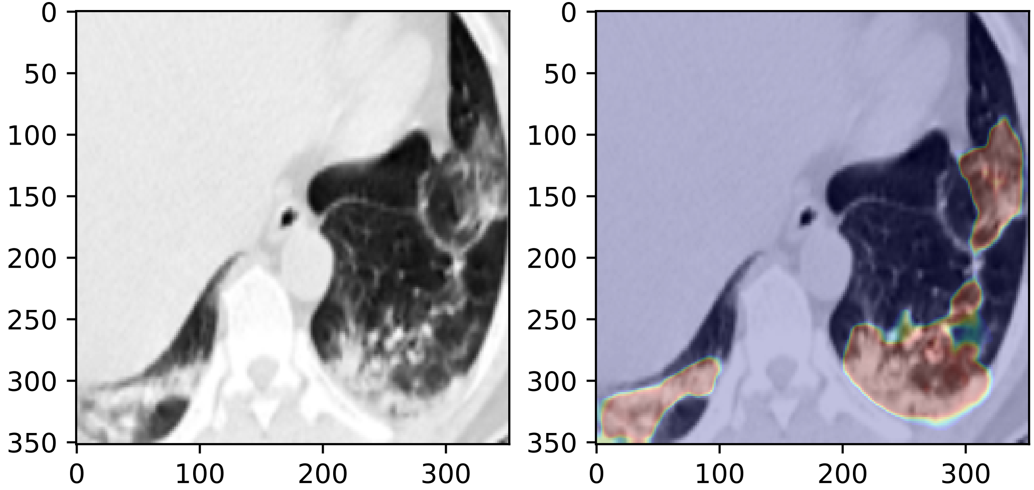

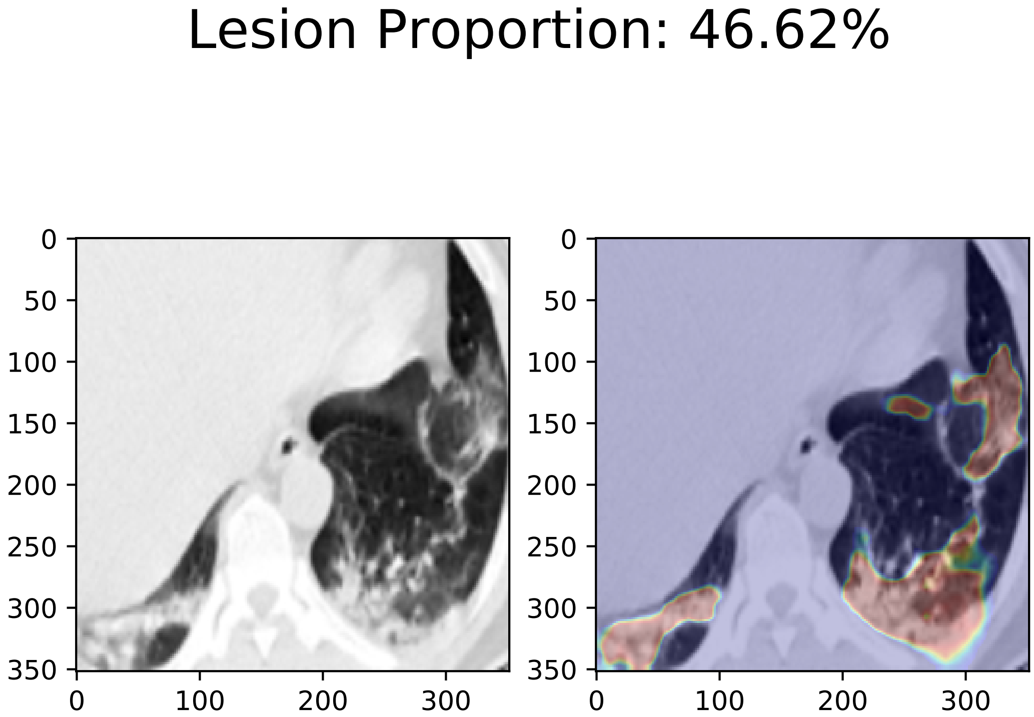
Lesion Proportion: 46.62%
0
50
100
150
200
250
300
350
0
100
200
300

Lesion Proportion: 43.66%

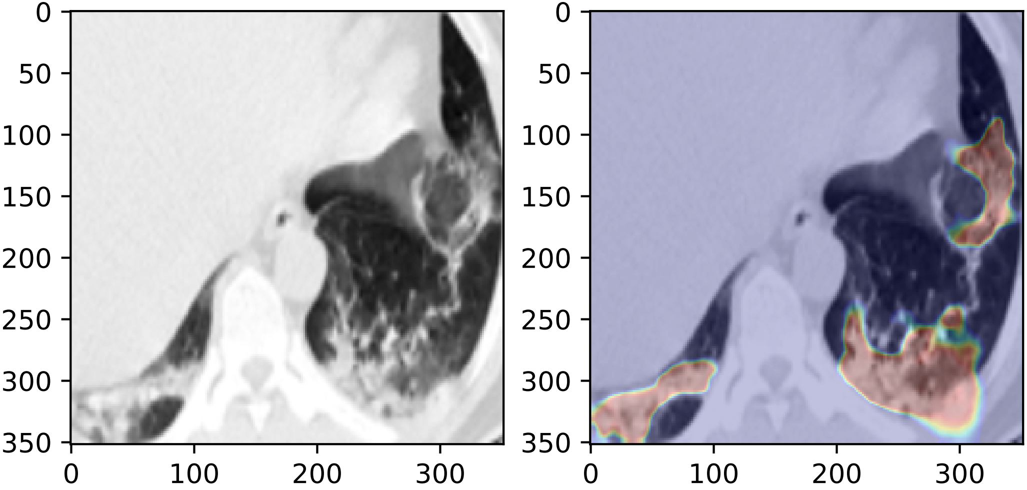

Lesion Proportion: 48.19%

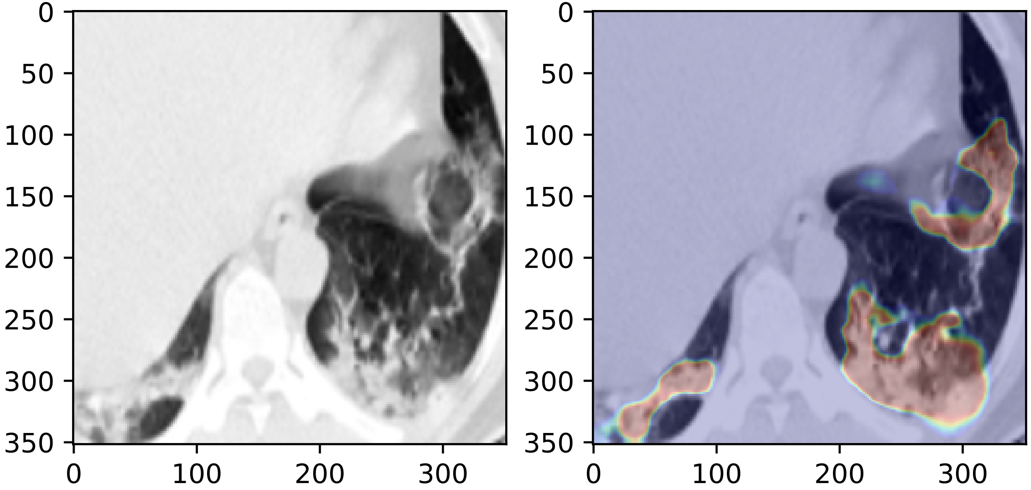

Lesion Proportion: 47.59%

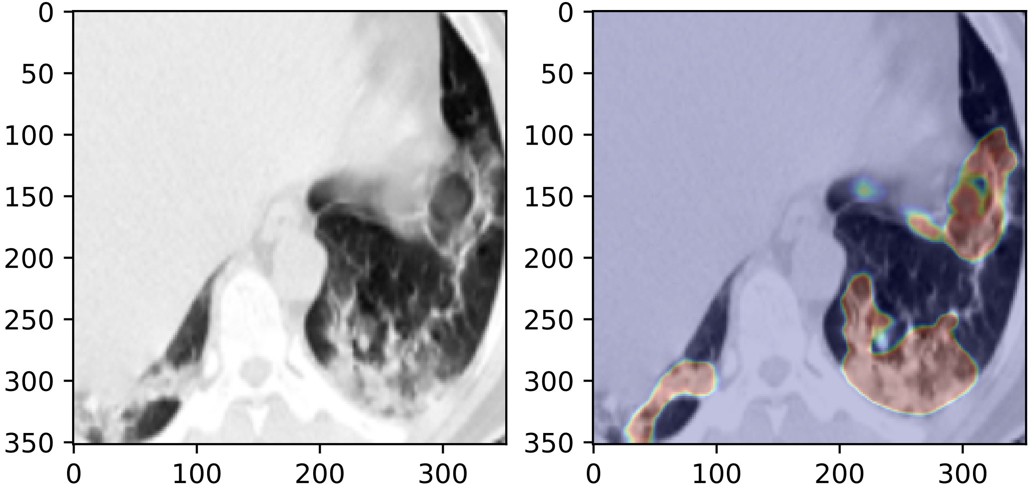

Lesion Proportion: 46.58%

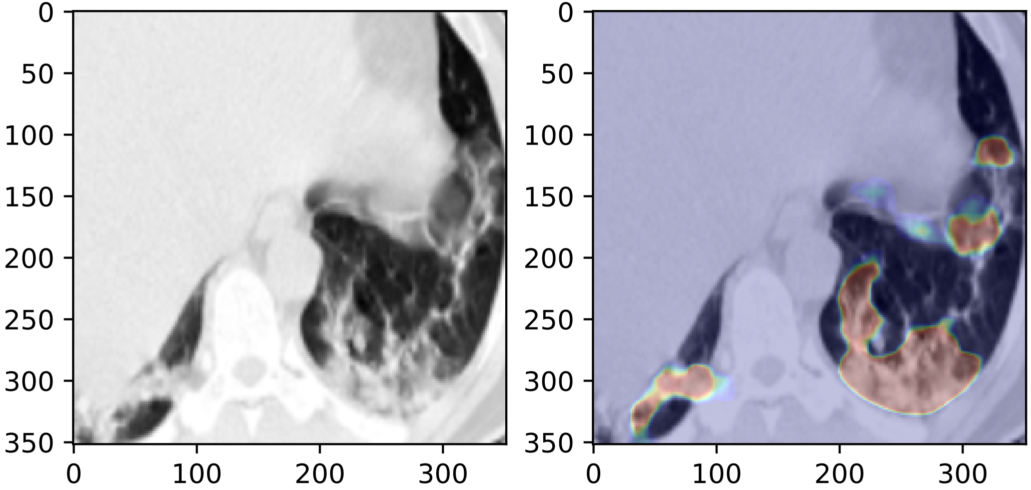

Lesion Proportion: 42.54%

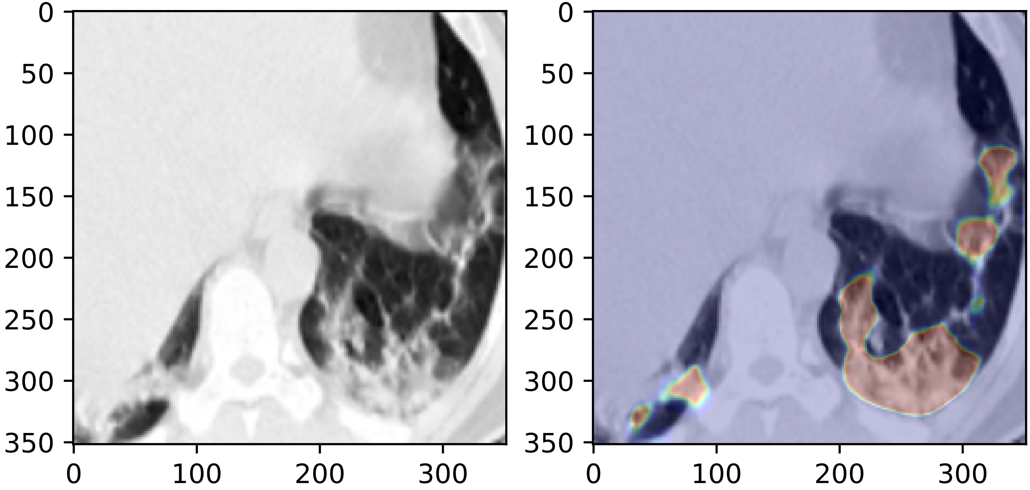

Lesion Proportion: 40.91%

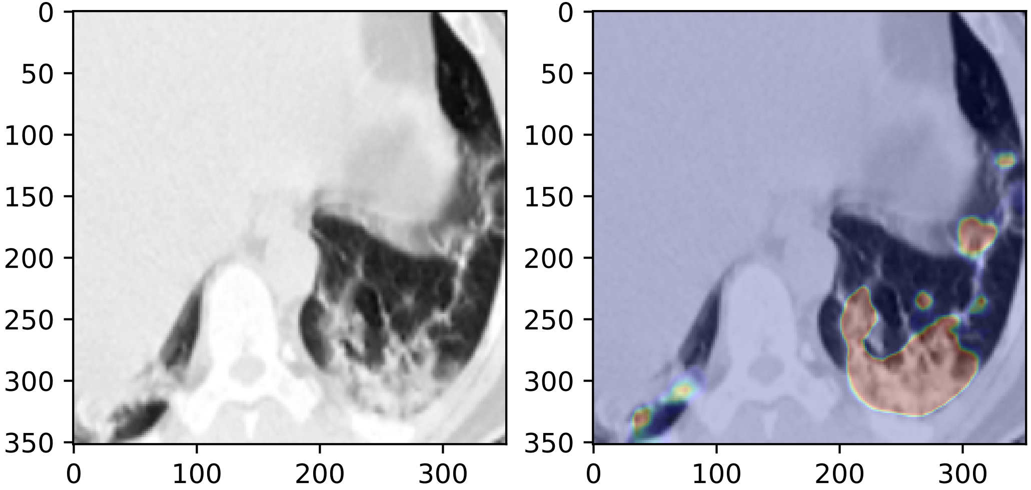

Lesion Proportion: 36.27%

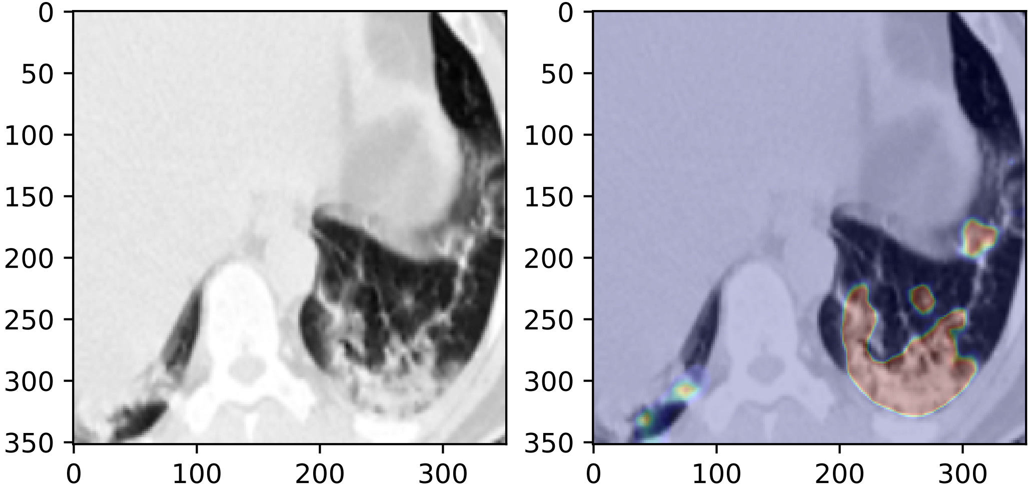

Lesion Proportion: 39.42%

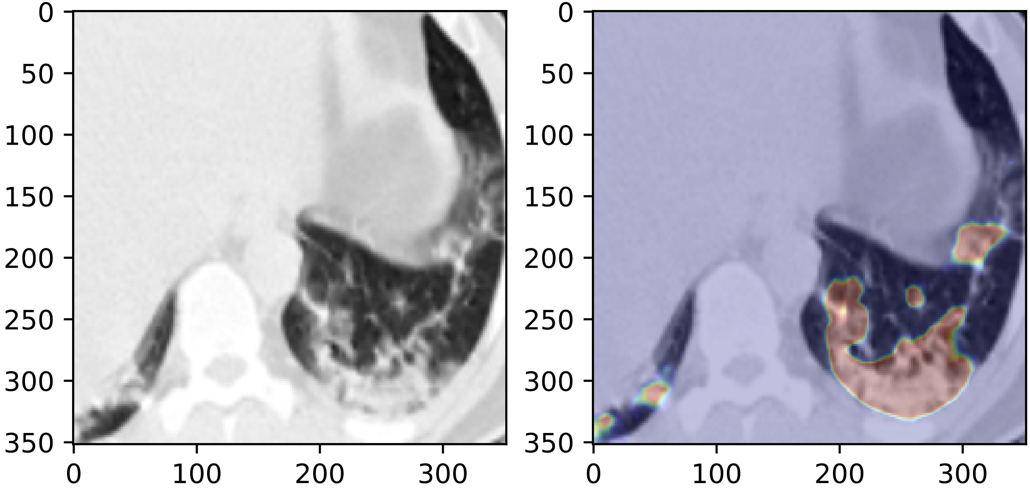

Lesion Proportion: 40.47%

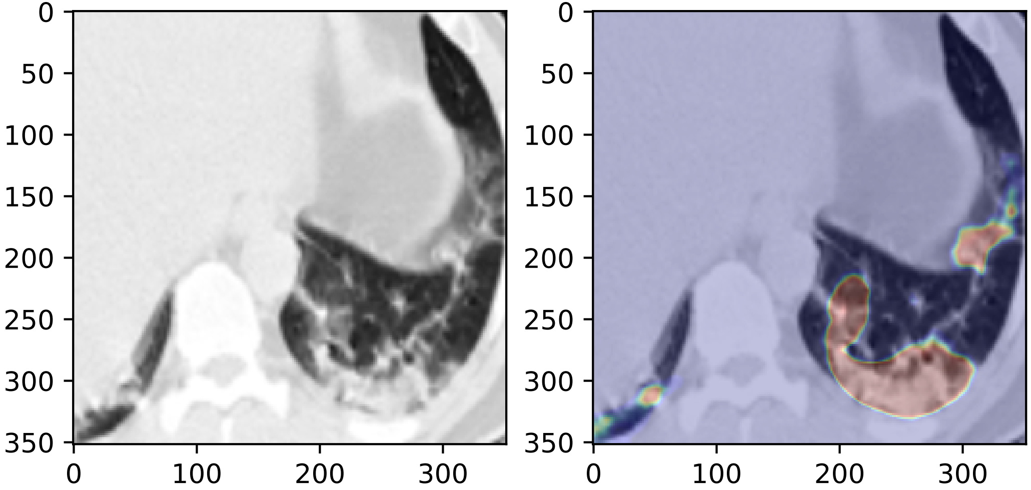

Lesion Proportion: 34.47%

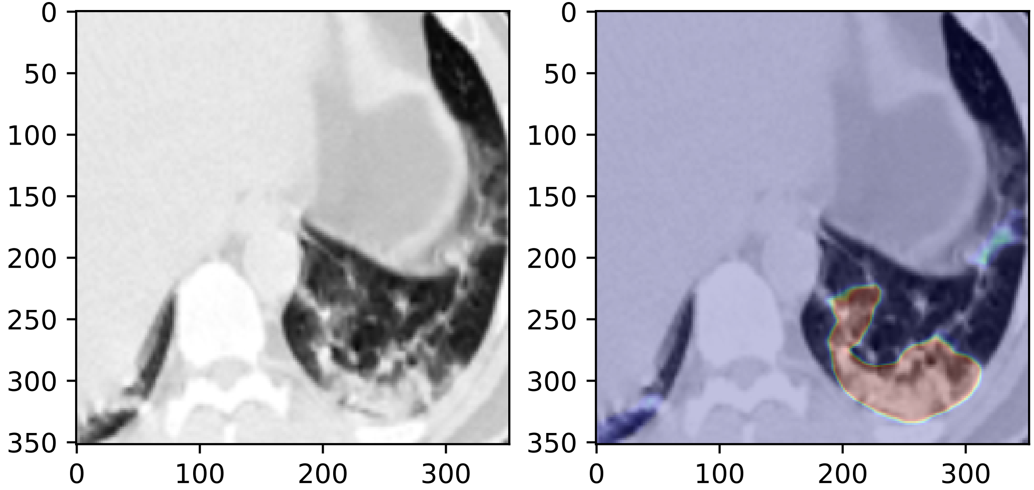

Lesion Proportion: 31.97%

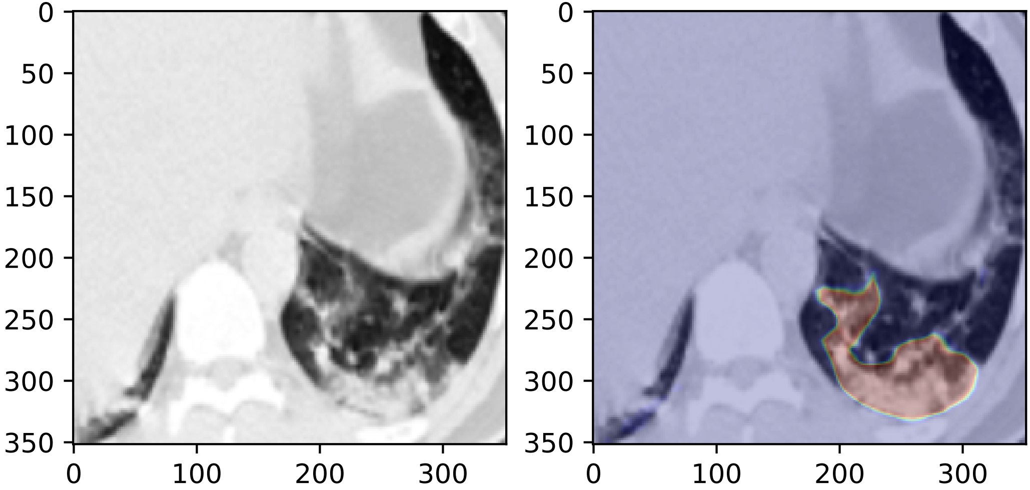

Lesion Proportion: 33.01%

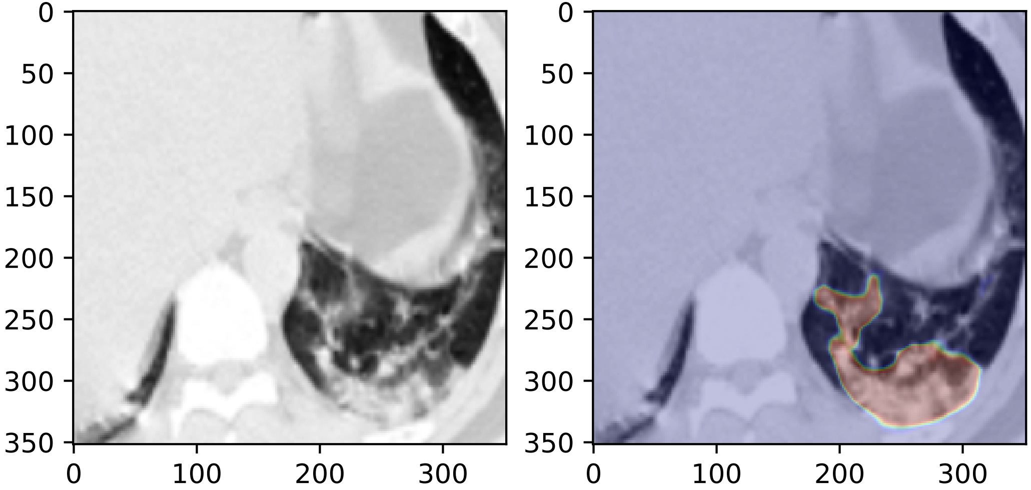

Lesion Proportion: 36.85%

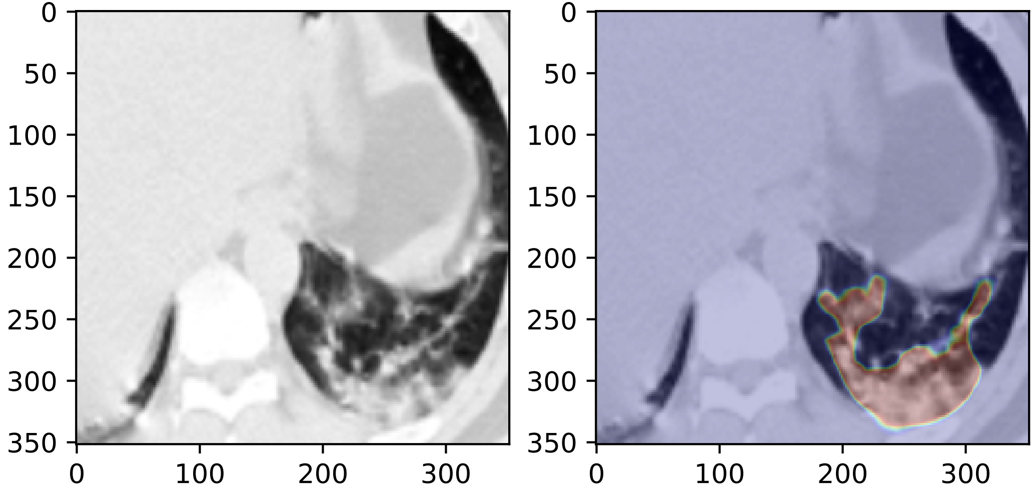

Lesion Proportion: 38.11%

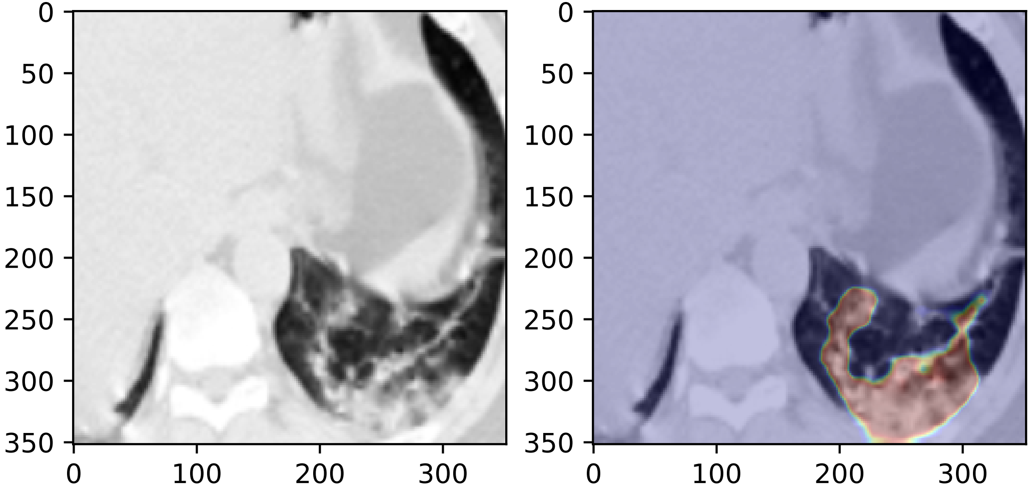

Lesion Proportion: 36.41%

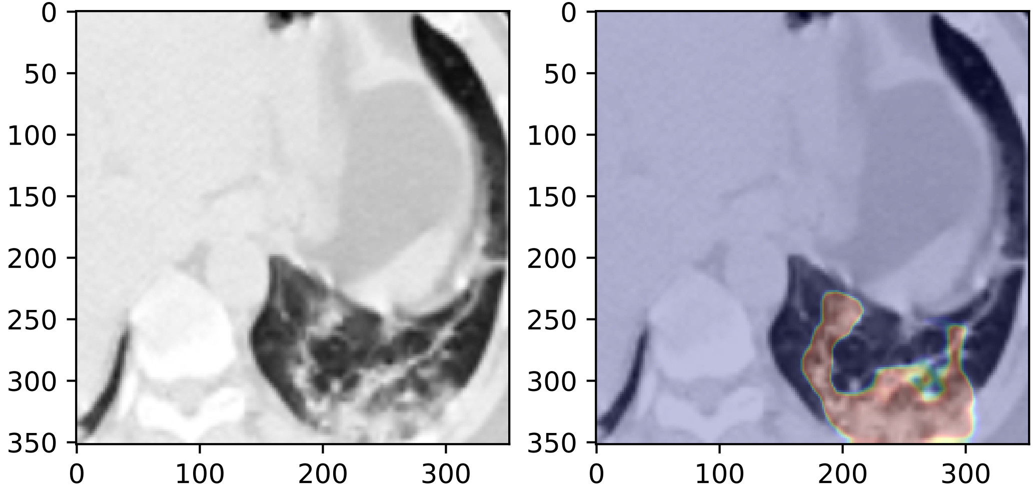

Lesion Proportion: 35.50%

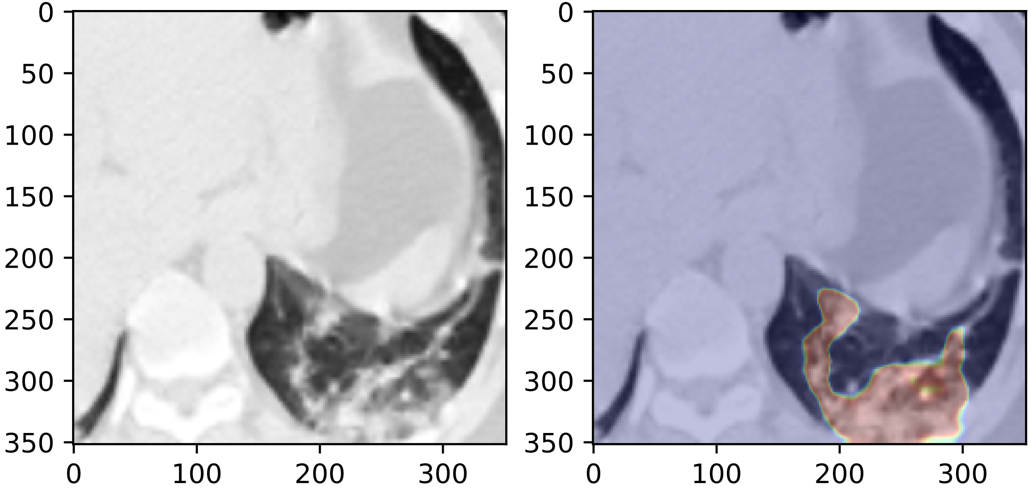

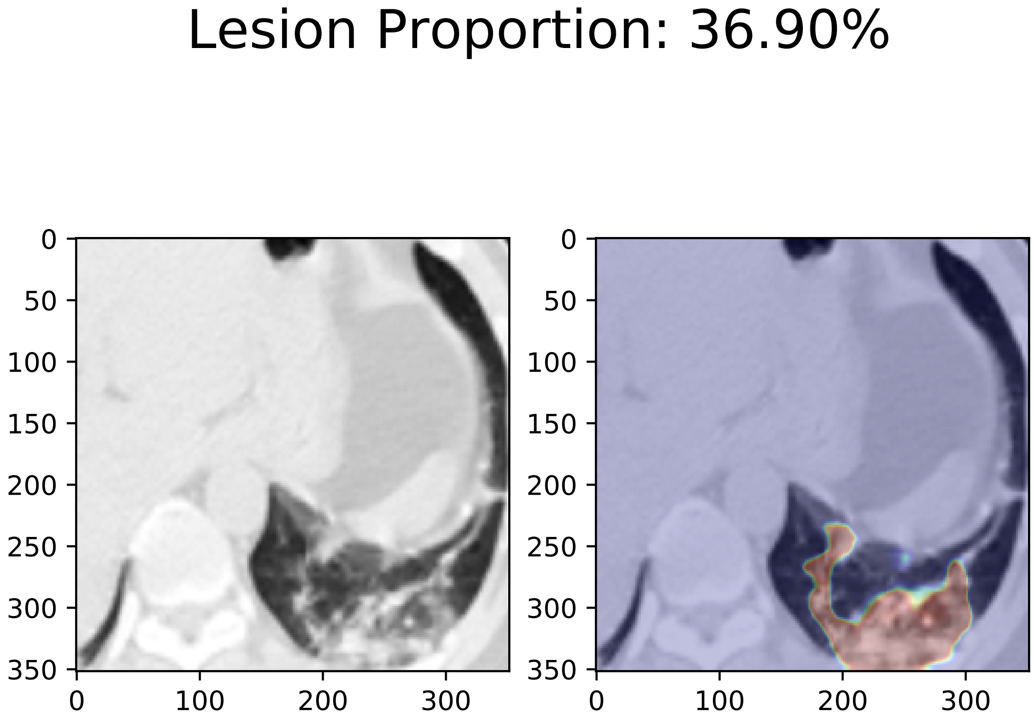
Lesion Proportion: 36.90%
0
50
100
150
200
250
300
350
0
100
200
300
0
50
100
150
200
250
300
350
0
100
200
300

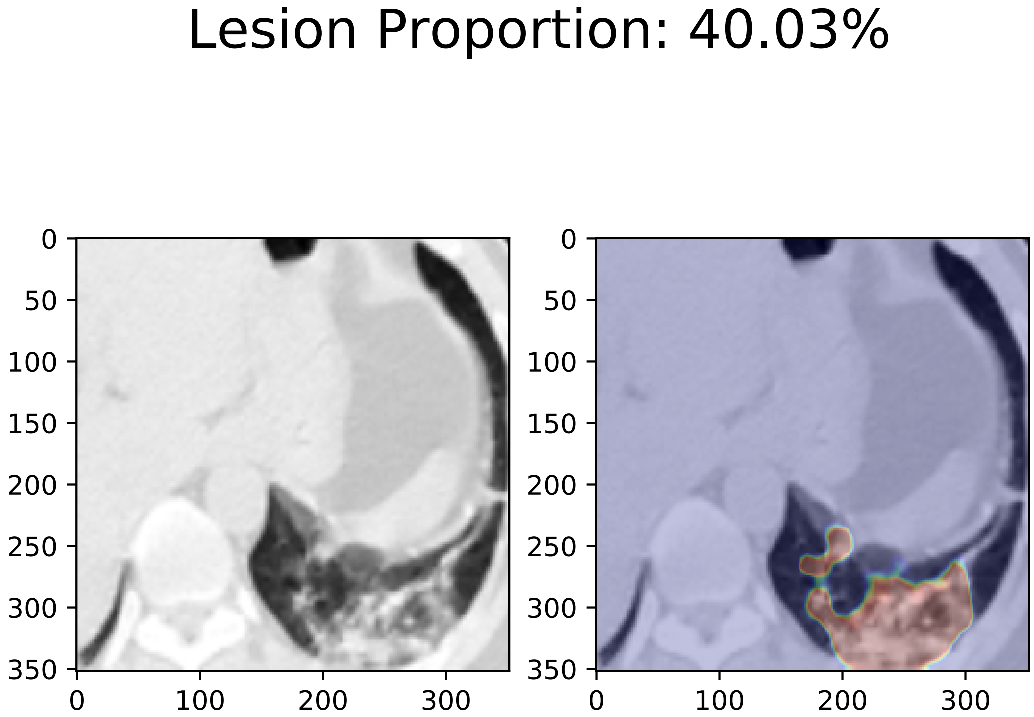
Lesion Proportion: 40.03%
0
50
100
150
200
250
300
350
0
100
200
300

Lesion Proportion: 40.66%

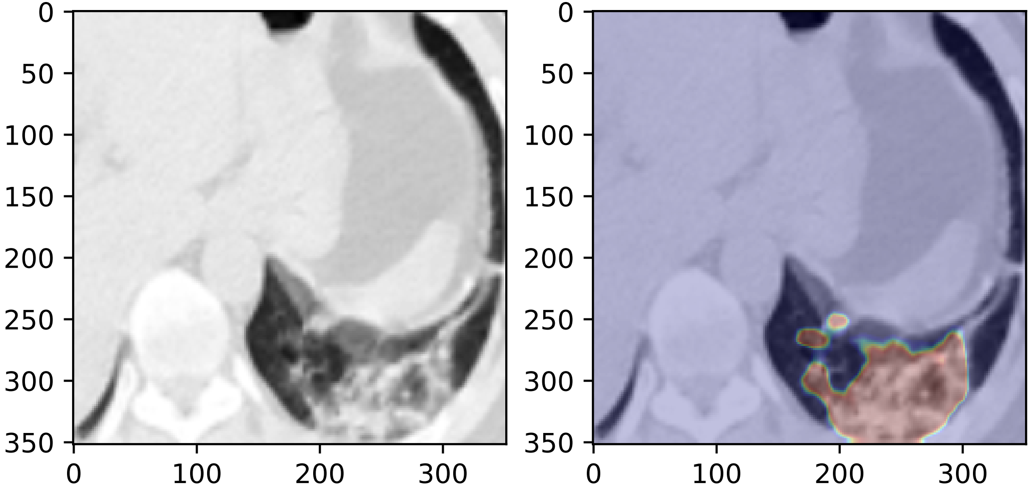

Lesion Proportion: 43.28%

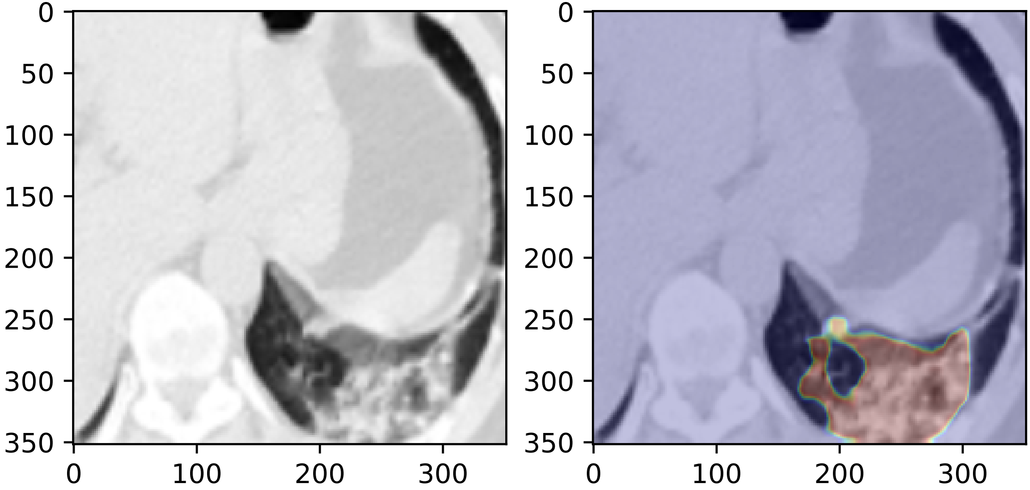

Lesion Proportion: 45.22%

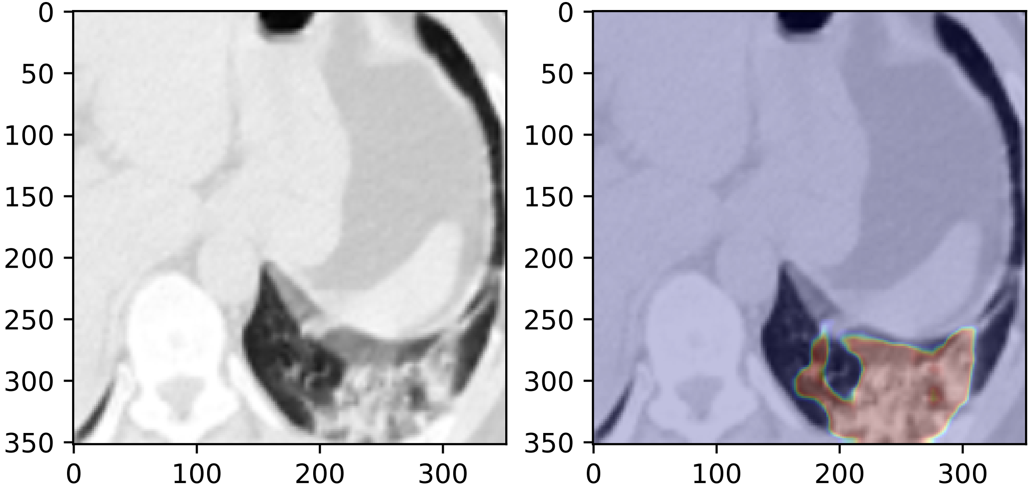

Lesion Proportion: 48.24%

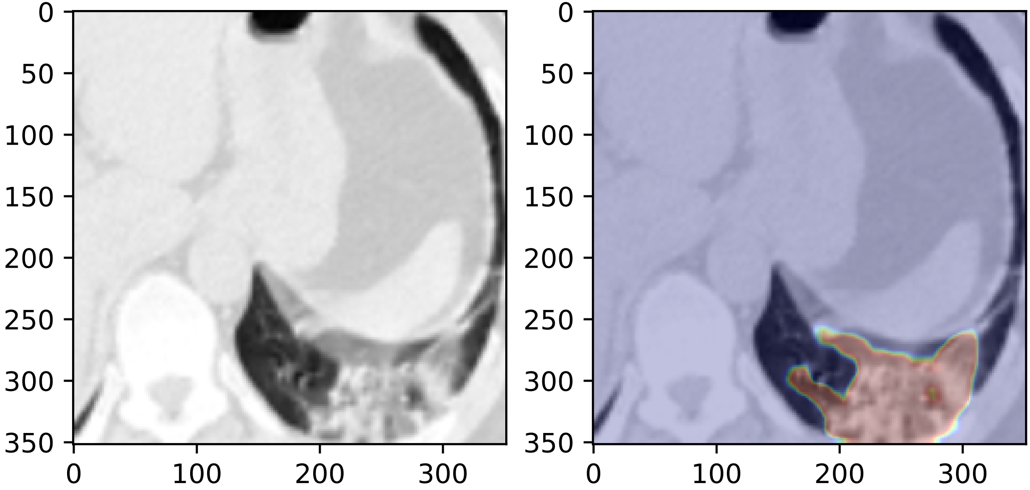

Lesion Proportion: 41.89%

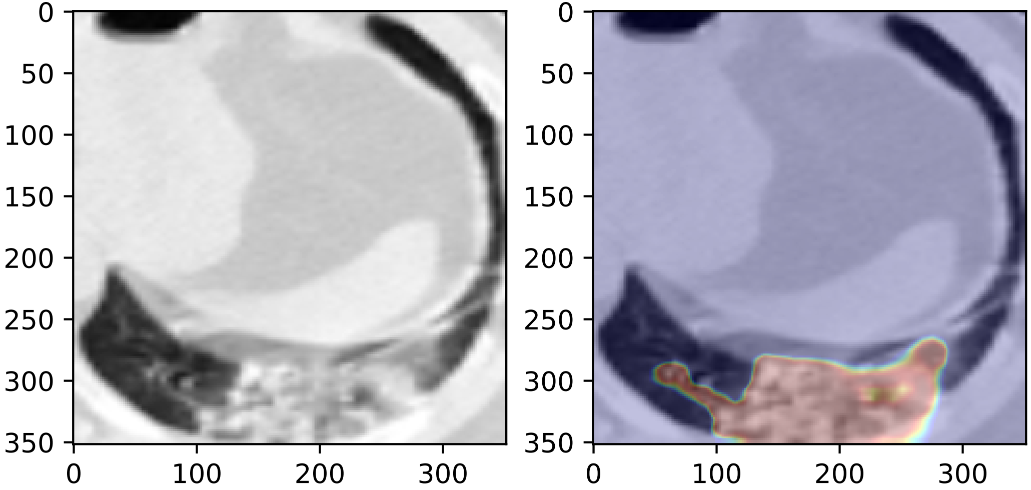

Lesion Proportion: 42.34%

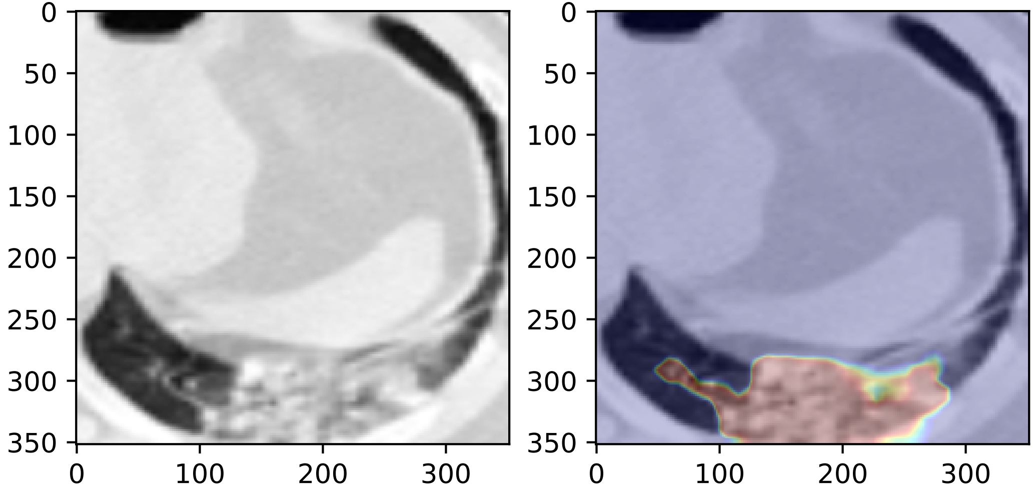

Lesion Proportion: 47.57%

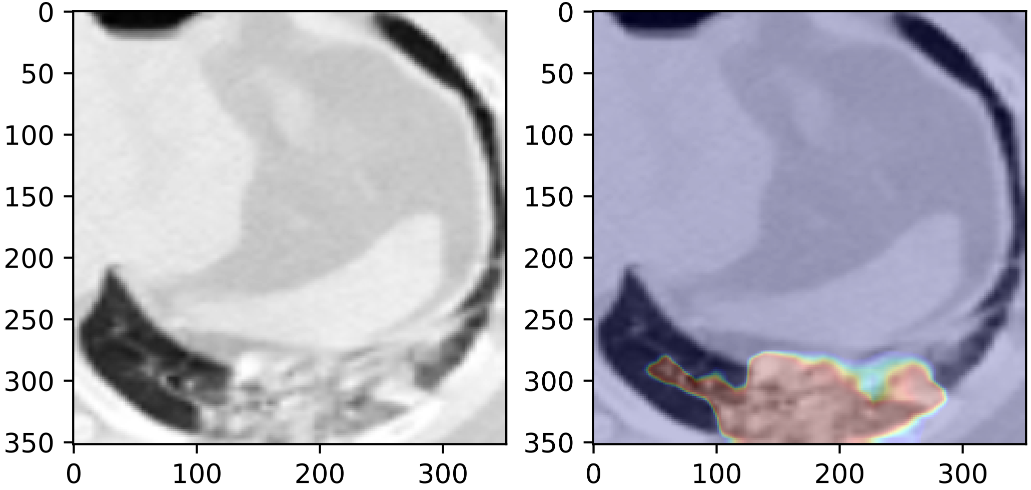

Lesion Proportion: 49.66%

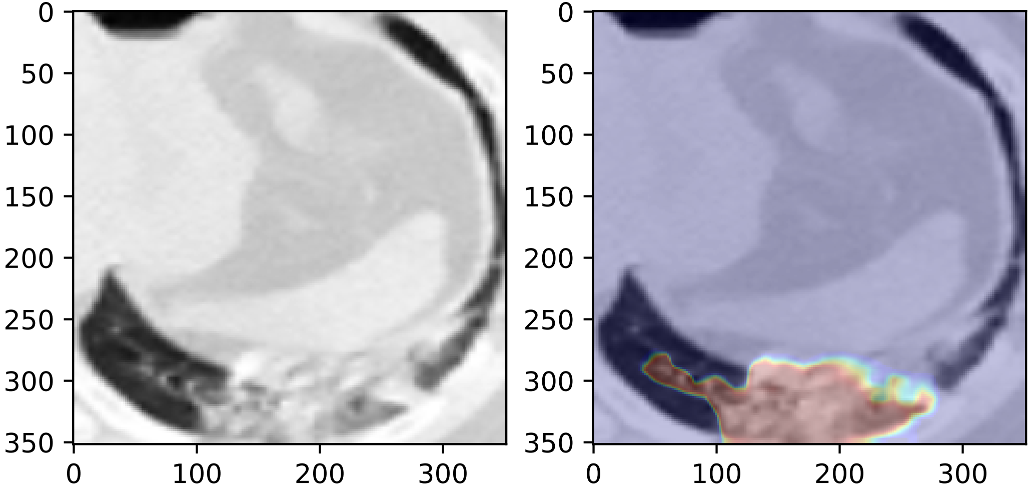

Lesion Proportion: 50.63%

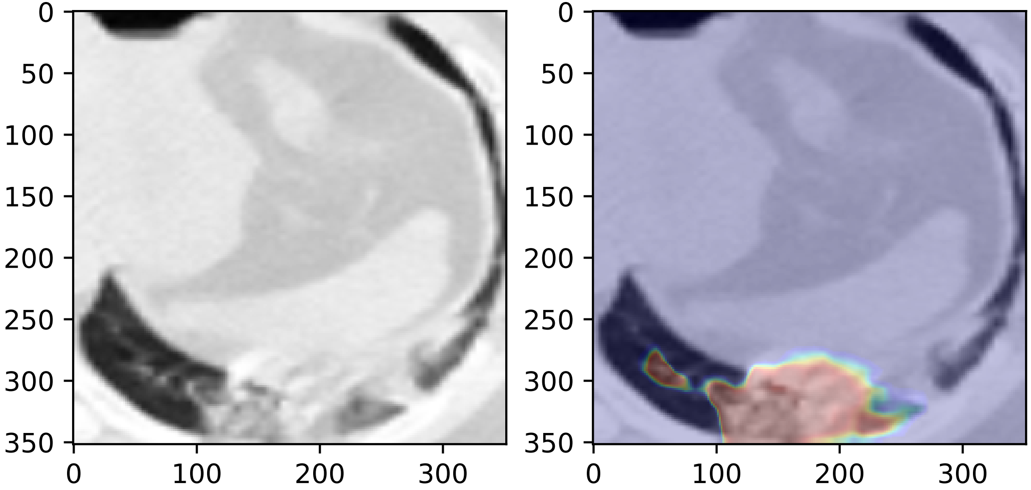

Lesion Proportion: 48.93%

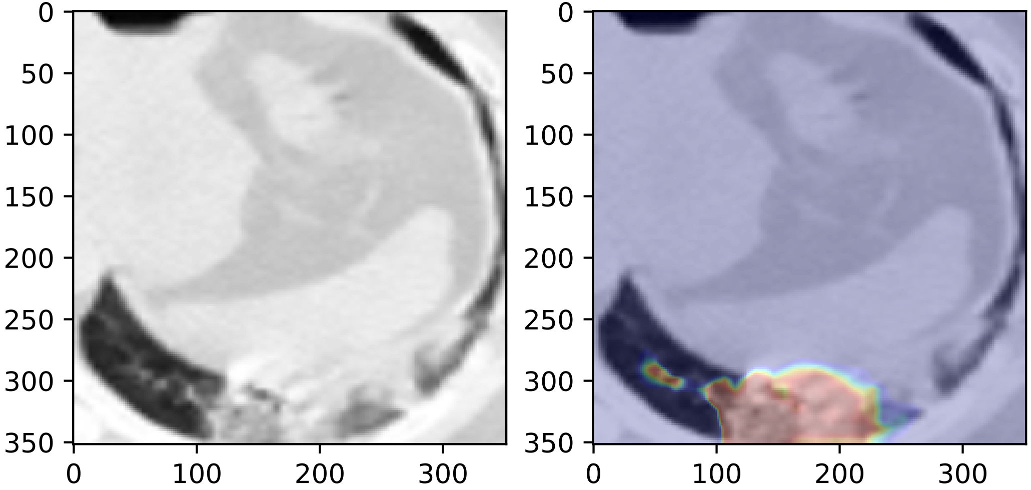

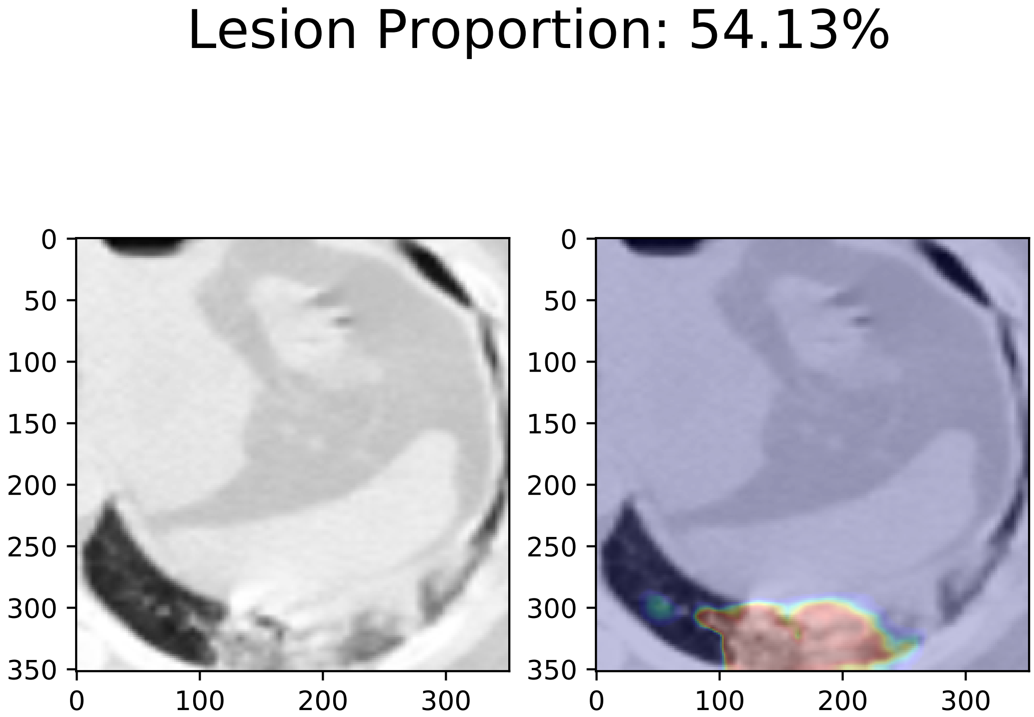
Lesion Proportion: 54.13%
0
50
100
150
200
250
300
350
0
100
200
300

Lesion Proportion: 52.24%

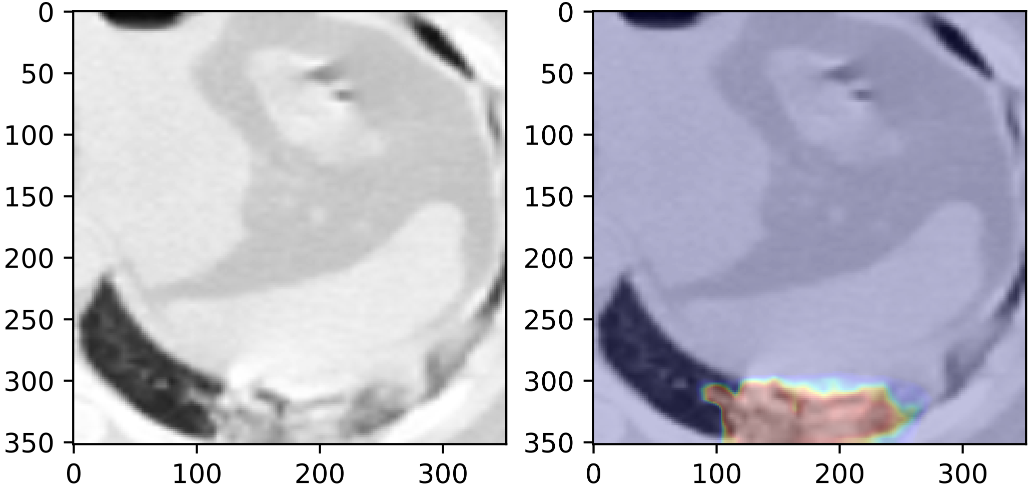

Lesion Proportion: 47.87%

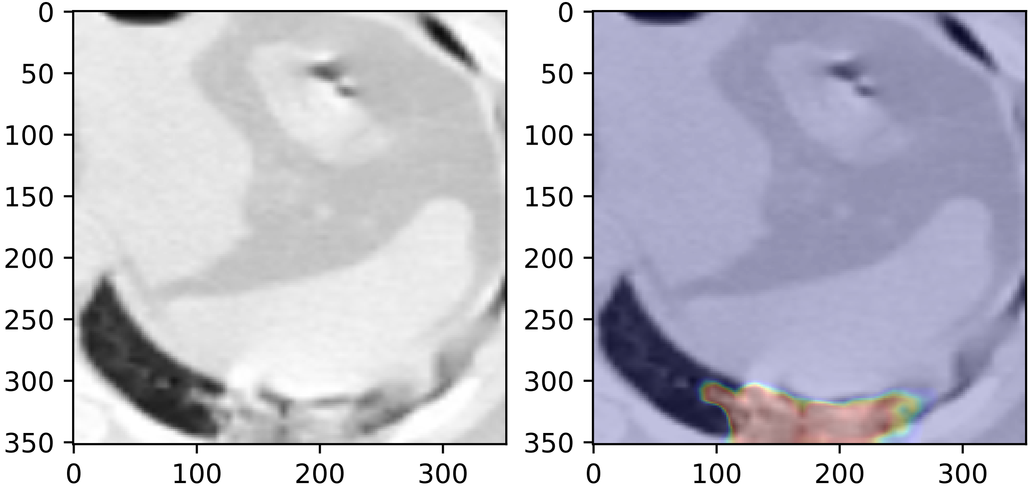

Lesion Proportion: 47.57%

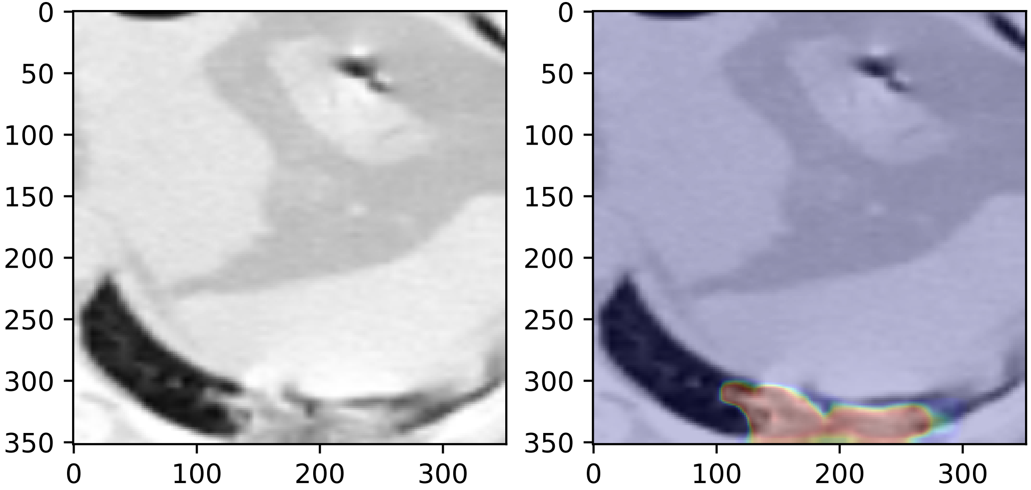

Lesion Proportion: 14.86%

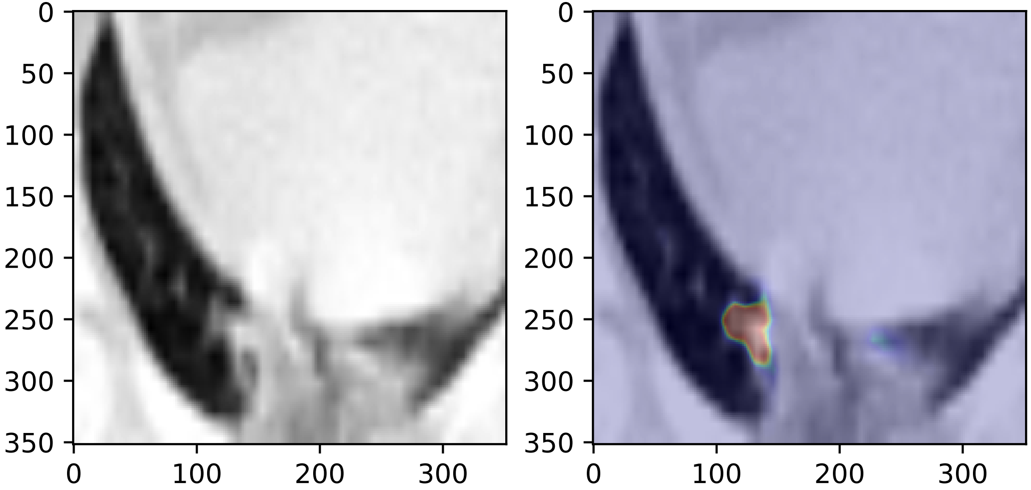

Lesion Proportion: 19.01%

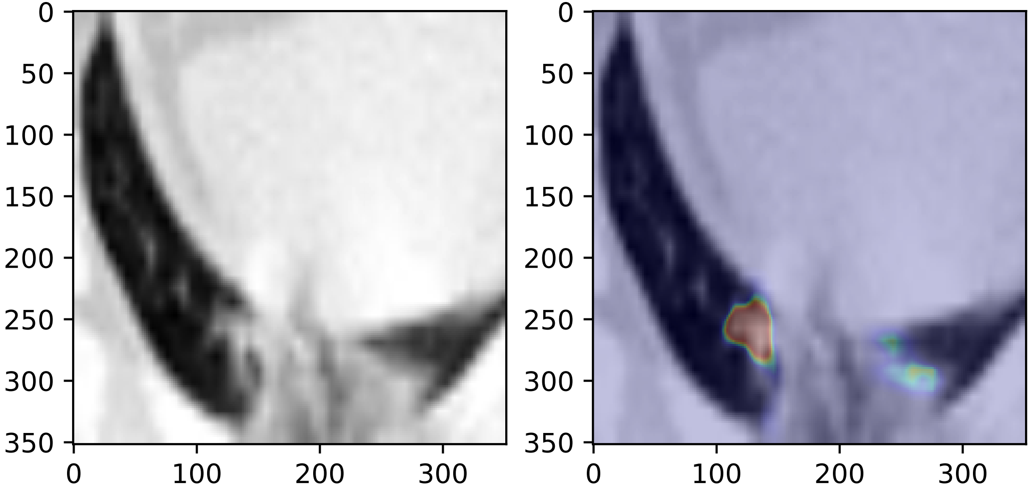

Lesion Proportion: 19.08%

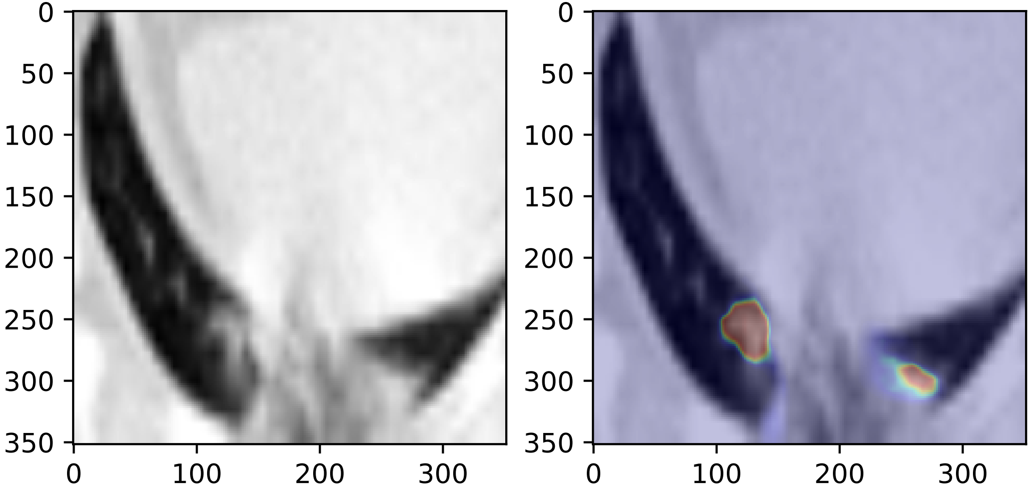

Lesion Proportion: 17.59%

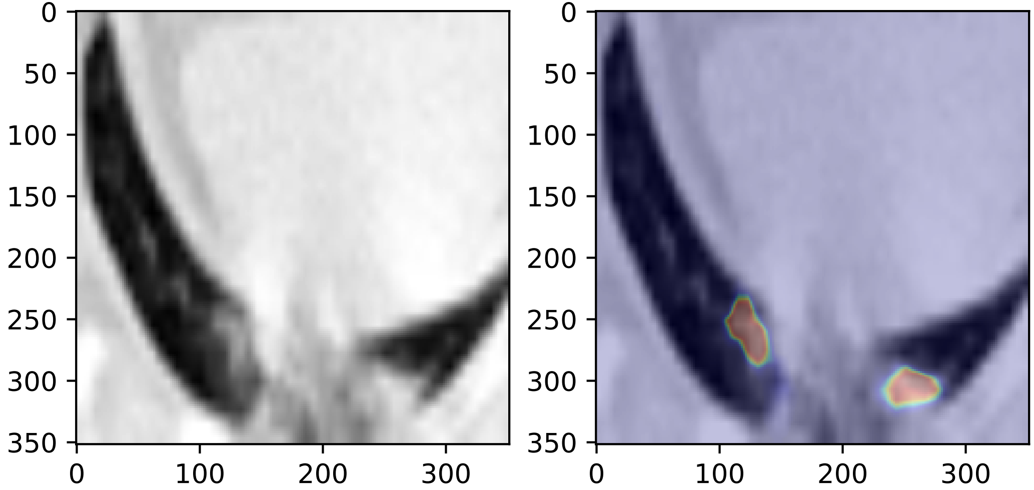

Lesion Proportion: 24.07%

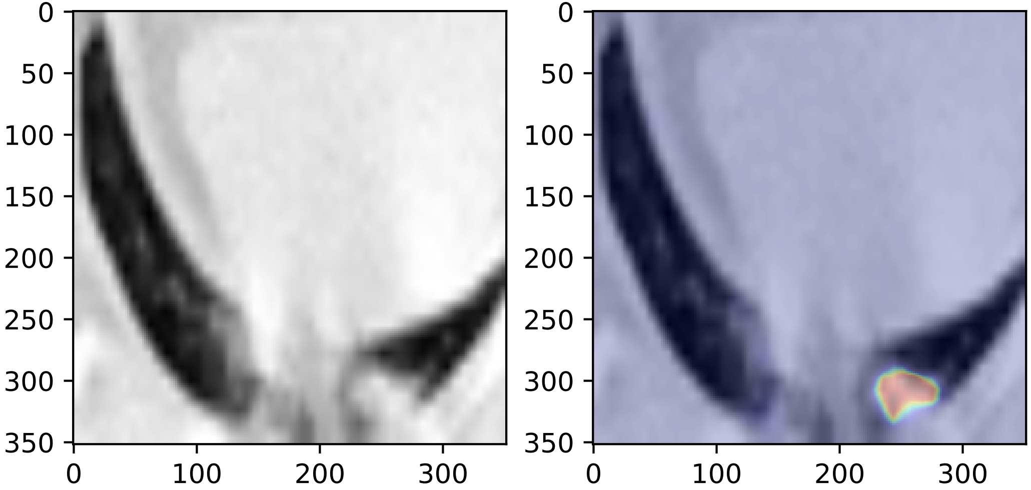

Lesion Proportion: 31.09%

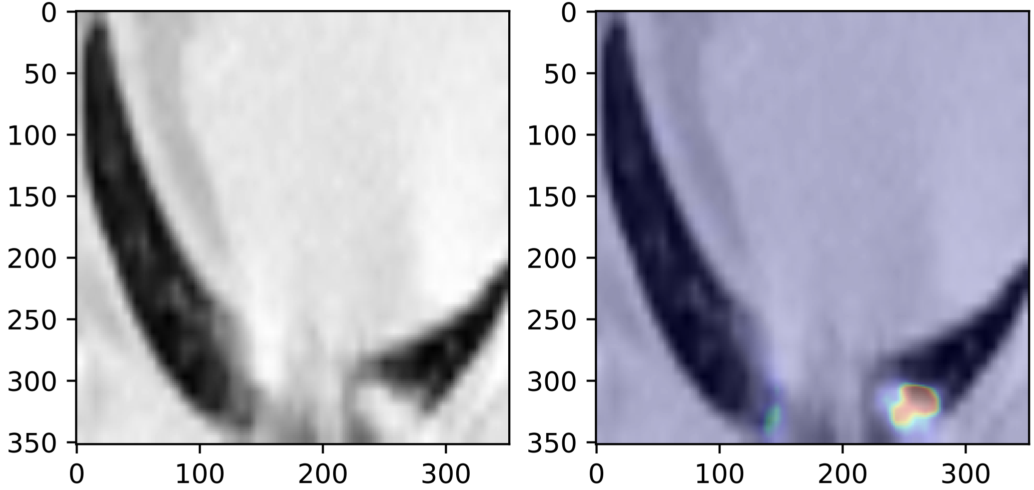

Lesion Proportion: 36.99%

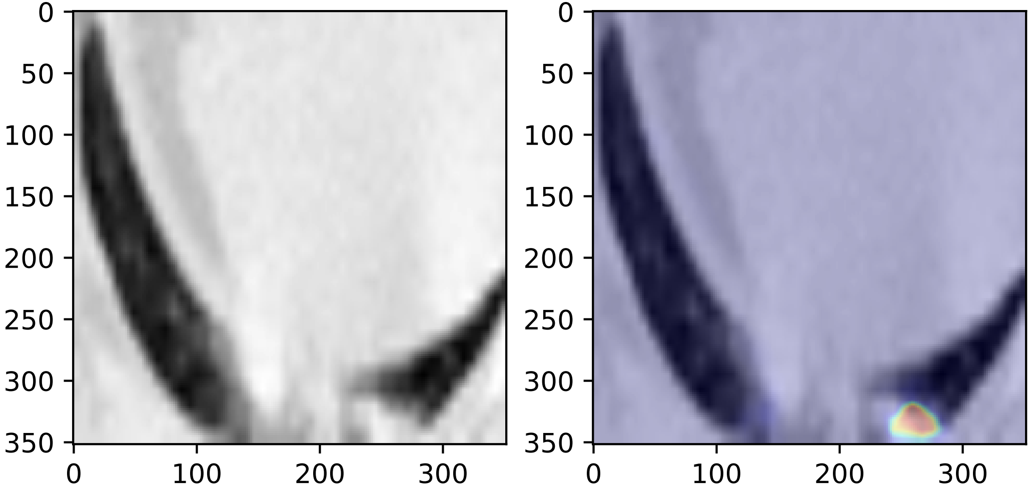

Lesion Proportion: 53.87%

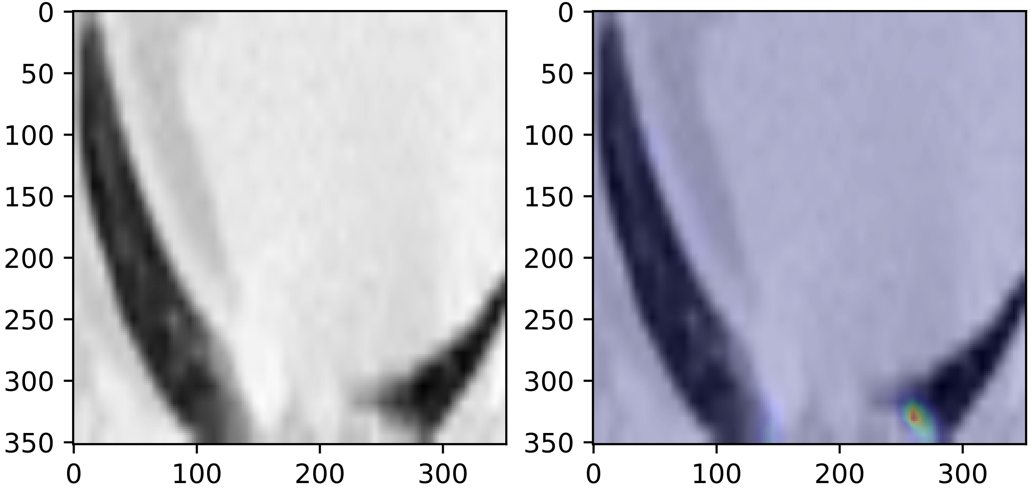

Lesion Proportion: 66.99%

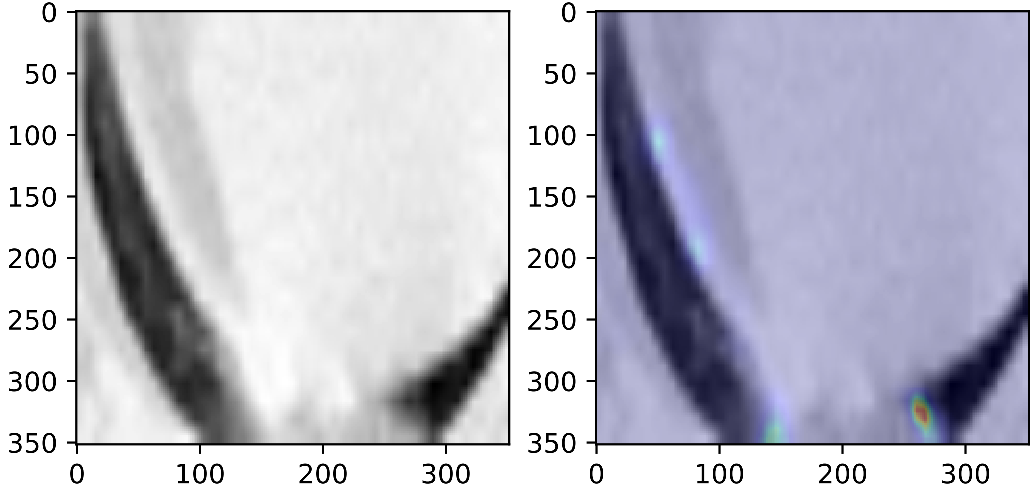

Lesion Proportion: 62.37%

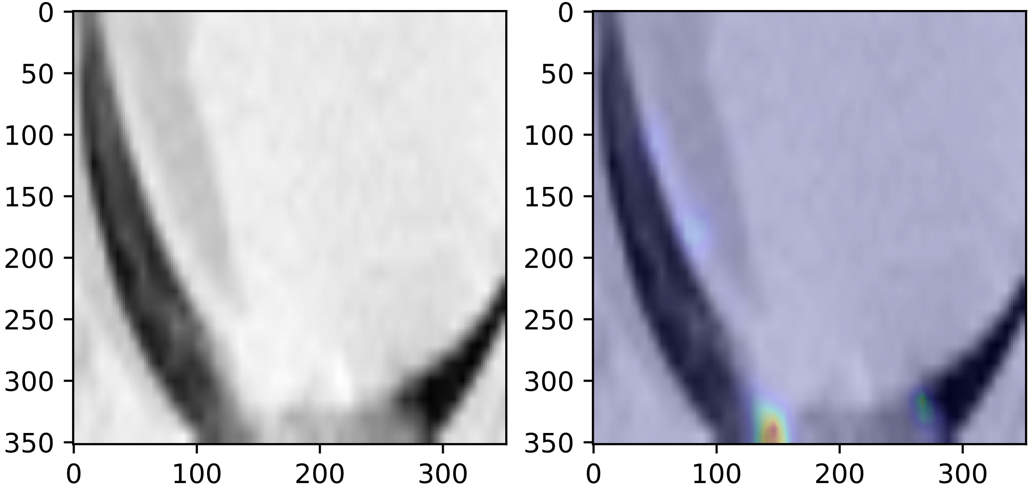

Lesion Proportion: 96.10%

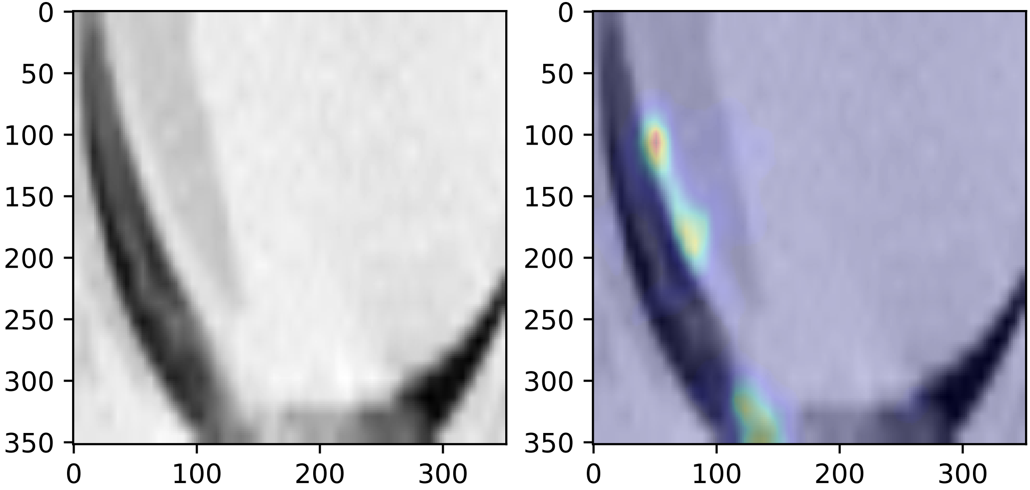

Lesion Proportion: 98.14%

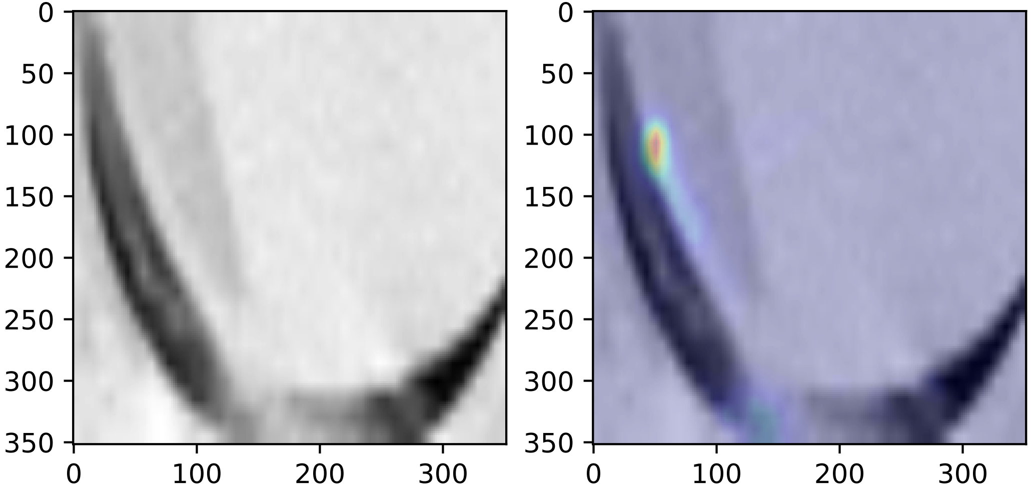

Lesion Proportion: 99.61%

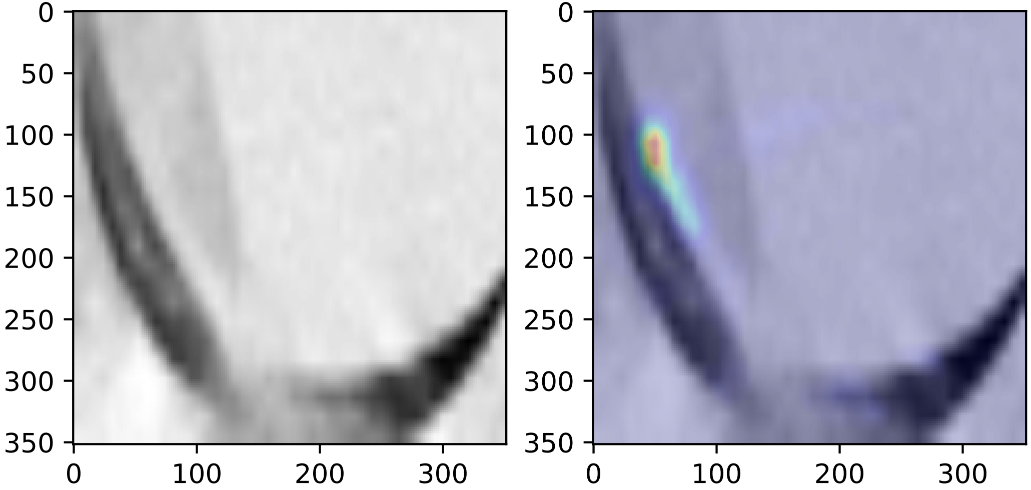

Lesion Proportion: 93.00%

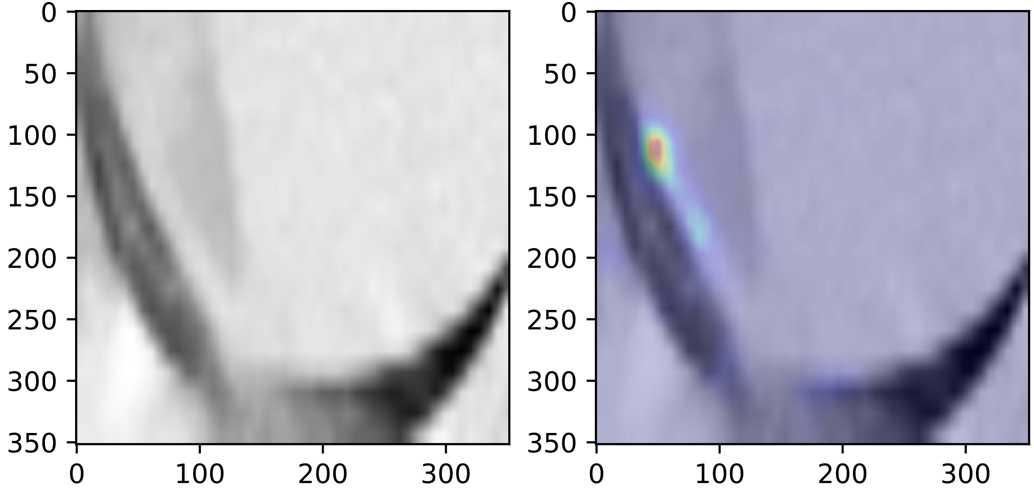

Lesion Proportion: 95.14%

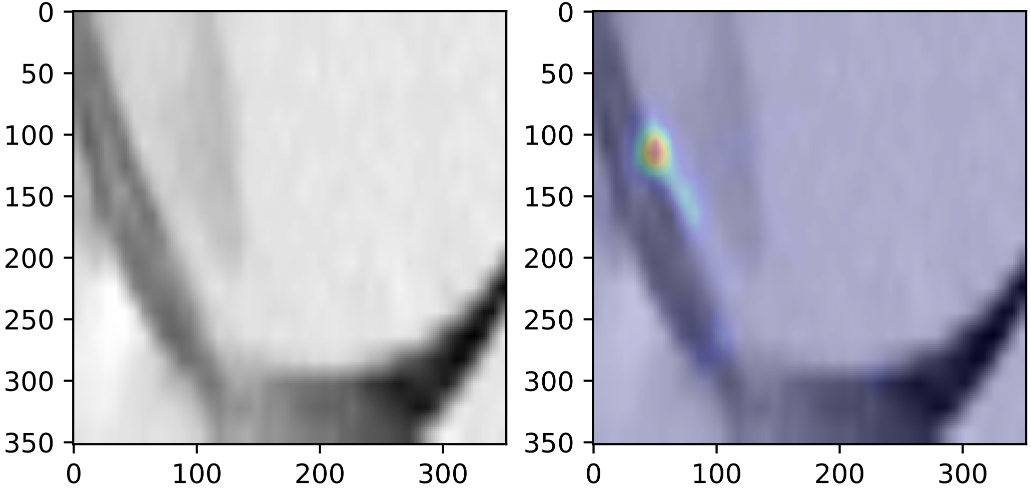

Lesion Proportion: 5.89%

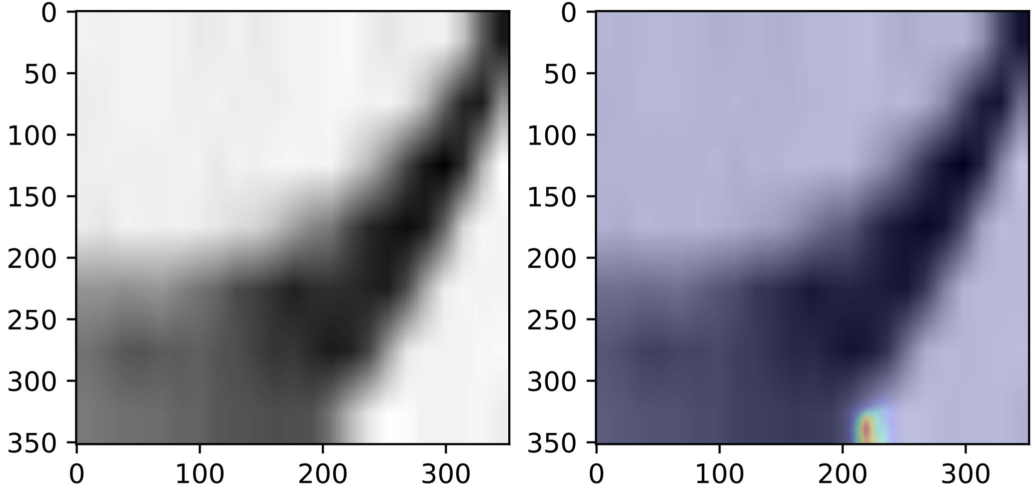

Lesion Proportion: 9.62%

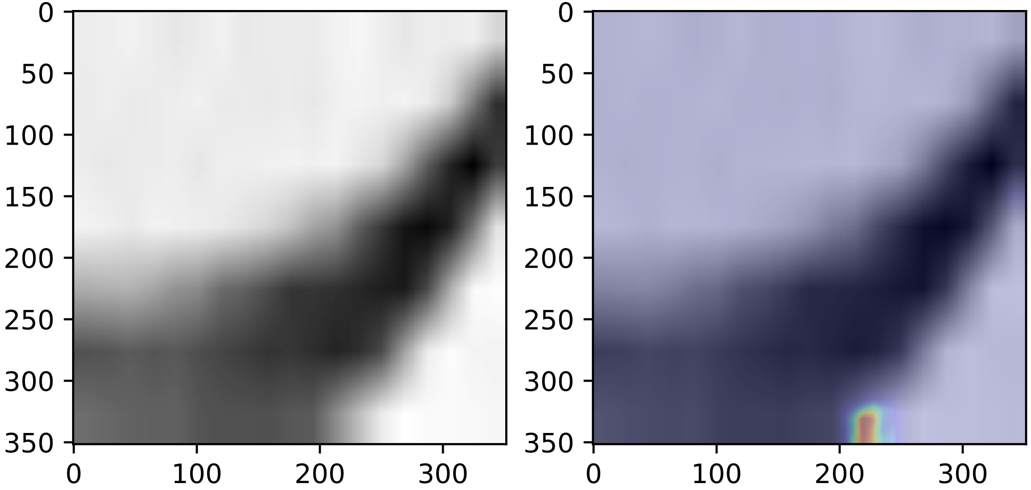

Lesion Proportion: 32.43%

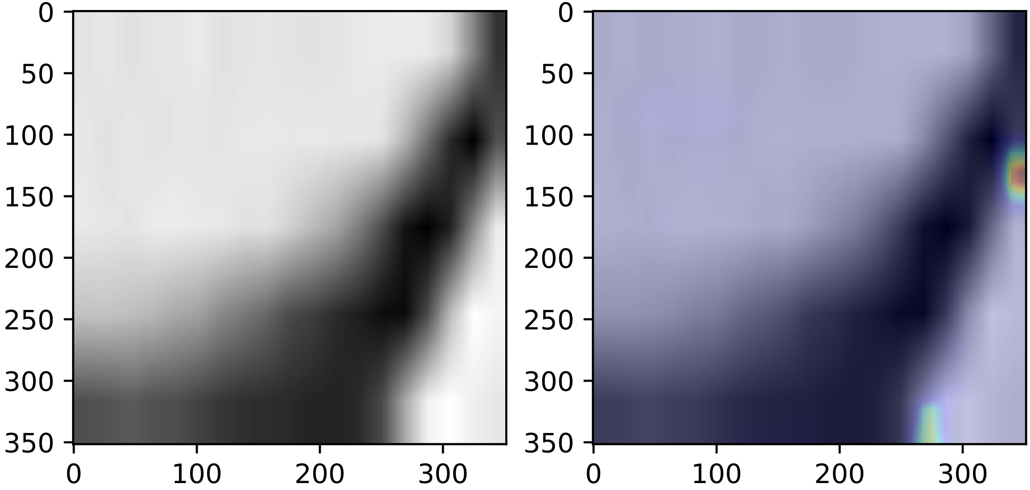

Lesion Proportion: 51.98%

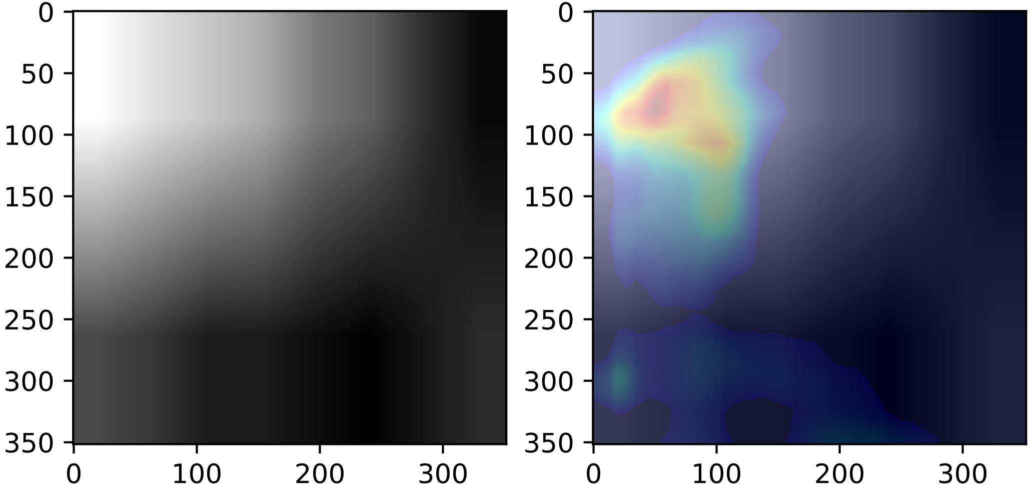

Supplement: Supplementary file 6 [file DataSheet6.pdf]
