# Supplementary material for: Medical decision support system using weakly-labeled lung CT scans
Source: Front Med Technol. 2022 Sep 28;4:980735. doi: 10.3389/fmedt.2022.980735 (PMC9554434; doi:10.3389/fmedt.2022.980735)
Supplement: Supplementary file 7 [file DataSheet7.pdf]

Lesion Proportion: 42.39%

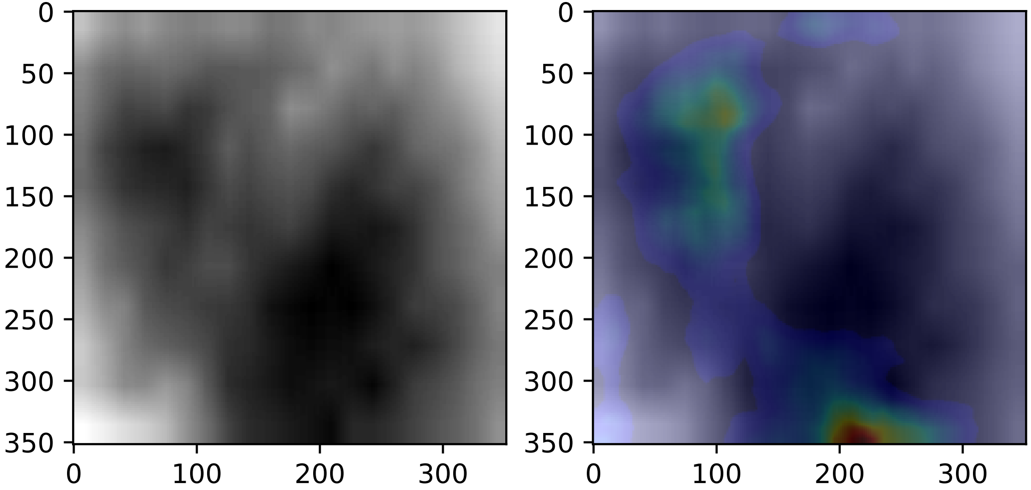

Lesion Proportion: 5.88%

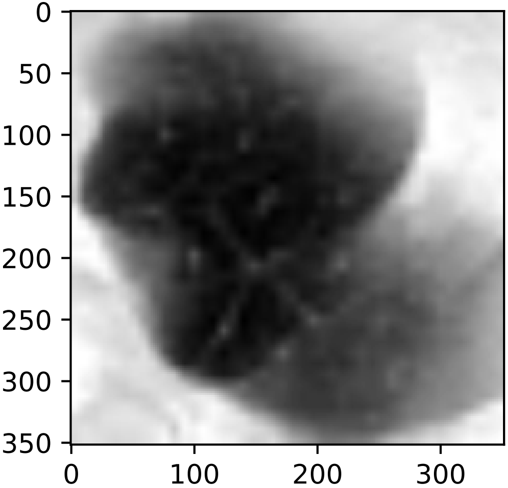

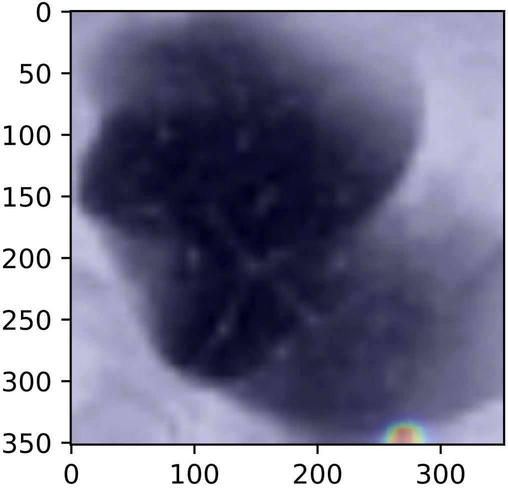

Lesion Proportion: 11.61%

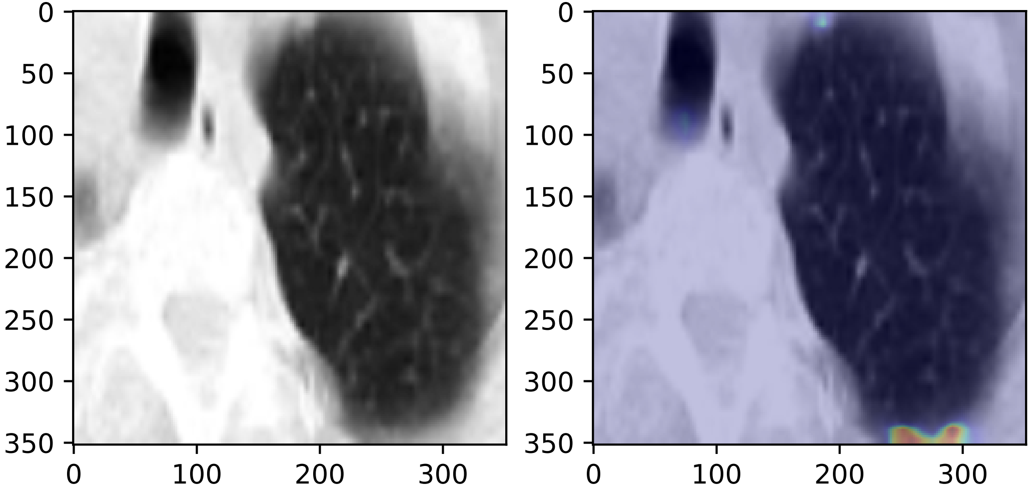

Lesion Proportion: 9.29%

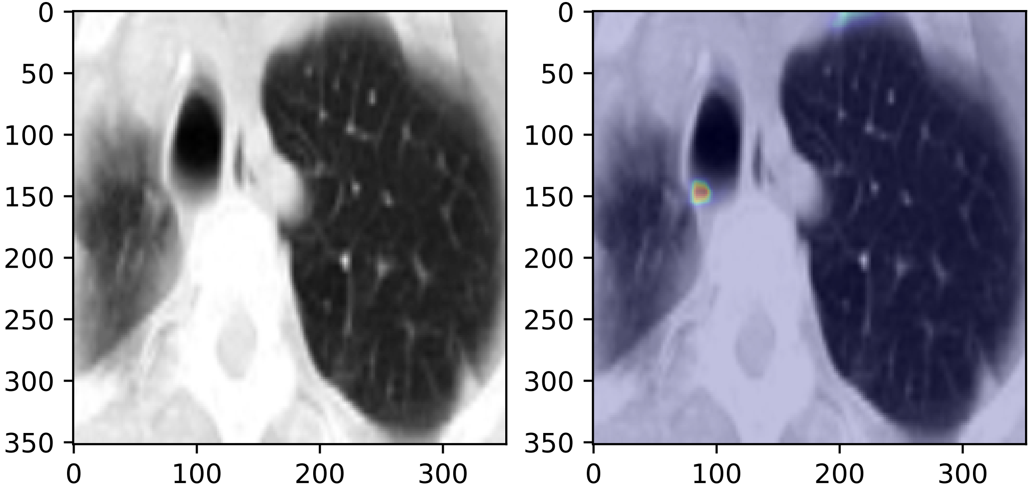

Lesion Proportion: 11.52%

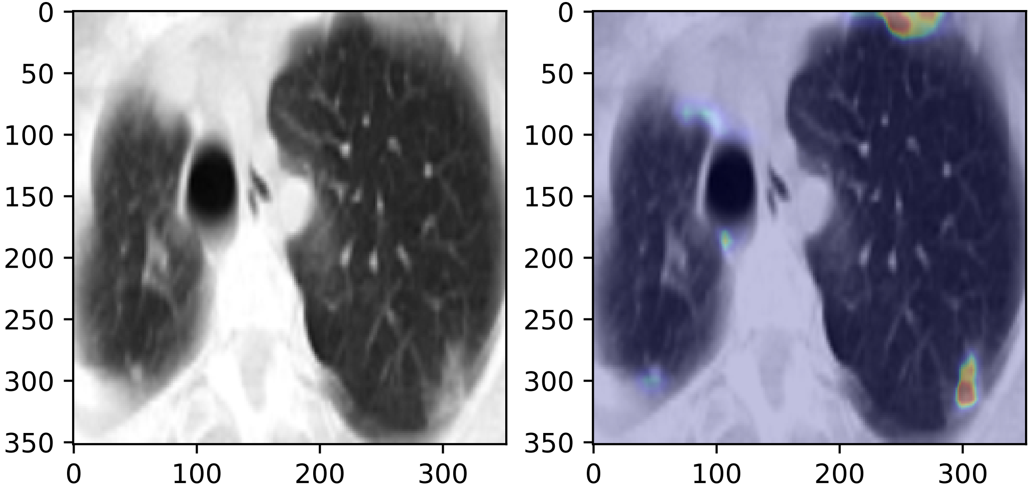

Lesion Proportion: 11.73%

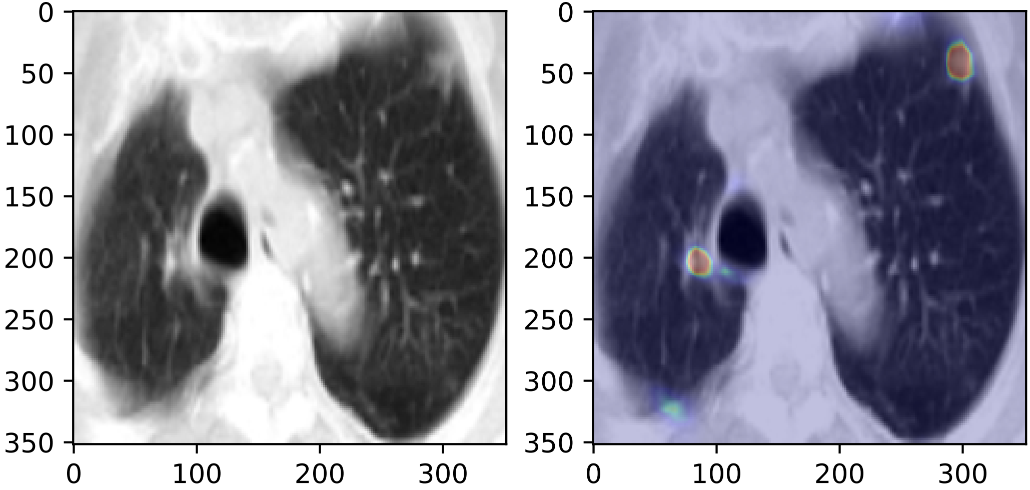

Lesion Proportion: 12.77%

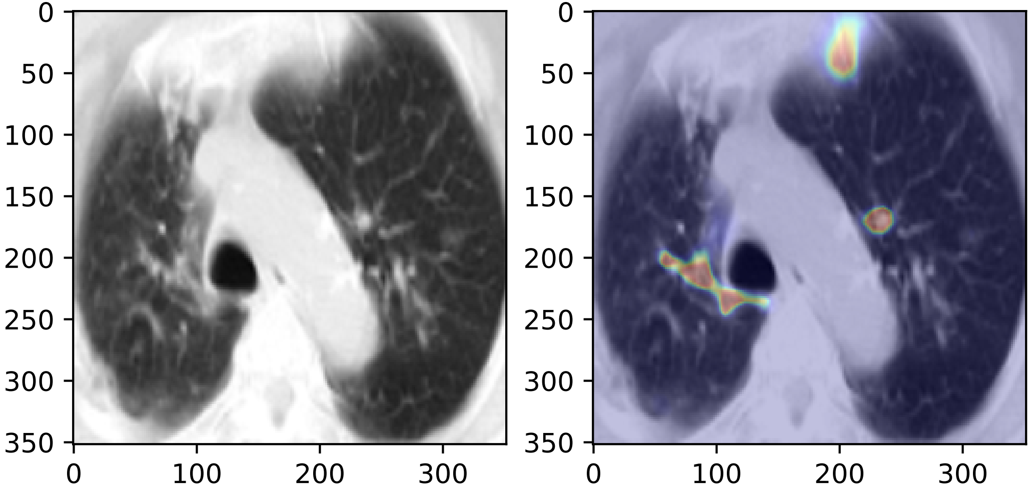

Lesion Proportion: 15.03%

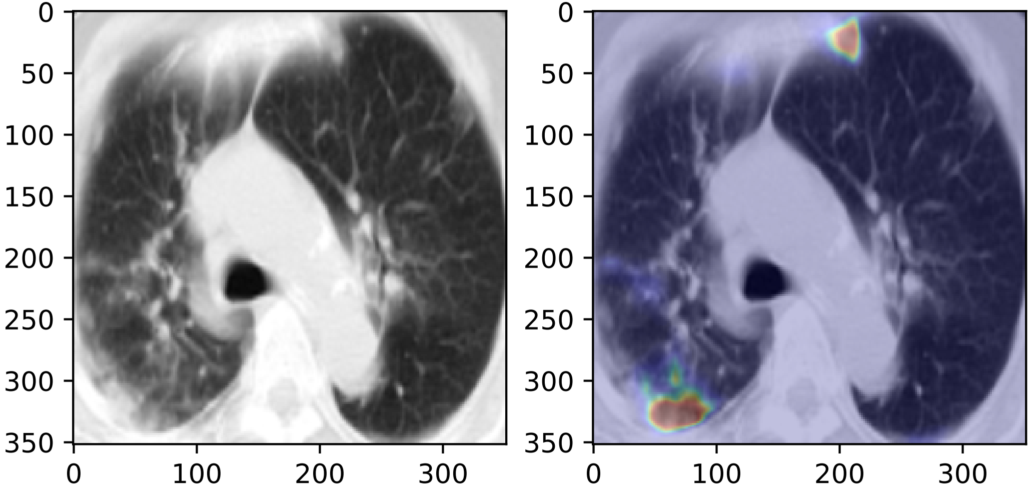

Lesion Proportion: 17.23%

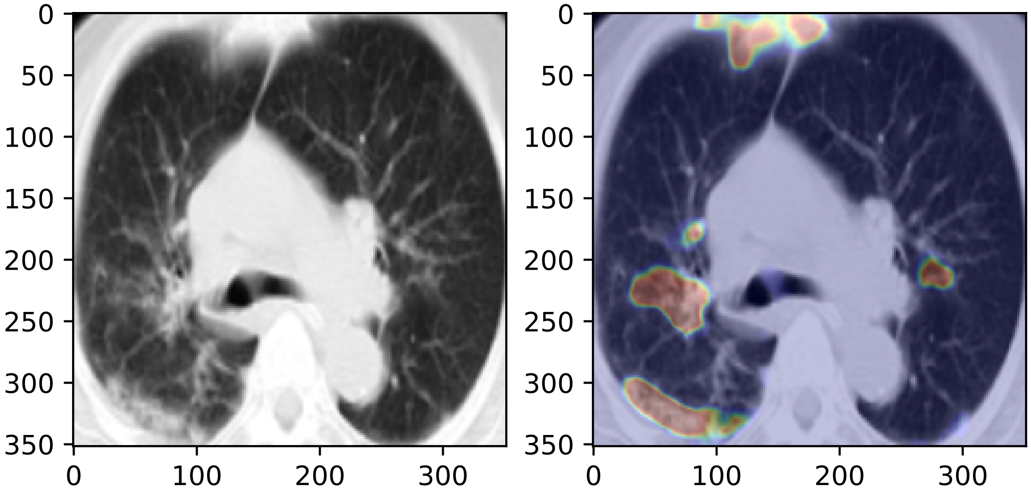

Lesion Proportion: 15.97%

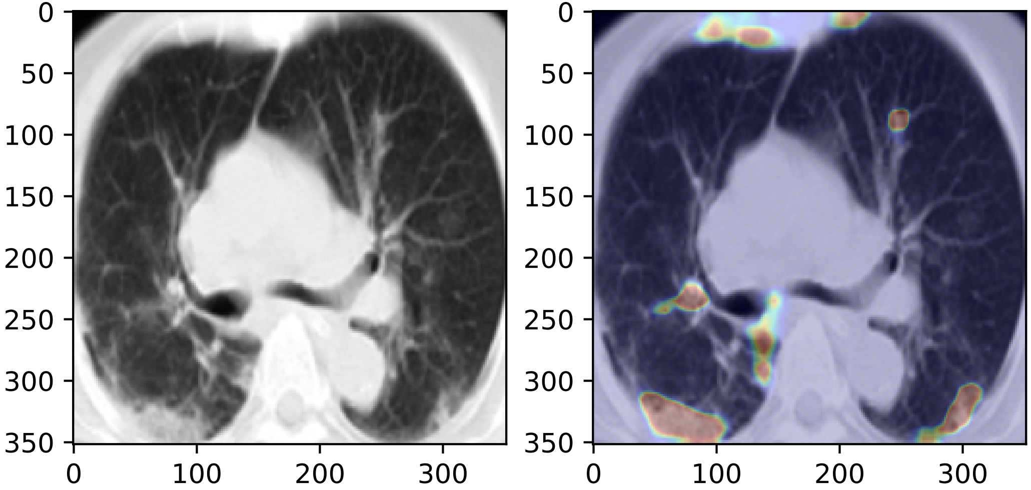

Lesion Proportion: 14.08%

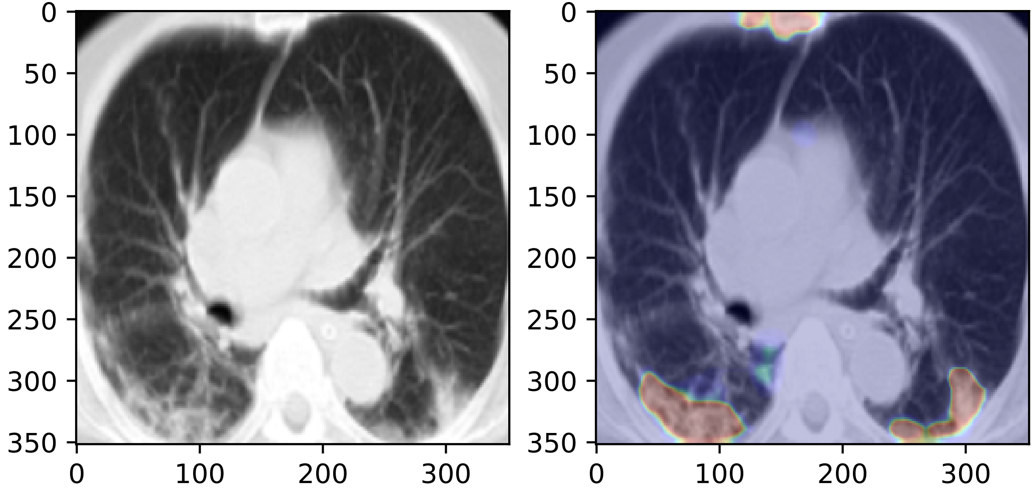

Lesion Proportion: 15.04%

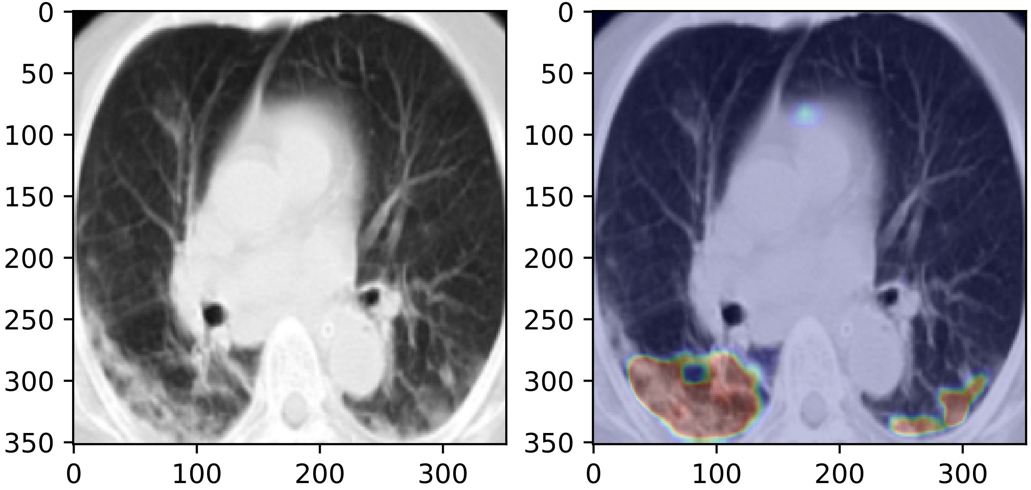

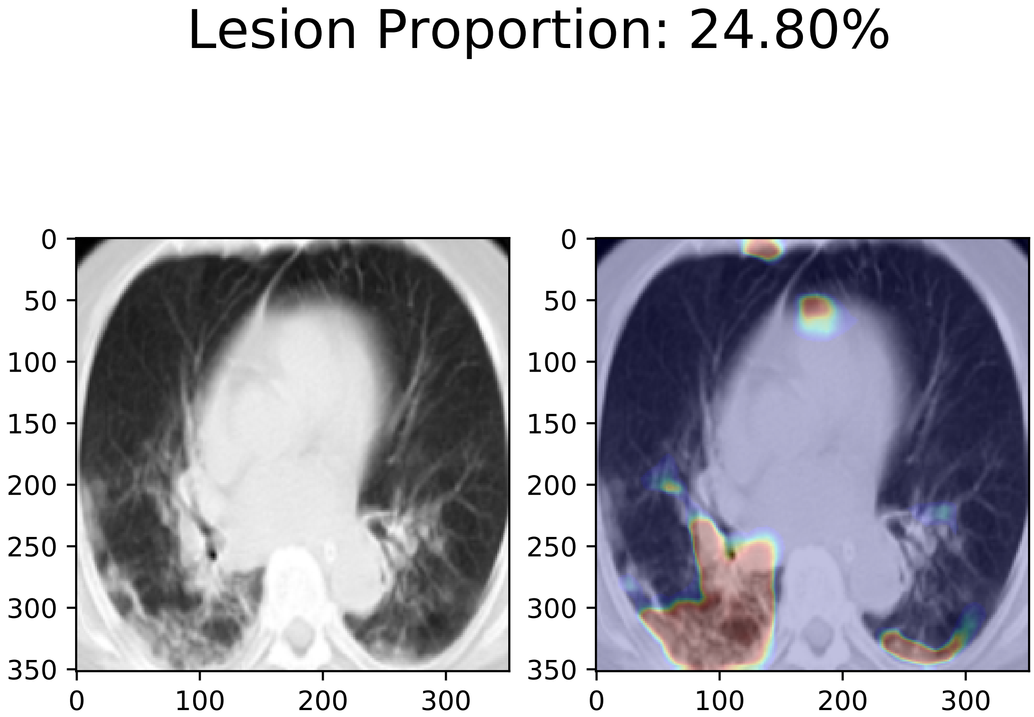

Lesion Proportion: 24.80%
0
50
100
150
200
250
300
350
0
100
200
300

Lesion Proportion: 27.91%

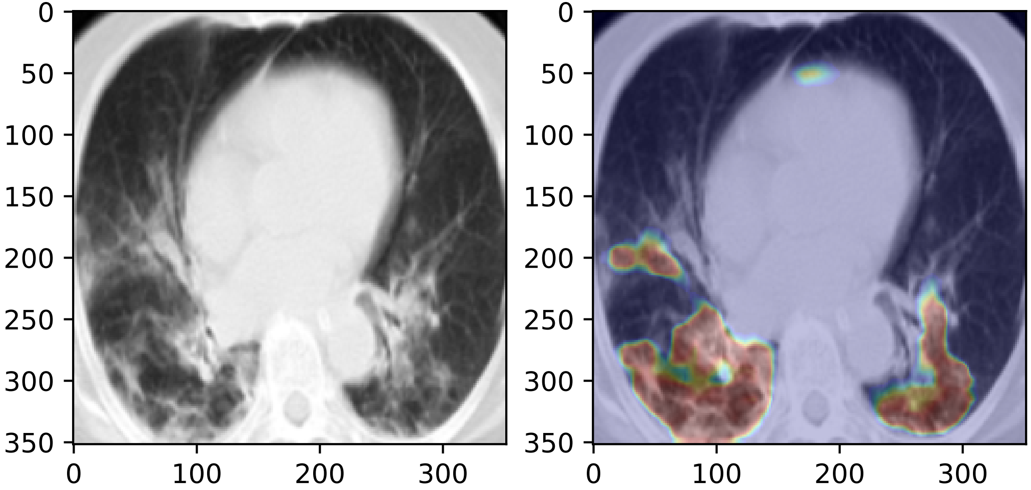

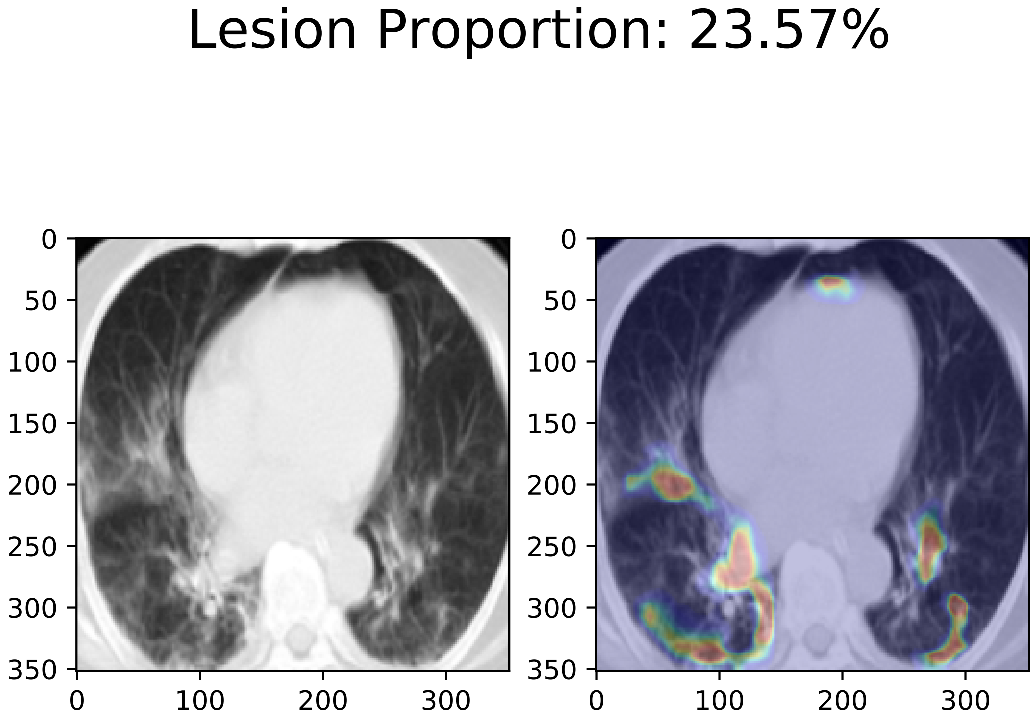
Lesion Proportion: 23.57%
0
50
100
150
200
250
300
350
0
100
200
300

Lesion Proportion: 30.97%

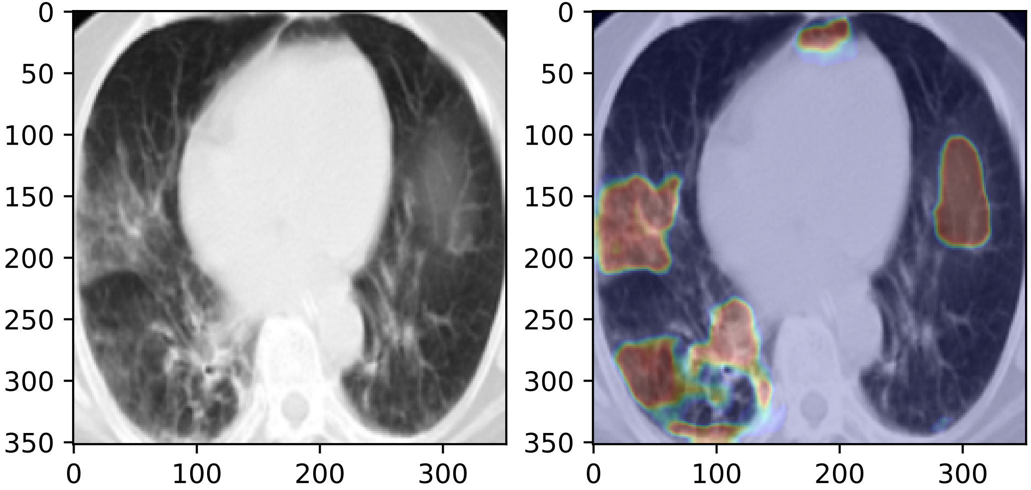

Lesion Proportion: 37.57%

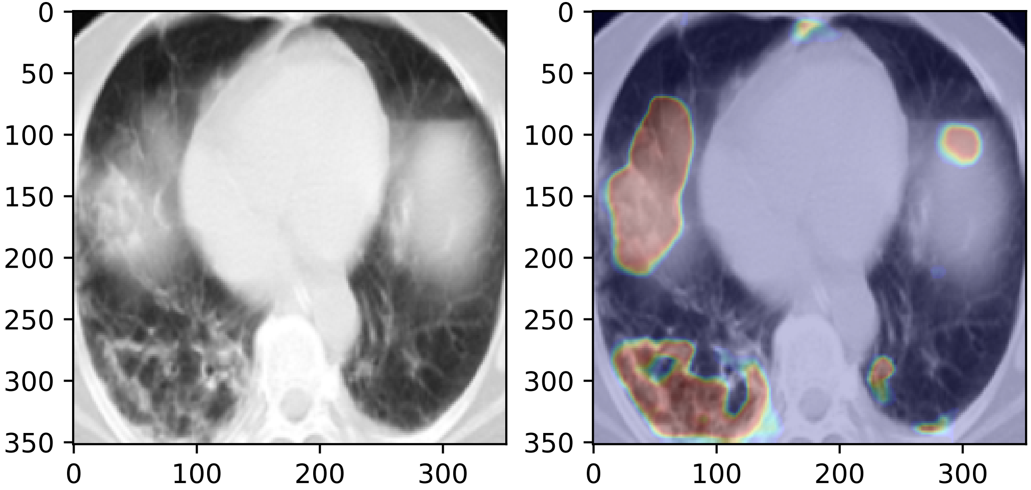

Lesion Proportion: 54.83%

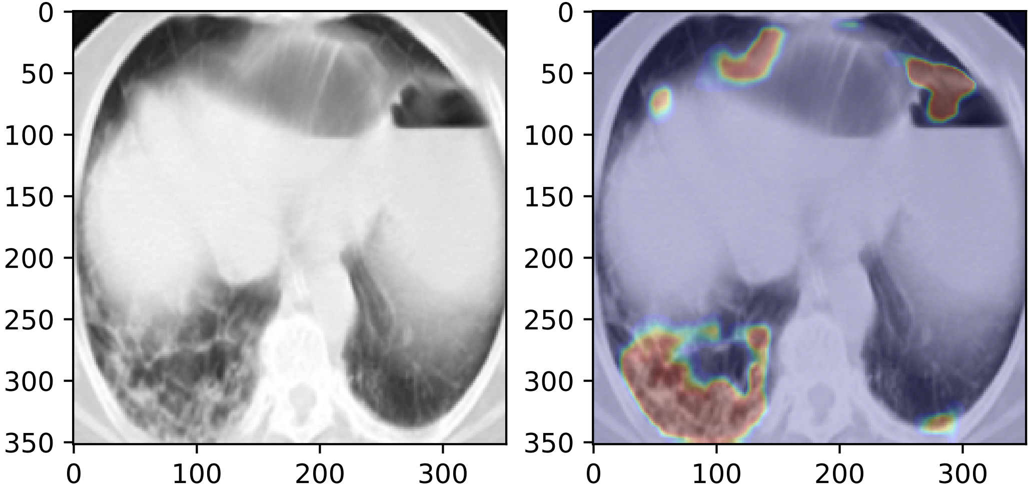

Lesion Proportion: 79.97%

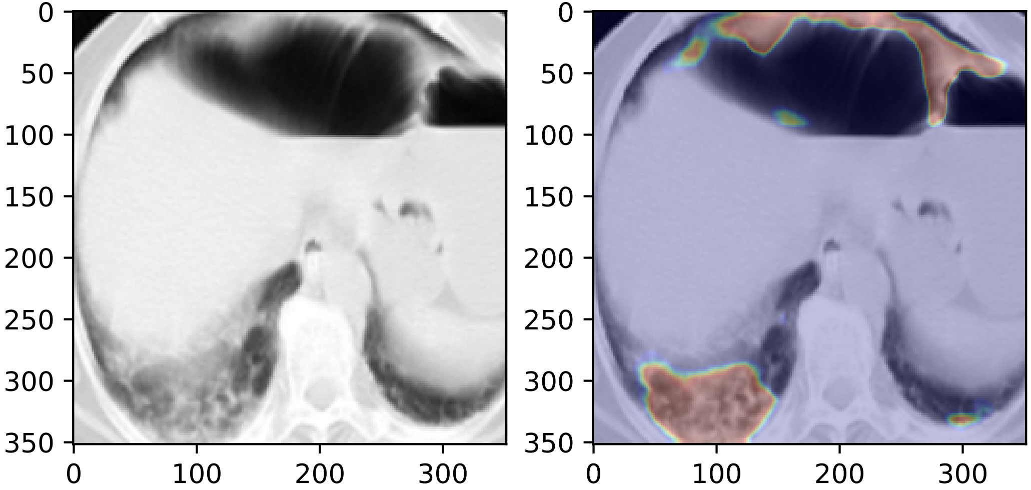

Lesion Proportion: 90.70%

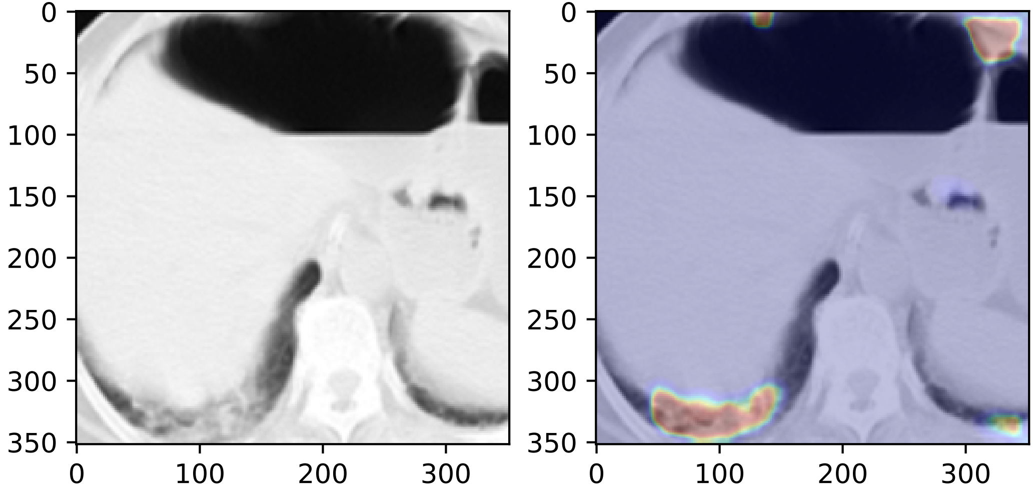

Lesion Proportion: 168.31%

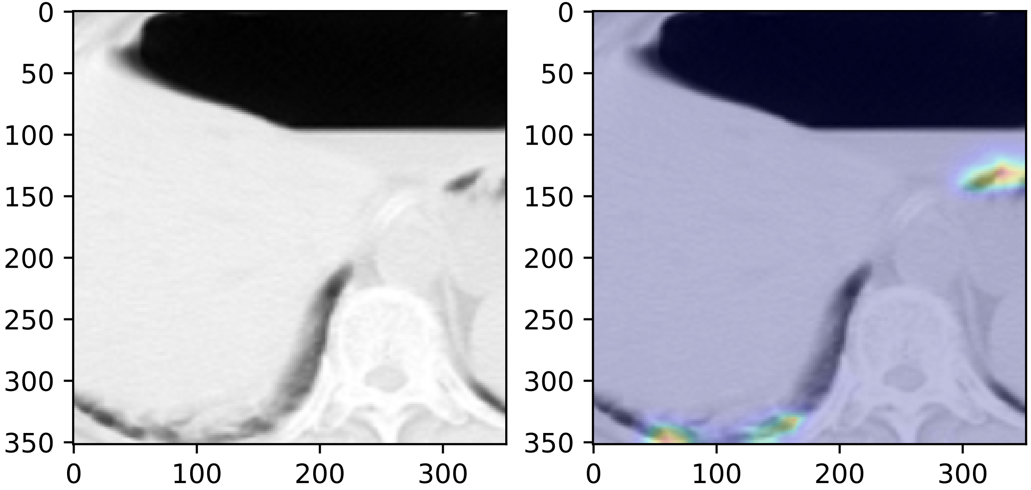

Lesion Proportion: 117.63%

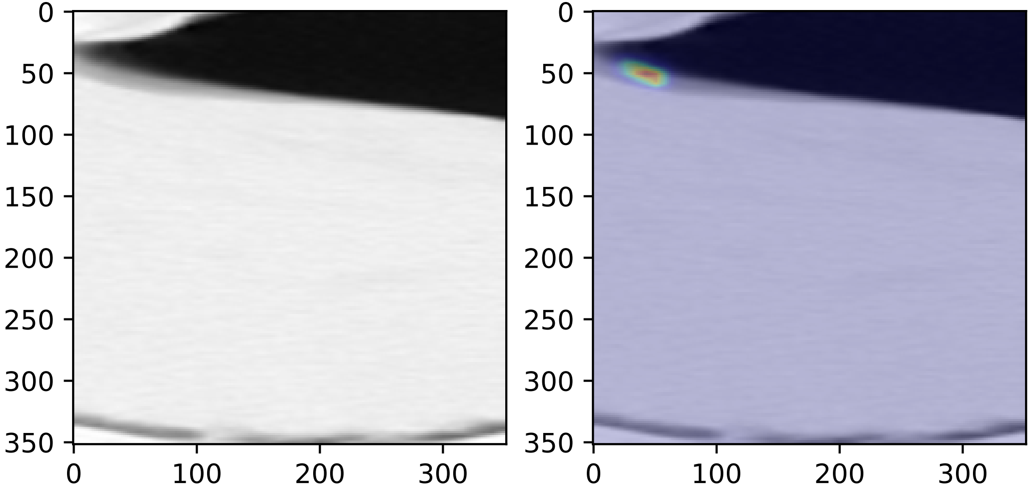

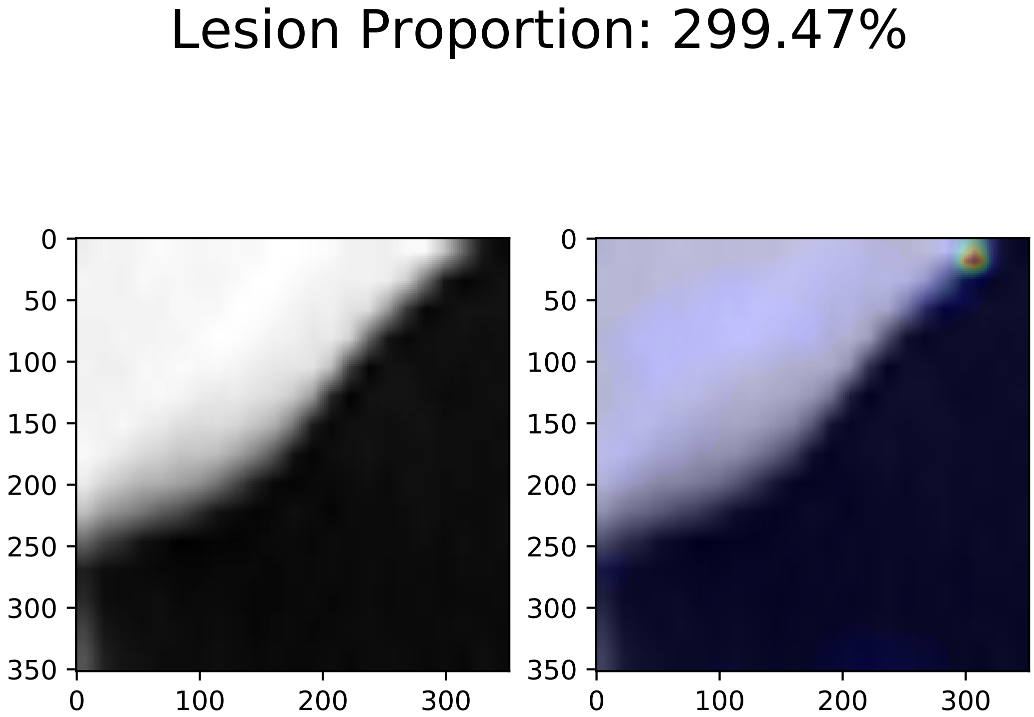
Lesion Proportion: 299.47%
0
50
100
150
200
250
300
350
0
100
200
300
